# Hydrogenation of carbon dioxide to methanol using a homogeneous ruthenium–Triphos catalyst: from mechanistic investigations to multiphase catalysis†
†Electronic supplementary information (ESI) available: Experimental procedures, analytical data. CCDC 1014053. For ESI and crystallographic data in CIF or other electronic format see DOI: 10.1039/c4sc02087a


**DOI:** 10.1039/c4sc02087a

**Published:** 2014-08-27

**Authors:** Sebastian Wesselbaum, Verena Moha, Markus Meuresch, Sandra Brosinski, Katharina M. Thenert, Jens Kothe, Thorsten vom Stein, Ulli Englert, Markus Hölscher, Jürgen Klankermayer, Walter Leitner

**Affiliations:** a Institut für Technische und Makromolekulare Chemie , RWTH Aachen University , Worringerweg 1 , 52074 Aachen , Germany . Email: jklankermayer@itmc.rwth-aachen.de ; Email: leitner@itmc.rwth-aachen.de; b Institut für Anorganische Chemie , RWTH Aachen University , Landoltweg 1 , 52074 Aachen , Germany

## Abstract

The hydrogenation of CO_2_ to methanol can be achieved using a single molecular organometallic catalyst. Whereas homogeneous catalysts were previously believed to allow the hydrogenation only *via* formate esters as stable intermediates, the present mechanistic study demonstrates that the multistep transformation can occur directly on the Ru–Triphos (Triphos = 1,1,1-tris(diphenylphosphinomethyl)ethane) centre. The cationic formate complex [(Triphos)Ru(η^2^-O_2_CH)(S)]^+^ (S = solvent) was identified as the key intermediate, leading to the synthesis of the analogous acetate complex as a robust and stable precursor for the catalytic transformation. A detailed mechanistic study using DFT calculations shows that a sequential series of hydride transfer and protonolysis steps can account for the transformation of CO_2_*via* formate/formic acid to hydroxymethanolate/formaldehyde and finally methanolate/methanol within the coordination sphere of a single Ru–Triphos-fragment. All experimental results of the systematic parameter optimisation are fully consistent with this mechanistic picture. Based on these findings, a biphasic system consisting of H_2_O and 2-MTHF was developed, in which the active cationic Ru-complex resides in the organic phase for recycling and methanol is extracted with the aqueous phase.

## Abstract 2

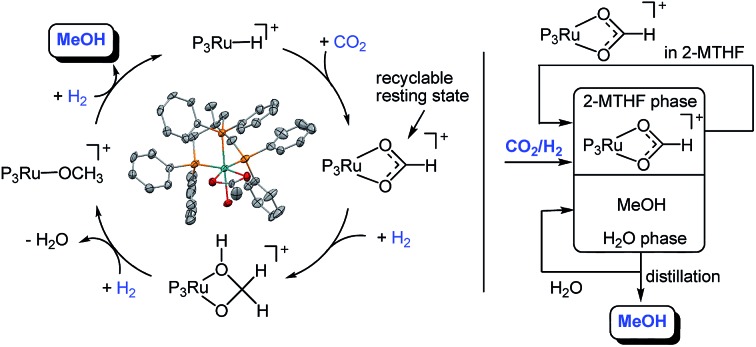
The hydrogenation of CO_2_ to methanol using a recyclable molecular organometallic catalyst in the absence of an alcohol additive is demonstrated for the first time.

## Introduction

The depletion of fossil carbon sources together with the increasing global energy consumption demand alternative ways for the sustainable production of fuels and chemicals. In this context, the usage of carbon dioxide (CO_2_) as an alternative carbon source has seen renewed and increasing interest at the interface of the chemical and energy sectors, as it is a readily available, non-toxic by-product of various large scale industrial processes.^1^–^11^ In particular, the effective hydrogenation of carbon dioxide to methanol could play an important role in supply chains with reduced carbon footprint economies, as methanol can serve as an energy carrier and a versatile basic chemical.^12^–^15^

Today, methanol is produced on a megaton scale from fossil feedstock-based syngas (CO/H_2_).^15^,^16^ These processes utilise heterogeneous catalysts at elevated temperatures (200–300 °C) and pressures (50–100 bar). A certain percentage of CO_2_ is added to the feedstock stream to balance the C/H ratio. The heterogeneously catalysed hydrogenation of pure CO_2_ to methanol has been implemented, capitalising on the specific regional energy and feedstock supply in Iceland, for example.^17^ A detailed picture of the elementary steps and the role of the multi-component catalyst material have been elucidated for the classical Cu/ZnO/Al_2_O_3_ systems, mapping out the complex series of bond cleavage and bond forming processes on the catalyst surface that enable the seemingly simple overall transformation of CO or CO_2_ and hydrogen to methanol.^18^

**Scheme 1:**
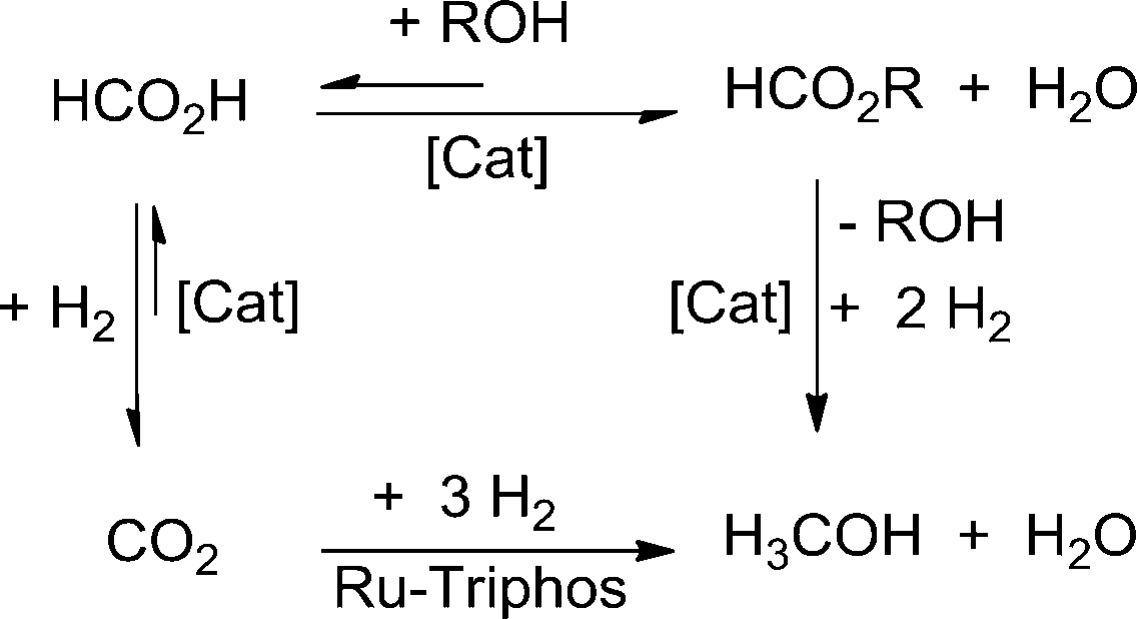
Hydrogenation of CO_2_ to methanol *via* formate esters as proposed by Milstein *et al.*^21^ and previously shown by Sanford/Huff^23^ and Klankermayer/Leitner^24^ (upper pathway), and hydrogenation of CO_2_ to methanol without the need for an alcohol additive as shown in the present report (lower pathway).

In sharp contrast, the hydrogenation of CO_2_ to methanol using a molecularly defined, single-site catalyst has remained elusive up to now. Tominaga *et al.* reported the formation of methanol, together with methane and CO, from CO_2_ hydrogenation using Ru_3_(CO)_12_ in the presence of alkaline iodides under harsh reaction conditions (240 °C, 80 bar). Under these conditions, CO_2_ was reduced to CO, followed by the hydrogenation of CO to methanol and methane.^19^ Later, the catalytic formation of methanol from CO_2_ was reported with organometallic complexes using high energy reduction reagents such as boranes.^20^ With hydrogen, indirect routes *via* the conversion of CO_2_-derived intermediates like organic carbonates, carbamates, formate esters and ureas were proposed (see Scheme 1, upper pathway, for formate esters). The viability of this concept was first demonstrated in the seminal work by Milstein *et al.*, who developed highly efficient ruthenium(ii) pincer complexes for the hydrogenation of these challenging substrates.^21^,^22^ Huff and Sanford reported a three-step, one-pot hydrogenation of CO_2_ to methanol *via* methyl formate as an intermediate using a combination of the Milstein catalyst with two other catalysts.^23^

**Scheme 2:**
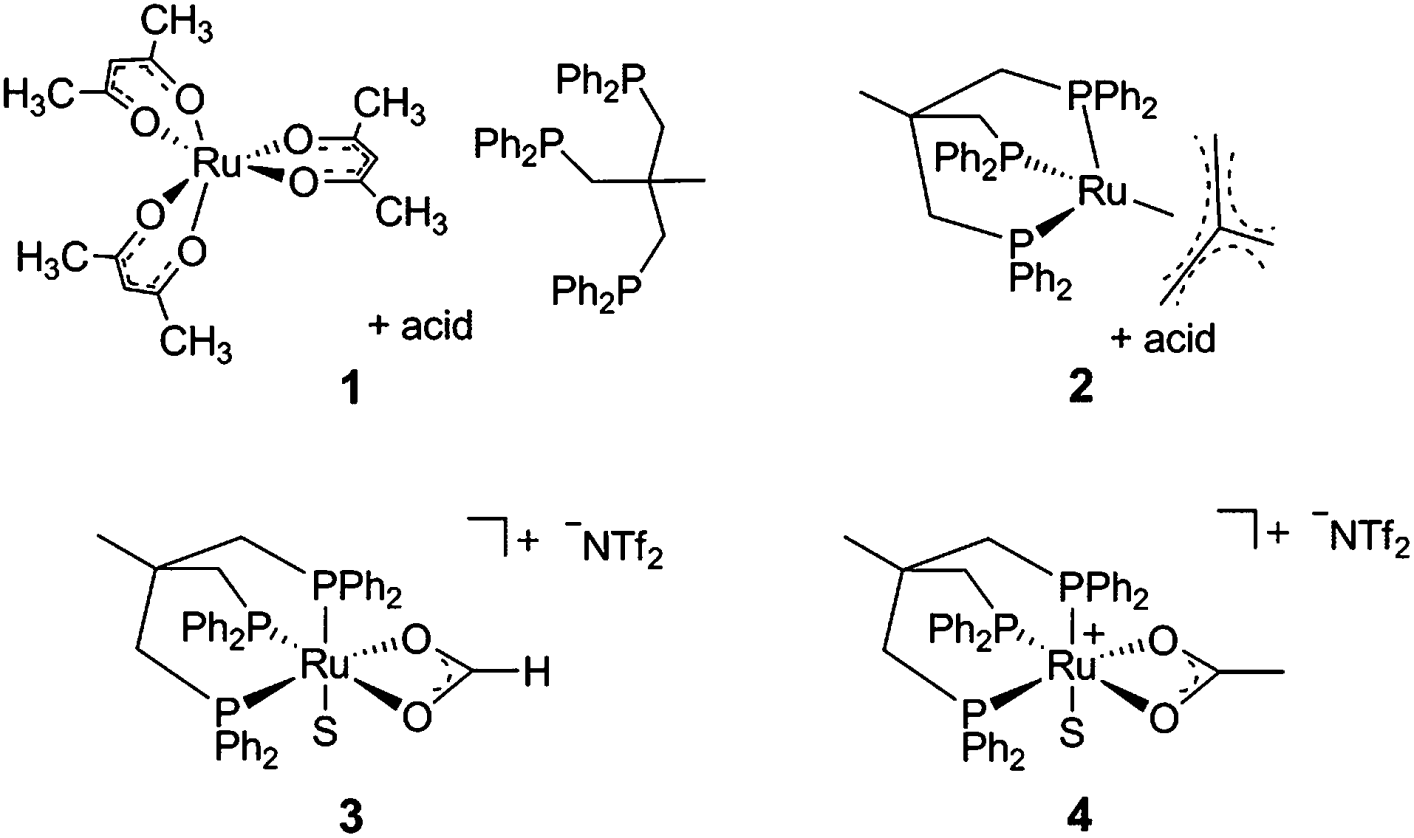
Catalyst precursors **1**, **2** and **4** (S = free coordination site or solvent) for the CO_2_ hydrogenation to methanol and the structure of the catalytically active intermediate **3** (S = solvent).

Most recently, we showed that the sequential reduction *via* a formate ester for the homogeneous hydrogenation of CO_2_ to methanol could be achieved in a fully integrated reaction with a single molecular catalyst based on ruthenium as the central metal and the tridentate ligand Triphos (Triphos = 1,1,1-tris(diphenylphosphinomethyl)ethane).^24^ The catalyst was formed *in situ* from Ru(acac)_3_ and Triphos **1** or using the readily accessible ruthenium(ii)-complex [(Triphos)Ru(TMM)] **2** (TMM = trimethylenemethane) as precursor, both in the presence of an acid co-catalyst (Scheme 2).^25^–^29^

In the present report we disclose for the first time the *hydrogenation of CO*_*2*_*to methanol using a single molecularly-defined homogeneous catalyst without the need for an alcohol additive* (Scheme 1, lower pathway). This fundamental step forward was derived from comprehensive mechanistic investigations concerning the catalyst system **2** which led to the identification of the cationic formate complex [(Triphos)Ru(η^2^-O_2_CH)(S)]^+^ (S = solvent) **3** as a catalytically active intermediate in solution (Scheme 2). Based on this, the analogous cationic acetate complex **4** was developed as a pre-catalyst (Scheme 2). These molecular catalysts allow the homogeneously catalysed formation of methanol using CO_2_ and H_2_ as the sole feedstock with turnover frequencies in the same range as those reported for the active sites of the heterogeneous systems. We also demonstrate the possibility to separate and recycle these catalysts from the MeOH–water product mixture in a biphasic aqueous system using 2-methyl tetrahydrofuran (2-MTHF) as the catalyst phase.

## Results and discussion

### Basic reactivity and identification of the active species

**Fig. 1:**
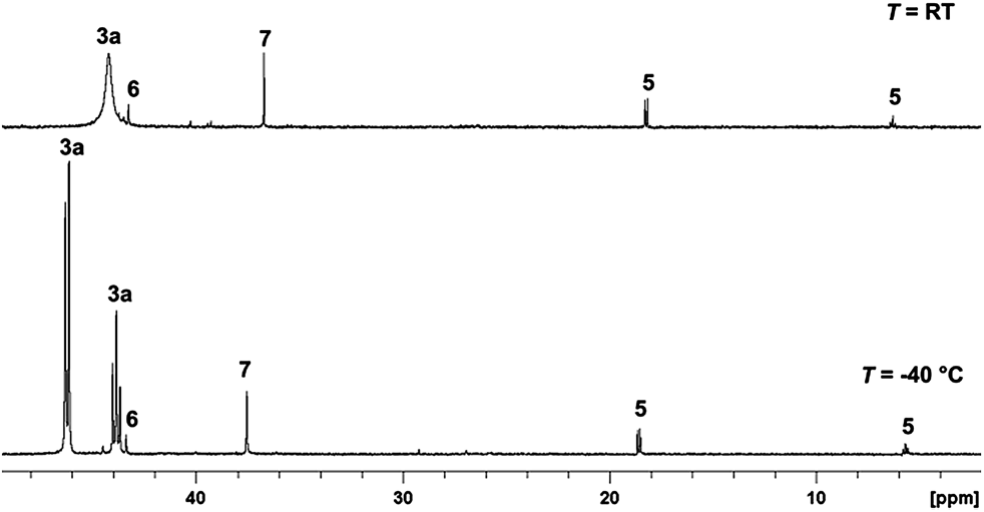
^31^P{^1^H}-NMR spectra (top: at r.t., bottom: at –40 °C) of the reaction solution after CO_2_ hydrogenation to methanol (20 bar CO_2_ + 60 bar H_2_, 140 °C, 1 h) with catalyst **2** (50 μmol) and HNTf_2_ (1 eq.) in *d*_8_-THF (2 mL). **5** = [(Triphos)RuH(CO)_2_]^+^, **6** = [Ru_2_(*μ*-H)_2_(Triphos)_2_], **7** = [Ru_2_(Cl)_3_(Triphos)_2_]^+^, **3a** = [(Triphos)Ru(η^2^-O_2_CH)(THF)]^+^.

Attempting to identify intermediates of the previously reported catalytic reaction sequence with catalyst **2**,^24^,^30^ multinuclear NMR experiments were carried out to monitor the formation of organometallic species upon stepwise addition of the required components. Thus, a solution of the precursor [(Triphos)Ru(TMM)] (**2**) and HNTf_2_ (1 eq.) in *d*_8_-THF was pressurised with CO_2_ (20 bar at r.t.) and H_2_ (60 bar at r.t.), stirred for 1 h at 140 °C, and then transferred to a NMR tube for analysis. Unexpectedly, a sharp signal at 3.27 ppm in the ^1^H-NMR spectrum indicated the catalytic formation of MeOH in the absence of any alcohol additive with a TON (turnover number = mmol MeOH per mmol catalyst) of 35. Thus, one of the species formed under these conditions must have been able to serve as a catalyst for the hydrogenation of CO_2_ to methanol. The ^31^P{^1^H}-NMR spectrum of the clear, yellow solution obtained under these conditions is depicted in Fig. 1, the corresponding ^1^H, ^13^C and 2D-correlation spectra are shown in the ESI.†

Formation of the cationic carbonyl complex [(Triphos)RuH(CO)_2_]^+^ (**5**) was inferred from the characteristic set of a doublet (18.6 ppm, *J* = 28.7 Hz) and triplet (6.3 ppm, *J* = 28.7 Hz) in the ^31^P{^1^H}-NMR spectrum.^31^ Correlation with the hydride signal at *δ* = –6.7 ppm in the [^1^H,^31^P]-HMBC-NMR spectrum and ESI-MS analysis further confirmed this assignment. The content in solution was about 4% according to the integral ratios in the ^31^P{^1^H}-NMR spectrum. The formation of complex **5** corroborates the assumption of cationic complexes as catalytically active species. The carbonyl ligands are most likely formed by decarbonylation of intermediates on the pathway to methanol.^28^,^32^,^33^ Supporting this hypothesis, **5** was synthesised in pure form by stirring complex **2** together with 1 equivalent of HNTf_2_ in ethyl formate and 60 bar H_2_ in the absence of CO_2_ for 24 hours at 140 °C. Testing the isolated complex **5** for its catalytic activity in CO_2_ hydrogenation in the absence of alcohol (standard conditions: *V*(THF) = 2.08 mL, *c*(Ru) = 12 mmol L^–1^, 1 eq. of HNTf_2_, *p*(CO_2_) = 20 bar at r.t., *p*(H_2_) = 60 bar at r.t., *T* = 140 °C, *t* = 24 h) only gave a TON of 4, identifying the formation of **5** as a possible deactivation pathway.

The sharp singlet at 43.3 ppm in the ^31^P{^1^H}-NMR spectrum was correlated with a broad hydride signal at –8.7 ppm in the ^1^H-NMR spectrum by [^1^H,^31^P]-HMBC-NMR. Comparison with the literature data and analysis of the mixture by ESI-MS allowed unambiguous assignment of the dimeric complex [Ru_2_(μ-H)_2_(Triphos)_2_] (**6**), which formed in about 2%.^29^ Using isolated **6** in the CO_2_ hydrogenation reaction under standard conditions resulted in no formation of methanol, revealing the formation of **6** as a second major deactivation pathway.

The small sharp singlet at 36.7 ppm in the ^31^P{^1^H}-NMR spectrum was assigned to [Ru_2_(Cl)_3_(Triphos)_2_]^+^ (**7**) by comparison with the literature data and analysis of the mixture by ESI-MS (6% according to the integral ratios in the ^31^P{^1^H}-NMR spectrum).^34^ A CO_2_ hydrogenation reaction under standard conditions using the [(Triphos)Ru(TMM)] (**2**) precursor but with the addition of 1-butyl-3-methylimidazolium chloride (3 eq.) only gave a TON of 1 after 24 hours. The ^31^P{^1^H}-NMR spectrum of the solution showed the formation of **7**, **5** and [(Triphos)RuH(CO)Cl] (**18**) indicating that Ru–Triphos complexes bearing chloro ligands are generally inactive in this transformation.^35^

The main species accounting for over 85% of the total signal intensity in the ^31^P{^1^H}-NMR spectrum gave rise to a broad singlet at 44.2 ppm, indicating fluxional behaviour at room temperature. Low-temperature NMR at 233 K resulted in the splitting of this signal into a doublet (46.3 ppm, 2P, *J* = 42.5 Hz) and a triplet (43.9 ppm, 1P, *J* = 42.5 Hz). A [^1^H,^31^P]-HMBC-NMR experiment revealed coupling of these signals to a proton signal at 8.7 ppm (bs), which is well in the range of ruthenium coordinated formate.^36^–^38^ A [^1^H, ^13^C]-HMBC-NMR experiment showed the coupling of the proton signal at 8.7 ppm to a singlet at 178.8 ppm in the ^13^C-NMR, further corroborating the formation of a formate-complex.^23^,^39^,^40^ No hydride signals corresponding to this species were detected in the respective correlation NMR spectra.

**Scheme 3:**
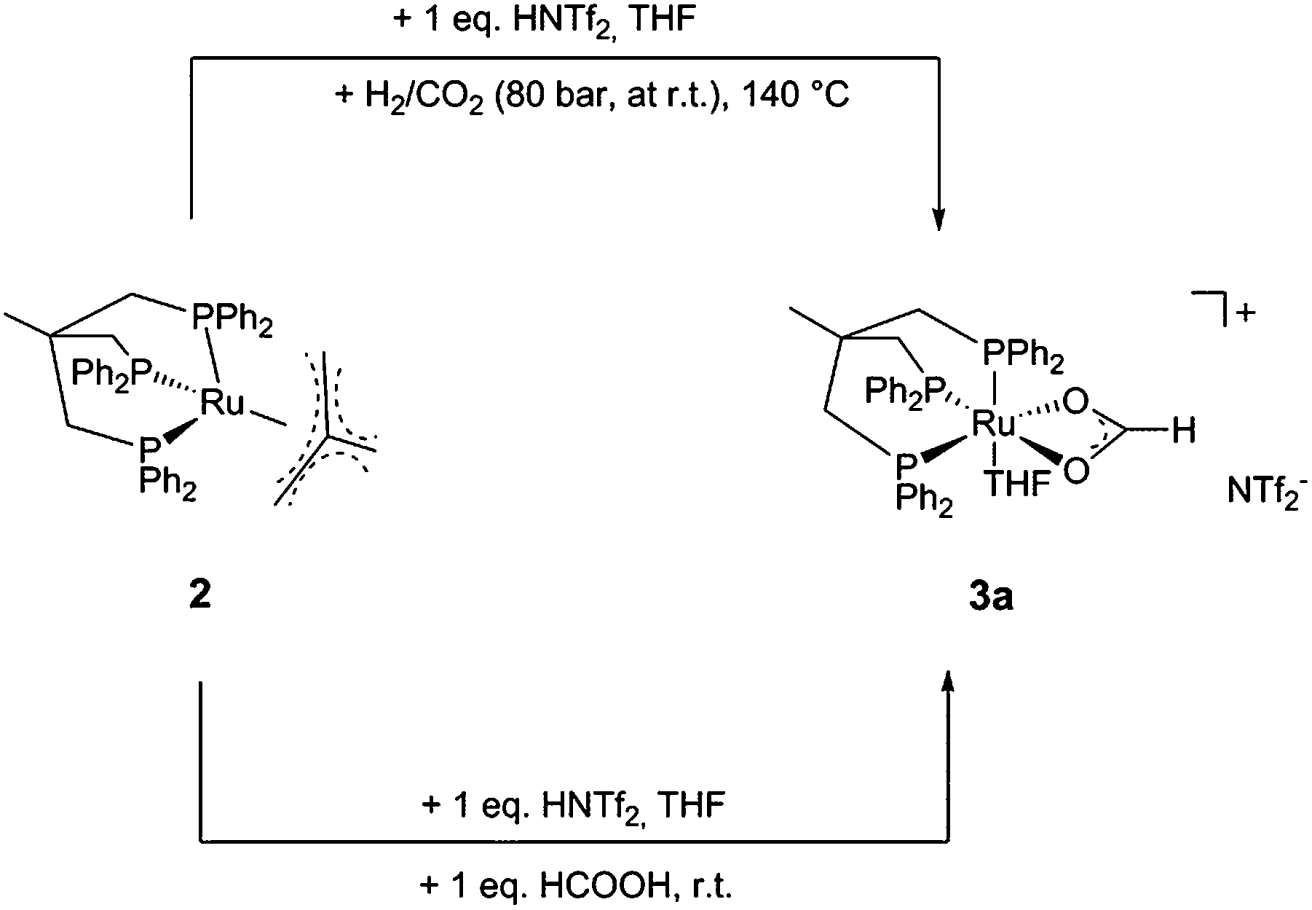
Formation of the catalytically active formate complex **3a** from catalyst precursor **2** in the presence of 1 eq. HNTf_2_ and H_2_/CO_2_ under reaction conditions (upper pathway) and by addition of 1 eq. HNTf_2_ and 1 eq. HCO_2_H in THF.

The same formate complex was generated independently by adding one equivalent of HNTf_2_ to complex **2** in *d*_8_-THF, followed by the addition of one equivalent of HCO_2_H at room temperature. NMR analysis of the crude reaction mixture at room temperature and 233 K showed an identical set of signals in the ^31^P{^1^H}-NMR spectrum in about 80% of the total intensity together with a second, yet unidentified phosphor containing species (singlet at 59 ppm, *ca.* 20% of total intensity), as well as in the ^1^H-NMR spectra (see ESI†). FT-IR analysis of this solution at room temperature showed a *ν*_CO_ stretching mode at 1543 cm^–1^, a typical value for η^2^-coordinated formate.^37^–^40^ Based on these data and on the basis of literature precedence,^37^ we assigned the structure of this complex as [(Triphos)Ru(η^2^-O_2_CH)(THF)]^+^ (**3a**), where the weakly bound solvent molecule THF accounts for the fluxionality at room temperature (Scheme 3).

**Scheme 4:**
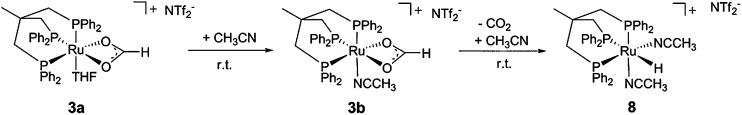
Formation of the acetonitrile formate complex **3b** from **3a** by addition of MeCN to a solution of **3a** in THF and decarboxylation of **3b** to the hydride complex **8** at room temperature.

This interpretation is supported by the formation of a non-fluxional formate complex upon addition of 0.1 mL acetonitrile to the freshly prepared solution of **3a** in 0.5 mL THF at room temperature (d, 42.8 ppm, 2P; t, 29.6 ppm, 1P, *J* = 42.2 Hz; see ESI† for details). FT-IR analysis of this solution at room temperature again showed a *ν*_CO_ stretching mode at 1544 cm^–1^, consistent with the structure [(Triphos)Ru(η^2^-O_2_CH)(MeCN)]^+^ (**3b**). Interestingly, the signals of **3b** decreased over a period of 5 hours at room temperature at the expense of new doublet (47.6 ppm, 2P, *J* = 20.6 Hz) and triplet (5.5 ppm, 1P, *J* = 20.6 Hz) signals. In parallel, the formate signal at 8.7 ppm disappeared with concomitant formation of an upfield hydride signal (dt, –5.5 ppm, *J* = 105.0 Hz, *J* = 19.3 Hz) in the ^1^H-NMR spectrum. These NMR-data are consistent with the decarboxylation of **3b** to give the literature reported complex [(Triphos)Ru(H)(MeCN)_2_]^+^ (**8**) (Scheme 4).^41^ Consequently, the formation of the formate complex **3** from **2** in the presence of HNTf_2_ under CO_2_ and hydrogen pressure is most plausibly explained *via* reversible CO_2_-insertion into the analogous solvent-coordinated cationic Ru–hydride complex as an intermediate.

**Scheme 5:**
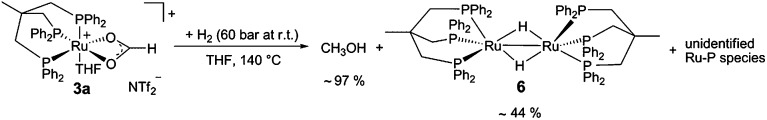
Methanol is formed with high yield by hydrogenation of complex **3a**.

Ruthenium–formate complexes are well known as intermediates in the CO_2_ hydrogenation to formic acid.^42^–^44^ In order to probe whether the formate complex **3** is a kinetically competent intermediate in the hydrogenation of CO_2_ to methanol, a solution of **3a** was prepared from **2**/HNTf_2_ (1 : 1) and HCO_2_H in *d*_8_-THF, pressurised with only 60 bar H_2_, and heated to 140 °C in an external oil bath in a high-pressure NMR tube for 40 minutes (Scheme 5). Indeed, this led to nearly complete conversion (*ca.* 97%) of the coordinated formate to methanol based on ^1^H-NMR analysis (see ESI†). In the corresponding ^31^P-NMR spectra the formation of [Ru_2_(μ-H)_2_(Triphos)_2_] (**6**) in around 44% yield was observed. Furthermore, *in situ* high pressure NMR studies using complex **2** directly under turnover conditions (**2**/HNTf_2_ (1 : 1), *d*_8_-THF, *T* = 80 °C, *p*(H_2_) = 60 bar, *p*(CO_2_) = 20 bar) revealed that complex **3a** was formed immediately after pressurisation and remained the major detectable phosphorus containing species present in solution throughout the reaction. No hydride signals that could be related to an active species were observed in the ^1^H-NMR spectra (see ESI†). These data indicate that the presumed cationic hydride intermediate is too short-lived to be observed on the NMR-time-scale,^37^ but is converted to the observable formate complex [(Triphos)Ru(η^2^-O_2_CH)(THF)]^+^**3a** as the resting state by rapid and reversible CO_2_ insertion into the metal–hydride bond under turnover conditions.^38^,^42^–^44^

**Fig. 2:**
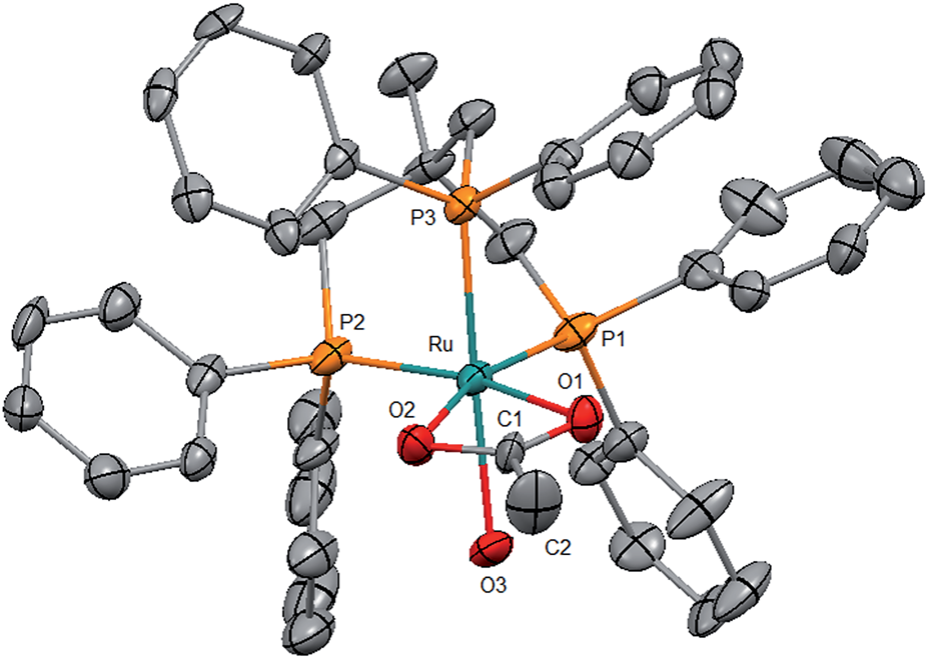
Molecular structure of the cation **4a** (S = H_2_O) in the solid state as derived from single crystal X-ray diffraction (hydrogen atoms are omitted for clarity). Some selected bond lengths (Å): Ru–P1 = 2.245(9); Ru–P2 = 2.255(3); Ru–P3 = 2.253(0); Ru–O1 = 2.171(2); Ru–O2 = 2.208(6); Ru–O3 = 2.204(7).

Identification of the formate complex **3** as the active intermediate suggests that the major role of the acid additive in the catalytic system **2**/HNTf_2_ is the generation of cationic species as the active site upon reductive removal of the TMM-ligand. In order to probe this assumption, we decided to start from an isolated cationic complex precursor. After numerous unsuccessful attempts to isolate complex **3** in a stable form as a solid, we turned our efforts towards the analogous cationic acetate complex. Stirring [(Triphos)Ru(η^2^-OAc)Cl] (**9**)^45^ together with one equivalent of AgNTf_2_ for 3 h at 60 °C in THF led to the precipitation of AgCl. The ^31^P{^1^H}-NMR spectrum showed the selective formation of only one sharp singlet at 44.0 ppm, indicating the formation of a symmetrical complex species. After filtration of the yellow solution over silica and removal of the solvent *in vacuo* a yellow powder was obtained. Characterisation of the material by ^1^H-, ^13^C- and ^19^F-NMR, FT-IR and ESI-HRMS revealed the presence of the cationic [(Triphos)Ru(η^2^-OAc)]^+^ fragment (see ESI†). Crystallisation from dichloromethane layered with pentane gave yellow single crystals of complex **4a** where the open coordination site was saturated with H_2_O from adventitious traces of water (Fig. 2). Thus, the acetate complex in solution can be formulated as [(Triphos)Ru(η^2^-OAc)(S)][NTf_2_] (**4**) with S being a free coordination site or weakly bound solvent molecule.^46^ The formation of dimeric or trimeric species [(Triphos)Ru(μ-OAc)]_*x*_[NTf_2_]_*x*_ could be excluded by using two structurally different Triphos derivatives and stirring an equimolar (12.5 μmol) mixture of [(Triphos)Ru(η^2^-OAc)Cl] (**9**) and [(Triphos-anisyl)Ru(η^2^-OAc)Cl] (**10)** (Triphos-anisyl = 1,1,1-tris{bis(4-methoxyphenyl)phosphinemethyl}ethan) together with AgNTf_2_ (30 μmol) in toluene (1.5 mL) at 60 °C for 5 hours. The toluene was removed *in vacuo*, the residue dissolved in *d*_2_-DCM (0.5 mL), and the mixture analysed by NMR. The ^31^P{^1^H}-NMR spectrum at room temperature showed two singlets at 43.5 and 45.3 ppm in a ratio of nearly 1 : 1 related to [(Triphos)Ru(η^2^-OAc)(S)]NTf_2_ (**4**) and [(Triphos-anisyl)Ru(η^2^-OAc)(S)]NTf_2_ (**11**), respectively. The absence of further signals due to mixed complexes (*e.g.* [Ru_2_(Triphos)(Triphos-anisyl)(μ-OAc)_2_][NTf_2_]_2_) supports the monomeric structure of **4** in solution.^47^

The reactivity of the acetate complex **4** under CO_2_ (20 bar at r.t.) and H_2_ (60 bar at r.t.) pressure was investigated in a high pressure NMR experiment (see ESI†). After 1.5 hours at 80 °C and 1 hour at 140 °C, *ca.* 60% (^31^P{^1^H}-NMR) of **4** was converted to the formate complex **3a** and ethanol from acetate hydrogenation was detected by ^1^H-NMR in the reaction mixture. Methanol was indeed observed in the solution with a TON of 5, confirming that the cationic complex **4** was operating as a molecularly defined direct precursor for the catalytic cycle for CO_2_ hydrogenation without the need of any acid additive.

### Mechanistic pathways on the basis of DFT calculations

**Scheme 6:**
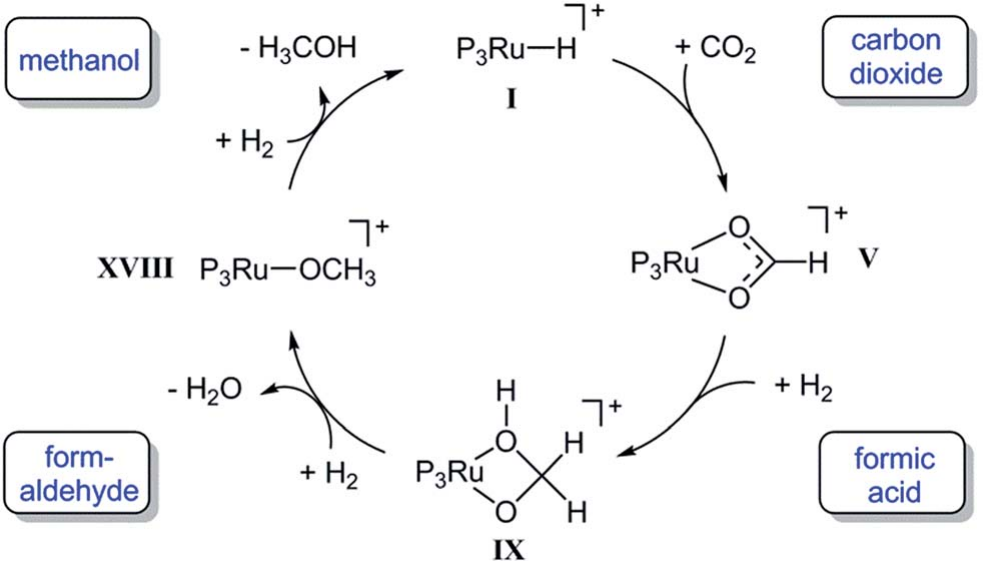
Basic catalytic cycle for the transformation of CO_2_ to methanol at the Ru–Triphos fragment *via* the formic acid and formaldehyde stage through the key intermediates **I**, **V**, **IX**, **XVIII**. P_3_Ru denotes the Triphos–Ru(ii) fragment comprising additional ligands to fill the coordination sphere as discussed in the detailed analysis.

In summary, the experimental results described above clearly demonstrate that the Ru–Triphos framework is able to act as a molecular single-site catalyst for the hydrogenation of CO_2_ to methanol. All observations are in accordance with a stepwise reduction of CO_2_ to methanol *via* the formate anion in the coordination sphere of a homogeneous cationic organometallic complex. Complex **3**, which is accessible from different precursors in the presence or absence of acid co-catalysts, represents the resting state under turnover conditions. Consequently, spectroscopic insight into the subsequent reduction steps cannot be obtained directly. We therefore used DFT calculations to explore possible reaction pathways for this multi-step transformation. Based on our previous investigations on the ruthenium–Triphos system and on recent work by other groups on the catalytic hydrogenation of CO_2_ or methanol reforming, a plausible basic catalytic cycle that reduces carbon dioxide stepwise through the formic acid and formaldehyde stage to methanol *via* the key intermediates **I**, **V**, **IX**, **XVIII** can be formulated as shown in Scheme 6.^21^,^23^,^28^,^30^,^37^,^48^–^51^

Starting from a cationic ruthenium–hydride complex **I**, the migratory insertion of CO_2_ results in the formation of the spectroscopically observed ruthenium–formate species **V**. Reaction with one equivalent of hydrogen leads to reduction beyond the formic acid stage to give the respective ruthenium–hydroxymethanolate species **IX**, which is then transformed to the ruthenium–methanolate complex **XVIII***via* intermediate formation of formaldehyde and consumption of a second equivalent of hydrogen.^30^,^50^,^51^ In the last step, hydrogenolysis of the Ru–OMe unit requires the third equivalent of H_2_ to liberate the product and closes the cycle by reforming the ruthenium–hydride complex **I**. A plausible structure for complex **I** as a starting point of the calculations is the cationic species [(Triphos)Ru(H)(H_2_)(THF)]^+^ that is, for example, most likely to be formed from complex **4** upon hydrogenative removal of the acetate ligand as the initiating step.^28^ The individual steps of the cycle shown in Scheme 6 were therefore analysed in detail from this starting point, whereby the reduction steps are composed of hydride migration/protonolysis events. For clarity, we constrain the discussion here to the most energetically favourable pathways and some particularly relevant alternatives and refer the reader to the ESI† for additional information.

#### Formation of hydroxymethanolate *via* formic acid (**I–IX**, Fig. 3)

**Fig. 3:**
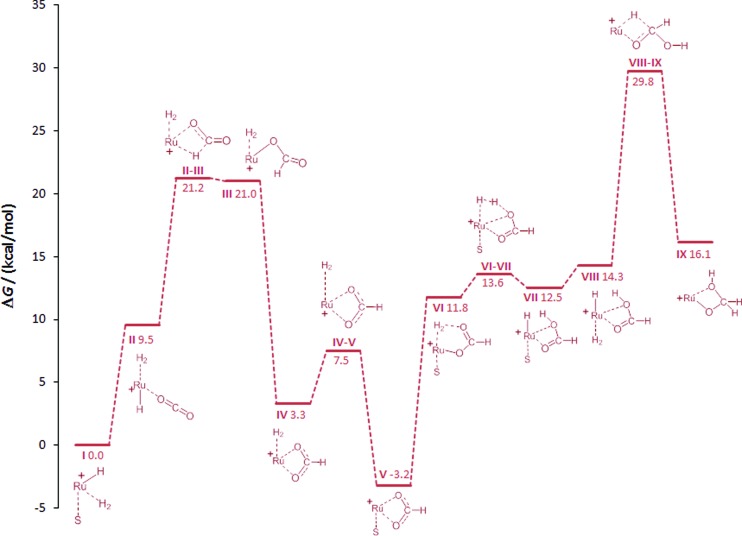
Initial steps of the DFT calculated reaction pathways for the hydrogenation of CO_2_ to methanol at the cationic Ru–Triphos centre, starting from complex **I** (S = THF) as the active species. The Triphos ligand is omitted for clarity.

The insertion of CO_2_ into metal–hydride bonds and subsequent hydrogenolysis of the metal–formate units has been the subject of numerous experimental and theoretical studies in the context of formic acid production. The closest related example to the Triphos-system are Ru(ii)-catalysts bearing three monodentate phosphine ligands whose high efficiency for formic acid production was rationalised in a comprehensive theoretical study by the group of Sakaki.^48^ An analogous route was therefore investigated for the initial step of the current system (Fig. 3).

In the starting complex **I** either the THF molecule or the H_2_ molecule is replaced by CO_2_, generating complexes **II** and **IIa**, respectively. Both compounds are endergonic with respect to the reference point **I** by 8 and 10.2 kcal mol^–1^, respectively. The classical hydride centre in **II** can subsequently be transferred to the carbon atom of CO_2_, passing through transition state **TSII–III**. The barrier is appreciably low (11.7 kcal mol^–1^) placing the **TSII–III** at 21.2 kcal mol^–1^ on the hyper surface. Rotation of the formate species in **III** about the Ru–O and the O–C bonds generates complex **IV** (3.3 kcal mol^–1^), which is significantly more stable than **III**.^43^,^48^ The barrier for the dissociation of H_2_ from **IV** is very low (4.2 kcal mol^–1^) and the exchange of H_2_ in **IV** by solvent generates the stable ruthenium formate complex **V** (–3.2 kcal mol^–1^), which is also the experimentally observed resting state complex **3a**.

In accordance with the work by Sakaki, the proton transfer to the carbonyl C=O bond in the six-membered transition state is also energetically favourable in the Ru–Triphos system.^48^ The change of coordination mode of the formate species in **V** from bidentate to monodentate with the subsequent coordination of H_2_ at the vacant coordination site forms **VI**. The coordinated H_2_ molecule is cleaved heterolytically *via***TSVI–VII** with a very small barrier of 1.7 kcal mol^–1^ to **VII** (12.5 kcal mol^–1^). At this point of the cycle, the generation of formic acid is complete and the Ru–H unit is regenerated for further reduction.

In contrast to the hydrogenation of CO_2_ to formic acid, far less is known about the reduction beyond the formate stage. Only most recently, ruthenium and iridium catalysts have been reported to facilitate this reaction step *via* unprecedented catalytic pathways.^30^,^50^,^52^,^53^ In the Ru–Triphos system, hydride transfer to the coordinated formic acid was found to be energetically accessible only after the solvent in **VII** is replaced by H_2_, generating complex **VIII**. The exchange of solvent by H_2_ is almost thermoneutral placing **VIII** at an energy of 14.3 kcal mol^–1^. The hydride transfer in **VIII** to the carbon atom of formic acid has a barrier of 15.5 kcal mol^–1^, placing **TSVIII–IX** at 29.8 kcal mol^–1^ on the hyper surface. This energetically feasible reaction step forms the ruthenium–hydroxymethanolate species (**IX**) which is the crucial intermediate for the unique performance of the Ru–Triphos system in the hydrogenation of CO_2_ beyond the formic acid stage. Three other energetically less favoured reaction pathways for the formation of hydroxymethanolate from CO_2_, including outer sphere attack of CO_2_, were calculated and are shown in the ESI.† The next key step is the cleavage of the carbon–oxygen bond leading to formaldehyde on the path to methanol.

#### Cleavage of the C–O bond and generation of formaldehyde (**IX–XV**; Fig. 4)

**Fig. 4:**
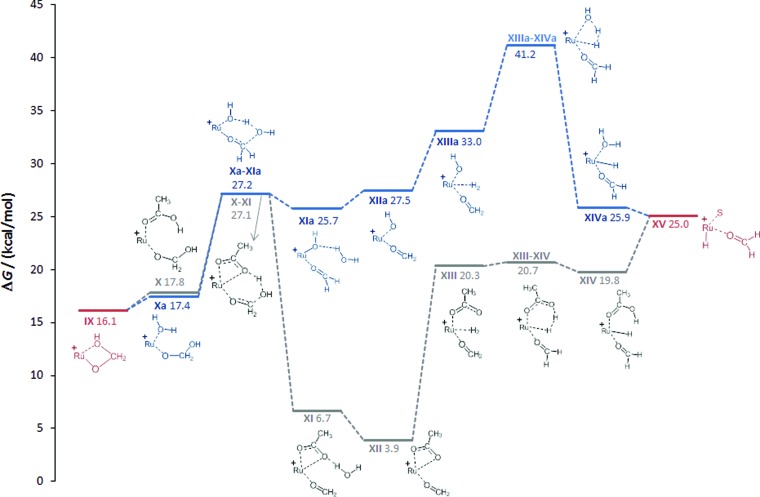
Calculated reaction pathways generating formaldehyde complex **XV***via* medium-assisted proton transfer (acetate: gray; water: blue). The Triphos ligand is omitted for clarity, S = THF.

The conversion between free methanediol and formaldehyde has been extensively explored.^50^ In the coordination sphere of **IX**, the protonolysis of the Ru–O is again required to initiate this process. Firstly, we considered the transfer of protons generated from the acidic Ru–H_2_ units under turnover conditions *via* the reaction medium (Fig. 4). A low energy pathway (grey profile) was calculated if acetic acid was used as the model for carboxylate units as proton shuttles in the presence of catalyst precursors **4** (acetate) or **2** (formate), according to the *in situ* NMR studies. After de-coordination of the hydroxy group in the hydroxymethanolate species **IX**, a molecule of acetic acid coordinates *via* the carbonyl oxygen atom, forming **X** in a practically thermoneutral event. The acetic acid then protonates the hydroxy group of the hydroxymethanolate (**TSX–XI**, 27.1 kcal mol^–1^), with a barrier of 9.3 kcal mol^–1^. Water is loosely coordinated after the reaction (**XI**) and cleaved off, generating **XII**. The dissociation of one of the acetate oxygen atoms of **XII**, changing the acetate coordination from bi- to monodentate, and association of H_2_ generates **XIII**, which is placed at a height of 20.3 kcal mol^–1^. The subsequent heterolytic cleavage of H_2_ during the regeneration of acetic acid is practically barrierless (0.4 kcal mol^–1^) and product **XIV** is only marginally more stable than the reactant. The dissociation of acetic acid and association of solvent generates **XV**. An analogous path using water, which is formed stoichiometrically in the overall hydrogenation sequence, as the proton shuttle gave a significantly higher barrier of 41.2 kcal mol^–1^ (**XIIIa–XIVa**, blue profile). In addition to the external proton transfer, direct protonolysis within the coordination sphere was also investigated (see ESI†).

In essence, the C–O bond cleavage can be achieved from the hydroxymethanolate intermediate **IX** through pathways involving medium-assisted proton transfer or intramolecular proton transfer within the coordination sphere of the Ru-centre. The lowest energy pathway (*ca.* 28 kcal mol^–1^) from the presently investigated alternatives is provided by external proton transfer using carboxylates as the proton shuttle. The intramolecular pathways result in significantly higher barriers (*ca.* 40 kcal mol^–1^), but still provide general viable alternatives that are also in line with the experimental results described below.

#### Hydrogenation of formaldehyde to methanol (**XV-I′**; Fig. 5)

Once the formaldehyde stage is reached in complex **XV** the solvent can be replaced again by H_2_ to arrive at **XVI**. The subsequent migratory transfer of the classical hydride (**TSXVI–XVII**) is almost barrierless at 0.7 kcal mol^–1^ and leads to the methanolate complex **XVII** that is stabilised by an agostic C–H–Ru interaction. The association of a solvent molecule opens the agostic bond to give **XVIII**. The intramolecular proton transfer from the coordinated H_2_ molecule through the “σ-bond metathesis-like”^54^,^55^ four-membered transition state **TSXVIII–XXIV** has an energy barrier of 31.5 kcal mol^–1^. Finally, the reaction product methanol dissociates from the corresponding complex **XXIV** and for completeness we calculated the barrier **TSXXIV-I′** for H_2_ association, which is below 10 kcal mol^–1^, indicating that this process is facile. It should be noted that **I′** lies 14.1 kcal mol^–1^ above the reference point, indicating that the overall reaction is endergonic under the boundary conditions of the calculation model. The inclusion of solvent effects, however, predicts the reaction to be exergonic, in accordance with the experimental observation and standard state thermodynamics (see ESI† for details).

**Fig. 5:**
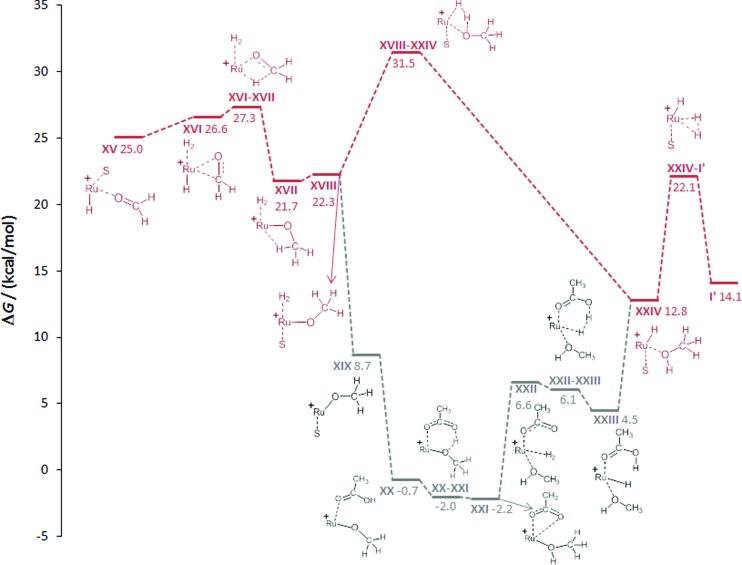
Calculated reaction pathways for the hydrogenation of formaldehyde generating methanol *via* intramolecular proton transfer (red) and carboxylate assisted proton transfer (acetic acid: grey). The Triphos ligand is omitted for clarity, S = THF.

Similar to the protonolysis of the hydroxymethanolate complex, carboxylate-assisted proton transfer provides an alternative low energy pathway. Substitution of the hydrogen molecule in **XVIII** by acetic acid is energetically favourable to give **XX** and the subsequent protonation of the methanolate oxygen atom *via***TSXX–XXI** has no significant barrier and results in the acetate–methanol complex **XXI** (–2.2 kcal mol^–1^). Hydrogen addition (**XXII**) and carboxylate-assisted heterolytic cleavage of H_2_ to regenerate acetic acid (**XXIII**) can occur through a six-membered transition state, rendering this more facile than the direct heterolytic cleavage of the Ru–methanolate unit. The dissociation of acetic acid leads to **XXIV** at which point the two pathways merge again.

Overall, the results of the DFT calculations demonstrate the possibility of a stepwise reduction of CO_2_ to methanol in the coordination sphere of a single Ru–Triphos centre. The individual reduction steps occur by migratory transfer of classical Ru–hydride ligands, exhibiting low to moderate barriers in all cases. The protonolysis steps of the resulting Ru–O bonds can occur intramolecularly *via* heterolytic cleavage of coordinated H_2_ molecules. External proton transfer assisted by carboxylate groups present under turnover conditions may lower the corresponding barriers significantly.

### Parameter variation and catalyst recycling in a biphasic system

After demonstrating the principle of the possibility for the catalytic hydrogenation of CO_2_ to methanol in the absence of an alcohol additive, the performance of the Ru–Triphos precursor systems **2** and **4** was evaluated further by the systematic variation of key reaction parameters and the results were corroborated for consistency with the mechanistic proposal (Table 1).

**Table 1:** Hydrogenation of carbon dioxide to methanol in the absence of alcohol additive^*a*^

Entry	Cat	Acid (eq.)	*T* [°C]	*p*_CO_2__/*p*_H_2__^*b*^ [bar/bar]	TON^*c*^
1	**2**	HNTf_2_ (1.0)	140	20/60	228
2	**2**	—	140	20/60	8
3	**4**	—	140	20/60	165
4	**4**	HNTf_2_ (0.5)	140	20/60	156
5	**2**	HNTf_2_ (1.5)	140	20/60	196
6	**2**	HNTf_2_ (2.0)	140	20/60	181
7	**2**	*p*-TsOH (1.0)	140	20/60	135
8	**2**	HNTf_2_ (1.0)	120	20/60	169
9	**2**	HNTf_2_ (1.0)	100	20/60	67
10	**2**	HNTf_2_ (1.0)	80	20/60	24
11	**2**	HNTf_2_ (1.0)	140	10/30	78
12	**2**	HNTf_2_ (1.0)	140	30/90	367
13	**2**	HNTf_2_ (1.0)	140	20/80	301
14	**2**	HNTf_2_ (1.0)	140	20/100	348
15^*d*^	**2**	HNTf_2_ (1.0)	140	20/60	335
16^*e*^	**2**	HNTf_2_ (1.0)	140	20/60	442
17^*d*^	**12**	HNTf_2_ (1.0)	140	20/60	256

^*a*^Reaction conditions: 25 μmol [Ru], 2.08 mL THF, 24 h.

^*b*^At room temperature.

^*c*^TON = mmol MeOH per mmol catalyst.

^*d*^12.5 μmol [Ru].

^*e*^6.3 μmol [Ru].

Firstly, the catalyst systems **2** and **4** were compared under a standard set of reaction conditions (*V*(THF) = 2.08 mL, *c*(Ru) = 12 mmol L^–1^, *p*(CO_2_) = 20 bar at r.t., *p*(H_2_) = 60 bar at r.t., *T* = 140 °C, *t* = 24 h). Using **4** as the catalyst precursor for the CO_2_ hydrogenation in THF gave a TON of 165 in the absence of any additives (Table 1, entry 3). Using an additional 0.5 eq. HNTf_2_ did not show an increased TON (Table 1, entry 4). In contrast, precursor **2** showed only a very poor performance in the absence of the acid additive (Table 1, entry 2). However, a TON of 228 was obtained when conducting the CO_2_ hydrogenation reaction with catalyst **2** and 1 eq. of HNTf_2_ (Table 1, entry 1). The need for an acid additive in the case of precursor **2** is consistent with the formation of the cationic species [(Triphos)Ru(H)(H_2_)(S)]^+^, which is the catalytically active species **I** used as the starting point in the calculated catalytic cycle.

The lower TON obtained when using the acetate complex **4** instead of **2**/HNTf_2_ (1 : 1) under otherwise identical conditions can be explained by the less efficient initiation with **4** due to the more difficult hydrogenation of the acetate groups to form the common intermediate **3a** (*vide supra*). This distinct reactivity is also reflected in the catalytic experiments for the hydrogenation of the corresponding free acids: using **2** together with 1 eq. HNTf_2_, 100 equivalents of formic acid could be fully converted to methanol at a hydrogen pressure of 60 bar (at r.t.) and a reaction temperature of 140 °C within 24 hours, whereas a reaction temperature of 180 °C was necessary for the full conversion of 100 equivalents acetic acid to ethanol (*c*(Ru) = 12.5 mmol L^–1^, 2.0 mL THF). The efficient hydrogenation of formic acid under these conditions is in accordance with the proposed catalytic cycle shown in Scheme 6. To complete the picture, the hydrogenation of 100 equivalents of paraformaldehyde was also assessed and a full conversion was indeed achieved with the same catalytic system (*c*(Ru) = 12.5 mmol L^–1^, 2.0 mL THF, 0.2 mL H_2_O, 60 bar H_2_ at r.t., 140 °C, 24 h). Again, the formation of formate complex **3a** was observed in the ^31^P{^1^H}-NMR spectrum, indicating the full reversibility of the catalytic cycle.

The lack of activity with catalyst **2** in the absence of acid can be directly corroborated with the formation of the neutral complex [(Triphos)Ru(H)_2_CO] (**12**), which was observed as the almost exclusive species present in solution by ^31^P{^1^H}-NMR spectroscopy under these conditions (see ESI†).^28^ The protonation of this complex with strong protic acids was shown by Zanobini *et al.* to lead to the formation of the complex [(Triphos)Ru(CO)(H)(H_2_)]^+^ (**13**), a cationic structure closely resembling the active hydride species **I** inferred above.^56^ Using the isolated complex **12** together with 1 equivalent of HNTf_2_ in THF indeed resulted in an active catalyst for the CO_2_ hydrogenation reaction yielding a TON of 256 after 24 h (Table 1, entry 17), which is about 76% of the TON obtained using the catalyst **2**/HNTf_2_ under identical conditions (Table 1, entry 15). Again, formation of the formate intermediate **3a** was observed when the solution was analysed by ^31^P{^1^H}-NMR (see ESI†).

Variation of the amount of HNTf_2_ added to complex **2** revealed a maximum observed TON at a 1 : 1 ratio (Table 1, entries 5 and 6) corresponding to the stoichiometric ratio required for the reductive removal of the TMM-ligand leading to **I**. Using **2** together with 1 eq. *p*-TsOH instead of HNTf_2_ gave a lower TON of 135 (Table 1, entry 7) under otherwise identical conditions. NMR-analysis of the reaction solution after 1 h reaction time showed the formation of the formate species **3a** as the major component in solution in both cases (see ESI†). However, [(Triphos)Ru(*p*-TsO)_2_] (**14**) was also present in about 15% as indicated by a broad singlet at 38.7 ppm in the ^31^P{^1^H}-NMR spectrum measured at room temperature, which split up into a triplet (*δ* = 42.0 ppm, *J* = 47.5 Hz) and doublet (*δ* = 36.1 ppm, *J* = 47.5 Hz) when measured at 233 K in *d*_8_-THF. This assignment was supported by mass spectrometry (FAB) and the independent generation of **14** by addition of 2 equivalents of *p*-TsOH to **2** in THF at room temperature. Thus, the presence of even weakly-coordinating anions in the reaction mixture hampers the formation of the formate species **3a**, explaining the preferred choice of HNTf_2_ as the acid additive.

After identifying the system **2**/HNTf_2_ (1 : 1) as the most practical and effective catalyst precursor so far, the influence of some key reaction parameters on the TON after 24 hours reaction time was assessed. Lowering the catalyst concentration together with the acid concentration from 12 μmol mL^–1^ to 6 μmol mL^–1^ and further to 3 μmol mL^–1^ resulted in a significant increase in TON from 228 to 335 and 442, respectively (Table 1, entries 1, 15 and 16). Although final conclusions cannot be drawn before a detailed kinetic analysis, the formation of the dimeric complex **6** as part of the deactivation mechanism is in line with this trend. Decreasing the reaction temperature to 120 °C, 100 °C and 80 °C resulted in reduced TONs of 169, 67 and 24 (Table 1, entries 8–10), respectively. Variation of the total pressure while maintaining the stoichiometric ratio of *p*(CO_2_)/*p*(H_2_) = 1/3 from 40 bar to 80 bar and 120 bar resulted in an increase of the obtained TONs from 78 to 228 and 367 (Table 1, entries 11, 1 and 12), respectively. Using an excess of H_2_ (20 bar CO_2_ + 80 bar or 100 bar H_2_) resulted in largely increased TONs of 301 and 348 (Table 1, entries 13 and 14), respectively. In the latter case about 40% of the totally available carbon feedstock CO_2_ was converted to methanol, as calculated from the amount of MeOH formed (8.7 mmol) and the amount of CO_2_ initially charged (22.1 mmol, determined by weight).

**Fig. 6:**
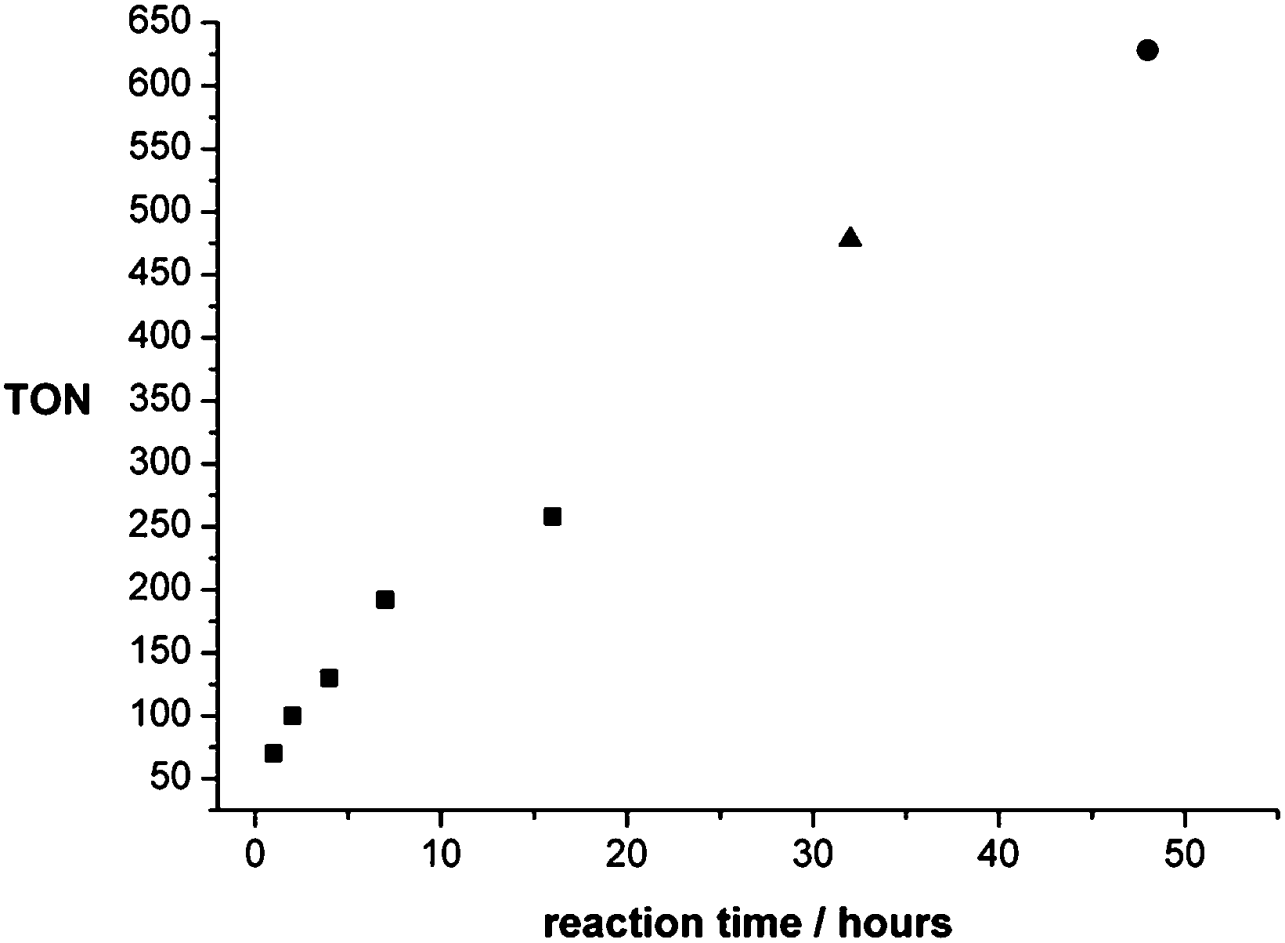
Conversion/time profile of the hydrogenation of CO_2_ to methanol using catalyst **2** (12.5 μmol catalyst **2**; 12.5 μmol HNTf_2_, 20 bar CO_2_ + 60 bar H_2_ at r.t.; 140 °C reaction temperature; 2.08 mL THF), as obtained from batch experiments terminated at the given reaction times. In the case of the reaction terminated after 32 h the autoclave was re-pressurised to the initial pressure with *p*(CO_2_)/*p*(H_2_) = 1/3 after 16 h (▲). In the case of the reaction terminated after 48 h the autoclave was re-pressurised after 16 h and again after 32 h ([black circle]).

A conversion/time profile of the CO_2_ hydrogenation to methanol in THF using **2**/HNTf_2_ (1 : 1, 12.5 μmol; otherwise standard conditions) was mapped out by the termination of batch reactions after different reaction times (Fig. 6). The reaction started without any pronounced induction period, reaching a TON of 70 after just 1 hour. This corresponds to an initial turnover frequency (TOF) of 70 h^–1^ that is well in the range of the activity of the active sites in the state-of-the-art heterogeneous Cu/ZnO-based catalysts.^18^ Methanol formation continued smoothly, reaching a TON of 258 after 16 hours. At this point the pressure in the reactor vessel had dropped from an initial 120 bar to 72 bar due to the consumption of the reactive gases. Therefore, a reaction was conducted for 32 h where the reactor was re-pressurised to the initial pressure with *p*(CO_2_)/*p*(H_2_) = 1/3 after 16 hours leading to a TON of 478. Finally, a reaction was run for 48 hours with re-pressurisation to the initial pressure with *p*(CO_2_)/*p*(H_2_) = 1/3 after 16 hours and again after 32 hours, yielding a total TON of 603. These experiments clearly indicate the high stability of the active catalyst, resulting in a nearly linear increase of the TON under isobaric conditions. The absence of an induction period indicates that the presence of methanol is not enhancing the rate of catalysis under these conditions. This does not rule out the possibility that the reaction also partly proceeds *via* a cascade reaction involving methyl formate as an intermediate once methanol has been formed, as catalyst **2** is able to promote the hydrogenation of alkyl formates to methanol.^24^

**Fig. 7:**
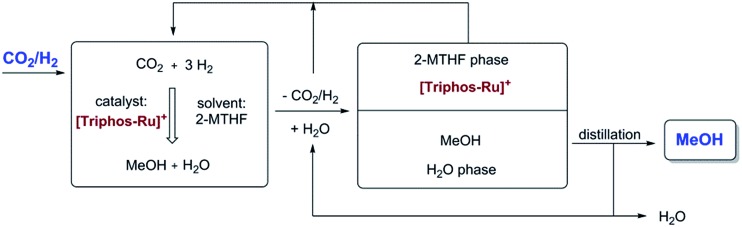
Aqueous biphasic system for recycling of the cationic Ru–Triphos catalyst in the organic 2-MTHF phase and removing the product MeOH in the aqueous phase for downstream processing.

As all of the previous results were consistent with the cationic Ru–Triphos formate complex **3** as the resting state in this process, we explored strategies for the recycling of the catalyst in its active form. Considering the various options for the isolation of the product MeOH from the homogeneous catalyst, distillation seemed an obvious possibility. However, the hydrogenation of CO_2_ yields a stoichiometric amount of water, which is the least volatile component in the THF–MeOH–water product mixture. Thus, it would accumulate upon stripping of the MeOH product, ultimately becoming the limiting factor even if the catalyst would be thermally stable for recycling. Multiphase catalysis offered an alternative strategy, where the separation is based on differences in solubility rather than volatility. For the current process, an aqueous biphasic system was envisaged, where the catalyst is retained and recycled in an organic phase, whereas the product is removed in an aqueous phase for downstream processing.^57^,^58^ Substitution of the solvent THF with 2-methyl tetrahydrofuran (2-MTHF) opened up the possibility for the realisation of such a biphasic reaction/separation system as 2-MTHF has a miscibility gap with water.^27^ All material streams can be recycled internally in such a process scheme, also providing the possibility for continuous-flow operation (Fig. 7).

**Fig. 8:**
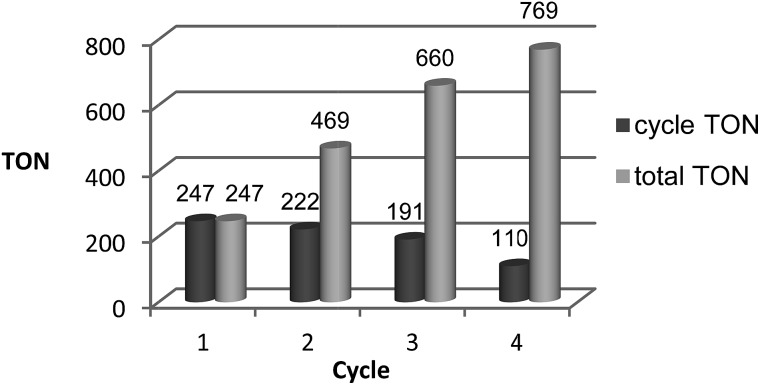
Recycling of the catalyst system **2** (12.5 μmol complex **2**, **2**/HNTf_2_ = 1 : 1) using 2-MTHF (2.0 mL) as the solvent and water (2.0 mL) as the extracting agent in an aqueous biphasic system. Each cycle was run for 16 h (20 bar CO_2_ + 60 bar H_2_ at r.t.; 140 °C reaction temperature). The TONs obtained per cycle are shown in dark grey, the total TONs summing up the cycles are shown in light grey.

Assessing the partitioning of methanol in a 2-MTHF–water biphasic mixture showed that 80% of 0.2 mL MeOH can be isolated from 1.0 mL of 2-MTHF using 1.0 mL water in a single extraction step. Application of 2-MTHF as the solvent for the catalytic reaction under standard conditions (*p*(CO_2_) = 20 bar/*p*(H_2_) = 60 bar at r.t., *T* = 140 °C, *V*(solvent) = 2.08 mL) was also found to be possible without any problems for the catalyst **2** (25 μmol complex **2**, **2**/HNTf_2_ = 1 : 1), albeit with a somewhat lower TON of 186 after 24 h as compared to THF. To validate the combination of reaction and separation, a reaction with **2** (12.5 μmol complex **2**, **2**/HNTf_2_ = 1 : 1) in 2-MTHF (2.0 mL) was terminated after 16 hours, the reaction mixture extracted by the addition of 2.0 mL H_2_O, and the orange catalyst/2-MTHF phase was recycled to the autoclave after simple decantation. A small amount of fresh 2-MTHF (0.25 mL) was added to compensate for any loss of 2-MTHF with the product phase. The aqueous layers were analysed for MeOH content by quantitative ^1^H-NMR in *d*_6_-acetone using mesitylene as the standard, and only the concentration in the aqueous streams was used for the calculation of the apparent TON. As seen from Fig. 8, the catalyst system **2**/HNTf_2_ could be recycled three times, resulting in a total TON of 769 after 4 cycles. The TON per cycle was reduced significantly especially between cycles three and four, but still nearly 50% of the initial productivity was retained in this non-optimised sequence.

## Conclusions

The results of this study demonstrate for the first time the hydrogenation of CO_2_ to methanol using a single organometallic catalyst in a homogenous solution without the need for an alcohol additive. The experimental and theoretical results are consistent with a mechanistic picture where this unprecedented transformation occurs at a cationic Triphos–Ru fragment as a molecularly-defined active site. The cationic formate complex [(Triphos)Ru(η^2^-O_2_CH)(S)]^+^ (**3**) (S = solvent) represents the resting state of the catalytic cycle under turnover conditions and can be obtained from various stable and readily available catalyst precursors. Through a series of hydride transfer and protonolysis steps, the CO_2_ reduction can pass through the formic acid and formaldehyde stages within the coordination sphere of a single ruthenium centre. The barriers for the proton transfer steps may be significantly lowered if assisted by the reaction medium. The active species shows remarkable stability, with decarbonylation and dimerisation as potential deactivation mechanisms. Recycling of the catalyst is possible in the aqueous biphasic system 2-MTHF–water, opening the possibility for continuous-flow operation.

The Triphos–ruthenium system is the very first homogeneous catalyst to enable this transformation. The facial coordination of the Triphos ligand imposes a favorable geometrical arrangement for the hydride transfer to carboxylate units in general (see ESI† for a comparison of facial with meridional arrangement).^28^ Furthermore, the heterolytic cleavage of hydrogen offers low-energy pathways for the protonolysis of Ru–O units during the regeneration of the hydride ligand. Together with the high thermal stability of Triphos–ruthenium complexes, these features seem to play an important role in the reduction of CO_2_ beyond the formate stage with this catalyst. Further developments on the basis of the methodological approach of organometallic chemistry *e.g.* by systematic ligand variation based on the current mechanistic hypothesis are likely to produce even more active and stable systems. Already at this early stage of the development, turnover frequencies per Ru-centre are in the same range as for the active sites in traditional heterogeneous catalysts for methanol synthesis. The possibility to operate under multiphase conditions provides opportunities to overcome the limitations in productivity inherent to batch or repetitive batch operation. Therefore, we believe that the results of this study provide not only fundamental mechanistic insights into the activation and transformation of CO_2_ and H_2_ in organometallic chemistry, but also open promising targets for research at the interface of molecular and engineering sciences.

## Supplementary Material

Supplementary information

Crystal structure data
