# Supplementary material for: Hydrogenation of carbon dioxide to methanol using a homogeneous ruthenium–Triphos catalyst: from mechanistic investigations to multiphase catalysis
Source: Chem Sci. 2014 Aug 27;6(1):693–704. doi: 10.1039/c4sc02087a (PMC6085670; doi:10.1039/c4sc02087a)
Supplement: Supplementary file 1 [file SC-006-C4SC02087A-s001.pdf]

Electronic Supplementary Material (ESI) for Chemical Science.
This journal is © The Royal Society of Chemistry 2014

Supporting information

Direct Hydrogenation of Carbon Dioxide to Methanol using a Homogeneous Ruthenium-Triphos Catalyst: From Mechanistic Investigations to Multiphase Catalysis

Sebastian Wesselbaum, Verena Moha, Markus Meuresch, Sandra Brosinski, Katharina M. Thenert, Jens Kothe, Thorsten vom Stein, Ulli Englert, Markus Hölscher, Jürgen Klankermayer, and Walter Leitner**

S. Wesselbaum, V. Moha, M. Meuresch, S. Brosinski, K. M. Thenert, J. Kothe, T. vom Stein, Dr. M. Hölscher,
Prof. Dr. J. Klankermayer, Prof. Dr. W. Leitner
Institut für Technische und Makromolekulare Chemie
RWTH Aachen University
Worringerweg 1, D-52074 Aachen, Germany
E-Mail: jklankermayer@itmc.rwth-aachen.de; leitner@itmc.rwth-aachen.de

Prof. Dr. Ulli Englert
Institut für Anorganische Chemie
RWTH Aachen University
Landoltweg 1, D-52074 Aachen, Germany

Table of contents

1 General 3

2 Synthesis and characterisation of [(Triphos)Ru(η^2-OAc)(S)]NTf_2 4: 4

3 Synthesis of 1,1,1-tris{bis(4-methoxyphenyl)phosphinomethyl}ethan (Triphos-Anisyl) 15 9

4 Synthesis of [Ru(η^2-OAc)Cl(Triphos-Anisyl)] 10 11

5 General procedure for CO_2 hydrogenation experiments 13

6 Spectroscopic investigations 15

6.1 NMR-spectroscopic analysis of the reaction mixture using complex 2 together with $HNTf_2$ 15

6.2 Generation and analysis of the formate complex 3 17

6.3 Conversion of formate species 3a to methanol 22

6.4 In-situ NMR study of the CO_2 hydrogenation reaction using complex 2 with $HNTf_2$ 24

6.5 NMR-spectroscopic analysis of the reaction mixture using complex 2 together with *p*-TsOH 27

6.6 NMR-spectroscopic analysis of the reaction mixture using complex 2 in the absence of acid 29

6.7 NMR-spectroscopic analysis of the reaction mixture using complex 12 together with $HNTf_2$ 30

6.8 In-situ NMR study of the CO_2 hydrogenation reaction using complex 4 32

7 Repetitive-Batch experiments using 2-MTHF/H_2O 35

7.1 Extraction of MeOH from 2-MTHF with water 35

7.2 Repetitive batch experiment using complex 2 together with $HNTf_2$ in 2-MTHF 35

8 Additional catalysis results 36

9 DFT Calculations 37

9.1 Computational Details 37

9.2 Hydrogenation of CO_2 to Methanediolate 38

9.3 Cleavage of the C-O bond and generation of formaldehyde – Protonolysis within the coordination sphere of Ru 38

9.4 Influence of the coordination geometry (facial vs. meridional) 40

9.5 Calculated Energies 42

10 X-Ray crystal structure of 4a 46

11 Literature 46

12 Appendix: XYZ Coordinates of the calculated structures (Gas) 48

1 General

Safety advice: High-pressure experiments with compressed gases represent a significant safety risk and must be conducted only following appropriate safety procedures and in conjunction with the use of suitable equipment.

For complex synthesis and catalytic experiments, moisture and oxygen were excluded by working in a glove box or by using Schlenk techniques. Argon 4.8 (Messer, Germany) was used as inert gas. Glassware was dried under vacuum with a heat gun, evacuated and refilled with argon at least three times. All solvents were purified by distillation prior to use. Tetrahydrofuran, toluene and pentane were degassed by bubbling argon with a frit, dried by passing over activated alumina in steel columns and stored over molecular sieves. Acetonitrile, dioxane, and 2-methyltetrahydrofuran were degassed by distillation under argon and dried over molecular sieves. Water was degassed by bubbling argon with a frit. All reagents were commercially supplied by Aldrich, Fluka, Alfa Aesar and Heraeus Precious Metals and used as received unless stated otherwise. Reaction gases hydrogen (5.0) and carbon dioxide (4.6) were supplied by Linde and PraxAir and used without further purification. The complexes [(Triphos)Ru(TMM)] **2**[1], [Ru(η^2-OAc)Cl(Triphos)] **9**[2], [(Triphos)Ru(H)$_2$CO] **12**[3] and [Ru$_2$(μ-H)$_2$(Triphos)$_2$] **6**[4] were synthesised according to literature procedures.

NMR spectra were recorded on samples in deuterated solvents with commercial spectrometers Bruker AV-600, AV-400 or AV-300 at room temperature unless stated otherwise. Chemical shifts δ are given in ppm relative to tetramethylsilane (^{1}H and ^{13}C) and 85% phosphoric acid (^{31}P). First order spin multiplicities are abbreviated as singlet (s), doublet (d), triplet (t), quadruplet (qua), quintet (qui), sextet (sext) and septet (sep). Couplings of higher order or overlapped signals are denoted as multiplet (m), broadened signals as (br). First order coupling constants *J* are given in Hz. Assignments are based on attached proton tests (ATP) and 2-D correlation spectroscopy (HSQC, HMQC, HMBC).

Gas chromatography was performed on a *Trace GC* gas chromatograph (Thermo Scientific) equipped with a SSL Inlet (250 °C, Split 83 mL/min), a FS-Innopeg-2000 column (60 m, inner diameter 0.25 mm, film thickness 0.25 µm) and a flame ionisation detector (250 °C) using helium as carrier gas and a temperature program: 10 min isothermal at 50 °C, ramp to 200 °C (8 °C/min), 30 min at 200 °C.

2 Synthesis and characterisation of [(Triphos)Ru(η^2-OAc)(S)]NTf$_2$ 4:

[Ru(η^2-OAc)Cl(Triphos)] **9** (123.0 mg, 0.15 mmol) and AgNTf$_2$ (60.0 mg, 0.155 mmol, 1.03 eq.) were stirred in tetrahydrofuran (12 mL) at 60 °C for 3 hours. The solution was filtered over celite to remove the greyish precipitate and the solvent was removed in vacuo to give a yellow-orange solid (84 mg, yield = 53 %). NMR-spectra of the isolated complex **4** are shown in Figures 1 and 2.

Characterisation of the material by ^{1}H-, ^{13}C- and ^{19}F-NMR, FT-IR and by ESI-HRMS revealed the presence of the cation [(Triphos)Ru(η^2-OAc)]$^+$ as well as the NTf$_2^-$ anion. Crystallisation from dichloromethane layered with pentane gave yellow single crystals of complex **4a** where the open coordination site was saturated with H_2O from adventitious traces of water (X-Ray crystal structure of **4a** is shown in Scheme 2 in the main manuscript and CIF-data is available as electronic supporting information). Thus, the acetate complex in solution can be formulated as [(Triphos)Ru(η^2-OAc)(S)][NTf$_2$] (**4**) with S being a free coordination site or weakly bound solvent molecule.

^{1}H-NMR (600 MHz, d_8-THF, rt): δ = 7.49 - 7.05 (m, 30H, C$_{Ar}$-H), 2.51 (br, 6H, P-CH_2), 2.07 (s, 3H, O$_2$CCH_3), 1.74 (br qua, $J_{\text{H-P}}$ = 2.5 Hz, 3H, CH_3) ppm.

^{13}C{^{1}H}-NMR (151 MHz, d_8-THF, rt): δ = 189.0 (s, O$_2$$CCH_3$), 135.7 (s, C_{Ar}), 132.2 (s, C_{Ar}-H), 129.2 (s, C_{Ar}-H), 127.7 (s, C_{Ar}-H), 120.3 (qua, $J_{\text{C-F}}$ = 321 Hz, CF$_3$), 39.0 [s, (Ph$_2$PCH$_2$)$_3$$C$-CH$_3$], 35.8 (qua, $J_{C\text{-}P}$ = 10.8 Hz, CH$_3$), 33.6 (br, P-CH$_2$), 24.0 (m, O$_2$CCH$_3$) ppm.

^{31}P{^{1}H}-NMR (243 MHz, d_8-THF, rt): δ = 44.0 (s) ppm.

^{19}F{^{1}H}-NMR (566 MHz, d_8-THF, rt): δ = -79.81 (s) ppm.

^{1}H-NMR (400 MHz, d_2-DCM, rt): δ = 7.29 - 7.05 (m, 30H, C$_{Ar}$-H), 2.38 (br, 6H, P-CH_2), 2.11 (s, 3H, O$_2$CCH_3), 1.69 (br qua, $J_{\text{H-P}}$ = 2.2 Hz, 3H, CH_3) ppm.

^{31}P{^{1}H}-NMR (243 MHz, d_2-DCM, rt): δ = 42.5 (s) ppm.

ESI-HRMS (THF) positive ion: calculated for [(Triphos)Ru(η^2-OAc)]$^+$: m/z = 785.14411; determined: m/z = 785.14337.

ATR-IR: $\tilde{\upsilon}$ = 3068 (w), 2966-2870 (w), 1521 (w), 1490 (w), 1464 (m), 1439 (m), 1352 (m), 1230 (w), 1184 (s), 1136 (m), 1094 (m), 1057 (s), 1002 (w), 949 (w), 837 (m), 791 (w), 739 (w), 693 (s), 654 (w), 614 (m), 600 (m), 571 (m), 548 (w), 517 (s), 489 (s), 415 (m) cm^{-1}.

The absorption bands between 1400 and 1600 cm^{-1} (Figure 3) are very similar to the bands in the starting complex [Ru(η^2-OAc)Cl(Triphos)] **9** (Figure 4) indicating a similar chelating binding mode of the acetate ligand.

From NMR-spectroscopy and HRMS it is not possible to discriminate between monomeric [(Triphos)Ru(η^2-OAc)(S)]NTf_2 **4** and multinuclearic species [(Triphos)Ru(η^2-OAc)]$_x$(NTf_2)$_x$. To make the discrimination possible, an experiment was performed as follows:

[Ru(η^2-OAc)Cl(Triphos)] **9** (10.3 mg, 12.5 µmol, 1 eq.) and [Ru(η^2-OAc)Cl(Triphos-Anisyl)] **10** (12.5 mg, 12.5 µmol, 1 eq.) were weighed into a Schlenk-tube and $AgNTf_2$ (11.6 mg, 30 µmol, 2.4 eq.) was added. Toluene (1.5 mL) was added and the solution was stirred at 60 °C for 5 hours. The toluene was removed in vacuo and the residue dissolved in d_2-DCM (0.5 mL). The orange solution with a white precipitate was passed through a syringe-filter and analysed by NMR-spectroscopy.

The ^{31}P{^{1}H}-NMR spectrum at room temperature showed two singlets at 43.5 and 45.3 ppm in a ratio of nearly 1:1 (Figure 5), indicating the formation of two complexes. The signal at 43.5 ppm was assigned to [(Triphos)Ru(η^2-OAc)(S)]NTf_2 **4** whereas the signal at 45.3 ppm was assigned to [(Triphos-Anisyl)Ru(η^2-OAc)(S)]NTf_2 **11**. The absence of further signals due to mixed complexes like [Ru_2(Triphos)(Triphos-Anisyl)(η^2-OAc)$_2$](NTf_2)$_2$ corroborates the monomeric structure of **4**. The ^{1}H-NMR spectrum (Figure 6) shows the corresponding proton signals of [(Triphos)Ru(η^2-OAc)(S)]NTf_2 **4** and [(Triphos-Anisyl)Ru(η^2-OAc)(S)]NTf_2 **11**.

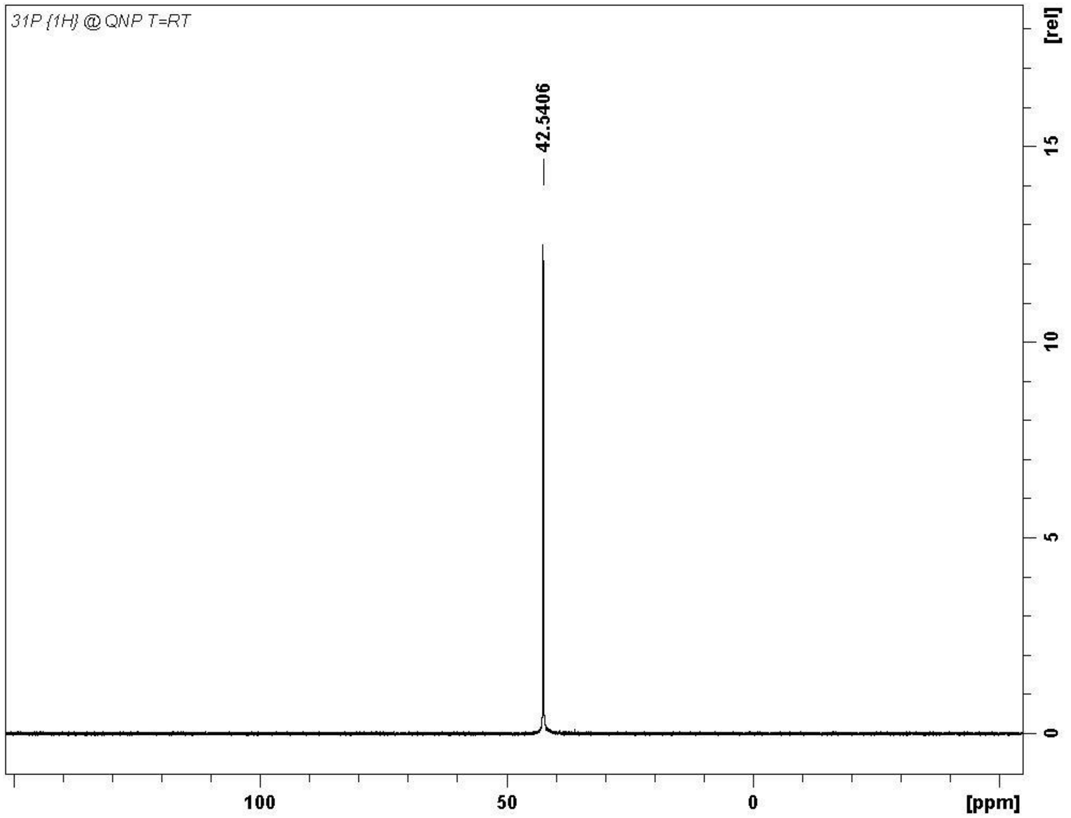

***Figure 1*:** ^{31}P{^{1}H}-NMR-spectrum (d_2-DCM, 243 MHz) of [(Triphos)Ru(η^2-OAc)(S)]NTf_2 **4**.

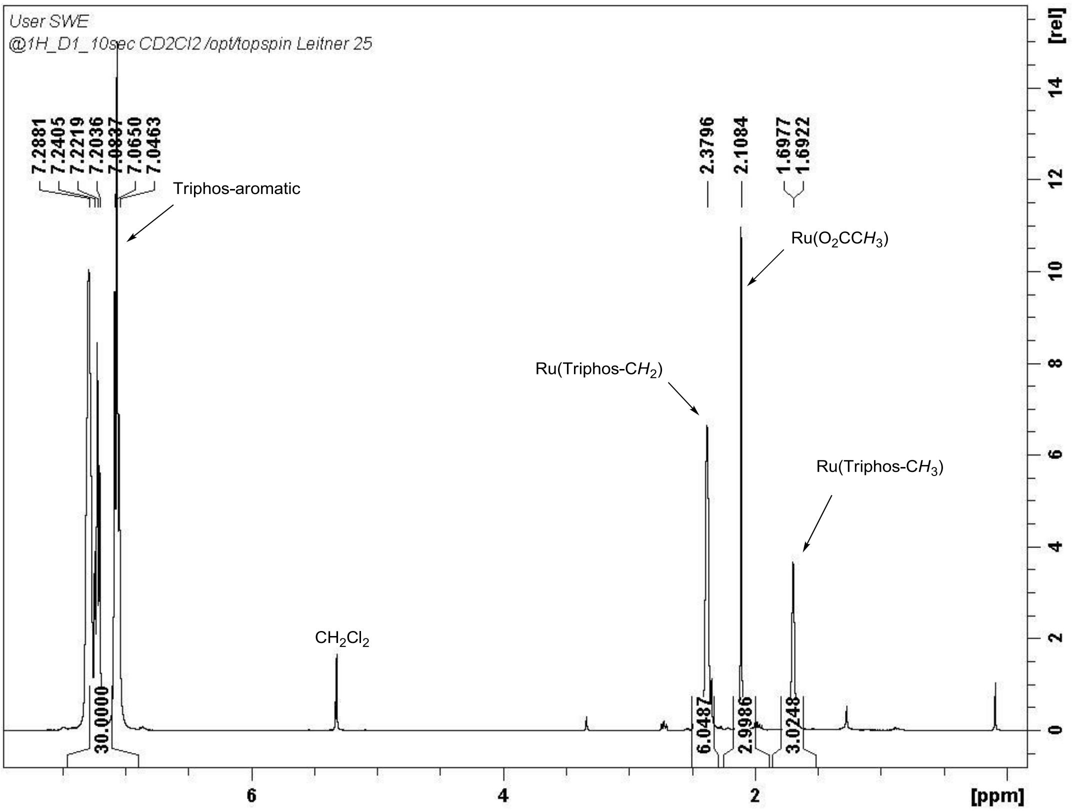

Figure 2: ^{1}H-NMR-spectrum (d_2-DCM, 400 MHz) of [(Triphos)Ru(η^2-OAc)(S)]NTf_2 **4**.

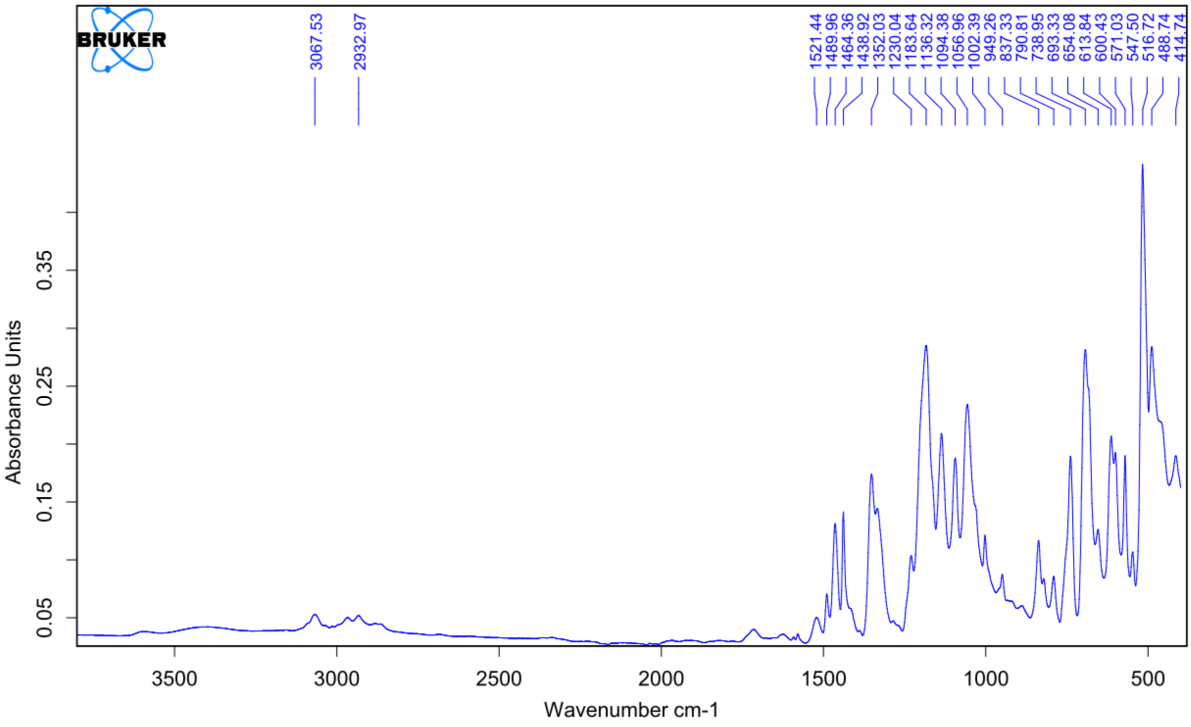

Figure 3: ATR-IR spectrum of [(Triphos)Ru(η^2-OAc)(S)]NTf_2 **4.**

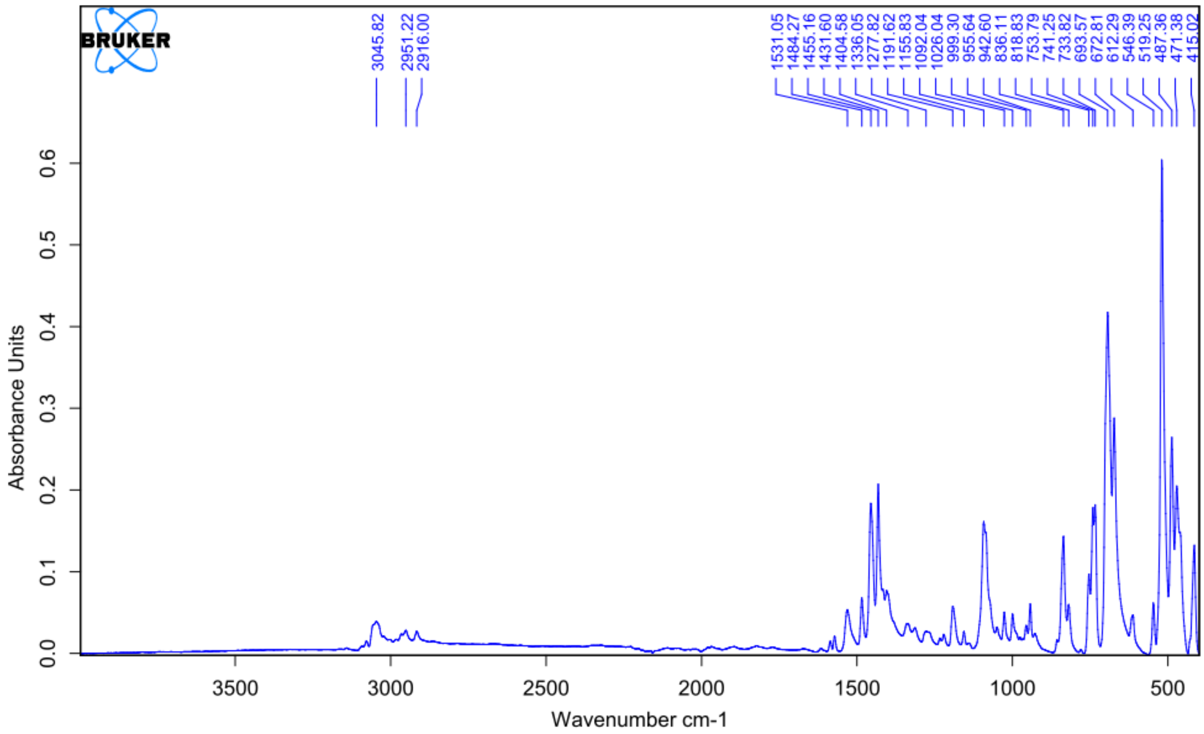

Figure 4: ATR-IR spectrum of the starting material [Ru(η^2-OAc)Cl(Triphos)] **9**.

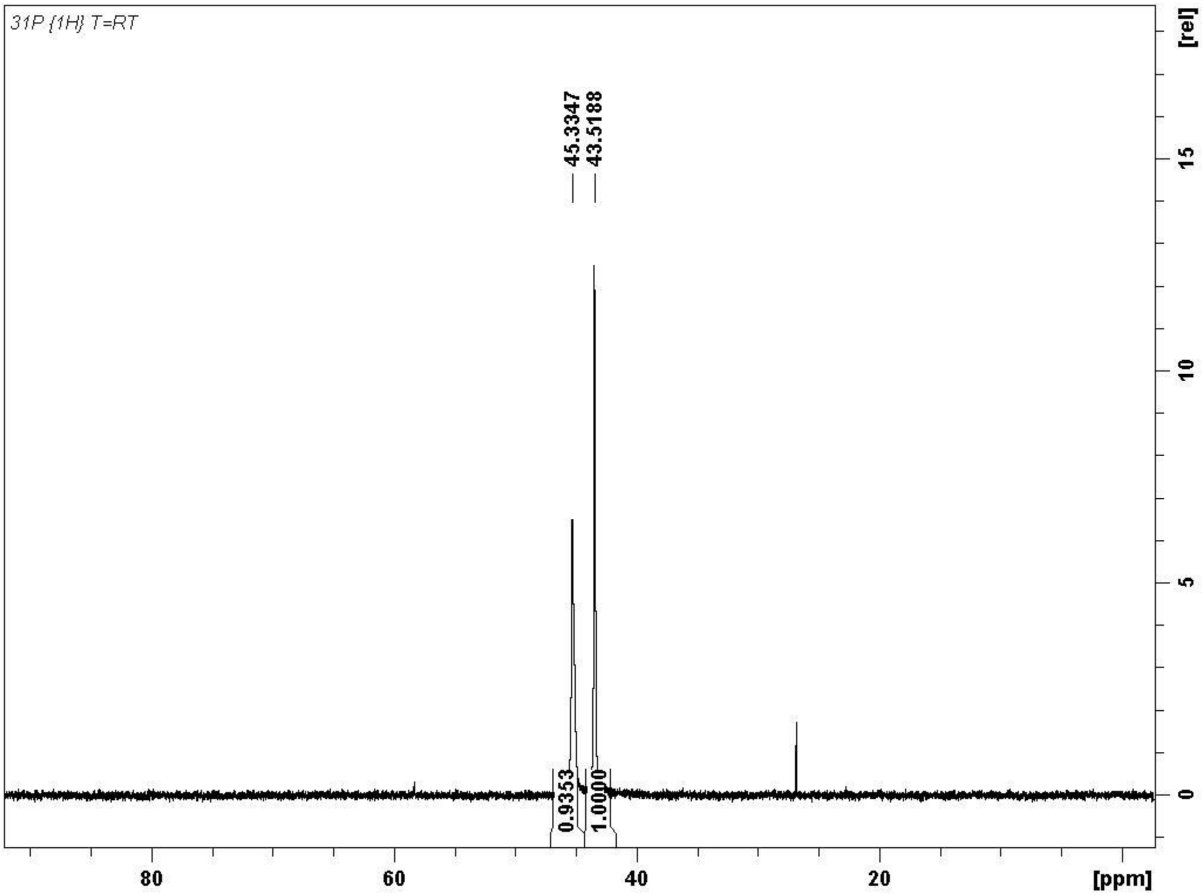

Figure 5: $^{31}P\{^1H\}$-NMR (d_2-DCM) of the reaction of [Ru(η^2-OAc)Cl(Triphos)] **9** and [Ru(η^2-OAc)Cl(Triphos-Anisyl)] **10** (1 eq.) with $AgNTf_2$ (2.4 eq.) in one pot.

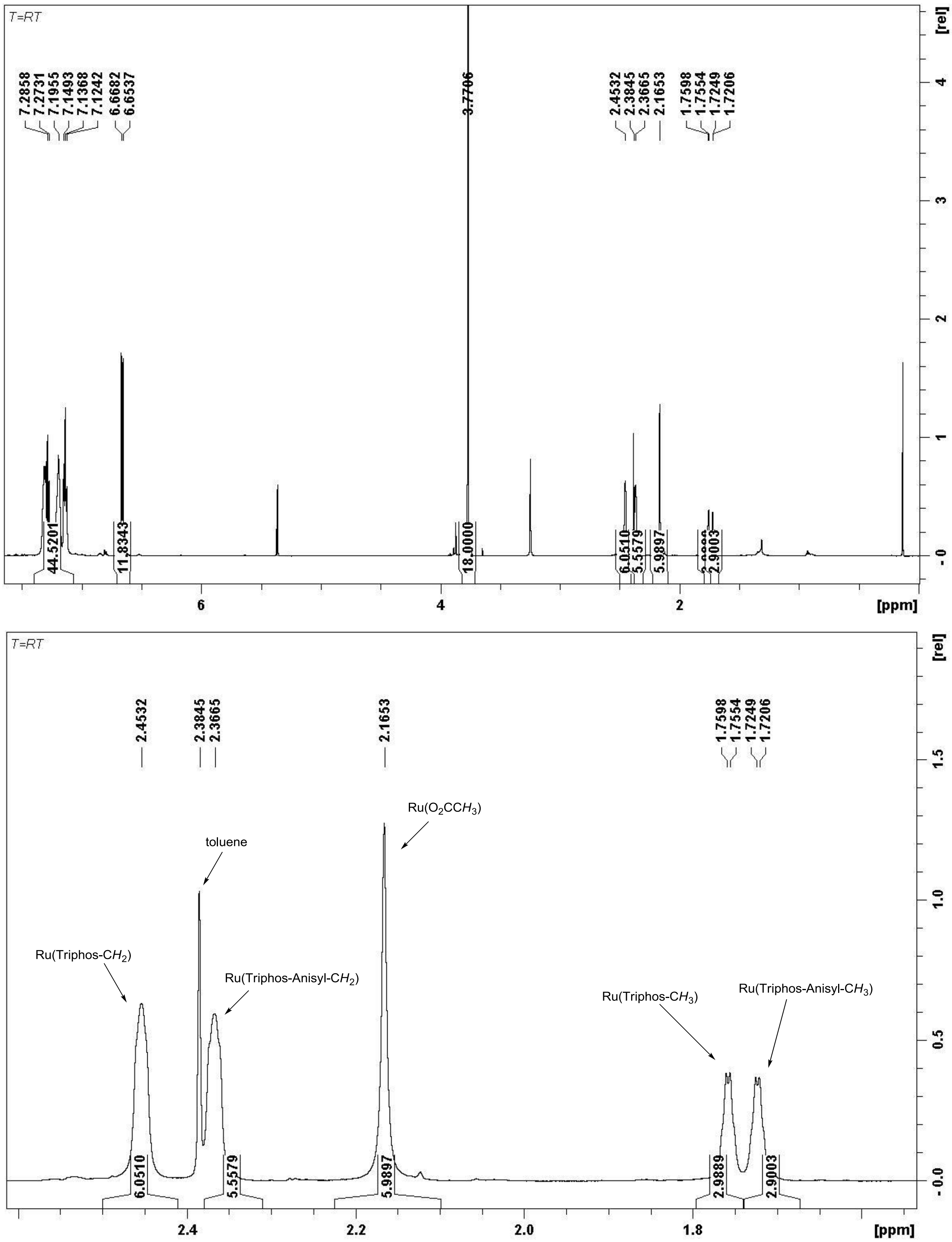

Figure 6: ^{1}H-NMR-spectra (d_2-DCM) of the reaction of [Ru(η^2-OAc)Cl(Triphos)] **9** and [Ru(η^2-OAc)Cl(Triphos-Anisyl)] **10** (1 eq.) with $AgNTf_2$ (2.4 eq.). Top: full spectrum. Bottom: zoom of the region 1.5-2.6 ppm.

3 Synthesis of 1,1,1-tris{bis(4-methoxyphenyl)phosphinomethyl}ethan (Triphos-Anisyl) 15

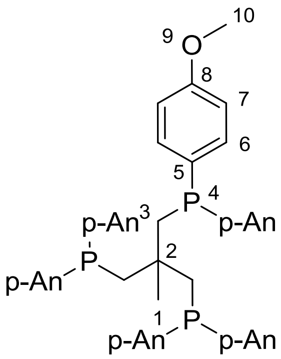

Potassium-tert-butoxide (202 mg, 1.80 mmol, 3.6 eq.) and bis(4-methoxyphenyl)phosphin (369 mg, 1.50 mmol, 1.5 eq.) were dissolved in DMSO (7 mL) and stirred for 0.5 h resulting in a red coloured solution. To this solution, a solution of 1,1,1-tris(chloromethyl)ethan (85 mg, 0.5 mmol, 1 eq.) in DMSO (5 mL) was added slowly over 5 min. The reaction solution was stirred at 60 °C over night during which a decolourisation occurred. Water (20 mL) was added carefully and the resulting emulsion was extracted with diethylether (5×10 mL). The solvent was removed from the extract in vacuo and the product **15** was obtained as a colourless solid (380 mg, 95 %). NMR-spectra of 15 are shown in Figures 7 and 8.

^{1}H-NMR (400 MHz, $CDCl_3$, rt): δ = 7,22-7,15 (m, 12 H, C^6-*H*), 6,74-6,68 (m, 12 H, C^7-*H*), 3,69 (s, 18 H, C^{10}-H_3), 2,26 (bs, 6 H, C^3-H_2), 0,85 (s, 3 H, C^1-H_3) ppm.
^{13}C-NMR (101 MHz, $CDCl_3$, rt): δ = 158,7 (s, C^8), 133,2 (m, C^6), 130,0 (m, C^5), 112,8 (m, C^7), 54,0 (s, C^{10}), 42,5 (m, C^1), 37,7 (m, C^2), 28,4 (m, C^3) ppm.
^{31}P-NMR (162 MHz, $CDCl_3$, rt): δ = -29,9 ppm.
ESI-MS (MeOH) positive ion: m/z = 805.4 (M+1).

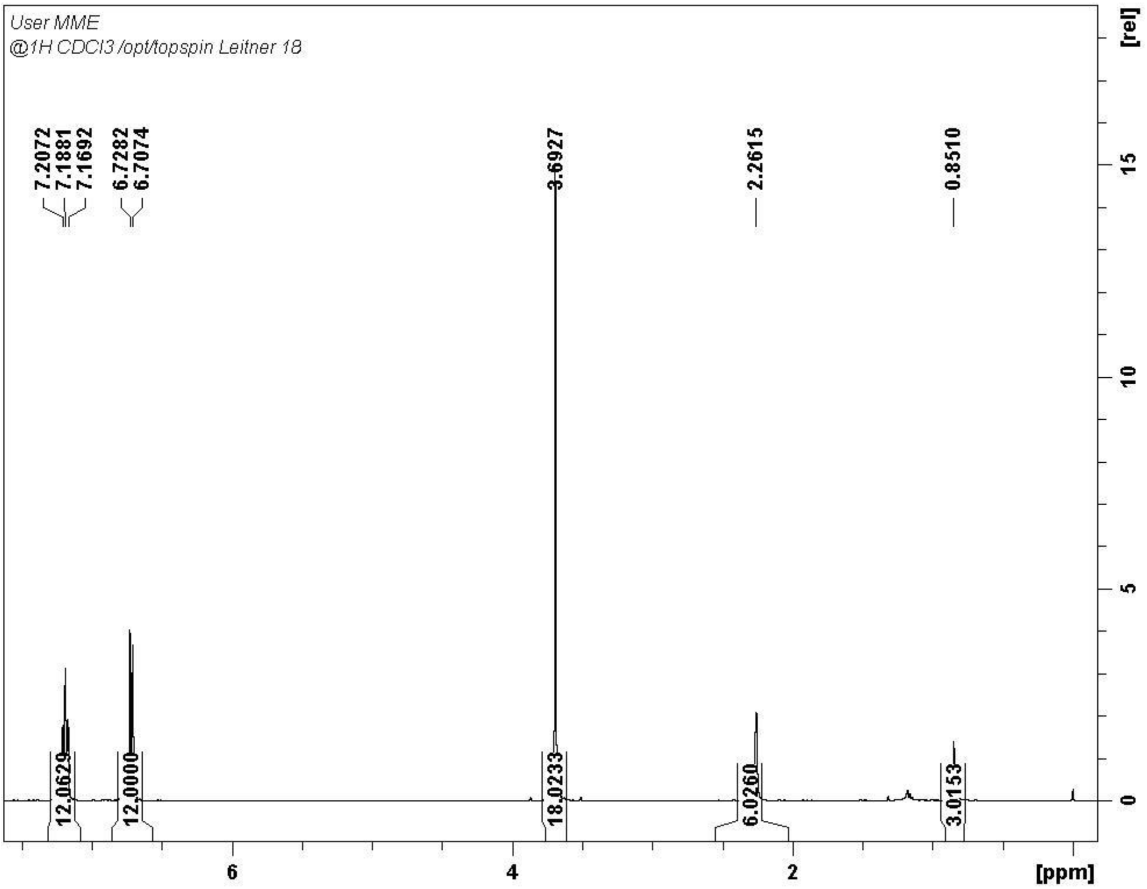

Figure 7**:** ^{1}H-NMR-spectrum ($CDCl_3$, 400 MHz) of Triphos-Anisyl **15**.

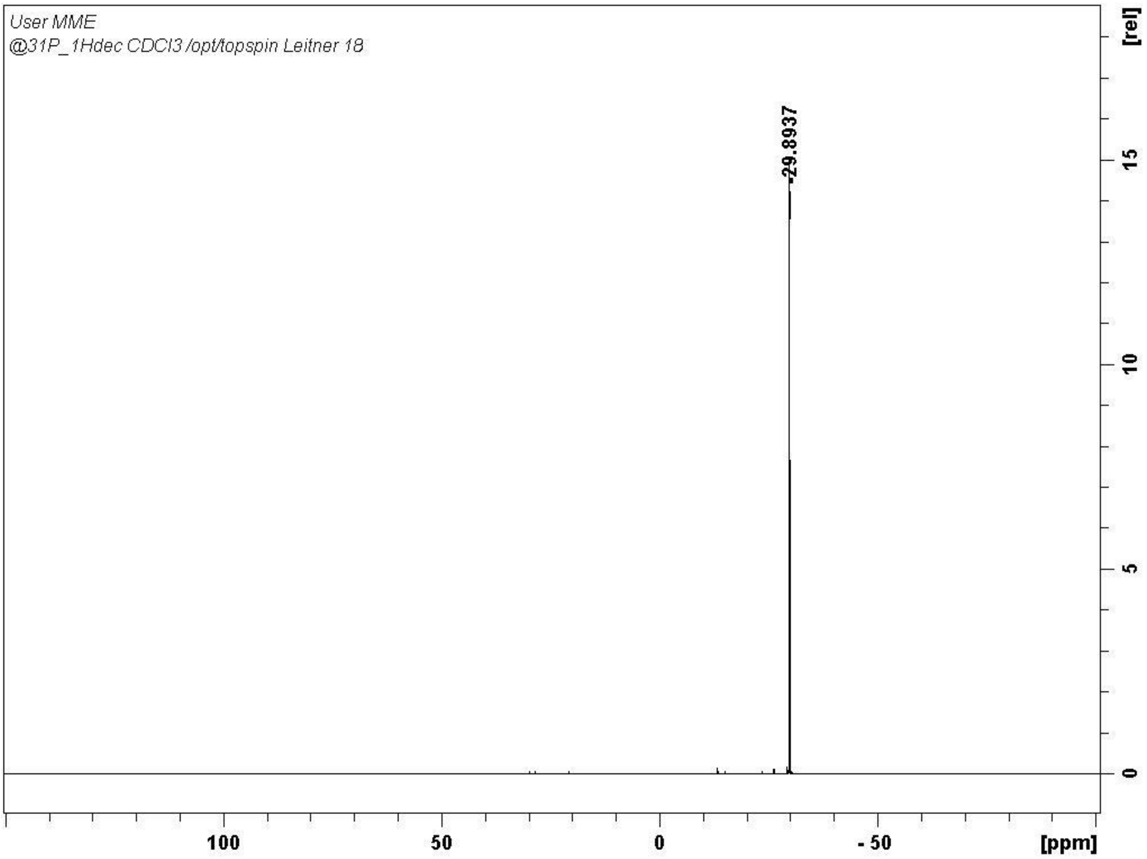

Figure 8**:** ^{31}P{^{1}H}-NMR-spectrum ($CDCl_3$, 400 MHz) of Triphos-Anisyl **15**.

4 Synthesis of [Ru(η^2-OAc)Cl(Triphos-Anisyl)] 10

[Ru(η^2-OAc)Cl(Triphos-Anisyl)] **10** was synthesised like the analogous complex [Ru(η^2-OAc)Cl(Triphos)] **9**.[2]

$Ru(PPh_3)_3$(η^2-OAc)Cl (98.2 mg, 0.1 mmol) and Triphos-Anisyl **15** (80.5 mg, 0.1 mmol, 1 eq.) were dissolved in toluene (9 mL) and the solution stirred and heated at 110 °C for 3 h. During this time, the solution becomes yellow. The solution was cooled to room temperature and after addition of pentane (5 mL) a yellow solid precipitated. After decantation of the supernatant the yellow solid was washed with pentane (3×10 mL) and dried in vacuum (76 mg, yield = 76 %).

^{1}H-NMR (600 MHz, d_8-THF, rt): δ = 7.53 – 6.58 (m, 24H, C_{Ar}-*H*), 3.73 (s, 18H, Ar-OCH_3), 2.17 (br, 6H, P-CH_2), 1.94 (s, 3H, O_2CCH_3), 1.46 (br qua, $J_{H\text{-}P}$ = 2.0 Hz, 3H, CH_3) ppm.
^{13}C{^{1}H}-NMR (151 MHz, d_8-THF, rt): δ = 186.2 (s, O_2CCH_3), 160.5 (s, C_{Ar}-OCH_3), 134.2 (s, C_{Ar}), 113.3 (s, C_{Ar}-H), 55.5 (s, O-CH_3), 38.3 [s, $(An_2PCH_2)_3C$-CH_3], 37.9 [qua, $J_{C\text{-}P}$ = 10.2 Hz, $(An_2PCH_2)_3C$-CH_3], 33.5 (br, P-CH_2), 25.8 (m, O_2CCH_3) ppm.
^{31}P{^{1}H}-NMR (243 MHz, d_2-DCM, rt): δ = 38.8 (br, 2P), 34.5 (br, 1P) ppm.

ESI-HRMS (THF) positive ion: [Ru(η^2-OAc)(Triphos-Anisyl)]$^+$ calculated: m/z = 965.20750; determined: m/z = 965.20648.

ATR-IR: $\tilde{\upsilon}$ = 3100-2900 (w), 2834 (w), 1593 (m), 1570 (m), 1531 (w), 1500 (m), 1454 (m), 1404 (m), 1288 (m), 1245 (s), 1180 (s), 1089 (s), 1028 (m), 942 (w), 844 (w), 822 (m), 797 (s), 745 (w), 722 (w), 674 (m), 622 (m), 540 (s), 525 (m), 497 (m), 465 (m), 432 (w), 418 (m) cm^{-1}.

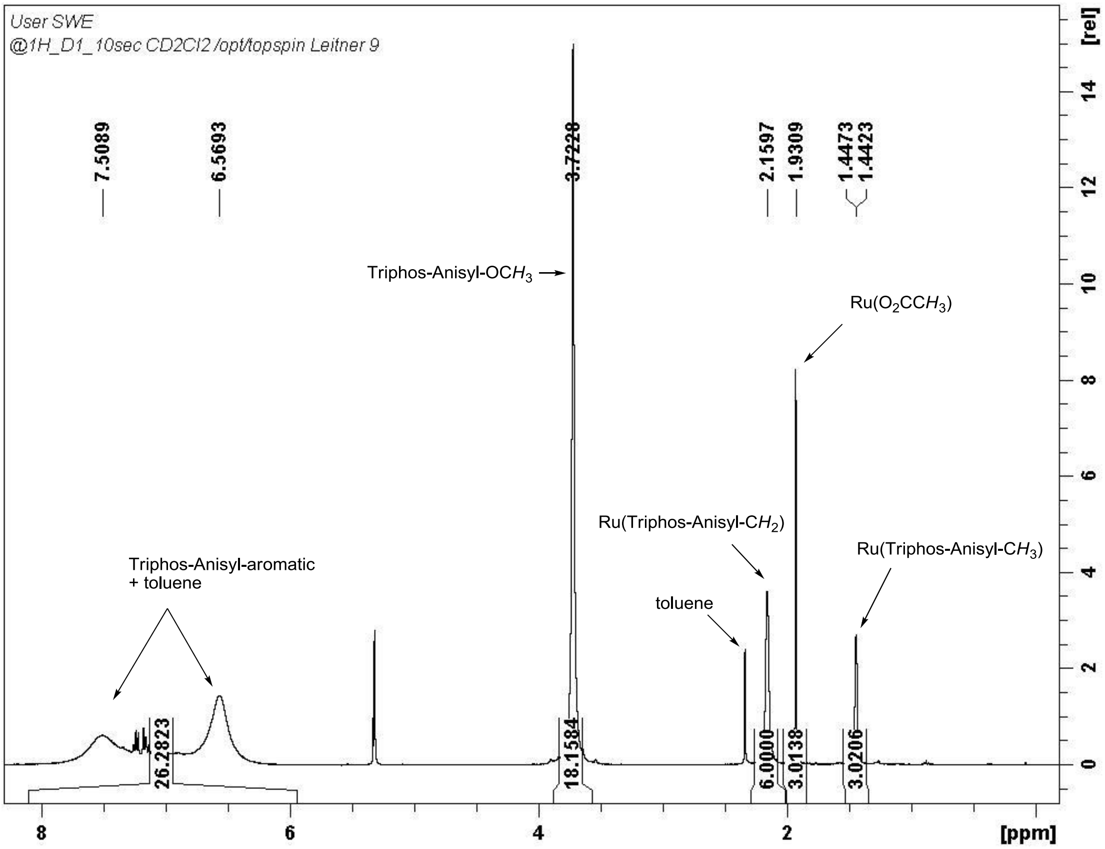

Figure 9: ^{1}H-NMR-spectrum (d_2-DCM, 400 MHz) of [Ru(η^2-OAc)Cl(Triphos-Anisyl)] **10**.

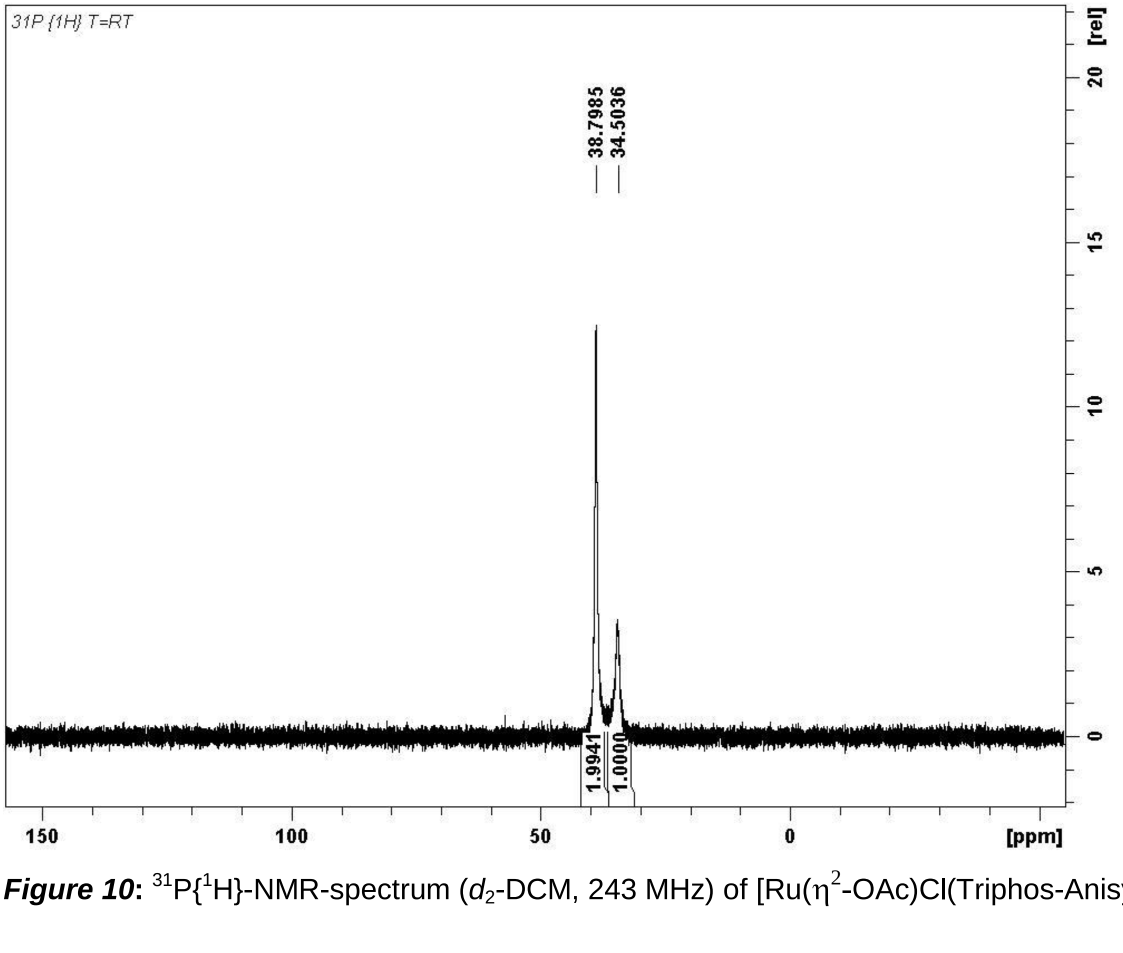

Figure 10: ^{31}P{^{1}H}-NMR-spectrum (d_2-DCM, 243 MHz) of [Ru(η^2-OAc)Cl(Triphos-Anisyl)] **10**.

5 General procedure for CO_2 hydrogenation experiments

All high pressure batch experiments were conducted in stainless steel autoclaves (inner volume = 13 mL) equipped with a glass inlet and a magnetic stir bar. Prior to use, the autoclave was evacuated and repeatedly purged with argon. Under an argon atmosphere, catalyst **2** (0.025 mmol), together with $HNTf_2$ (1 eq.) was weighed into a Schlenk tube and dissolved in THF (2.08 mL). Alternatively, a THF solution of catalyst **4** (0.025 mmol) was prepared by heating [Ru(η^2-OAc)Cl(Triphos)] **9** (0.025 mmol, 20.5 mg) together with $AgNTf_2$ (1.1 eq., 0.0275 mmol, 10.7 mg) in 1 mL of THF for 3 h at 60 °C. The solution was filtrated over silica and the silica washed with 1 mL of THF. Alternatively, isolated catalyst **4** (0.025 mmol) could be used in THF (2.08 mL) and gave the same results.

In either case the solution was transferred via cannula to a stainless steel autoclave under argon atmosphere. The autoclave was pressurised with carbon dioxide to 20 bar and then hydrogen was added up to a total pressure of 80 bar. The reaction mixture was stirred and heated at 140 °C in an oil bath giving a total pressure of about 120 bar. After 24 h, the autoclave was cooled to 0 °C in an ice bath and then carefully vented. The resulting clear solution was analysed by ^{1}H-NMR (D1 = 10 s) in d_6-DMSO with internal standard mesitylene and the results confirmed by gas chromatography using heptane as internal standard. Figure 11 shows a representative ^{1}H-NMR spectrum and Figure 12 a representative GC chromatogram.

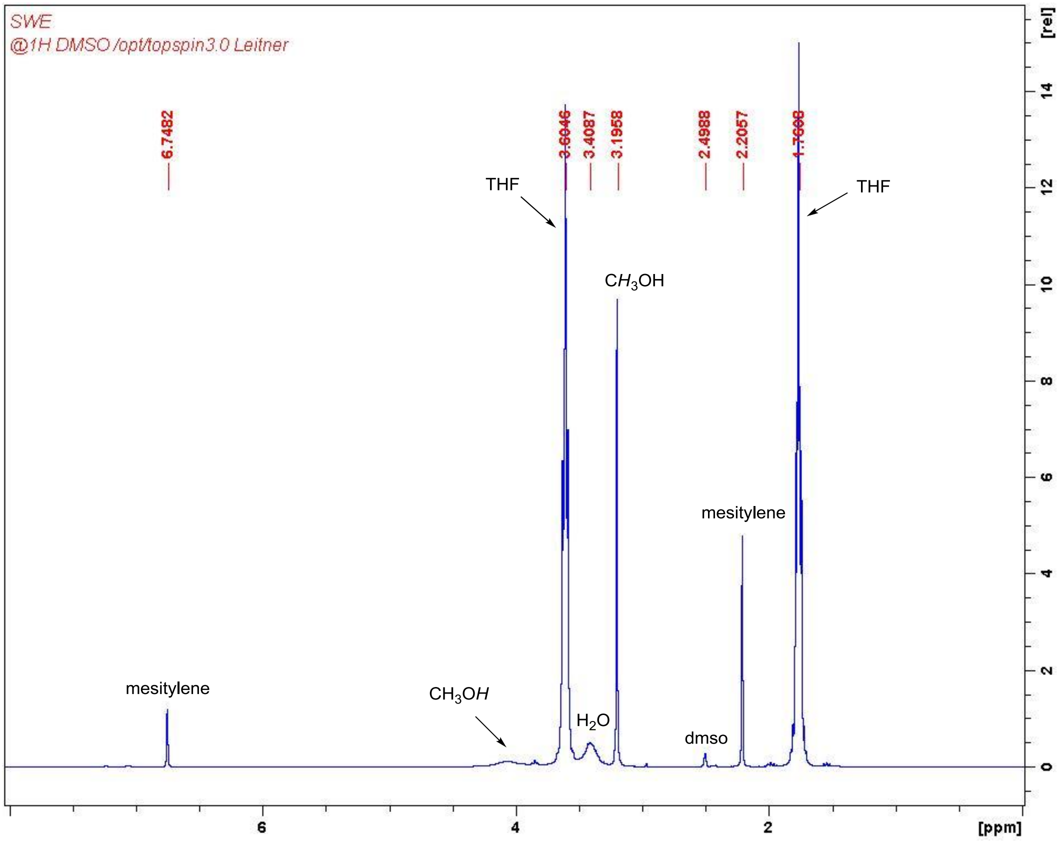

Figure 11: ^{1}H-NMR (300 MHz, d_6-dmso) spectrum of the reaction solution from a CO_2 hydrogenation reaction using above described standard protocol (internal standard mesitylene).

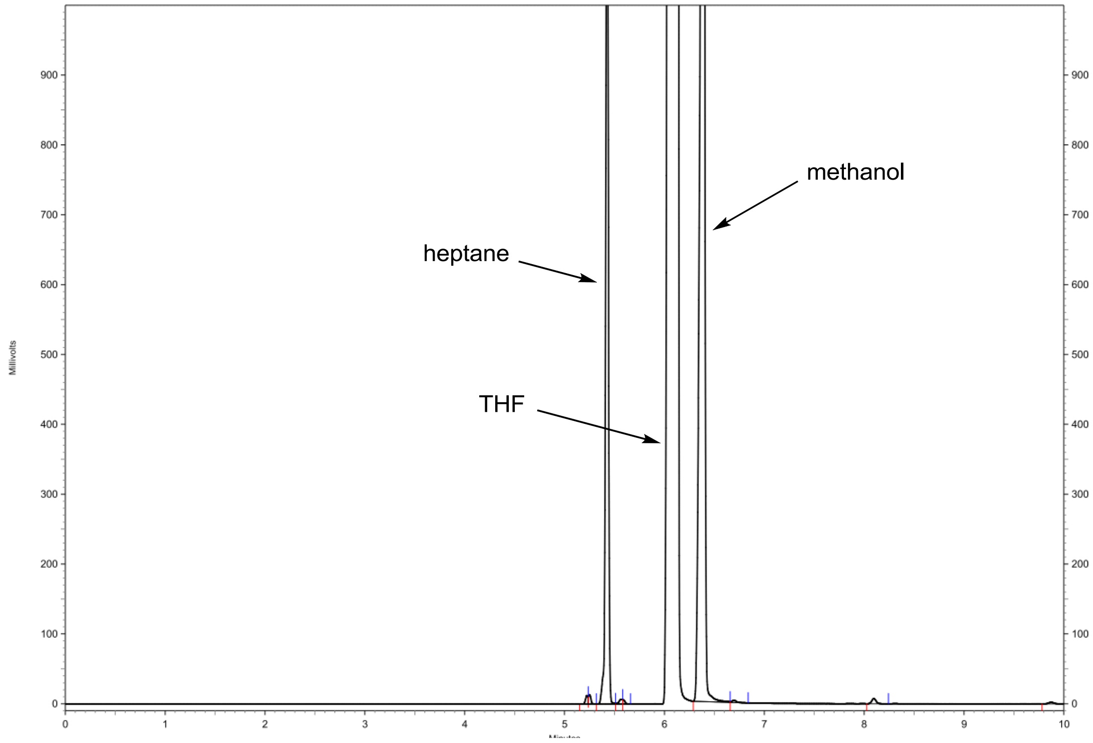

Figure 12: Representative gas chromatogram of the reaction solution from a CO_2 hydrogenation reaction using above described standard protocol (internal standard heptane).

6 Spectroscopic investigations

6.1 NMR-spectroscopic analysis of the reaction mixture using complex 2 together with HNTf$_2$

A CO_2-hydrogenation reaction was performed under routine conditions (25 µmol complex **2**, 1 equivalent HNTf$_2$, 2.08 mL d_8-THF, $p(CO_2)$ = 20 bar at r.t., $p(H_2)$ = 60 bar at r.t., 140 °C) and terminated after 1 hour. The reaction solution was analysed via NMR. Figure 13 shows the $^{31}P\{^1H\}$-NMR spectrum at -40 °C and the assigned complex structures. Figure 14 shows the ^1H-NMR-Spectrum which shows the signal due to coordinated formate in **3a** at 8.7 ppm, as well as the hydride signals of [(Triphos)RuH(CO)$_2$]$^+$ **5** and [Ru$_2$(μ-H)$_2$(Triphos)$_2$] **6**. In Figure 15 the [^{31}P,^{1}H]-HMBC spectrum at -40 °C is depicted which shows the signal due to the coordinated formate in [(Triphos)Ru(η^2-O$_2$CH)(THF)]$^+$ **3a**. Figure 16 depicts the [^{13}C,^{1}H]-HMQC spectrum which also shows the signal due to the coordinated formate.

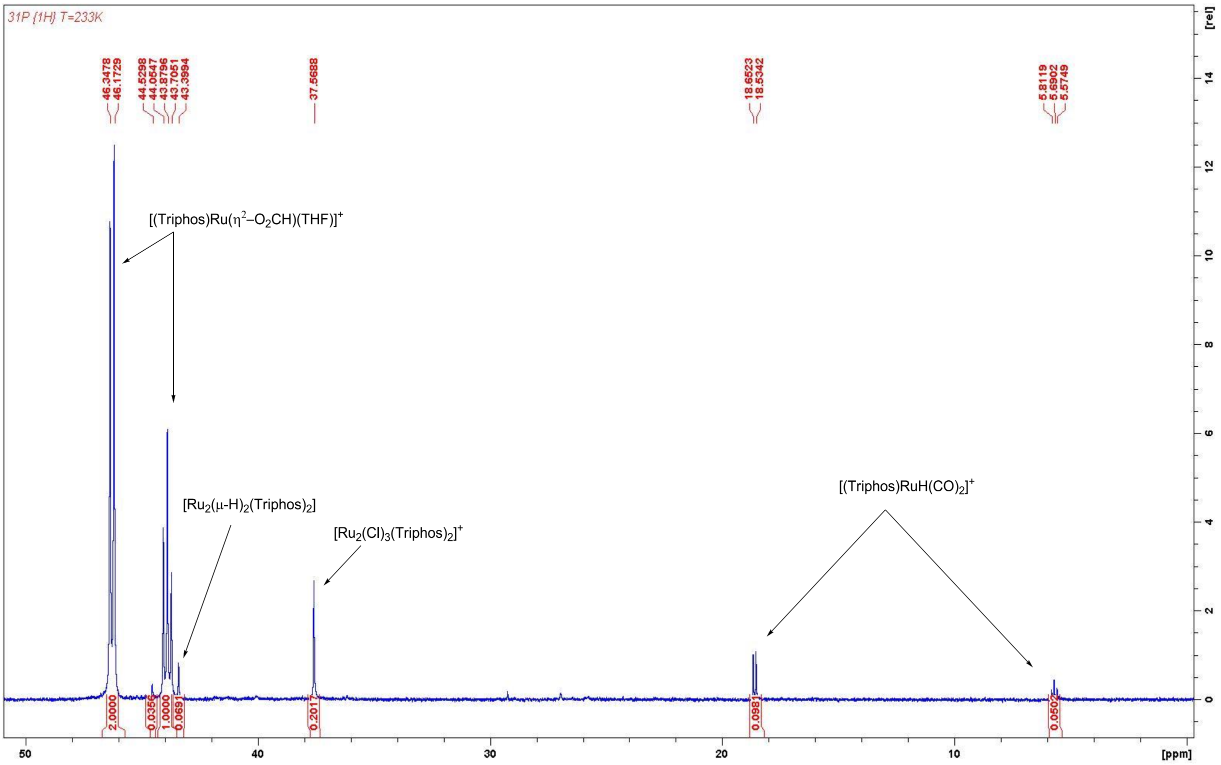

Figure 13: $^{31}P\{^1H\}$-NMR (243 MHz, d_8-THF, -40 °C) spectrum of the reaction solution of a CO_2-hydrogenation reaction (25 µmol complex **2**, 1 equivalent HNTf$_2$, 2.08 mL d_8-THF, $p(CO_2)$ = 20 bar at r.t., $p(H_2)$ = 60 bar at r.t., 140 °C, 1 h reaction time).

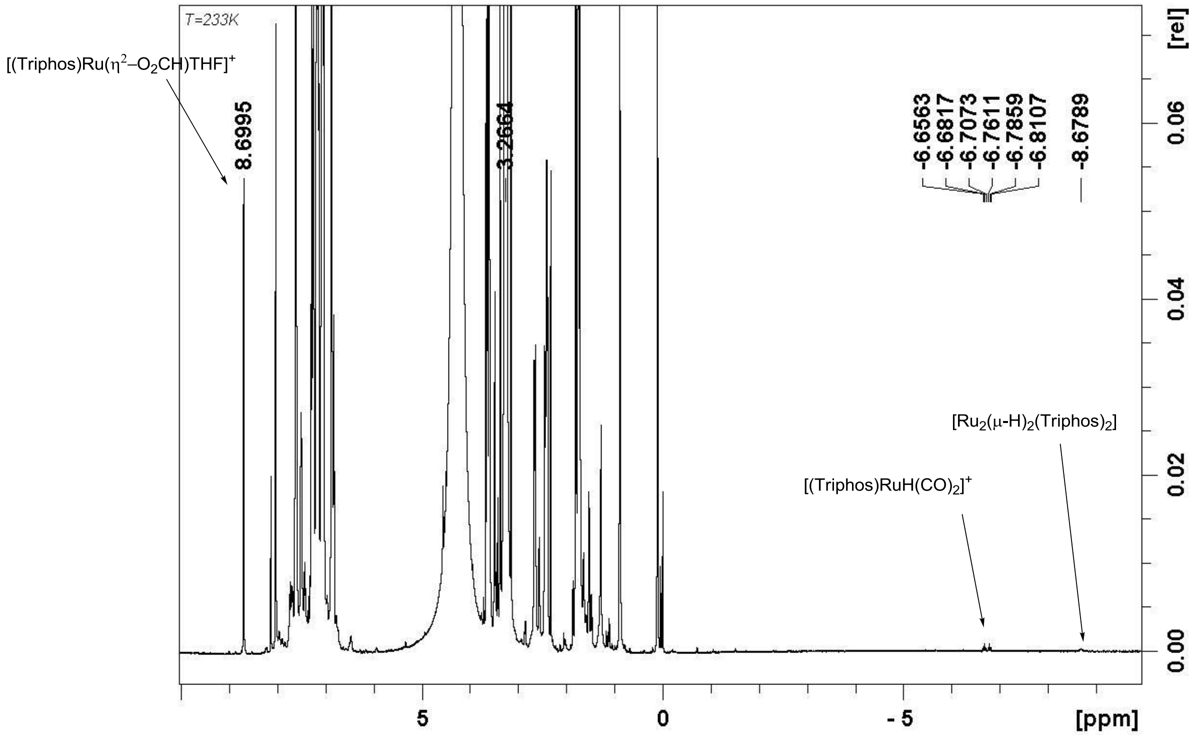

Figure 14: ^{1}H-NMR (600 MHz, d_8-THF, -40 °C) spectrum of the reaction solution of a CO_2-hydrogenation reaction (25 μmol complex **2**, 1 equivalent $HNTf_2$, 2.08 mL d_8-THF, $p(CO_2)$ = 20 bar at r.t., $p(H_2)$ = 60 bar at r.t., 140 °C, 1 h reaction time).

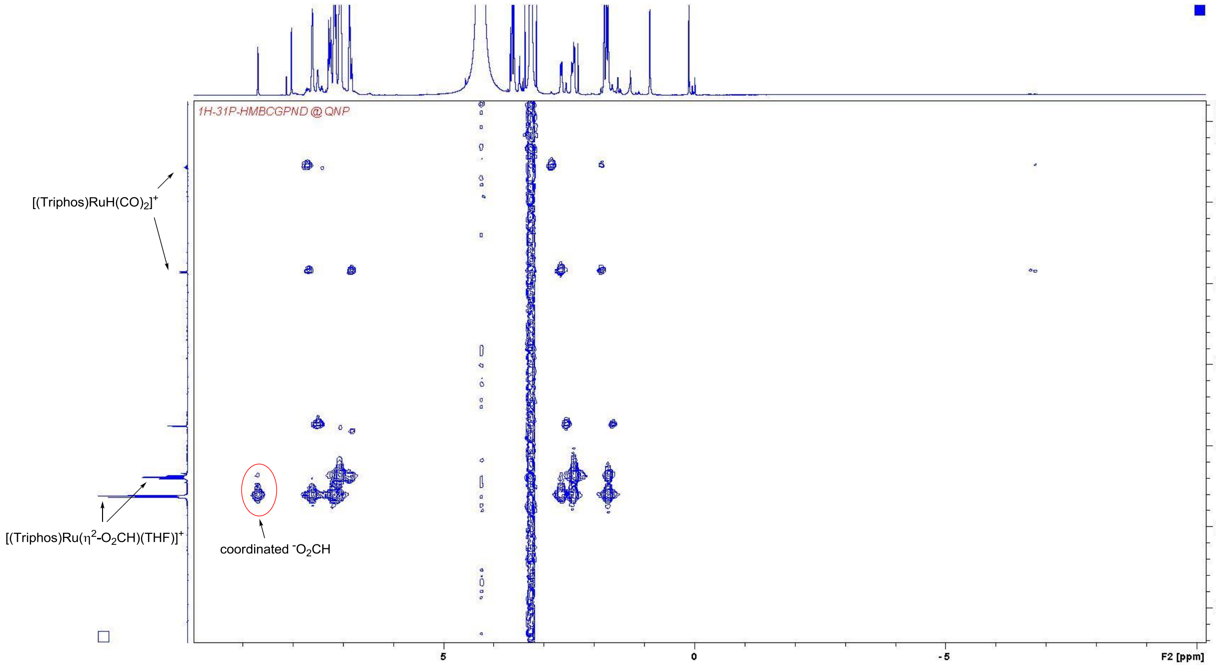

Figure 15: [^{31}P,^{1}H]-HMBC (600 MHz, d_8-THF, -40 °C) spectrum of the reaction solution of a CO_2-hydrogenation reaction (25 μmol complex **2**, 1 equivalent $HNTf_2$, 2.08 mL d_8-THF, $p(CO_2)$ = 20 bar at r.t., $p(H_2)$ = 60 bar at r.t., 140 °C, 1 h reaction time).

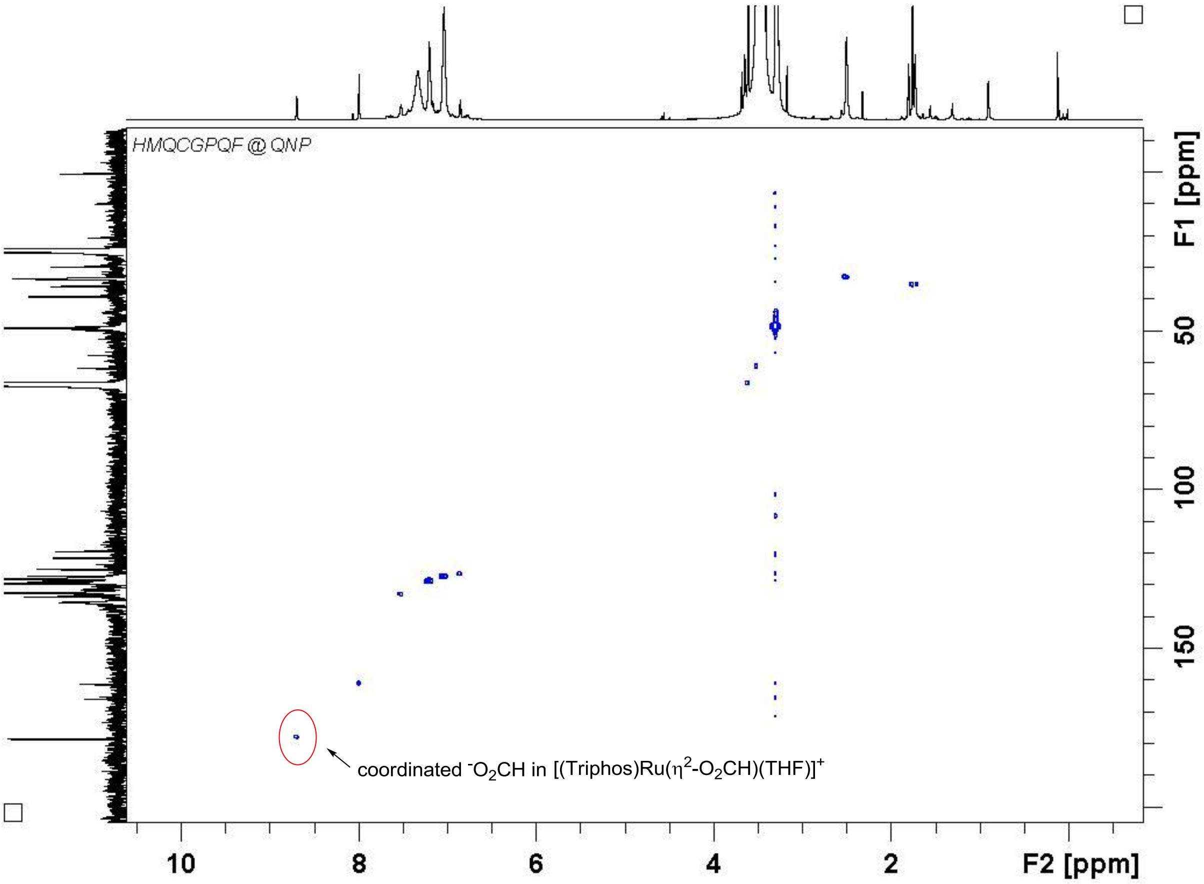

Figure 16: [^{13}C,^{1}H]-HMQC (600 MHz, d_8-THF, -40 °C) spectrum of the reaction solution of a CO_2-hydrogenation reaction (25 µmol complex **2**, 1 equivalent $HNTf_2$, 2.08 mL d_8-THF, $p(CO_2)$ = 20 bar at r.t., $p(H_2)$ = 60 bar at r.t., 140 °C, 1 h reaction time).

6.2 Generation and analysis of the formate complex 3

$HNTf_2$ (7.0 mg, 0.025 mmol) was dissolved in d_8-THF (0.5 mL) and added to complex **2** (19.5 mg, 0.025 mmol, 1 eq.) in d_8-THF (0.5 mL) at room temperature giving a deep red coloured solution. HCO_2H (0.9 µL, 0.025 mmol, 1 eq.) was added via micro-syringe and the solution became orange. The solution was analysed via NMR-spectroscopy (Figures 17-19). The same formate species **3a** as observed in the CO_2 hydrogenation reaction was detected. The transmission IR-spectrum recorded in d_8-THF solution (Figure 20) showed a $\nu_{COassym}$ stretching mode at 1543 cm^{-1}, a typical value for η^2-coordinated formate. After addition of acetonitrile (0.1 mL) the solution was again analysed by NMR-spectroscopy. The $^{31}P\{^1H\}$-NMR showed new signals due to the formation of complexes bearing the MeCN ligand. The assignment of the signals to structures is shown in Figure 21. The signals due to [(Triphos)Ru(MeCN)$_2$H]$^+$ **8**, which increased over time, indicate reversibility of the CO_2 insertion. The corresponding ^{1}H-NMR spectra are shown in Figure 22.

NMR-Data of [(Triphos)Ru(η^2-O_2CH)(THF)][NTf_2] **3a**:

^{1}H-NMR (600 MHz, d_8-THF, -40 °C): δ = 8.75 (br, 1H, O_2CH), 7.60 – 6.90 (m, 30H, C_{Ar}-H), 3.60 (br, THF), 2.69 (d, $J_{H\text{-}P}$ = 15.6 Hz, 2H, P-CH_2), 2.47 (d, $J_{H\text{-}P}$ = 15.6 Hz, 2H, P-CH_2), 2.43 (d, $J_{H\text{-}P}$ = 9.6 Hz, 2H, P-CH_2), 1.76 (br, THF), 1.75 [br, 3H, $(Ph_2PCH_2)_3$C-CH_3] ppm.

^{31}P{^{1}H}-NMR (243 MHz, d_8-THF, -40 °C): δ = 47.1 (d, $J_{P\text{-}P}$ = 42.4 Hz, 2P), 44.2 (t, $J_{P\text{-}P}$ = 42.4 Hz, 1P) ppm.

^{13}C{^{1}H}-NMR (151 MHz, d_8-THF, rt): δ = 178.4 (s, O_2*C*H), 135.4 (s, C_{Ar}), 132.3 (s, C_{Ar}-H), 129.3 (s, C_{Ar}-H), 127.8 (s, C_{Ar}-H), 120.3 (qua, $J_{C\text{-}F}$ = 322 Hz, CF_3), 66.4 (m, THF), 39.3 [s, $(Ph_2PCH_2)_3$*C*-CH_3], 35.7 (m, *C*H_3), 33.8 (br, P-*C*H_2), 24.4 (m, THF) ppm.

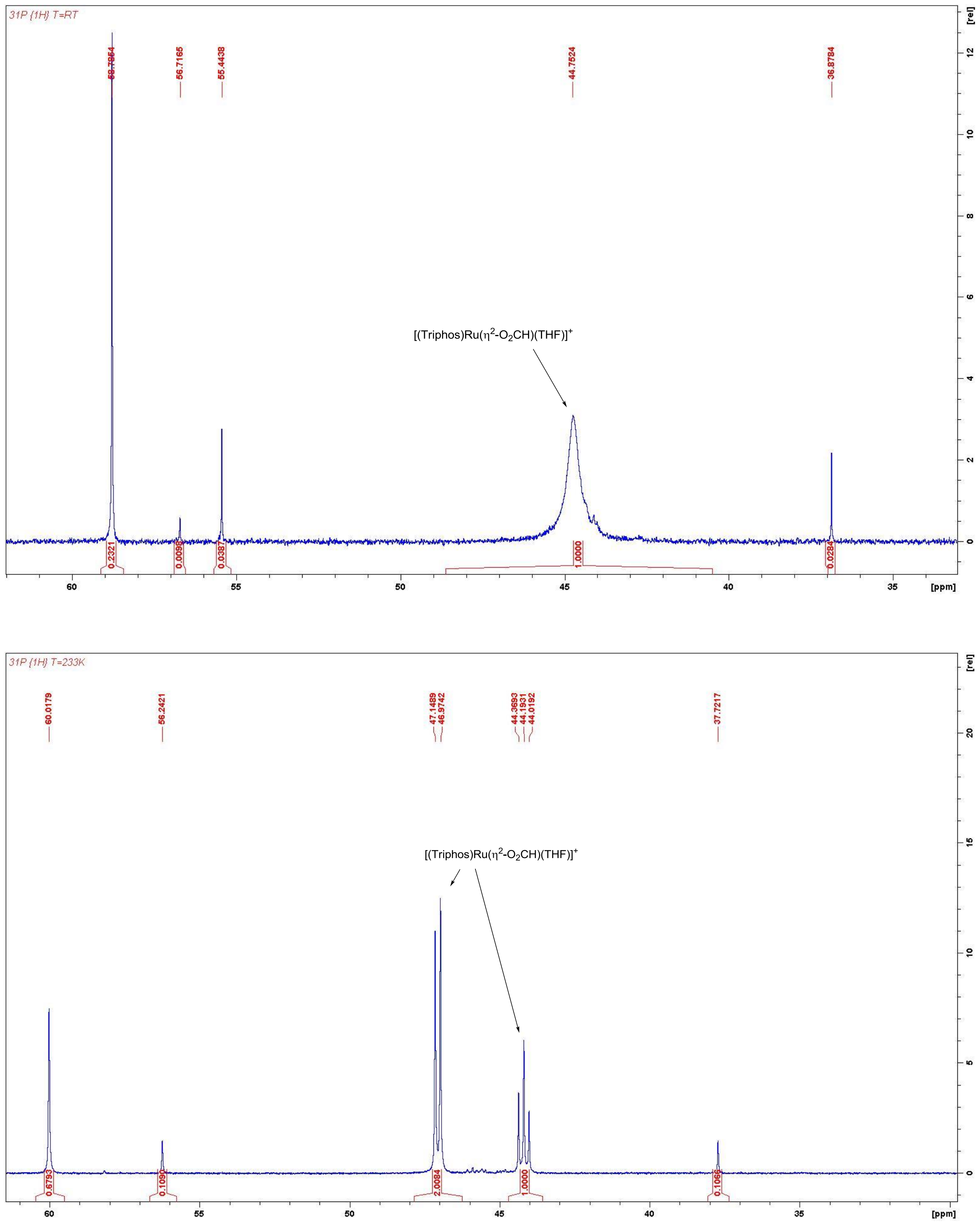

Figure 17: $^{31}P\{^{1}H\}$-NMR (243 MHz, d_8-THF) spectrum after addition of 1 equivalent $HNTf_2$ and 1 equivalent HCO_2H to complex **2** in d_8-THF.Top: at room temperature, bottom at -40 °C.

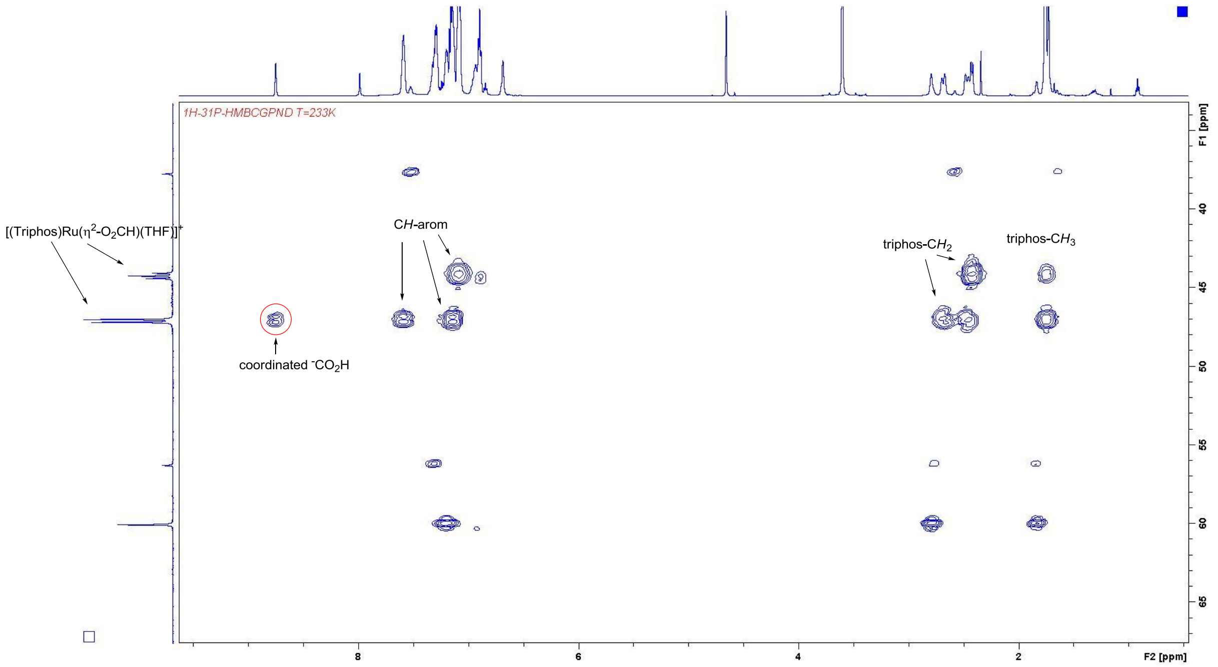

Figure 18: [^{31}P,^{1}H]-HMBC (600 MHz, d_8-THF, -40 °C) spectrum after addition of 1 equivalent $HNTf_2$ and 1 equivalent HCO_2H to complex **2** in d_8-THF.

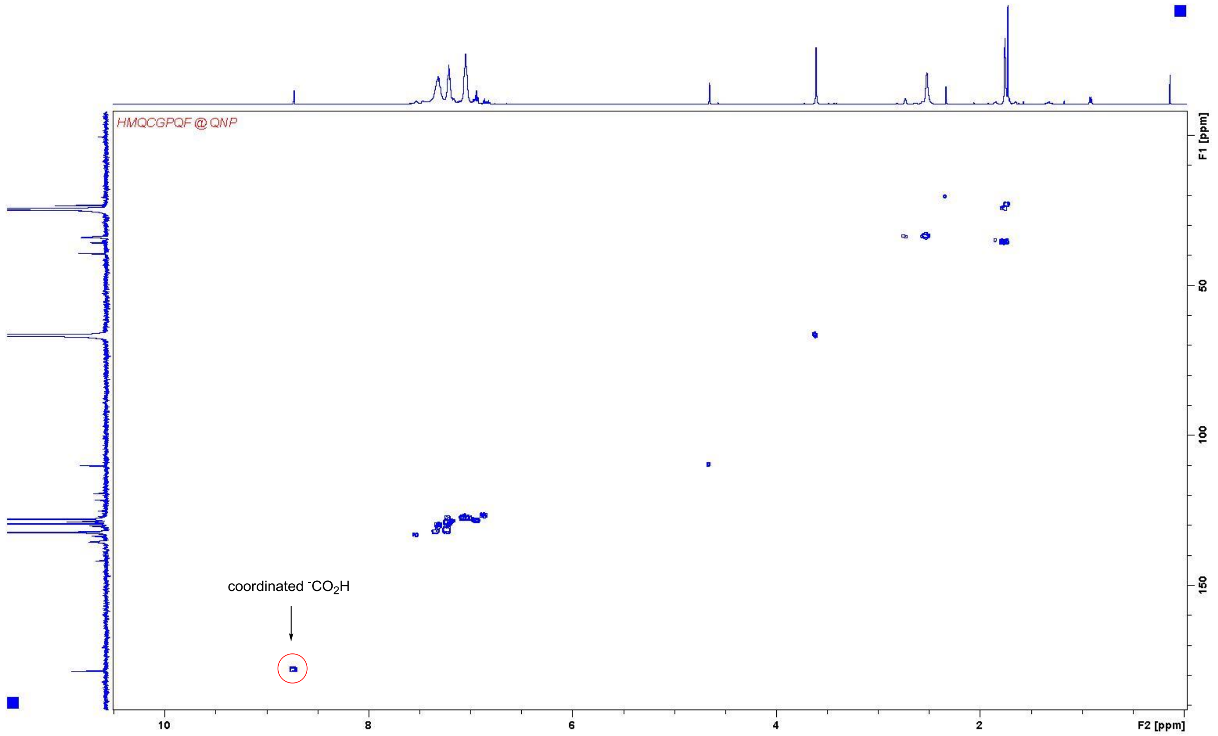

Figure 19: [^{13}C,^{1}H]-HSQC (600 MHz, d_8-THF, -40 °C) spectrum after addition of 1 equivalent $HNTf_2$ and 1 equivalent HCO_2H to complex **2** in d_8-THF.

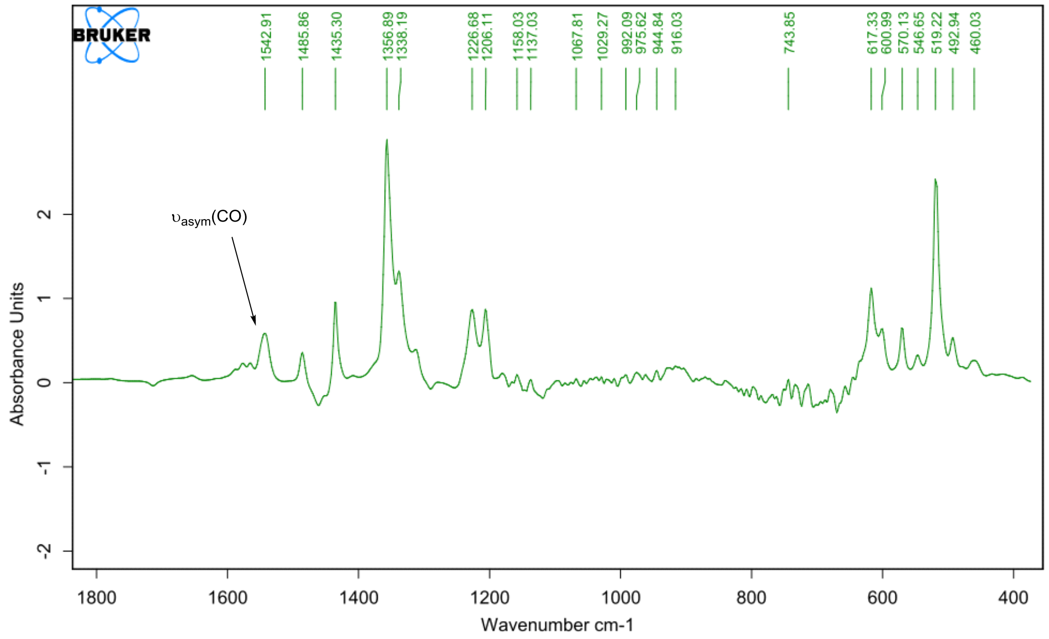

Figure 20: IR spectrum (transmission) after addition of 1 equivalent $HNTf_2$ and 1 equivalent HCO_2H to complex **2** in d_8-THF.

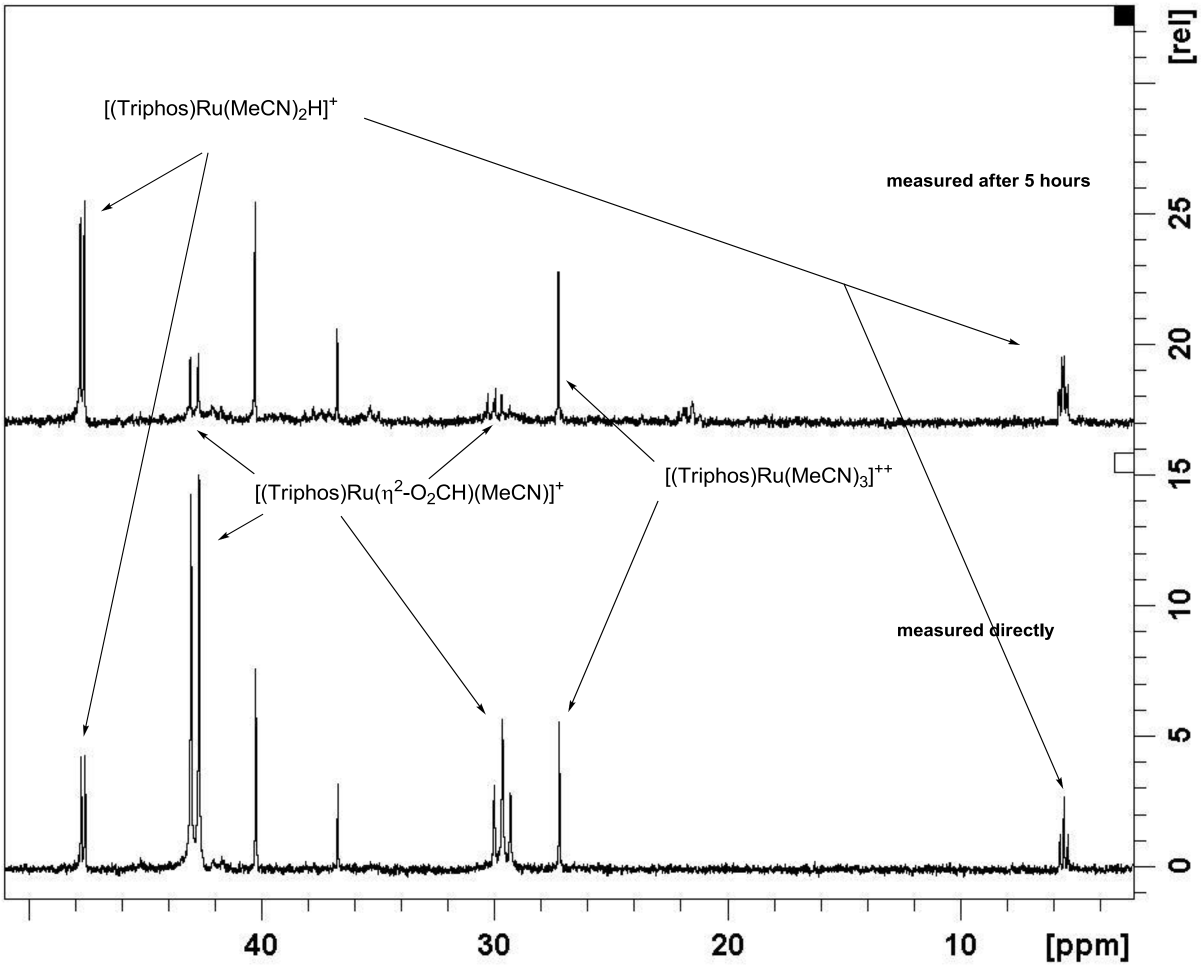

Figure 21: $^{31}P\{^1H\}$-NMR (121 MHz, d_8-THF, room temperature) spectrum after addition of 1 equivalent $HNTf_2$ and 1 equivalent HCO_2H to complex **2** in d_8-THF and 0.1 mL acetonitrile measured directly (bottom) and again after 5 hours at room temperature (top).

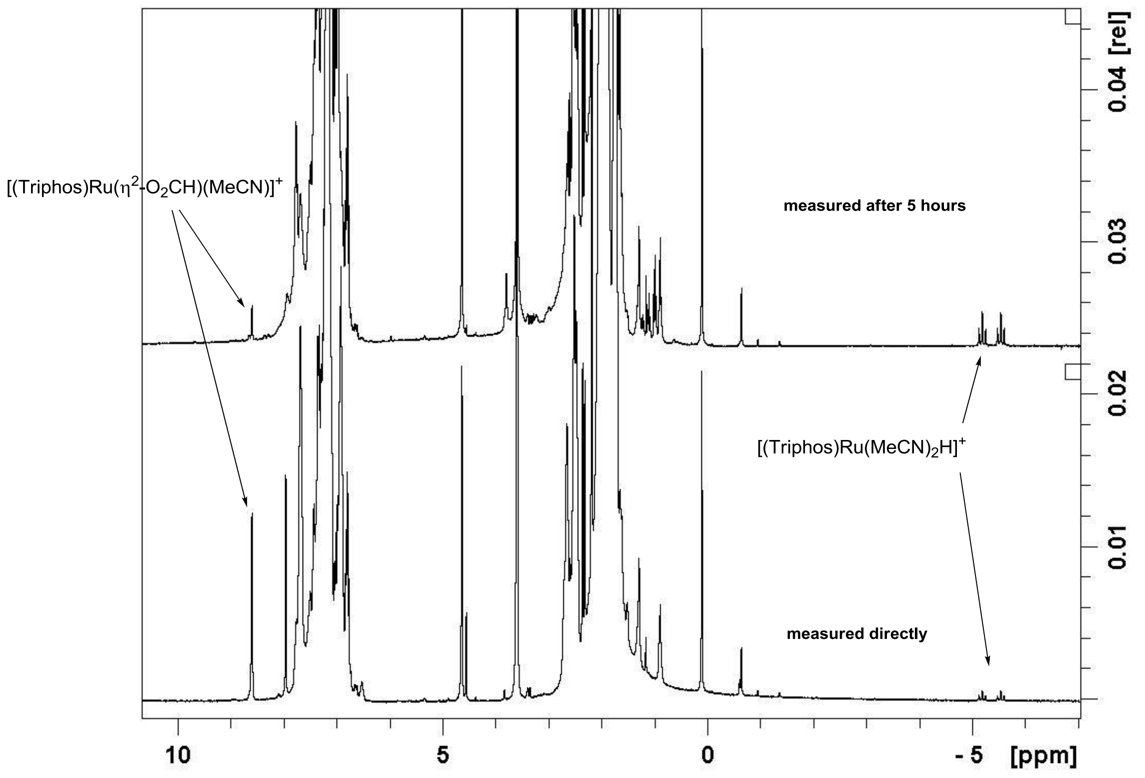

Figure 22: ^{1}H-NMR (300 MHz, d_8-THF, room temperature) spectrum after addition of 1 equivalent $HNTf_2$ and 1 equivalent HCO_2H to complex **2** in d_8-THF and 0.1 mL acetonitrile measured directly (bottom) and again after 5 hours at room temperature (top).

6.3 Conversion of formate species 3a to methanol

$HNTf_2$ (7.0 mg, 0.025 mmol) was dissolved in d_8-THF (0.5 mL) and added to complex **2** (19.5 mg, 0.025 mmol, 1 eq.) in d_8-THF (0.5 mL) at room temperature giving a deep red coloured solution. HCO_2H (0.9 µL, 0.025 mmol, 1 eq.) was added via micro-syringe and the solution became orange. 0.3 mL of this solution were transferred to a high-pressure NMR tube (volume = 0.93 mL). A ^{1}H-NMR spectrum of this solution was measured at ambient pressure and room temperature (Figure 23, top) and showed formation of **3a** in about 86 %. The NMR-tube was pressurised with 60 bar of hydrogen and carefully (only the lower 4 cm of the tube were dipped into the oil-bath) heated at 140 °C in an external oil-bath for 40 minutes. After that, a ^{1}H-NMR-spectrum of the solution was measured at 80 °C in the spectrometer (Figure 23, bottom). Integration of the formate signal and the methanol signal relative to the aromatic protons of the triphos-ligand revealed a ca. 97 % conversion of the formate-ligand to methanol. In the corresponding ^{31}P{^{1}H}-NMR spectra the formation of **6** was observed in about 44 % of the total intensity besides some other, yet unknown species (Figure 24). In the hydride region of the ^{1}H-NMR-spectrum the signal corresponding to **6** (bs, -8.8 ppm) was observed. Besides that, two small signals at -6.7 ppm (bs) and -9.4 ppm (bs) corresponding to yet unknown species were observed.

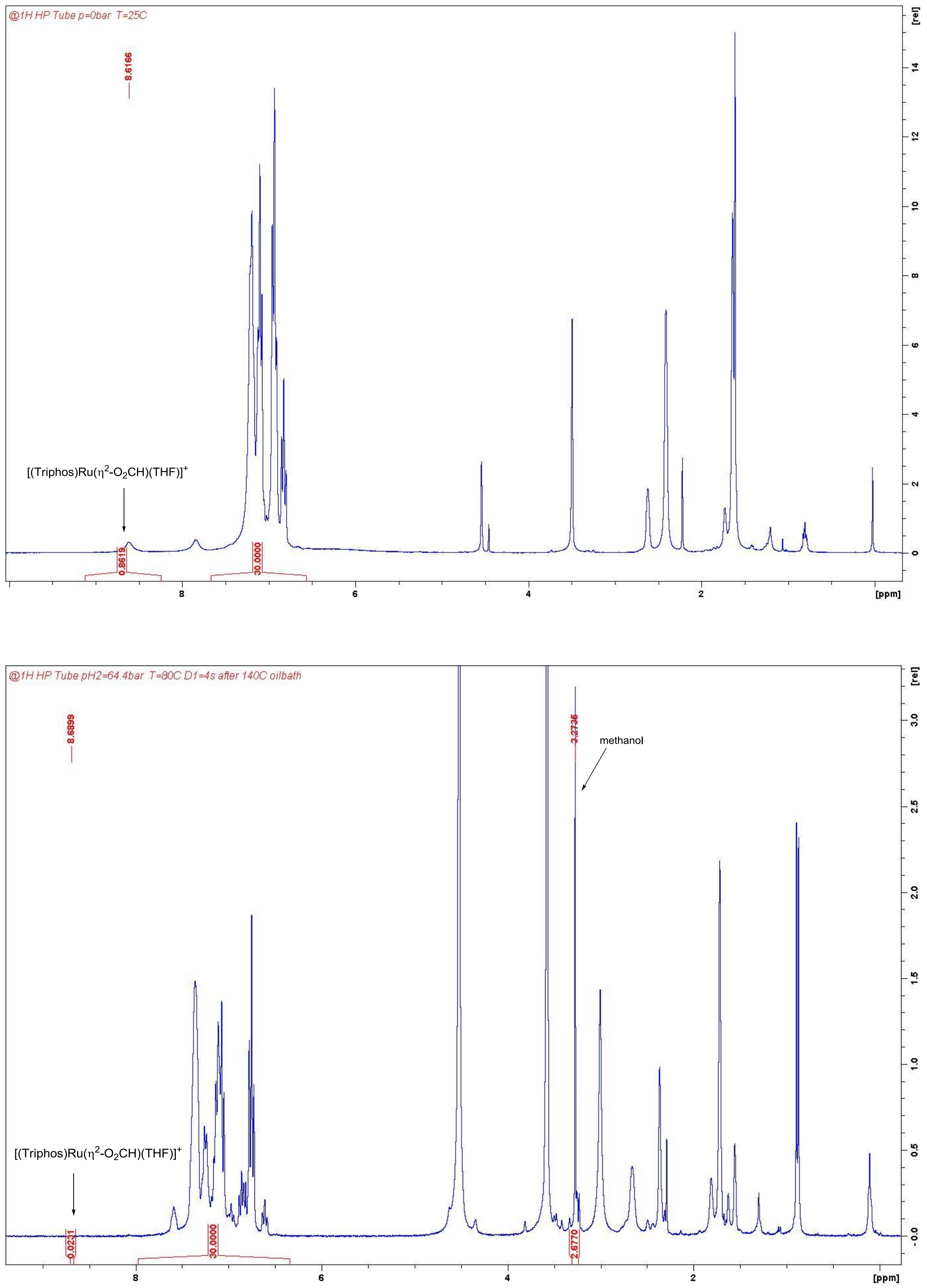

Figure 23: ^{1}H-NMR (300 MHz, d_8-THF) spectrum of the reaction mixture after addition of 1 equivalent $HNTf_2$ and 1 equivalent HCO_2H to complex **2** in d_8-THF measured in a high pressure NMR tube at room temperature before pressurising (top) and at 80 °C after heating to 140 °C in an oil-bath under 60 bar H_2-pressure (bottom).

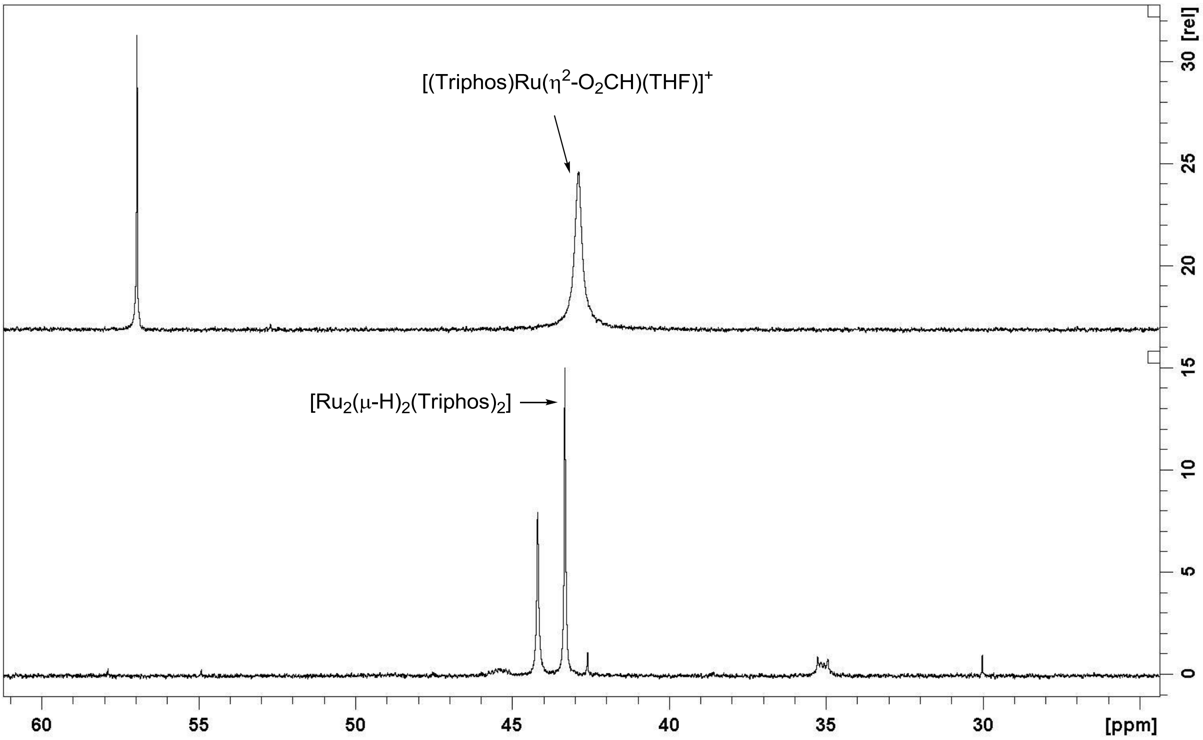

Figure 24: $^{31}P\{^{1}H\}$-NMR (121 MHz, *d*$_8$-THF, room temperature) spectrum of the reaction mixture after addition of 1 equivalent $HNTf_2$ and 1 equivalent HCO_2H to complex **2** in *d*$_8$-THF measured in a high pressure NMR tube at room temperature before pressurising (top) and at 80 °C after heating to 140 °C in an oil-bath under 60 bar H_2-pressure for 40 minutes (bottom).

6.4 In-situ NMR study of the CO_2 hydrogenation reaction using complex 2 with $HNTf_2$

$HNTf_2$ (3.5 mg, 0.0125 mmol) and complex **2** (9.8 mg, 0.0125 mmol, 1 eq.) were dissolved in *d*$_8$-THF (0.5 mL) at room temperature giving a deep red coloured solution. 0.3 mL of this solution were transferred to a high-pressure NMR tube (volume = 0.93 mL). Of this solution ^{1}H-NMR and $^{31}P\{^{1}H\}$-NMR spectra were recorded at 25 °C. After pressurising with 20 bar of carbon dioxide and 60 bar of hydrogen again spectra were recorded at room temperature. The NMR-tube was heated at 80 °C in the NMR-machine and ^{1}H-NMR and $^{31}P\{^{1}H\}$-NMR spectra were recorded directly and again after 1, 2 and 4 hours at 80 °C. The time shift between recording the ^{1}H-NMR spectra and the $^{31}P\{^{1}H\}$-NMR spectra was 30 minutes due to shimming and time for measuring the ^{1}H-NMR. After cooling to room temperature again a ^{1}H-NMR was recorded.

The recorded $^{31}P\{^{1}H\}$-NMR spectra are shown in Figure 25. The spectrum recorded at 0 bar and 25 °C showed two singlets at 48.6 and 58.8 ppm and no signal due to the initial complex **2**. We may speculate that the signal at 48.6 ppm is due to the cationic complex $[Ru(Triphos)(methylallyl)]^{+}$ resulting from protonation of the TMM ligand in complex **2**,[5] whereas the signal at 58.8 ppm might belong to a solvato complex $[Ru(Triphos)S_3]^{2+}$ forming after complete protonation of the TMM ligand to isobutene. After pressurising with H_2/CO_2 already at room temperature both singlets disappeared almost completely giving rise to a broad singlet with a shoulder at 43.8 ppm and after heating at 80 °C

only this broad singlet remained. The same signal was observed for the formate complex [(Triphos)Ru(η^2-O_2CH)(THF)]$^+$ **3a** in the investigations done before (vide supra). Formation of a small singlet at 43.2 ppm due to [Ru_2(μ-H)$_2$(Triphos)$_2$] **6** was also observed after 90 minutes at 80 °C.
Figure 26 shows a magnification of the formate-area of the recorded ^{1}H-NMR spectra. Already after pressurising CO_2/H_2 at room temperature a formate signal at 8.7 ppm appeared. Together with the ^{31}P{^{1}H}-NMR data formation of a certain amount of the formate complex [(Triphos)Ru(η^2-O_2CH)(THF)]$^+$ **3a** already at room temperature seems to be likely. At 80 °C the formate signal was strongly broadened indicating fluctuating formate species. At the same time formation of methanol was observed. After cooling again to room temperature, clearly the signal at 8.7 ppm due to the formate complex [(Triphos)Ru(η^2-O_2CH)(THF)]$^+$ **3a** was observed. This behaviour indicates that **3a** is a resting state.

In Figure 27 the hydride areas of the recorded ^{1}H-NMR spectra are depicted. In the spectra after 1 and 2 hours a very small signal at -6.7 ppm was observed. However, this signal disappeared again after two further hours, making it very unlikely to play a role in the catalytic transformation of CO_2 and H_2 to methanol. The hydride signal at -8.8 ppm forming after 60 minutes is due to [Ru_2(μ-H)$_2$(Triphos)$_2$] **6**.

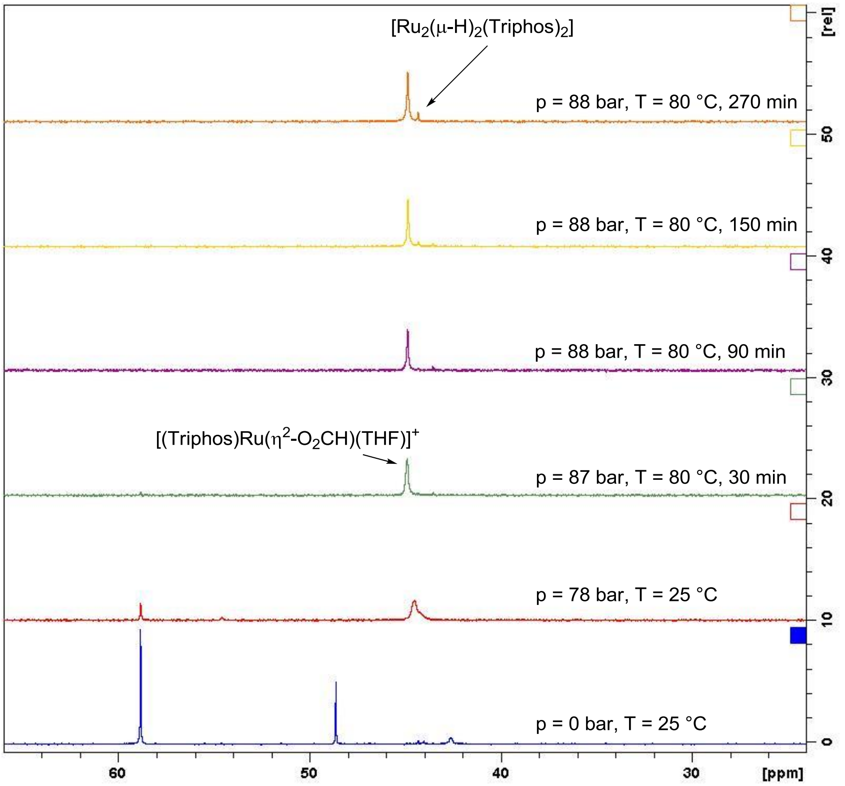

Figure 25: ^{31}P{^{1}H}-NMR (121 MHz, d_8-THF) spectra of a CO_2 hydrogenation reaction carried out in a high-pressure NMR-tube. Reaction conditions: c = 25 µmol/mL complex **2**, 1 equivalent HNTf$_2$, 0.3 mL d_8-THF, $p(CO_2)$ = 20 bar at r.t., $p(H_2)$ = 60 bar at r.t..

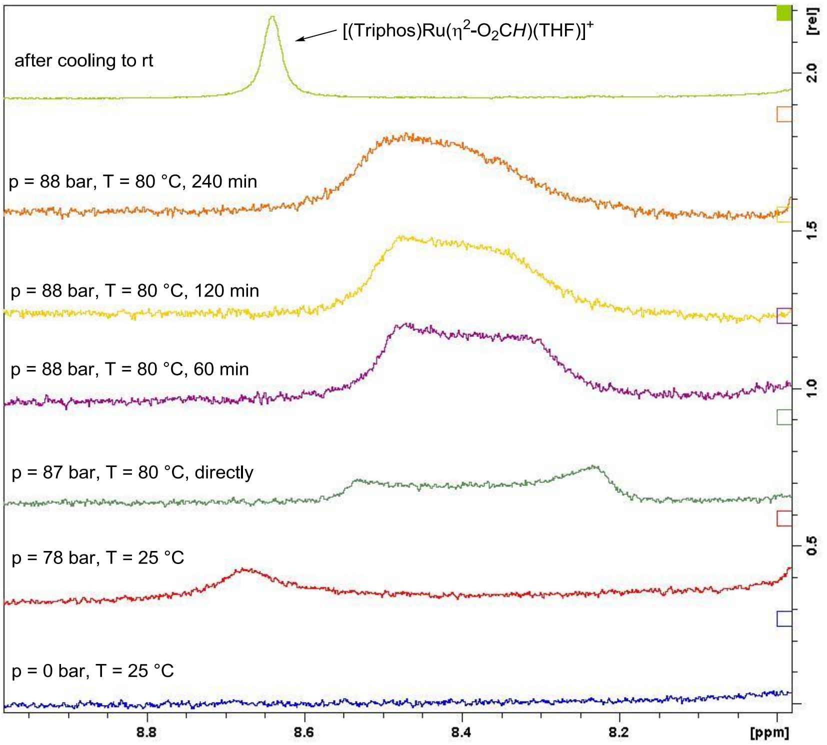

Figure 26: ^{1}H-NMR (300 MHz, d_8-THF) spectra of a CO_2 hydrogenation reaction carried out in a high-pressure NMR-tube. Magnification of the formate area. Reaction conditions: c = 25 µmol/mL complex **2**, 1 equivalent $HNTf_2$, 0.3 mL d_8-THF, $p(CO_2)$ = 20 bar, $p(H_2)$ = 60 bar.

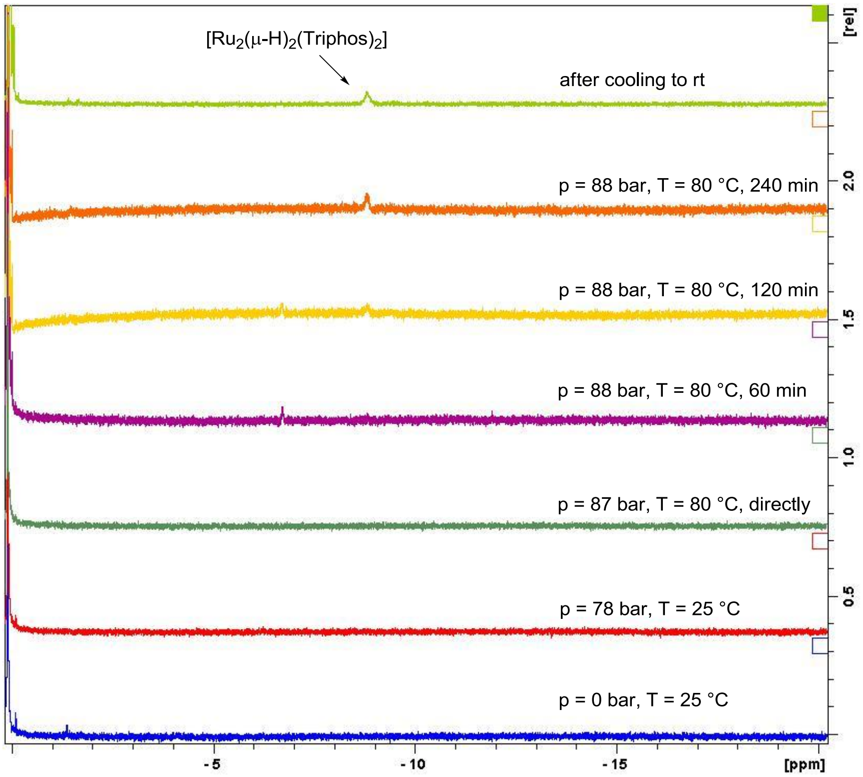

Figure 27: ^{1}H-NMR (300 MHz, d_8-THF) spectra of a CO_2 hydrogenation reaction carried out in a high-pressure NMR-tube. Magnification of the hydride area. Reaction conditions: c = 25 µmol/mL complex **2**, 1 equivalent $HNTf_2$, 0.3 mL d_8-THF, $p(CO_2)$ = 20 bar, $p(H_2)$ = 60 bar.

6.5 NMR-spectroscopic analysis of the reaction mixture using complex 2 together with *p*-TsOH

p-TsOH (0.0375 mmol, 1.5 eq.) and complex **2** (0.025 mmol) were dissolved in d_8-THF (1.0 mL) at room temperature giving a clear yellow solution. The solution was transferred to the autoclave and a CO_2 hydrogenation reaction performed at 140 °C, 60 bar (at r.t.) of hydrogen and 20 bar (at r.t.) of carbon dioxide. After 1 hour the reaction was terminated and the reaction solution analysed by NMR at -40 °C. The ^{31}P{1H}-NMR spectrum recorded at -40 °C (Figure 28) showed the same signals of the formate complex [(Triphos)Ru(η^2-O_2CH)(THF)]$^+$ **3a** as observed when $HNTf_2$ was used as acid additive. Compared to the spectrum shown in Figure 13 some new signals were observed: The triplet (42.0 ppm) and doublet (36.1 ppm) were assigned to the complex [(Triphos)Ru(*p*-TsO)$_2$] **14** based on NMR and mass spectrometric data. Additionally, two sets of three correlating doublet of doublets [assigned as a) and b) in Figure 28] were observed, indicating the formation of two different complexes with facially coordinated triphos-ligand and three different further ligands (eg. H, CO, THF). **14** could be prepared independently by addition of 2 equivalents of *p*-TsOH to complex **2** in THF at room temperature (Figures 29-30).

Analytical data of [(Triphos)Ru(*p*-TsO)$_2$] **14**:

^{1}H-NMR (400 MHz, d_8-THF, rt): δ = 7.71 (d, $^3J_{H\text{-}H}$ = 7.8 Hz, 4H, C_{Ar}-*H* in *p*-TsOH), 7.47 (br, 12H, C_{Ar}-*H* in P-Ph), 7.14 (m, 6H, C_{Ar}-*H* in P-Ph), 7.08 (d, $^3J_{H\text{-}H}$ = 7.8 Hz, 4H, C_{Ar}-*H* in *p*-TsOH), 6.97 (m, 12H, C_{Ar}-*H* in P-Ph), 2.40 (br, 6H, P-CH_2), 2.31 (s, 6H, CH_3 in *p*-TsOH), 1.54 [br, 3H, (Ph_2PCH_2)$_3$C-CH_3] ppm.

^{31}P{^{1}H}-NMR (162 MHz, d_8-THF, rt): δ = 36.4 (br, 3P) ppm.

^{31}P{^{1}H}-NMR (243 MHz, d_8-THF, -40 °C): δ = 40.5 (t, $J_{P\text{-}P}$ = 47.5 Hz, 1P), 36.5 (d, $J_{P\text{-}P}$ = 47.5 Hz, 2 P) ppm.

^{13}C{^{1}H}-NMR (100 MHz, d_8-THF, rt): δ = 135.4 (s, C_{Ar}), 134.9 (s, C_{Ar}), 134.5 (s, C_{Ar}), 132.2 (s, C_{Ar}-H), 128.6 (s, C_{Ar}-H), 127.7 (s, C_{Ar}-H), 127.5 (s, C_{Ar}-H), 126.2 (s, C_{Ar}-H), 37.0 [s, (Ph_2PCH_2)$_3$*C*-CH_3], 36.0 (m, *C*H_3), 32.5 (br, P-*C*H_2), 20.0 (s, CH_3 in *p*-TsOH) ppm.

MS(FAB) (+): m/z = 1067.2

MS(FAB) (-): m/z = 171.1

Measuring a ^{31}P{^{1}H}-NMR spectrum of [(Triphos)Ru(*p*-TsO)$_2$] **14** in d_3-MeCN yielded a sharp singlet at 27.3 ppm, which was assigned to the literature known solvato complex [(Triphos)Ru(MeCN)$_3$]$^{++}$ **16**.[6]

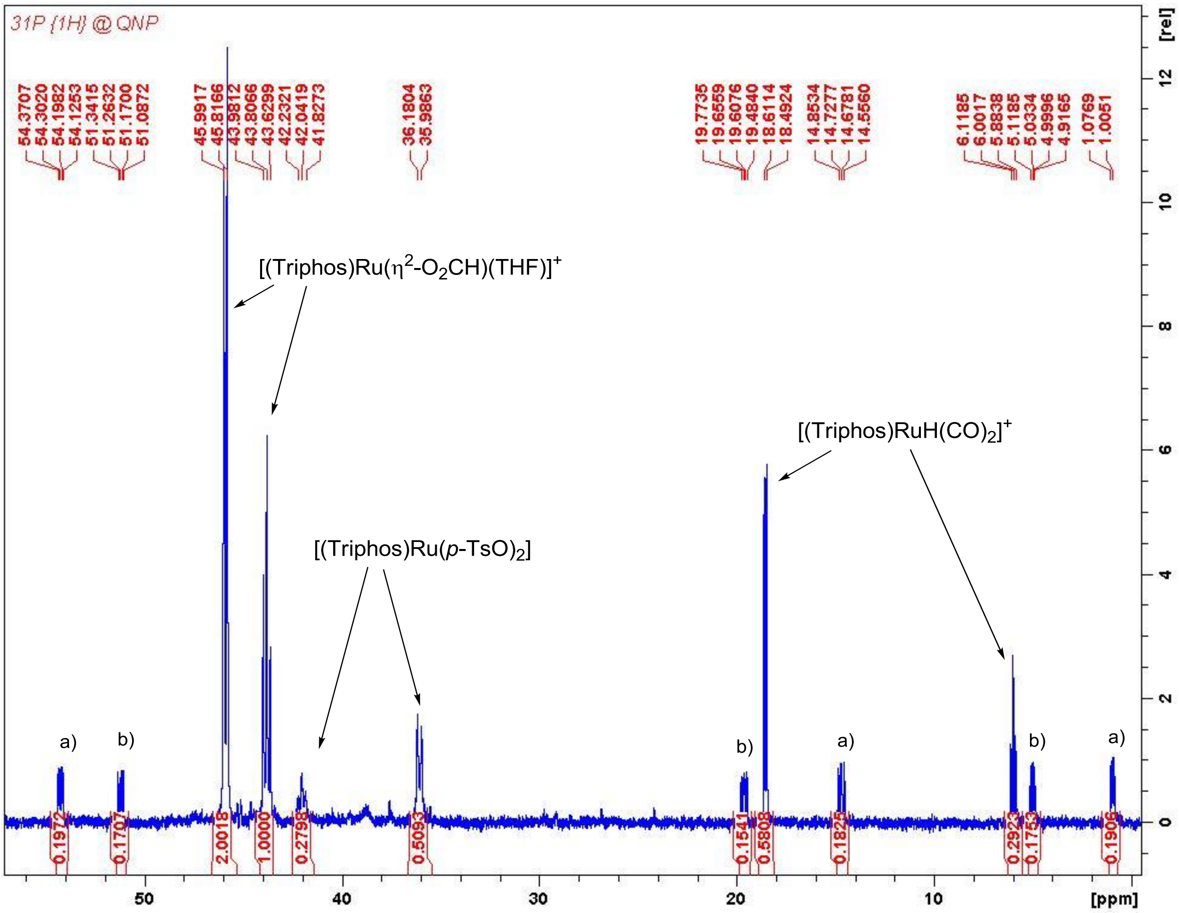

Figure 28: $^{31}P\{^1H\}$-NMR (243 MHz, d_8-THF, -40 °C) spectrum of the reaction solution of a CO_2-hydrogenation reaction (25 µmol complex **2**, 1.5 equivalents *p*-TsOH, 1.0 mL d_8-THF, $p(CO_2)$ = 20 bar, $p(H_2)$ = 60 bar, 140 °C, 1 h reaction time).

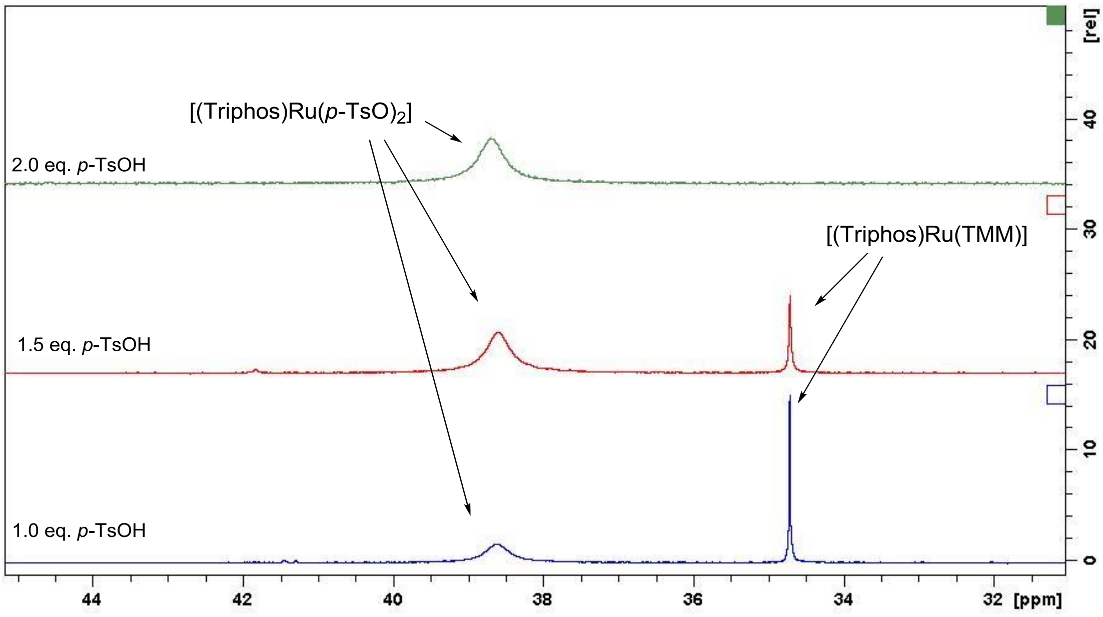

Figure 29: $^{31}P\{^1H\}$-NMR (162 MHz, d_8-THF, rt) spectra of the stepwise addition of *p*-TsOH to complex **2** in d_8-THF at room temperature.

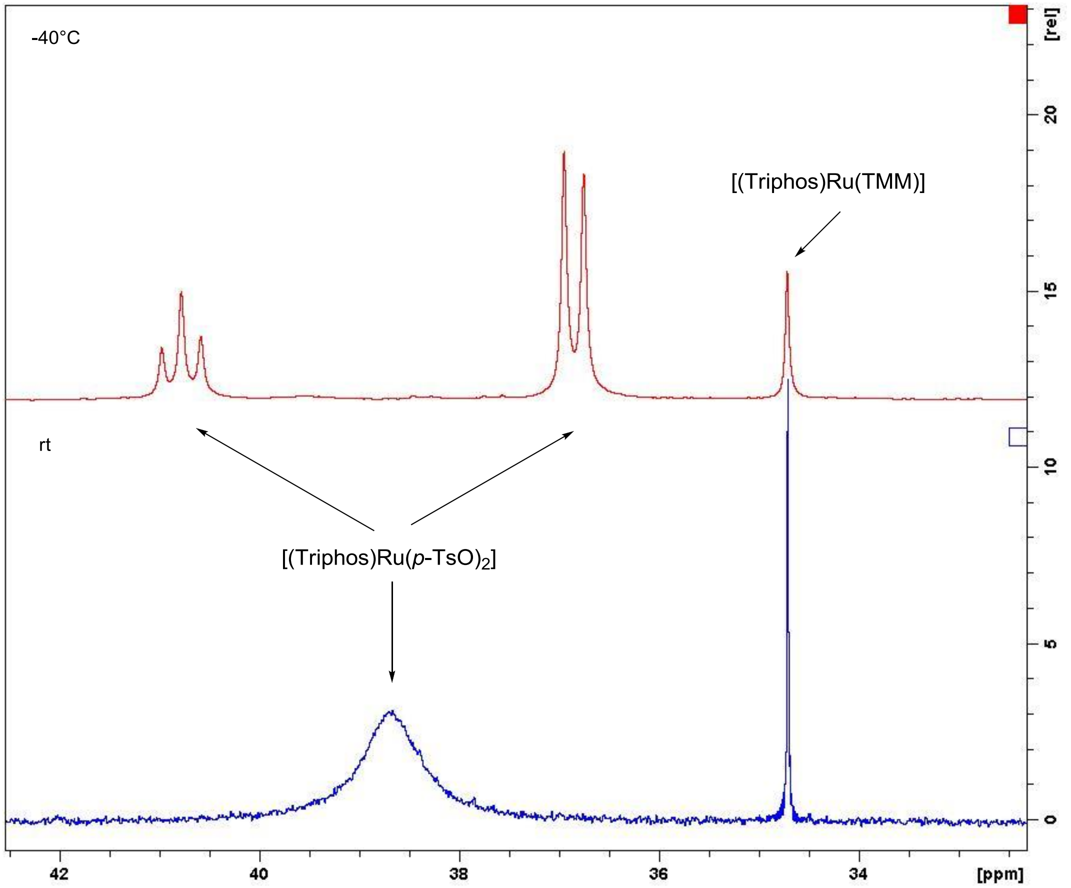

Figure 30: $^{31}P\{^1H\}$-NMR (243 MHz, d_8-THF) spectra of the reaction solution after addition of *p*-TsOH (1.5 eq.) to complex **2** in d_8-THF at room temperature (bottom) and -40 °C (top).

6.6 NMR-spectroscopic analysis of the reaction mixture using complex 2 in the absence of acid

A CO_2 hydrogenation experiment was performed using complex **2** in the absence of any acid additive and terminated after 1 hour (25 µmol complex **2**, 1.0 mL d_8-THF, $p(CO_2)$ = 20 bar at r.t., $p(H_2)$ = 60 bar at r.t., 140 °C). The reaction solution was analysed by NMR. The $^{31}P\{^1H\}$-NMR spectrum showed almost exclusively the formation of [(Triphos)Ru(H)$_2$CO] **12** (Figure 31). The hydride region of the ^1H-NMR showed the corresponding doublet of doublets (δ = -7.25 ppm, $J_{H\text{-}P}$ = 50.6 Hz, $J_{H\text{-}P}$ = 18.1 Hz).

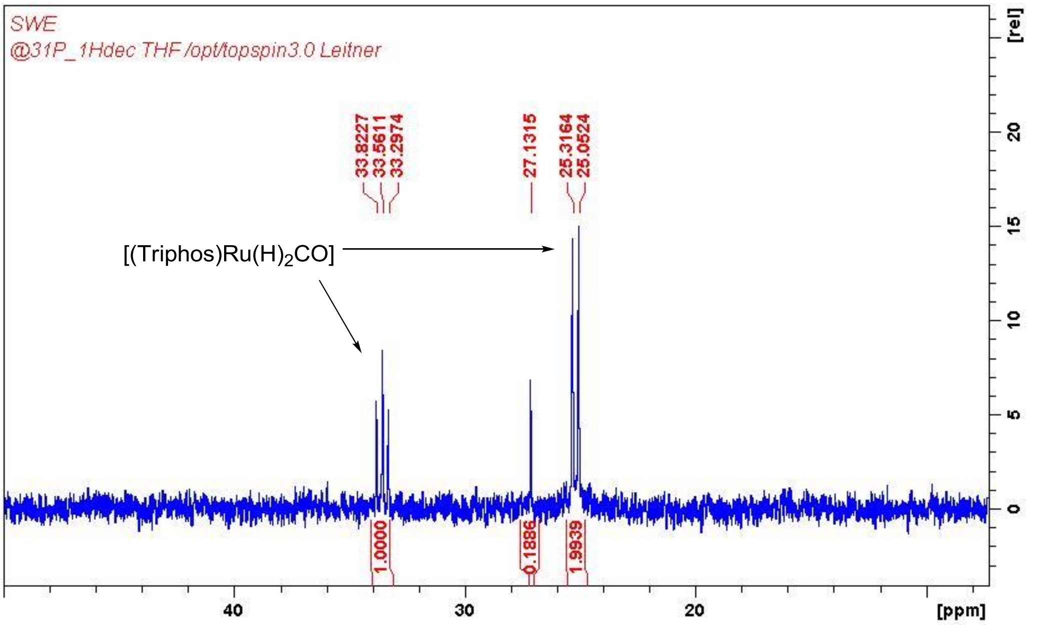

Figure 31: $^{31}P\{^1H\}$-NMR (121 MHz, d_8-THF, r.t.) spectrum of the reaction solution of a CO_2-hydrogenation reaction in the absence of acid additive (25 μmol complex **2**, 1.0 mL d_8-THF, $p(CO_2)$ = 20 bar at r.t., $p(H_2)$ = 60 bar at r.t., 140 °C, 1 h reaction time).

6.7 NMR-spectroscopic analysis of the reaction mixture using complex 12 together with $HNTf_2$

The catalytic hydrogenation of CO_2 to methanol using the carbonyl complex [(Triphos)Ru(H)$_2$CO] **12** was investigated using NMR-spectroscopy. After addition of one equivalent $HNTf_2$ in d_8-THF to [(Triphos)Ru(H)$_2$CO] **12** gas evolution was observed and the $^{31}P\{^1H\}$-NMR showed the formation of three doublets of doublets (Figure 32). In the ^1H-NMR the hydride signal of [(Triphos)Ru(H)$_2$CO] **12** (-7.36 ppm, dd, $J_{H\text{-}P}$ = 50.2 Hz, $J_{H\text{-}P}$ = 18.4 Hz) disappeared and a pseudo doublet of triplets appeared (-5.53 ppm, dt, $J_{H\text{-}P}$ = 88.0 Hz, $J_{H\text{-}P}$ = 17.7 Hz). Based on this data we tentatively propose the loss of a hydride ligand upon addition of the acid and formation of a solvate complex [(Triphos)Ru(H)(CO)S]$^+$ **17**.

After performing a CO_2 hydrogenation reaction using this mixture (25 μmol complex **12**, 1 equivalent $HNTf_2$, 1.0 mL d_8-THF, $p(CO_2)$ = 20 bar at r.t., $p(H_2)$ = 60 bar at r.t., 140 °C, 1 h reaction time) the reaction solution was analysed by $^{31}P\{^1H\}$-NMR at -40 °C. The spectrum (Figure 33) again showed the formation of the formate complex [(Triphos)Ru(η^2-O_2CH)(THF)]$^+$ **3a**.

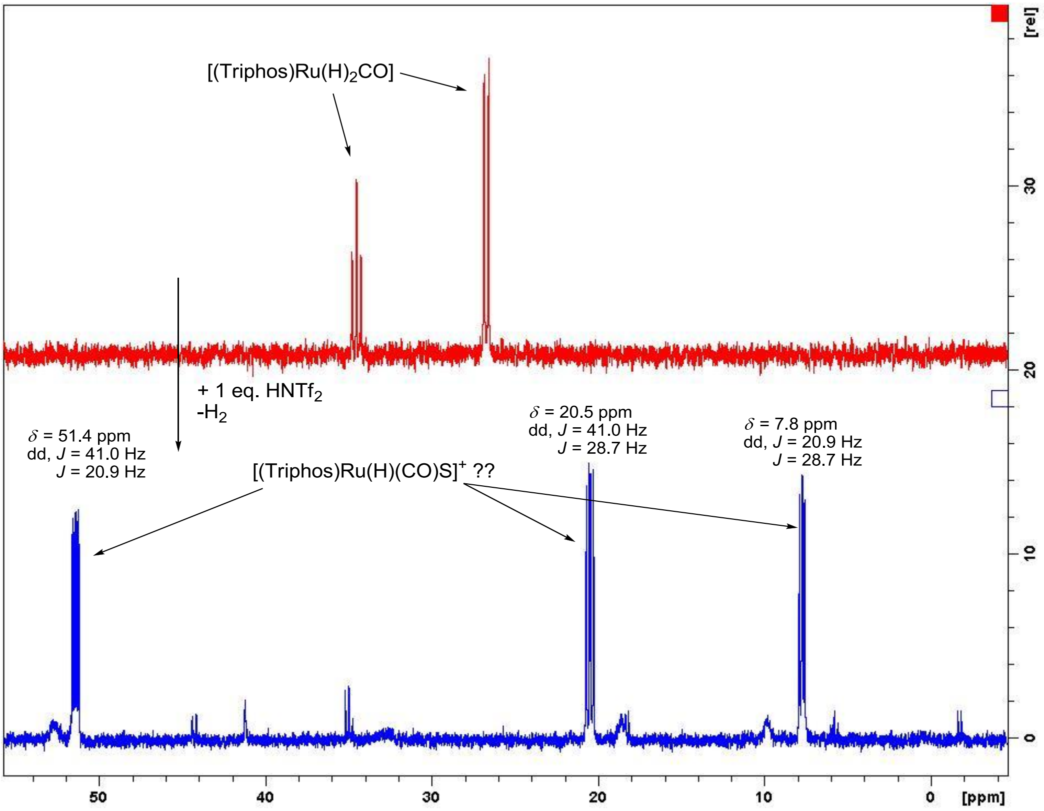

Figure 32: $^{31}P\{^1H\}$-NMR (162 MHz, d_8-THF, r.t.) spectrum before (top) and after (bottom) addition of 1 equivalent $HNTf_2$ to complex **12** in d_8-THF. S = THF, H_2O.

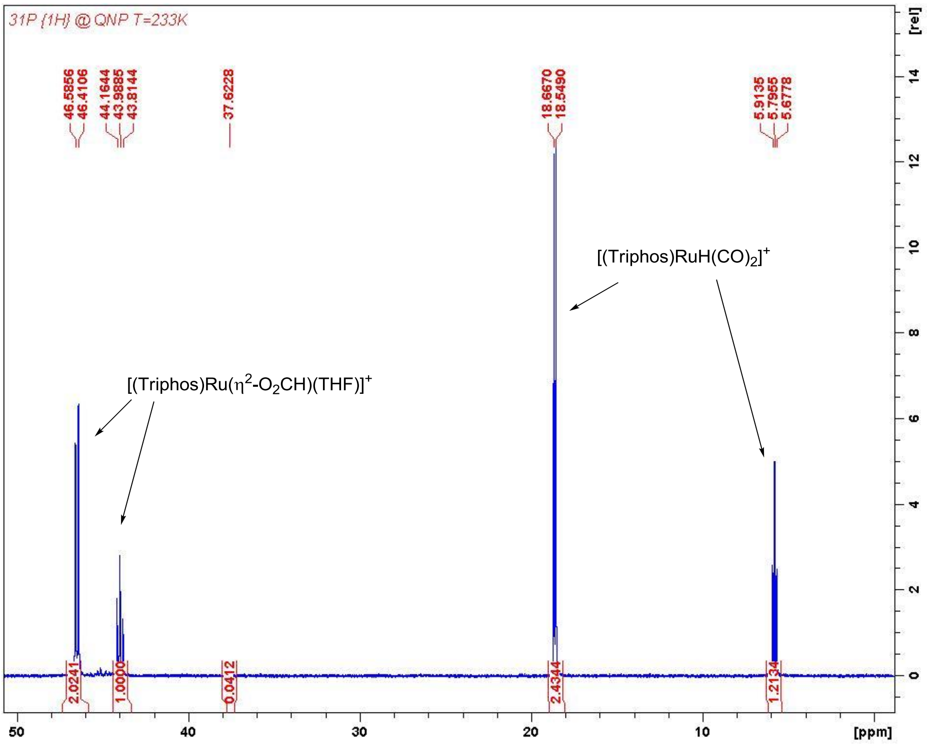

Figure 33: $^{31}P\{^1H\}$-NMR (243 MHz, d_8-THF, -40 °C) spectrum of the reaction solution of a CO_2-hydrogenation reaction (25 μmol complex **12**, 1 equivalent $HNTf_2$, 1.0 mL d_8-THF, $p(CO_2)$ = 20 bar at r.t., $p(H_2)$ = 60 bar at r.t., 140 °C, 1 h reaction time).

6.8 In-situ NMR study of the CO_2 hydrogenation reaction using complex 4

[(Triphos)Ru(η^2-OAc)(S)][NTf_2] **4** (13.3 mg, 0.0125 mmol) was dissolved in d_8-THF (0.5 mL) and 0.3 mL of this yellow solution transferred to a high-pressure NMR-tube (volume = 0.93 mL). Of this solution ^{1}H-NMR and ^{31}P{^{1}H}-NMR spectra were recorded at 25 °C. After pressurising with 20 bar of carbon dioxide and 60 bar of hydrogen again spectra were recorded at room temperature. The NMR-tube was heated at 80 °C in the NMR-machine and ^{1}H-NMR and ^{31}P{^{1}H}-NMR spectra were recorded directly and again after 1.5 hours at 80 °C. The NMR-tube was carefully (only the lower 4 cm of the tube containing the solution were dipped into the oilbath) heated at 140 °C in an external oil-bath for 1 hour and after that again measured at 80 °C. After cooling to room temperature further ^{1}H-NMR and ^{31}P{^{1}H}-NMR spectra were recorded at room temperature.

Figure 34 shows the recorded ^{31}P{^{1}H}-NMR spectra. At 25 °C and 0 bar the singlet due to the starting complex **4** was observed at 42.3 ppm. After pressurisation with CO_2/H_2 a small and broad singlet at 42.9 ppm appeared already at 25 °C. After heating at 80 °C inside the NMR-machine both signals merged and again only one broad singlet was observed at around 42 ppm. No changes were observed in the ^{31}P{^{1}H}-NMR spectra after heating for 1.5 hours at 80 °C and after heating for 1 hour at 140 °C. After cooling down to 25 °C two signals were observed again at 42.3 ppm and 42.9 ppm. The signal at 42.3 ppm integrated to around 40 % of the total intensity and was ascribed to the starting complex **4**. The broader signal at 42.9 ppm was assigned to the formate complex [(Triphos)Ru(η^2-O_2CH)(THF)]$^+$ **3a** as the formate signal of **3a** at 8.6 ppm was also observed in the ^{1}H-NMR (Figure 35).

Figure 35 shows a magnification of the formate area of the recorded ^{1}H-NMR spectra. Directly after pressurising with CO_2/H_2 at 25 °C a small signal at 8.6 ppm appeared. This was in line with the formation of a small signal at 42.9 ppm in the corresponding ^{31}P{^{1}H}-NMR spectrum, and indicated formation of a small amount of [(Triphos)Ru(η^2-O_2CH)(THF)]$^+$ **3a**. At the same time a very small broad hump at 10.6 ppm (not shown in the magnification) was observed in the ^{1}H-NMR, indicating that a very little amount of free carboxylic acid was released into the solution. Upon heating to 140 °C this signal disappeared. Directly after heating to 80 °C, a singlet at 8.1 ppm appeared which became broader after heating to 140 °C. After cooling to 25 °C, this broad singlet disappeared and two singlets at 7.6 ppm and 8.6 ppm were observed. The signal at 8.6 ppm was assigned to the formate complex [(Triphos)Ru(η^2-O_2CH)(THF)]$^+$ **3a** taking into account the corresponding ^{31}P{^{1}H}-NMR spectrum. The singlet at 7.6 ppm was assigned to methyl formate, which is formed in small amounts once methanol is present in the reaction mixture, especially at lower temperatures. Methyl formate does not accumulate in the reaction mixture, as it is also hydrogenated to methanol. However, this reaction pathway does not seem to be significant, as no autocatalytic effect was observed during the course of the reaction.

In the ^{1}H-NMR spectrum directly after heating to 80 °C formation of methanol (s, 3.2 ppm) from CO_2 hydrogenation was observed as well as formation of ethanol (t, 1.1 ppm) from hydrogenation of the acetate ligands of starting complex **4**.

In Figure 36 a magnification of the hydride region of the recorded ^{1}H-NMR spectra is depicted. No hydride species was observed in any of the ^{1}H-NMR spectra.

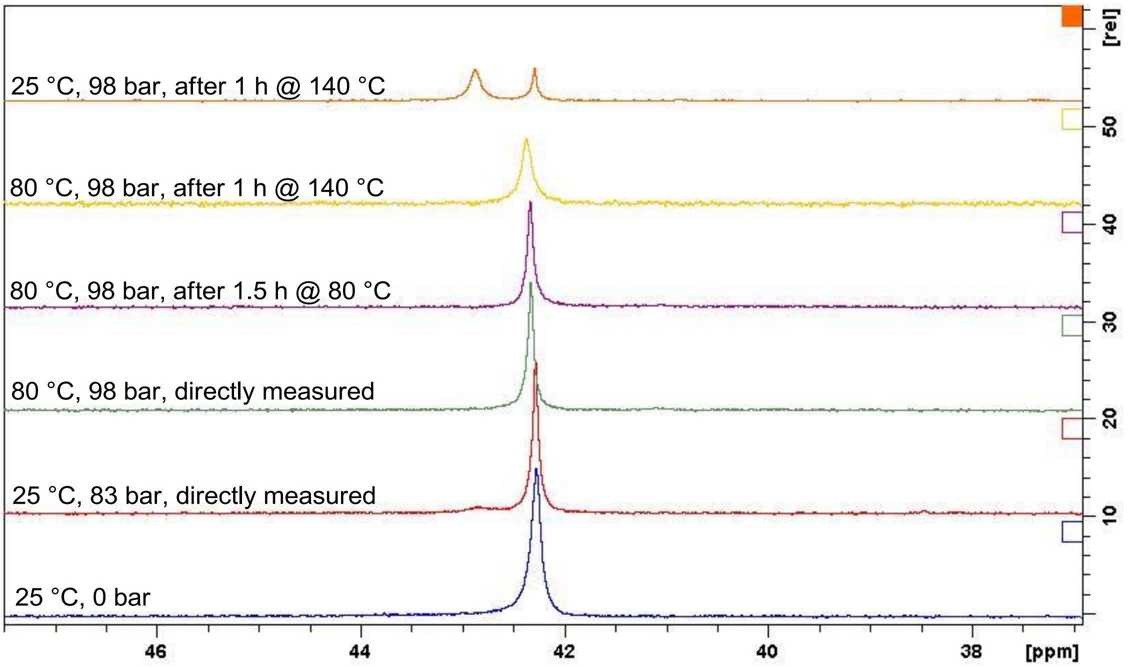

Figure 34: $^{31}P\{^{1}H\}$-NMR (121 MHz, d_8-THF) spectra of a CO_2 hydrogenation reaction carried out in a high-pressure NMR-tube. Reaction conditions: *c* = 25 μmol/mL complex **4**, 0.3 mL d_8-THF, $p(CO_2)$ = 20 bar at r.t., $p(H_2)$ = 60 bar at r.t..

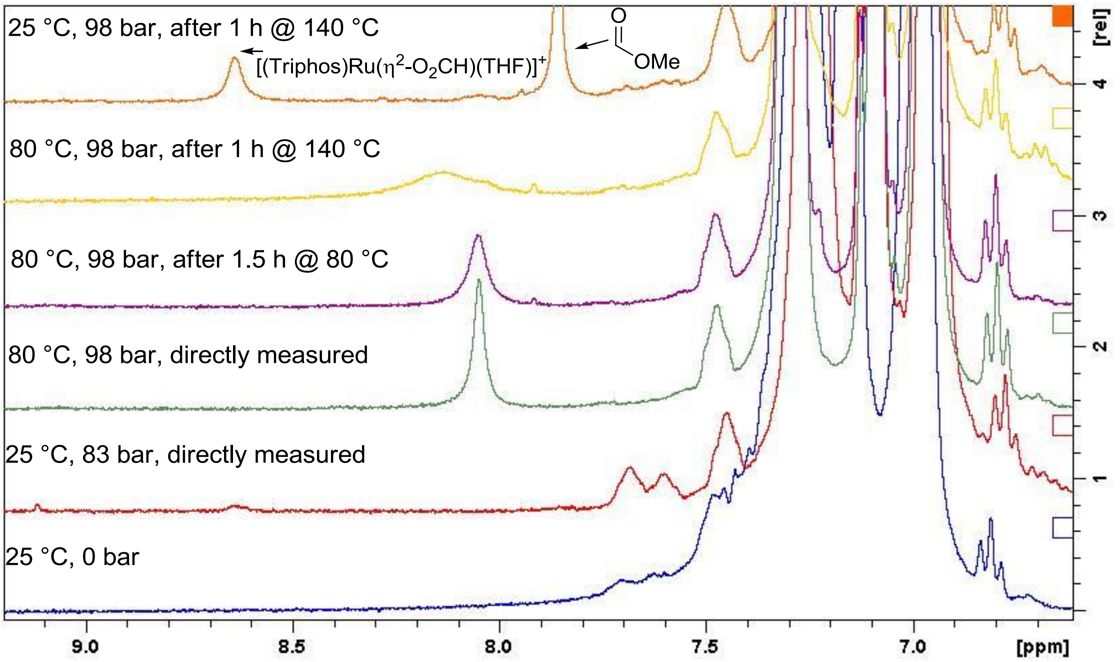

Figure 35: ^{1}H-NMR (300 MHz, d_8-THF) spectra of a CO_2 hydrogenation reaction carried out in a high-pressure NMR-tube. Reaction conditions: c = 25 μmol/mL complex **4**, 0.3 mL d_8-THF, $p(CO_2)$ = 20 bar at r.t., $p(H_2)$ = 60 bar at r.t.. Magnification of the formate area.

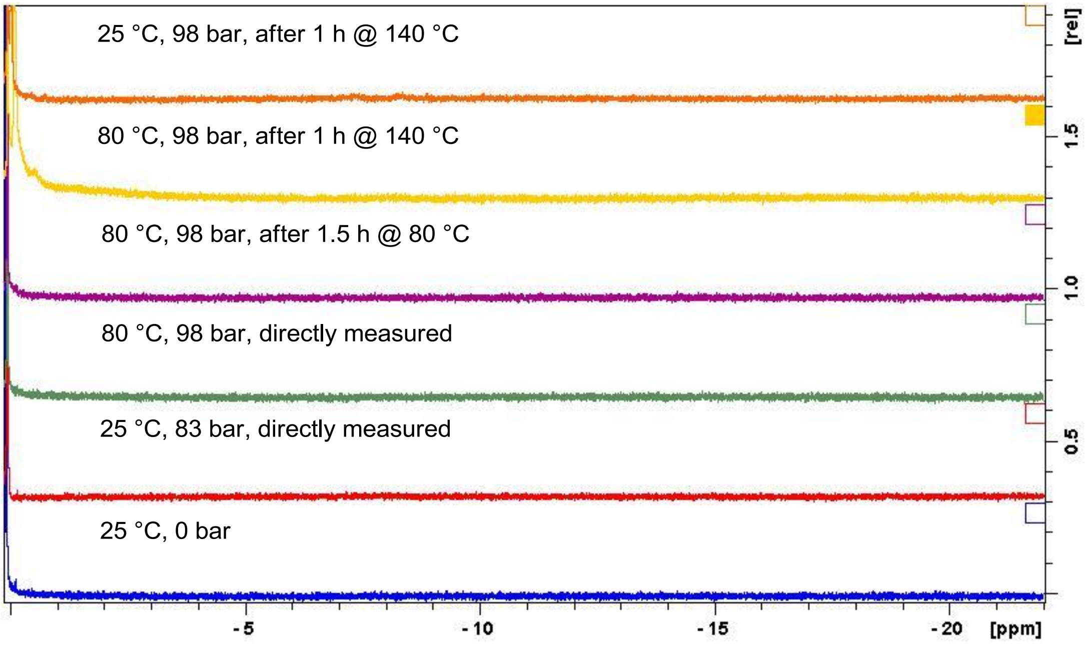

Figure 36: ^{1}H-NMR (300 MHz, d_8-THF) spectra of a CO_2 hydrogenation reaction carried out in a high-pressure NMR-tube. Reaction conditions: c = 25 μmol/mL complex **4**, 0.3 mL d_8-THF, $p(CO_2)$ = 20 bar at r.t., $p(H_2)$ = 60 bar at r.t.. Magnification of the hydride area.

7 Repetitive-Batch experiments using 2-MTHF/H_2O

7.1 Extraction of MeOH from 2-MTHF with water

An extraction experiment was conducted as follows: MeOH (0.2 mL, 158 mg, 4.94 mmol) was dissolved in 2-MTHF (1.0 mL, 892 mg). Water (1.0 mL, 996 mg) was added, the solution stirred (ca. 20 seconds) and the phases separated (after ca. 10 minutes). Phase separation was fast and two clear phases were obtained. The phases were analysed for their methanol content using mesitylene as internal standard in d_6-acetone via ^{1}H-NMR.

The 2-MTHF phase (765 mg) contained 20 % of the overall MeOH (0.98 mmol), whereas the water phase (1.281 g) contained 80 % of the overall MeOH (3.89 mmol). The mass balance was closed to 99 %.

7.2 Repetitive batch experiment using complex 2 together with $HNTf_2$ in 2-MTHF

Catalyst **2** (0.0125 mmol) and $HNTf_2$ (0.0125 mmol, 1 eq.) were dissolved in 2-MTHF (2.0 mL) and a CO_2 hydrogenation reaction performed like described above ($p(CO_2)$ = 20 bar at r.t., $p(H_2)$ = 60 bar at r.t., 140 °C). After 16 hours the reaction was stopped by cooling the autoclave in an ice/water bath and subsequent venting of the autoclave to release the remaining pressure. The autoclave was connected to a Schlenk line and the reaction solution transferred to a Schlenk tube via cannula. Degassed water (2.0 mL) was added and the solution stirred for ca. 20 seconds (Figure 37). After 10 minutes the phases were separated. The almost clear water phase was weighed and analysed for its methanol content via ^{1}H-NMR analysis using mesitylene as internal standard in d_6-acetone. The orange, clear 2-MTHF phase was transferred back to the autoclave. 2-MTHF (0.25 mL) was added to compensate the loss of 2-MTHF in the water phase. The second, third, and fourth batch experiment were run under the same conditions as the first one.

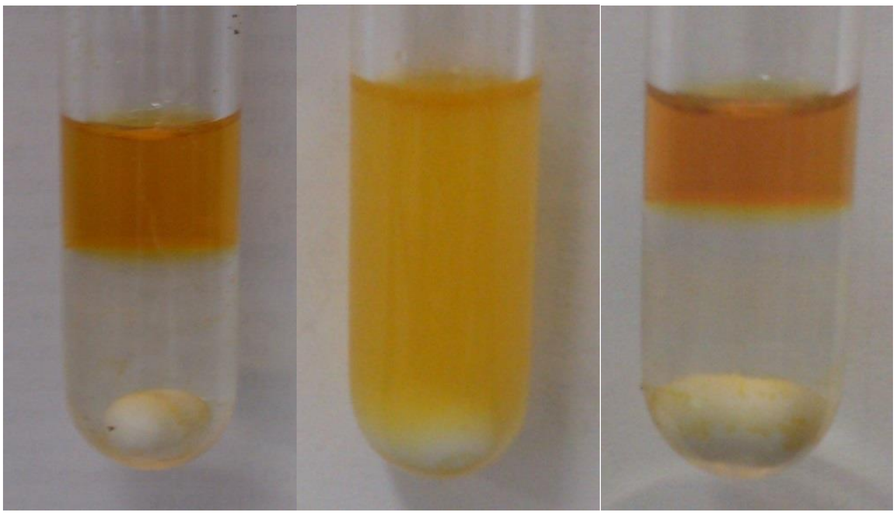

Figure 37: Phase behavior during the extraction of methanol from the 2-MTHF phase containing catalyst **2** and $HNTf_2$. The orange colour of the MTHF phase results from the dissolved catalyst. Left: after addition of H_2O to the 2-MTHF phase; middle: during mixing; right: phase separation after 10 minutes.

8 Additional catalysis results

Table S1. Additional variation of reaction parameters in the catalytic hydrogenation of carbon dioxide to methanol.[a]

Entry	Cat	Acid (eq)	T [°C]	p_{H_2} / p_{CO_2} [b] [bar/bar]	Solvent	TON[c]
1	**2**	*p*-TsOH (1.5)	140	20/60	THF	152
2	**2**	*p*-TsOH (2.0)	140	20/60	THF	115
3	**2**	MSA (1.0)	140	20/60	THF	61
4	**2**	MSA (1.5)	140	20/60	THF	68
5	**2**	MSA (2.0)	140	20/60	THF	20
6	**2**	$HNTf_2$ (1.0)	140	20/60	dioxane	194
7	**2**	$HNTf_2$ (1.0)	140	20/60	toluene	11
8[d]	**2**	$HNTf_2$ (1.0)	140	20/60	2-MTHF	295
9[e]	**2**	$HNTf_2$ (1.0)	140	20/60	2-MTHF	403

[a] Reaction conditions: Catalyst (25 µmol), 2.08 mL solvent, 24 h; [b] = at room temperature; [c] TON = mmol MeOH/mmol catalyst; [d] 12.5 µmol catalyst; [e] 6.3 µmol catalyst.

9 DFT Calculations

9.1 Computational Details

All calculations in this work were carried out with the Gaussian09 program series (Revision C.01 and D.01).[7]

Gas phase calculations:
The geometries of all structures were optimised with no constraints or restraints using the M06-L density functional[8] and the def2-SVP[9] basis set with the associated ECP[10] for ruthenium. The automatic density fitting approximation was activated.[11] The structures were characterised by frequency calculations to be local minima (i = 0) or transition states (i = 1). The obtained energies are listed in Table S2 below. For most of the optimised transition states IRC calculations were performed to ensure the transition state to connect the independently localised local minima. Thermochemical corrections were computed for standard state conditions. Additionally, single point energies were obtained on the M06-L def2-TZVP level of theory. The thermochemical corrections from the lower-level geometry optimisations were added to the electronic energies of the higher-level single-point calculations to arrive at corrected values for the Gibbs free energies. These are listed in Table S2 and are used for the discussion in the main text and in the figures in the main text. Figure 3 of the main manuscript is repeated here (Figure 38) to show three additional high energy pathways leading from **I** to **VIII**.

Solvent phase calculations:
While the study was ongoing Gaussian09 in Revision D.01 became available to us, which contains the MN12-L density functional. MN12-L has been tested to describe transition metal thermochemistry very accurately[12]. Accordingly, solvent phase calculations were calculated using MN12-L. The geometries of selected gas phase structures were optimised with no constraints or restraints using the MN12-L density functional and the def2-TZVP basis set[9] with the associated ECP[10] for ruthenium. The automatic density fitting approximation was activated.[11] Solvent effects (THF) were considered implicitly by applying the IEF-PCM as well as the CPCM[13] formalism. The structures were characterised by frequency calculations to be local minima (i = 0) or transition states (i = 1). Thermochemical corrections were computed for a temperature of 413.15 K. A pressure of 302 atm was specified to account for entropy corrections in the condensed phase as was described elsewhere.[14] The obtained energies are listed in Table S3 below.

9.2 Hydrogenation of CO_2 to Methanediolate

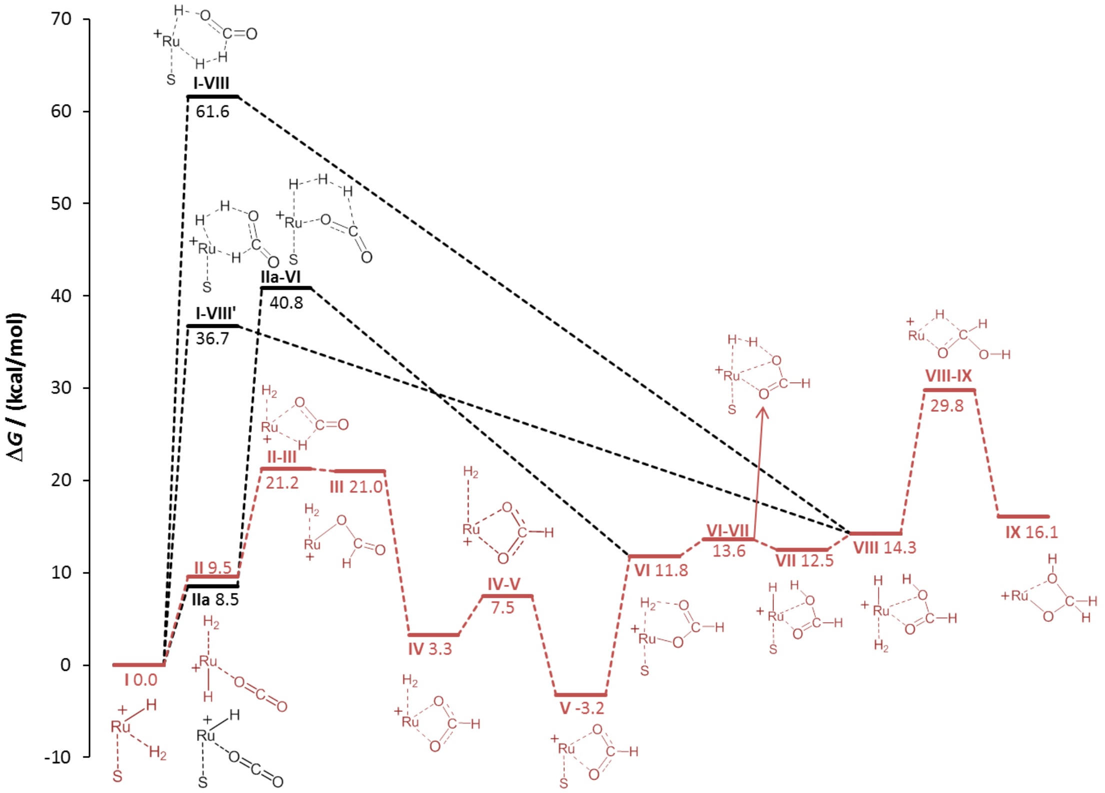

Figure 38: Calculated reaction pathways generating formic acid complex **VIII** and methane-diolate complex **IX**. The Triphos ligand is omitted for clarity, S = THF.

9.3 Cleavage of the C-O bond and generation of formaldehyde – Protonolysis within the coordination sphere of Ru

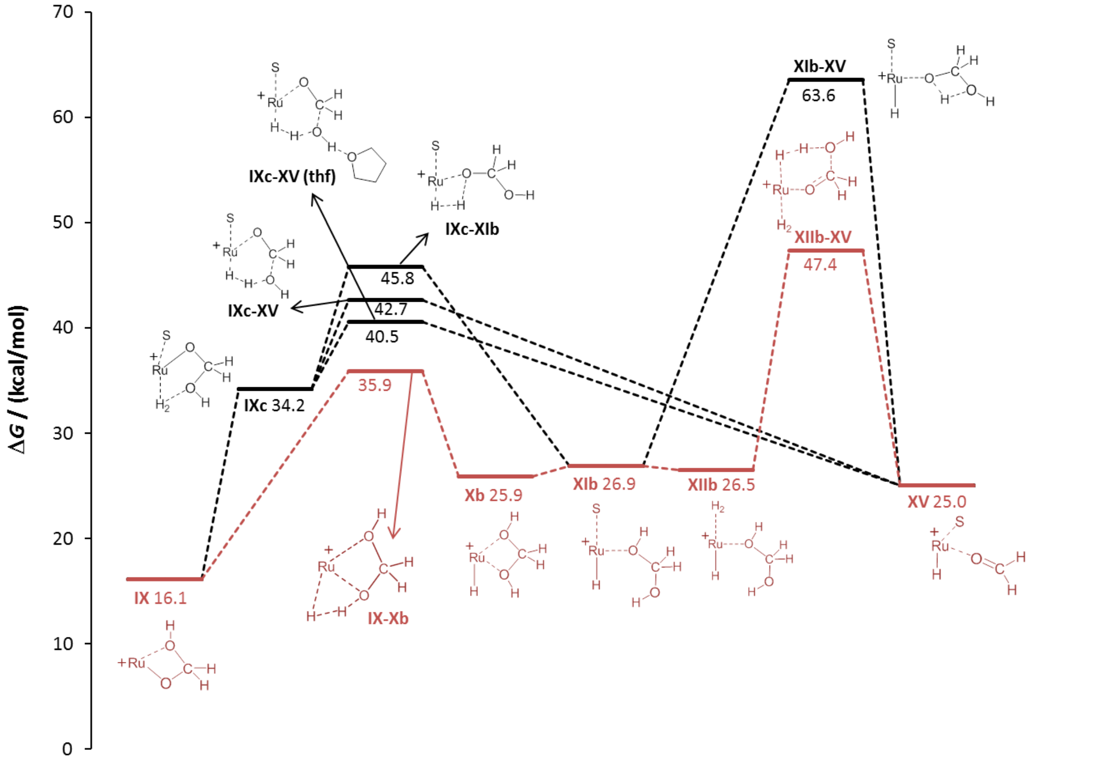

Figure 39: Calculated reaction pathways generating formaldehyde complex **XV** *via* intramolecular proton transfer. The Triphos ligand is omitted for clarity, S = THF.

Cleavage of the C-O bond and generation of formaldehyde (IX – XV; Figure 39): In addition to the external proton transfer (as shown in Figure 4 in the main manuscript), direct protonolysis within the coordination sphere through heterolytic cleavage of the coordinated H_2-molecule was also investigated (Figure 39, ESI). Addition of H_2 to **IX** and heterolytic H_2 cleavage *via* the four-membered transition state **TSIX-Xb** (35.9 kcal/mol) leads to complex **Xb** (25.9 kcal/mol) with the resulting hydroxymethanolate coordinating to the metal via both oxygen atoms. After dissociation of one hydroxy group from the metal the vacant coordination site in **Xb** can either be occupied by solvent (**XIb**, 26.9 kcal/mol) or by H_2 (**XIIb**, 26.5 kcal/mol). Subsequently, the proton of one OH group transfers to the classical hydride centre of **XIIb** forming a H_2 molecule, while the second hydroxy group accepts a proton from the H_2 molecule at the metal centre. The transition state **TSXIIb-XV** leading to the C-O cleavage under formation of water defines the highest point of this energy profile (47.4 kcal/mol). The water molecule dissociates from the complex to give the formaldehyde complex **XV** (25.0 kcal/mol), in which solvent again has taken the place of the H_2 molecule. An alternative intramolecular pathway for the proton transfer from the free OH group in complex **XIb** to the coordinated OH would have to pass *via* **TSXIb-XV** at 63.6 kcal/mol, ruling this pathway out.

As complex **XV** comprises a coordinated THF molecule, pathways where the solvent molecule interacts with the active centre from the beginning were also investigated. Coordination of THF to **IX** results in **IXc** (34.2 kcal/mol). The intermediate **XIb** is reached from there over a barrier of 45.8 kcal/mol and still requires passing through the following high barrier for the C-O cleavage. However, starting from **IXc** also offers the possibility to lead the system directly *via* **TSIXc-XV** to complex **XV** (25.0 kcal/mol). The transition state **TSIXc-XV** involves the proton transfer together with the C-O bond cleavage and resides at a height of 42.7 kcal/mol on the hyper surface. Involving an additional THF molecule to assist the intramolecular proton transfer by forming a hydrogen bridge lowers the barrier even further to 40.5 kcal/mol in **TSIXc-XV(thf)**.

9.4 Influence of the coordination geometry (facial vs. meridional)

To obtain an impression of how large the influence of the coordination geometry (fac or mer) is on one of the key steps of the catalytic cycle presented in the main manuscript we constructed a model ligand, being similar to Triphos chemically, however, with no interconnected carbon backbone to allow for the setup of calculations in the facial and the meridional coordination mode. When three of such $P(Ph)_2(Me)$ model ligands are used to construct the complexes which correspond to the structures **VIII**, **TSVIII-IX** and **XI** (Figure 3 in the main paper), the barrier heights for the facial and the meridional reaction pathways can be computed independently. It is interesting to note that the model complex in the facial coordination mode generates a barrier height of ca. 15 kcal/mol for the transfer of the ruthenium bound hydride centre to the carbon atom of the formic acid molecule leading to the methandiolate complex **IX**. This is in very nice accordance with the barrier computed for the Triphos complex indicating the model ligand not to change the electronic and steric requirements markedly. If, however, the same reaction is computed for the meridional case the barrier height has a value of ca. 40 kcal/mol. This finding corroborates nicely the assumption that it is the facial coordination mode of Triphos which creates the necessary electronic and spatial requirements for the reaction to proceed at comparatively low barriers.

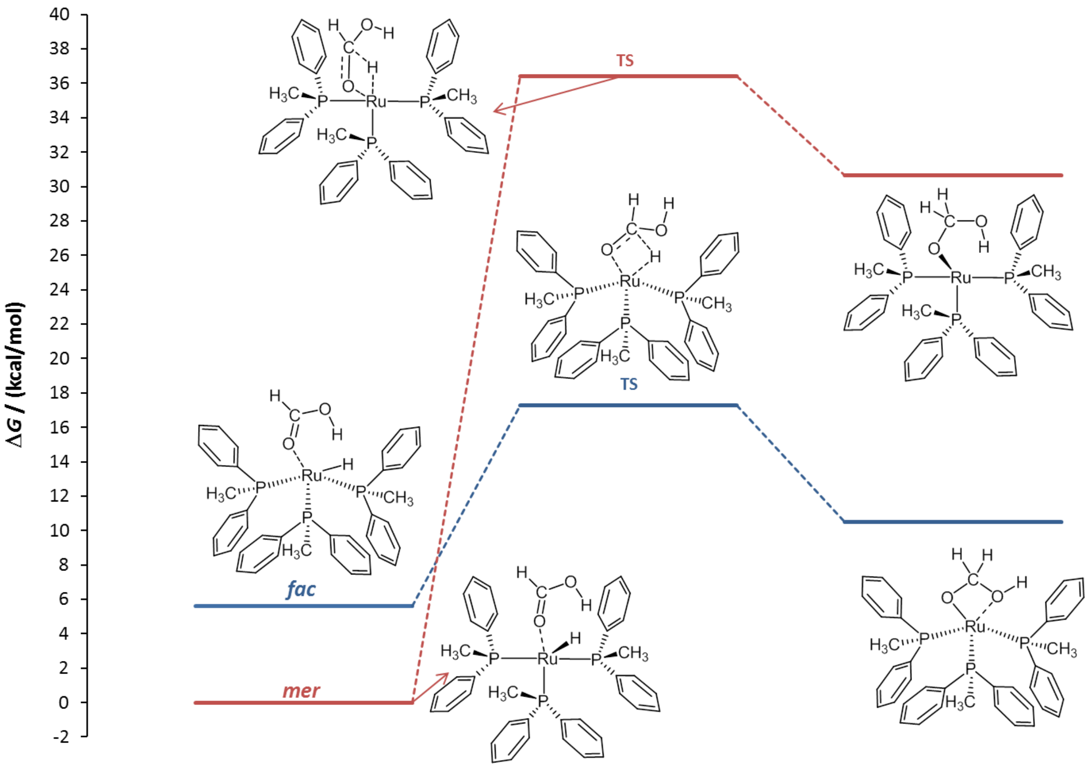

Figure 40: Recalculation of the structures **VIII**, **TSVIII-IX** and **XI** (Figure 3 in the main paper) using $P(Ph)_2(Me)$ as model ligand to compare meridional with facial coordination.

9.5 Calculated Energies

Table S2. Calculated energies of the optimised structures (M06-L/def2-SVP) and single point energies (M06-L/def2TZVP) in Hartree and relative Gibbs free energies G_{rel} (kcal/mol) belonging to Figures 3-5 in the main text and to Figure 38 and 39 in the ESI.

**Compd.**	***E***	***E***	***H***	***G***	**A**	***E*(def2-TZVP)**	***E*(def2-TZVP)+A**	***G***
Figure 3 main text + Figure 38 ESI					***G-E***		***G*(def2-TZVP)**	
**I**	-2937.206033	-2936.377217	-2936.328731	-2936.458045	0.747988	-2939.482418	-2938.734430	0.0
**I-VIII**	-3125.563800	-3124.725010	-3124.673634	-3124.809075	0.754725	-3128.053256	-3127.298531	61.6
**I-VIII'**	-3125.610071	-3124.767369	-3124.716309	-3124.850186	0.7598852	-3128.098067	-3127.338182	36.7
**IIa**	-3124.458009	-3123.633709	-3123.581939	-3123.719914	0.738094	-3126.948137	-3126.210043	8.5
**II**	-2893.374980	-2892.652286	-2892.605887	-2892.731434	0.643546	-2895.614406	-2894.970859	9.5
**IIa-VI**	-3125.597431	-3124.758624	-3124.707247	-3124.842332	0.755099	-3128.086784	-3127.331685	40.8
**II-III**	-2893.356804	-2892.635432	-2892.589702	-2892.713490	0.643314	-2895.595541	-2894.952227	21.2
**III**	-2893.358934	-2892.635158	-2892.589129	-2892.714388	0.644546	-2895.597173	-2894.952627	21.0
**IV**	-2893.392334	-2892.665937	-2892.620231	-2892.743098	0.649236	-2895.630074	-2894.980839	3.3
**IV-V**	-2893.376549	-2892.654674	-2892.608094	-2892.731850	0.644699	-2895.618784	-2894.974085	7.5
**V**	-3124.490276	-3123.661103	-3123.610669	-3123.742931	0.747345	-3126.976054	-3126.228709	-3.2
**VI**	-3125.652190	-3124.806393	-3124.754952	-3124.890142	0.762048	-3128.139997	-3127.377949	11.8
**VI-VII**	-3125.649428	-3124.805717	-3124.754801	-3124.888337	0.761091	-3128.136126	-3127.375035	13.6
**VII**	-3125.652301	-3124.805299	-3124.753894	-3124.889608	0.762693	-3128.139533	-3127.376840	12.5
**VIII**	-2894.565787	-2893.820128	-2893.773759	-2893.898708	0.667079	-2896.803519	-2896.136440	14.3
**VIII-IX**	-2894.535099	-2893.790785	-2893.744679	-2893.869210	0.665889	-2895.588049	-2894.938634	29.8
**IX**	-2894.547663	-2893.798549	-2893.752559	-2893.875654	0.672009	-2895.614582	-2894.960368	16.1
Figure 4								
**X**	-3122.295391	-3121.499208	-3121.448289	-3121.582862	0.712529	-3124.785699	-3124.073170	17.8
**X-XI**	-3047.094244	-3046.313073	-3046.262588	-3046.396804	0.697440	-3124.767558	-3124.058387	27.1
**XI**	-3122.306312	-3121.513793	-3121.461313	-3121.597752	0.708560	-3124.799505	-3124.090945	6.7
**XII**	-3045.936148	-3045.168599	-3045.119094	-3045.250846	0.685302	-3048.341539	-3047.656237	3.9
**XIII**	-3048.305615	-3047.497405	-3047.446102	-3047.579741	0.725874	-3049.502956	-3048.803106	20.3

**XIII-XIV**	-3047.094244	-3046.313073	-3046.262588	-3046.396804	0.697440	-3049.499977	-3048.802538	20.7
**XIV**	-3047.099824	-3046.314356	-3046.263661	-3046.398241	0.701583	-3049.505590	-3048.804008	19.8
**Xa**	-2969.753251	-2968.994888	-2968.947252	-2969.075221	0.678030	-2972.075504	-2971.397473	17.4
**Xa-XIa**	-2969.731847	-2968.977641	-2968.930300	-2969.057648	0.674199	-2972.056166	-2971.381967	27.2
**XIa**	-2969.730178	-2968.976287	-2968.927121	-2969.057140	0.673038	-2972.057314	-2971.384276	25.7
**XIIa**	-2893.353148	-2892.624575	-2892.578229	-2892.702284	0.650864	-2895.593176	-2894.942312	27.5
**XIIIa**	-2894.531684	-2893.786880	-2893.739380	-2893.865223	0.666461	-2896.773001	-2896.106540	33.0
**XIIIa-XIVa**	-2894.517126	-2893.774311	-2893.727215	-2893.853838	0.663288	-2896.756828	-2896.093539	41.2
**XIVa**	-2894.548351	-2893.800752	-2893.753059	-2893.880274	0.668077	-2896.786055	-2896.117979	25.9
**XV**	-3050.445334	-3049.604789	-3049.553569	-3049.688590	0.756744	-3052.847471	-3052.090727	25.0
Figure 39 (ESI)								
**IXc**	-3126.812801	-3125.943433	-3125.891497	-3126.027040	0.785761	-3129.301136	-3128.515375	34.2
**IXc-XIb**	-3126.792706	-3125.925410	-3125.873802	-3126.008444	0.784262	-3129.281094	-3128.496832	45.8
**IXc-XV**	-3126.795397	-3125.929800	-3125.878194	-3126.013302	0.782095	-3129.283919	-3128.501823	42.7
**IXc-XV(thf)**	-3359.066872	-3358.082404	-3358.024660	-3358.175119	0.891753	-3361.807648	-3360.915896	40.5
**IX-Xb**	-2894.534095	-2893.787273	-2893.741592	-2893.864628	0.669467	-2896.771468	-2896.102002	35.9
**Xb**	-2894.552571	-2893.801254	-2893.754809	-2893.880025	0.672546	-2896.790529	-2896.117983	25.9
**XIb**	-3126.826178	-3125.954887	-3125.902907	-3126.038873	0.787305	-3129.314297	-3128.526993	26.9
**XIIb**	-2895.74208	-2894.97244	-2894.92544	-2895.05163	0.690447	-2897.980522	-2897.290074	26.5
**XIIb-XV**	-2895.70289	-2894.9393	-2894.89293	-2895.01609	0.686802	-2897.942088	-2897.255286	47.4
**XIb-XV**	-3126.759289	-3125.894222	-3125.842540	-3125.977625	0.781664	-3129.250204	-3128.468540	63.6
Figure 5								
**XVI**	-2819.360950	-2818.621027	-2818.575669	-2818.697653	0.663297	-2821.514022	-2820.850725	26.6
**XVI-XVII**	-2819.359250	-2818.620448	-2818.575471	-2818.696252	0.662998	-2821.512537	-2820.849538	27.3
**XVII**	-2819.370717	-2818.628213	-2818.583030	-2818.704478	0.666239	-2821.524706	-2820.858467	21.7
**XVIII**	-3051.643214	-3050.780277	-3050.729073	-3050.862471	0.780743	-3054.049031	-3053.268288	22.3
**XVIII-XXIV**	-3051.629367	-3050.766996	-3050.716491	-3050.848366	0.781001	-3054.034649	-3053.253648	31.5
**XXIV**	-3051.659474	-3050.794347	-3050.742536	-3050.878892	0.780582	-3054.063947	-3053.283365	12.8
**XXIV-I'**	-2937.181830	-2936.358698	-2936.308973	-2936.440486	0.741344	-2939.463054	-2938.721710	22.1
**XIX**	-3050.475611	-3049.629833	-3049.579311	-3049.712369	0.763242	-3052.880069	-3052.116827	8.7
**XX**	-3047.139895	-3046.349904	-3046.299957	-3046.431450	0.708445	-3049.545136	-3048.836691	-0.7

**XX-XXI**	-3047.139146	-3046.351866	-3046.302268	-3046.433142	0.706004	-3049.544722	-3048.838719	-2.0
**XXI**	-3047.139664	-3046.350021	-3046.299614	-3046.433274	0.706390	-3049.545365	-3048.838975	-2.2
**XXII**	-3048.317422	-3047.508944	-3047.457974	-3047.591403	0.726019	-3050.724144	-3049.998125	6.6
**XXII-XXIII**	-3048.315166	-3047.509617	-3047.458652	-3047.593185	0.721981	-3050.720939	-3049.998958	6.1
**XXIII**	-3048.320936	-3047.511165	-3047.459891	-3047.595636	0.725300	-3050.726790	-3050.001490	4.5
**H_2**	-1.167208	-1.157176	-1.153871	-1.168693	-0.001485	-1.171647	-1.173133	
**H_2O**	-76.350531	-76.328864	-76.325085	-76.346517	0.004014	-76.443229	-76.439215	
**CO_2**	-188.435335	-188.423309	-188.419749	-188.444004	-0.008669	-188.653625	-188.662294	
**THF**	-232.243090	-232.126405	-232.120623	-232.154325	0.088764	-232.499422	-232.410657	
**MeOH**	-115.613822	-115.562421	-115.558169	-115.585137	0.028685	-115.748665	-115.719980	

Comment on the endergonicity of the net reaction CO_2 + 3 H_2 -> H_3COH + H_2O

The M06-L/def2-TZVP//M06-L/def2-SVP values for the above mentioned net reaction generates a reaction Gibbs free energy of 14.5 kcal/mol, i.e. an endergonic reaction, which is counter intuitive with regard to the fact that the reaction should have a negative Gibbs free reaction energy. However, it should be noted that these computed values are values for gas phase reactions which for the general understanding and implementation of a transition metal complex catalysed reaction mechanism in most cases serve sufficiently well. While the small molecules CO_2, H_2, H_3COH and H_2O can easily be reoptimised in the solvent (see Table S3) this is by no means a fast approach for the reoptimisation of all of the molecules of the rather complex reaction pathway scenario outlined in Figures 3 to 5 of the main text and Figure 39 in the ESI. We have therefore restricted the general discussion to the gas phase energy profiles and refer the reader to the fact that a recomputation in the solvent phase most likely would generate energy profiles which are shifted relative to the ones being presented here, while the relative energy differences between the various intermediates and transition states will most likely not change markedly. However, for completeness we have recalculated the net reaction with the MN12-L density functional using the IEF-PCM and the CPCM continuum model and the IEF-PCM additionally with a radii model recently developed by the Truhlar group.[15] The results demonstrate that the reaction is exergonic in accordance with experimental observation and with standard state thermodynamics (see Table S3). For comparison the gas phase energy values are also included in Table S3.

Table S3. Calculated energies of the optimised structures of the net reaction in the gas phase and in solvent phase with different methods and radii models (Hartree). Relative reaction free energies $G_{rel.}$ are given in kcal/mol.[a]

	E	***E***	***H***	***G***	**A**	***E*(def2-TZVP)**	***E*(def2-TZVP)+A**	**$G_{rel.}$(I')**
M06-L/def2-TZVP// M06-L/def2-SVP (gas)								14.1
H_2	-1.167208	-1.157176	-1.153871	-1.168693	-0.001485	-1.171647	-1.173133	
H_2O	-76.350531	-76.328864	-76.325085	-76.346517	0.004014	-76.443229	-76.439215	
MeOH	-115.613822	-115.562421	-115.558169	-115.585137	0.028685	-115.748665	-115.719980	
CO_2	-188.435335	-188.423309	-188.419749	-188.444004	-0.008669	-188.653625	-188.662294	
M06-L/def2-TZVP (gas)								
H_2	-1.171714	-1.161827	-1.158522	-1.173318				14.2
H_2O	-76.443285	-76.421912	-76.418133	-76.439553				
MeOH	-115.748941	-115.697536	-115.693261	-115.720276				
CO_2	-188.653662	-188.641746	-188.638197	-188.662431				
MN12L/def2-TZVP (THF) (ief-pcm)								
H_2	-1.156961	-1.146909	-1.143605	-1.153010				-1.9
H_2O	-76.385907	-76.364068	-76.360289	-76.376305				
MeOH	-115.642896	-115.590992	-115.586714	-115.608342				
CO_2	-188.519245	-188.507280	-188.503729	-188.522560				
MN12L/def2-TZVP (THF) (ief-pcm, smd)								
H_2	-1.156351	-1.146326	-1.143022	-1.152429				-4.1
H_2O	-76.386444	-76.364749	-76.360970	-76.376990				
MeOH	-115.643021	-115.591364	-115.587067	-115.608730				
CO_2	-188.518526	-188.506598	-188.503033	-188.521883				
MN12L/def2-TZVP (THF) (cpcm)								
H_2	-1.156971	-1.146920	-1.143615	-1.153020				-2.2
H_2O	-76.386313	-76.364474	-76.360695	-76.376712				
MeOH	-115.643189	-115.591295	-115.587016	-115.608646				
CO_2	-188.519399	-188.507437	-188.503886	-188.522717				

[a] According to tabulated values for standard state thermodynamics the net reaction of CO_2 +3 H_2 -> CH_3OH + H_2O is exergonic by -2.1 kcal/mol.

10 X-Ray crystal structure of 4a

Intensity data were collected with a Bruker D8 goniometer equipped with a Bruker APEX CCD area detector and an Incoatec microsource (Mo-K_{α} radiation, λ = 0.71073 Å, multilayer optics) at 100 K (Oxford Cryostream 700 instrument).

Crystal data for **4a**, $C_{43}H_{42}O_3P_3Ru^+C_2F_6NO_4S_2^- \cdot CH_2Cl_2$: monoclinic space group $P2_1$, a = 12.731(2), b = 15.301(3), c = 13.247(2) Å, β = 109.038(3)°, V = 109.038(3) Å^3, Z = 2, 26352 reflections collected within θ_{max} = 25.14 °, R_{int} = 0.0998.

Data were integrated with SAINT[16] and corrected for absorption by multi-scan methods.[17] The structure was solved by direct methods (SHELXS-97) and refined by full matrix least squares procedures based on F^2 as implemented in SHELXL-97.[18] One of the trifluoromethylsulfonate groups in the anion was disordered over two positions, with a site occupancy of 0.599(5) for the majority conformer. A total of 24 similarity restraints for bond distances and angles in the alternative conformations were applied. C, F and O atoms in the minority conformer were treated as isotropic. Anisotropic displacement parameters were assigned to all other non-hydrogen atoms, and hydrogen atoms were treated as riding. Refinement of 647 variables based on 8701 independent data converged for $wR2$ (all reflections) = 0.1212, $R1$ (observed reflections) = 0.0571, GOF = 1.045 and a Flack enantiomorph polarity parameter[19] of -0.05(4).

11 Literature

(1) S. Wesselbaum, T. vom Stein, J. Klankermayer, W. Leitner, *Angew. Chem.* **2012**, *124*, 7617-7620; *Angew. Chem. Int. Ed.* **2012**, *51*, 7499-7502.
(2) A. B. Chaplin, P. J. Dyson, *Inorg. Chem.* **2008**, *47*, 381-390.
(3) F. M. A. Geilen, B. Engendahl, M. Hölscher, J. Klankermayer, W. Leitner, *J. Am. Chem. Soc.* **2011**, *133*, 14349-14358.
(4) J. Coetzee, D. L. Dodds, J. Klankermayer, S. Bronsinski, W. Leitner, A. M. Z. Slawin, D. J. Cole-Hamilton, *Chem. Eur. J.* **2013**, *19*, 11039-11050.
(5) P. Scherl, A. Kruckenberg, S. Mader, H. Wadepohl, L. H. Gade, *Organometallics* **2012**, *31*, 7024-7027.
(6) L. F. Rhodes, C. Sorato, L. M. Venanzi, F. Bachechi, *Inorg. Chem.* **1988**, *27*, 604-610.
(7) Gaussian 09, Revision C.01, M. J. Frisch, G. W. Trucks, H. B. Schlegel, G. E. Scuseria, M. A. Robb, J. R. Cheeseman, G. Scalmani, V. Barone, B. Mennucci, G. A. Petersson, H. Nakatsuji, M. Caricato, X. Li, H. P. Hratchian, A. F. Izmaylov, J. Bloino, G. Zheng, J. L. Sonnenberg, M. Hada, M. Ehara, K. Toyota, R. Fukuda, J. Hasegawa, M. Ishida, T. Nakajima, Y. Honda, O. Kitao, H. Nakai, T. Vreven, J. A. Montgomery, Jr., J. E. Peralta, F. Ogliaro, M. Bearpark, J. J. Heyd, E. Brothers, K. N. Kudin, V. N. Staroverov, T. Keith, R. Kobayashi, J. Normand, K. Raghavachari, A. Rendell, J. C. Burant, S. S. Iyengar, J. Tomasi, M. Cossi, N. Rega, J. M. Millam, M. Klene, J. E. Knox, J. B. Cross, V. Bakken, C. Adamo, J. Jaramillo, R. Gomperts, R. E. Stratmann, O. Yazyev, A. J. Austin, R. Cammi, C. Pomelli, J. W. Ochterski, R. L. Martin, K. Morokuma, V. G. Zakrzewski, G. A. Voth, P. Salvador, J. J. Dannenberg, S. Dapprich, A. D. Daniels, O. Farkas, J. B. Foresman, J. V. Ortiz, J. Cioslowski, and D. J. Fox, Gaussian, Inc., Wallingford CT, **2010**.
(8) a) Y. Zhao, D. G. Truhlar, *J. Chem. Phys*. **2006**, *125*, 194101; b) Y. Zhao, D. G. Truhlar, *Theor. Chem.*

Acc. **2008**, *120*, 215–241; c) C. J. Cramer, D. G. Truhlar, *Phys. Chem. Chem. Phys*. **2009**, 11, 10757 – 10816; d) B. B. Averkiev, Y. Zhao, D. G. Truhlar, *J. Mol. Catal. A* **2010**, *324*, 80– 88; e) A. D. Kulkarni, D. G. Truhlar, *J. Chem. Theory Comput*. **2011**, *7*, 2325 –2332.
(9) a) A. Schäfer, H. Horn, R. Ahlrichs, J. Chem. Phys. **1992**, 97, 2571; b) A. Schäfer, C. Huber, R. Ahlrichs, J. Chem. Phys. **1994**, 100, 5829; c) K. Eichkorn, F. Weigend, O. Treutler, R. Ahlrichs, *Theor. Chem. Acc*. **1997**, *97*, 119 –124; d) F. Weigend, F. Furche, R. Ahlrichs, *J. Chem. Phys*. 2003, *119*, 12753; e) F. Weigend, R. Ahlrichs, *Phys. Chem. Chem. Phys*. **2005**, *7*, 3297 –3305.
(10) a) D. Andrae, U. Häußermann, M. Dolg, H. Stoll, H. Preuß, *Theor. Chim. Acta* **1990**, *77*, 123–141; b) B. Metz, H. Stoll, M. Dolg, *J. Chem. Phys*. **2000**, *113*, 2563.
(11) a) B. I. Dunlap, *J. Chem. Phys*. **1983**, *78*, 3140; b) B. Dunlap, *J. Mol. Struct*. **2000**, *529*, 37–40.
(12) R. Peverati, D. G. Truhlar, *Phys Chem Chem Phys* **2012**, *10*, 13171.
(13) a) J. Tomasi, B. Mennucci, R. Cammi, *Chem. Rev*. **2005**, *105*, 2999–3093; b) E. Cances, B. Mennucci, J. Tomasi, *J. Chem. Phys*. **1997**, *107*, 3032.
(14) a) R. L. Martin, P. J. Hay, L. R. Pratt, *J. Phys. Chem*. A **1998**, *102*, 3565–3573; b) N. Sieffert, M. Bühl, *Inorg. Chem*. **2009**, *48*, 4622 – 4624
(15) A. V. Marenich, C. J Cramer, D. G. Truhlar, *J. Phys. Chem. B.* **2009**, *113*, 6378-6396.
(16) Bruker (2009) SAINT+. Version 7.68. Bruker AXS Inc., Madison, Wisconsin, USA.
(17) Bruker (2001) SMART. Version 5.624. Bruker AXS Inc., Madison Wisconsin, USA.
(18) G. M. Sheldrick, Acta Crystallogr. A64 (2008) 112-122.
(19) H. D. Flack, Acta Crystallogr. A39 (1983) 876-881.

12 Appendix: XYZ Coordinates of the calculated structures (Gas)

I

```
H       4.217747    4.885684   -1.803856
H       4.537885    3.013760   -3.415754
C       3.752477    3.916132   -1.614040
C       3.929367    2.867929   -2.520791
H       2.847803    4.529731    0.250201
C       2.990428    3.717530   -0.466469
C       3.340806    1.629484   -2.282398
H       3.517101    0.811887   -2.987672
H       6.571800   -1.394723   -0.276667
H       6.179396   -3.835541   -0.562117
H       0.067271    7.031497    0.877746
H      -0.041797    6.399863   -1.526707
C       2.390181    2.479503   -0.231489
C       5.564897   -1.768680   -0.473277
C       5.344891   -3.134982   -0.633085
C       2.551923    1.427081   -1.137192
H       4.695331    0.198379   -0.432401
C      -0.120954    5.994255    0.592926
C       4.502618   -0.869408   -0.562929
C      -0.181827    5.640560   -0.754384
H       1.780812    2.334734    0.662520
C       4.053567   -3.605932   -0.870680
H       3.866739   -4.676634   -0.978076
H      -0.265534    5.295228    2.633378
C      -0.306550    5.021551    1.576698
C       3.205973   -1.334191   -0.818885
H       1.729459   -0.294423   -3.257679
C       2.990740   -2.713407   -0.952580
C      -0.423543    4.318110   -1.123948
H      -0.467714    4.062672   -2.185713
H       0.430981    1.945355   -2.471726
H       1.978879   -3.104151   -1.089392
H       0.172423    0.662131   -4.806841
P       1.756127   -0.209146   -0.856733
C      -0.546478    3.699905    1.210658
C       1.006524   -0.621680   -2.494125
C      -0.602801    3.337653   -0.141095
H      -0.686130    2.930727    1.976732
H       0.991440   -1.720561   -2.563047
H      -0.348584   -1.033864   -4.767361
C      -0.521783    1.413702   -2.321929
C      -0.537354   -0.037287   -4.341958
H      -1.250576    1.955679   -2.940792
C      -0.390138   -0.046567   -2.818892
H      -1.551251    0.265466   -4.642640
P      -0.967800    1.593044   -0.545904
Ru     -0.018549   -0.097952    0.752111
H      -3.107517    1.895570   -2.669156
H      -0.221117   -5.806496    1.555674
H      -1.035669   -3.488538    1.541550
C      -0.269755   -5.261720    0.611066
C      -0.721416   -3.946649    0.599748
C      -1.526929   -0.928301   -2.251300
C      -3.580460    1.813167   -1.688610
H      -1.670027   -1.815306   -2.885004
C      -2.819353    1.634116   -0.523602
H      -1.281034    0.433349    1.641141
H       0.475398   -6.910237   -0.570525
C       0.119137   -5.878176   -0.578284
H      -2.473456   -0.372966   -2.321317
P      -1.347002   -1.466112   -0.495438
C      -0.786343   -3.216140   -0.596176
H      -5.541068    2.011999   -2.554823
C      -4.972086    1.881693   -1.631882
C       0.036417   -5.174946   -1.777484
```

C	-0.413655	-3.854167	-1.788314
C	-3.496590	1.559985	0.701871
H	0.320456	-5.653818	-2.716991
H	-2.934403	1.420853	1.628702
H	-0.467308	-3.338104	-2.749454
C	-5.630068	1.789961	-0.408308
C	-3.056687	-1.647243	0.135110
C	-4.885050	1.638107	0.759682
H	-2.419686	-1.566520	2.205540
H	-4.023835	-1.774073	-1.808352
H	-6.720027	1.839996	-0.364329
C	-3.264868	-1.691849	1.521249
C	-4.154792	-1.786040	-0.723353
H	-5.387066	1.568656	1.727130
C	-4.545343	-1.872228	2.037355
C	-5.437896	-1.947973	-0.202694
H	-4.692086	-1.911114	3.118779
H	-6.287154	-2.045417	-0.882513
C	-5.635733	-1.992825	1.176197
H	-6.641389	-2.125825	1.580649
H	1.265425	-1.326593	0.653408
C	2.706670	0.646392	2.562498
C	1.789760	0.933211	4.700952
C	3.023862	1.291118	3.887214
H	3.219987	1.092618	1.699324
H	2.912707	-0.437064	2.564340
H	1.646234	1.563518	5.587491
H	1.845301	-0.112614	5.036706
H	3.122730	2.382548	3.772690
H	3.958382	0.915836	4.322676
O	1.280589	0.867115	2.392885
C	0.669788	1.089028	3.695126
H	-0.145531	0.363356	3.829678
H	0.239935	2.104733	3.690715
H	-0.096993	-1.446986	2.090987
C	0.872192	-2.000014	2.585751
O	0.761218	-2.143319	3.776353
O	1.743070	-2.303770	1.732972

I-VIII

H	4.217747	4.885684	-1.803856
H	4.537885	3.013760	-3.415754
C	3.752477	3.916132	-1.614040
C	3.929367	2.867929	-2.520791
H	2.847803	4.529731	0.250201
C	2.990428	3.717530	-0.466469
C	3.340806	1.629484	-2.282398
H	3.517101	0.811887	-2.987672
H	6.571800	-1.394723	-0.276667
H	6.179396	-3.835541	-0.562117
H	0.067271	7.031497	0.877746
H	-0.041797	6.399863	-1.526707
C	2.390181	2.479503	-0.231489
C	5.564897	-1.768680	-0.473277
C	5.344891	-3.134982	-0.633085
C	2.551923	1.427081	-1.137192
H	4.695331	0.198379	-0.432401
C	-0.120954	5.994255	0.592926
C	4.502618	-0.869408	-0.562929
C	-0.181827	5.640560	-0.754384
H	1.780812	2.334734	0.662520
C	4.053567	-3.605932	-0.870680
H	3.866739	-4.676634	-0.978076
H	-0.265534	5.295228	2.633378
C	-0.306550	5.021551	1.576698
C	3.205973	-1.334191	-0.818885
H	1.729459	-0.294423	-3.257679
C	2.990740	-2.713407	-0.952580
C	-0.423543	4.318110	-1.123948
H	-0.467714	4.062672	-2.185713
H	0.430981	1.945355	-2.471726
H	1.978879	-3.104151	-1.089392
H	0.172423	0.662131	-4.806841
P	1.756127	-0.209146	-0.856733
C	-0.546478	3.699905	1.210658
C	1.006524	-0.621680	-2.494125
C	-0.602801	3.337653	-0.141095
H	-0.686130	2.930727	1.976732
H	0.991440	-1.720561	-2.563047
H	-0.348584	-1.033864	-4.767361
C	-0.521783	1.413702	-2.321929
C	-0.537354	-0.037287	-4.341958
H	-1.250576	1.955679	-2.940792
C	-0.390138	-0.046567	-2.818892
H	-1.551251	0.265466	-4.642640
P	-0.967800	1.593044	-0.545904
Ru	-0.018549	-0.097952	0.752111
H	-3.107517	1.895570	-2.669156
H	-0.221117	-5.806496	1.555674
H	-1.035669	-3.488538	1.541550
C	-0.269755	-5.261720	0.611066
C	-0.721416	-3.946649	0.599748
C	-1.526929	-0.928301	-2.251300
C	-3.580460	1.813167	-1.688610
H	-1.670027	-1.815306	-2.885004
C	-2.819353	1.634116	-0.523602
H	-1.281034	0.433349	1.641141
H	0.475398	-6.910237	-0.570525
C	0.119137	-5.878176	-0.578284
H	-2.473456	-0.372966	-2.321317
P	-1.347002	-1.466112	-0.495438
C	-0.786343	-3.216140	-0.596176
H	-5.541068	2.011999	-2.554823
C	-4.972086	1.881693	-1.631882
C	0.036417	-5.174946	-1.777484
C	-0.413655	-3.854167	-1.788314
C	-3.496590	1.559985	0.701871
H	0.320456	-5.653818	-2.716991
H	-2.934403	1.420853	1.628702
H	-0.467308	-3.338104	-2.749454
C	-5.630068	1.789961	-0.408308

C	-3.056687	-1.647243	0.135110
C	-4.885050	1.638107	0.759682
H	-2.419686	-1.566520	2.205540
H	-4.023835	-1.774073	-1.808352
H	-6.720027	1.839996	-0.364329
C	-3.264868	-1.691849	1.521249
C	-4.154792	-1.786040	-0.723353
H	-5.387066	1.568656	1.727130
C	-4.545343	-1.872228	2.037355
C	-5.437896	-1.947973	-0.202694
H	-4.692086	-1.911114	3.118779
H	-6.287154	-2.045417	-0.882513
C	-5.635733	-1.992825	1.176197
H	-6.641389	-2.125825	1.580649
H	1.265425	-1.326593	0.653408
C	2.706670	0.646392	2.562498
C	1.789760	0.933211	4.700952
C	3.023862	1.291118	3.887214
H	3.219987	1.092618	1.699324
H	2.912707	-0.437064	2.564340
H	1.646234	1.563518	5.587491
H	1.845301	-0.112614	5.036706
H	3.122730	2.382548	3.772690
H	3.958382	0.915836	4.322676
O	1.280589	0.867115	2.392885
C	0.669788	1.089028	3.695126
H	-0.145531	0.363356	3.829678
H	0.239935	2.104733	3.690715
H	-0.096993	-1.446986	2.090987
C	0.872192	-2.000014	2.585751
O	0.761218	-2.143319	3.776353
O	1.743070	-2.303770	1.732972

I-VIII'

H	-5.500702	-3.667950	-1.725949
H	-5.066587	-2.050069	-3.569487
C	-4.759797	-2.876347	-1.595648
C	-4.514156	-1.972390	-2.630675
H	-4.241996	-3.464621	0.417368
C	-4.059677	-2.760540	-0.397777
C	-3.570035	-0.961550	-2.468338
H	-3.415309	-0.252455	-3.286197
H	-5.789285	3.194244	-0.848345
H	-4.615197	5.258216	-0.101474
H	-2.149713	-6.535027	1.424251
H	-1.903206	-6.164490	-1.024134
C	-3.108256	-1.753087	-0.236546
C	-4.704012	3.193216	-0.726636
C	-4.045919	4.350213	-0.310787
C	-2.848804	-0.841377	-1.268356
H	-4.524601	1.123976	-1.281591
C	-1.647298	-5.638769	1.054498
C	-3.986377	2.025189	-0.979358
C	-1.509134	-5.431319	-0.317097
H	-2.547446	-1.685455	0.698001
C	-2.661425	4.338939	-0.149040
H	-2.134428	5.229354	0.199864
H	-1.232677	-4.864298	3.030170
C	-1.135232	-4.702474	1.954152
C	-2.591712	2.007488	-0.828214
H	-1.542634	0.435160	-3.459249
C	-1.940182	3.175872	-0.403732
C	-0.868312	-4.289102	-0.795421
H	-0.774535	-4.148400	-1.874924
H	-0.859397	-2.010741	-2.395510
H	-0.859691	3.178126	-0.246567
H	-0.257385	-1.076714	-4.816651
P	-1.595412	0.483779	-1.025929
C	-0.494500	-3.561746	1.478818
C	-0.790361	0.617282	-2.677800
C	-0.360754	-3.341560	0.101492
H	-0.091736	-2.826214	2.182385
H	-0.491686	1.669358	-2.802212
H	0.672902	0.430896	-4.921018
C	0.187456	-1.707538	-2.238314
C	0.596682	-0.529933	-4.391222
H	0.786065	-2.467985	-2.760283
C	0.424693	-0.319723	-2.886006
H	1.507138	-1.105470	-4.614739
P	0.512003	-1.823126	-0.425011
Ru	-0.001096	0.127610	0.640902
H	2.590751	-2.924397	-2.358502
H	1.634681	5.594407	1.137823
H	1.927840	3.159334	1.254583
C	1.539260	5.000105	0.226995
C	1.697051	3.619622	0.290415
C	1.730733	0.305385	-2.351677
C	3.022722	-2.845112	-1.358486
H	2.099910	1.077748	-3.043002
C	2.273889	-2.353461	-0.279478
H	1.161903	-0.236344	1.938442
H	1.130884	6.698668	-1.045949
C	1.258279	5.615521	-0.993523
H	2.505611	-0.475488	-2.347927
P	1.671412	1.000048	-0.643509
C	1.564722	2.824611	-0.859453
H	4.913626	-3.620094	-2.037614
C	4.347214	-3.244624	-1.182685
C	1.153665	4.842412	-2.147247
C	1.306037	3.456820	-2.083120
C	2.882728	-2.296013	0.982298
H	0.951995	5.317027	-3.109736
H	2.324776	-1.914955	1.841708
H	1.223617	2.884368	-3.009502

C	4.942184	-3.169981	0.074424
C	3.386902	0.806923	-0.008214
C	4.202838	-2.699927	1.158670
H	2.834702	1.278632	2.040766
H	4.308847	0.341069	-1.924754
H	5.980799	-3.479001	0.209659
C	3.645928	1.031139	1.352576
C	4.460622	0.498871	-0.855032
H	4.658145	-2.637492	2.149372
C	4.942078	0.940456	1.852795
C	5.755425	0.395598	-0.347733
H	5.122358	1.124028	2.914044
H	6.577779	0.146475	-1.022055
C	6.000320	0.613629	1.005914
H	7.015990	0.535923	1.399515
H	0.936521	0.508052	2.465731
C	-2.822972	0.559913	2.239972
C	-2.188560	0.058036	4.464519
C	-3.412239	0.384273	3.622022
H	-3.490943	0.241514	1.424281
H	-2.516397	1.601089	2.045494
H	-2.429341	-0.432876	5.415845
H	-1.612324	0.967716	4.692111
H	-4.127805	-0.452532	3.637324
H	-3.947715	1.281404	3.956466
O	-1.637142	-0.285683	2.213594
C	-1.404988	-0.833859	3.534678
H	-0.321919	-0.841406	3.717479
H	-1.776527	-1.874413	3.548409
H	-0.257562	1.896706	1.458880
C	-0.124070	2.287870	2.577023
O	-0.743463	3.292276	2.818117
O	0.656120	1.567948	3.271111

IIa

```
H        3.057147   -5.988933   -0.829991
H        1.786653   -5.341357   -2.872114
C        2.335425   -5.170221   -0.790444
C        1.626770   -4.805573   -1.933880
H        2.652867   -4.773268    1.309118
C        2.108501   -4.489226    0.405005
C        0.702623   -3.761290   -1.887493
H        0.158605   -3.514548   -2.801790
H       -4.940652   -4.282618   -1.274023
H       -5.322508   -4.700081    1.150627
H        6.840225   -2.010183   -0.212287
H        5.014366   -3.240533   -1.384325
C        1.190624   -3.443292    0.450247
C       -4.239787   -3.891560   -0.533262
C       -4.453073   -4.125709    0.823718
C        0.482615   -3.054973   -0.696368
H       -2.982725   -3.003048   -2.025842
C        5.866389   -1.539162   -0.361335
C       -3.130254   -3.159725   -0.954732
C        4.846120   -2.226179   -1.014975
H        1.022906   -2.906985    1.385550
C       -3.547766   -3.628649    1.761520
H       -3.705077   -3.812847    2.826643
H        6.433055    0.314765    0.594952
C        5.637699   -0.238954    0.090908
C       -2.229542   -2.636112   -0.017990
H       -0.965062   -2.015080   -2.934662
C       -2.447348   -2.886682    1.342256
C        3.601931   -1.625437   -1.209840
H        2.823531   -2.196201   -1.720246
H        1.420324   -1.066361   -2.745198
H       -1.757469   -2.465704    2.078984
H       -0.157946   -0.726566   -4.808203
P       -0.737819   -1.681603   -0.517598
C        4.398542    0.363054   -0.107371
C       -1.142532   -1.171722   -2.252598
C        3.357930   -0.320863   -0.759524
H        4.241262    1.384782    0.250751
H       -2.228710   -0.997985   -2.285088
H       -1.718290    0.081466   -4.549955
C        1.103974   -0.027416   -2.555240
C       -0.646174    0.121597   -4.305982
H        1.643932    0.588734   -3.292085
C       -0.419012    0.078751   -2.793657
H       -0.238098    1.044544   -4.742796
P        1.696149    0.468326   -0.865103
Ru      -0.068629    0.027737    0.767970
H        3.715461    1.717056   -2.655972
H       -5.609691   -0.871617    1.509230
H       -3.250081   -0.125646    1.513440
C       -5.190532   -0.403315    0.615638
C       -3.864759    0.024574    0.621841
C       -1.018942    1.379774   -2.189095
C        3.207992    2.521443   -2.114148
H       -1.851985    1.737792   -2.812039
C        2.204054    2.216209   -1.180601
H       -7.007154   -0.594991   -0.538718
C       -5.970629   -0.251165   -0.528660
H       -0.264444    2.181867   -2.226566
P       -1.545473    1.213602   -0.432202
C       -3.295103    0.617329   -0.512844
H        4.364397    4.064797   -3.071742
C        3.580743    3.842662   -2.344128
C       -5.420049    0.345483   -1.661928
C       -4.096836    0.783857   -1.652219
C        1.586984    3.267578   -0.500074
H       -6.024955    0.478108   -2.561541
H        0.771562    3.040820    0.187669
H       -3.702681    1.257463   -2.554375
C        2.961949    4.882247   -1.644823
C       -1.825482    2.923015    0.173670
```

C	1.959048	4.594110	-0.722936
H	-2.390318	2.172779	2.124141
H	-1.331917	3.990139	-1.653378
H	3.263410	5.916512	-1.823406
C	-2.254769	3.065712	1.503629
C	-1.660369	4.069018	-0.613945
H	1.461200	5.395907	-0.171666
C	-2.509099	4.327980	2.033530
C	-1.908840	5.332574	-0.076647
H	-2.845892	4.425960	3.067936
H	-1.775009	6.219280	-0.700443
C	-2.331317	5.465388	1.244694
H	-2.527023	6.455951	1.659992
H	-1.281023	-0.261067	1.829837
C	0.663716	-1.339656	3.628791
C	2.969009	-1.027897	3.959234
C	1.826871	-1.938209	4.383485
H	-0.149914	-2.043395	3.402755
H	0.223362	-0.470912	4.149133
H	3.964759	-1.462245	4.113435
H	2.933273	-0.084288	4.525929
H	2.008958	-2.972663	4.050013
H	1.663233	-1.966786	5.468008
O	1.231598	-0.901668	2.379640
C	2.662902	-0.780420	2.496797
H	2.958275	0.219285	2.144992
H	3.128985	-1.525399	1.826747
C	0.663197	2.962468	2.576166
O	0.586683	1.816934	2.358094
O	0.762133	4.092173	2.805400

II

H	-3.588026	-5.669312	0.719009
H	-2.863755	-4.506355	2.798809
C	-2.811179	-4.903283	0.677595
C	-2.408080	-4.250159	1.839712
H	-2.500851	-5.104320	-1.451915
C	-2.203928	-4.585524	-0.537827
C	-1.416506	-3.268888	1.789711
H	-1.124046	-2.779646	2.721995
H	4.508332	-4.442718	1.430694
H	4.933680	-5.042530	-0.948205
H	-6.884909	-1.203596	-0.912785
H	-5.538044	-2.364366	0.833371
C	-1.210388	-3.611815	-0.586620
C	3.857541	-4.057272	0.642908
C	4.094810	-4.393342	-0.688437
C	-0.812541	-2.926933	0.573164
H	2.610996	-3.000983	2.032651
C	-5.902003	-0.820230	-0.632164
C	2.784287	-3.233088	0.979096
C	-5.148796	-1.469166	0.342952
H	-0.713941	-3.395319	-1.538030
C	3.254687	-3.900392	-1.686751
H	3.432838	-4.161250	-2.732280
H	-5.975992	0.838097	-2.016210
C	-5.392032	0.321991	-1.251152
C	1.949855	-2.715806	-0.020127
H	0.576445	-1.813327	2.817611
C	2.194516	-3.060960	-1.355847
C	-3.892909	-0.980291	0.706055
H	-3.327128	-1.510061	1.474472
H	-1.741392	-0.608988	2.387815
H	1.564569	-2.642465	-2.146547
H	-0.219489	-0.293378	4.531551
P	0.505553	-1.656682	0.378205
C	-4.139112	0.811731	-0.893104
C	0.853725	-1.041461	2.084266
C	-3.375106	0.171185	0.099198
H	-3.754395	1.714307	-1.379478
H	1.948670	-0.953639	2.162022
H	1.430237	0.322573	4.306311
C	-1.287611	0.368933	2.164655
C	0.382947	0.456045	3.997210
H	-1.779382	1.087495	2.839297
C	0.227534	0.314239	2.481554
H	0.056657	1.448492	4.339860
P	-1.697881	0.834807	0.422548
Ru	0.001303	0.087451	-1.084465
H	-3.532097	2.374513	2.127528
H	5.443160	-1.250361	-1.718265
H	3.169386	-0.278578	-1.802055
C	5.042457	-0.739199	-0.840076
C	3.766633	-0.182939	-0.891078
C	0.986216	1.493765	1.817074
C	-2.995716	3.089551	1.495369
H	1.845730	1.780410	2.440569
C	-2.050068	2.634926	0.561604
H	-0.533150	1.454536	-2.082632
H	6.794126	-1.099941	0.372124
C	5.797368	-0.655914	0.328227
H	0.334022	2.382483	1.799734
P	1.547877	1.227409	0.078843
C	3.224461	0.470848	0.223570
H	-4.018811	4.787337	2.337468
C	-3.277732	4.447182	1.611088
C	5.274412	0.000523	1.441056
C	4.001941	0.566996	1.387830
C	-1.408405	3.568169	-0.258333
H	5.861496	0.079643	2.358496
H	-0.677297	3.234909	-0.999530
H	3.629681	1.084312	2.275139
C	-2.620652	5.371376	0.795427

C	1.950502	2.907810	-0.528327
C	-1.688573	4.930602	-0.140303
H	2.149604	2.213538	-2.570998
H	1.863681	3.920290	1.394590
H	-2.843291	6.436433	0.888095
C	2.199460	3.075635	-1.898230
C	2.040024	4.018347	0.321103
H	-1.173763	5.645029	-0.786713
C	2.513082	4.331480	-2.410847
C	2.352881	5.275099	-0.195978
H	2.704783	4.448617	-3.479430
H	2.415199	6.133970	0.475688
C	2.584038	5.435798	-1.560685
H	2.827139	6.421487	-1.962697
H	1.151001	-0.525846	-2.082692
O	-1.632690	-1.043651	-2.370634
H	0.127800	1.075443	-2.517386
C	-2.634020	-1.621853	-2.537058
O	-3.608328	-2.210112	-2.741794

IIa-VI

H	0.555521	-6.615425	-1.793231
H	-1.772900	-5.945722	-2.365539
C	0.209825	-5.602103	-1.578389
C	-1.095890	-5.226536	-1.899775
H	2.091460	-4.964742	-0.722642
C	1.067009	-4.681014	-0.979633
C	-1.544713	-3.937908	-1.621528
H	-2.575441	-3.663737	-1.865291
H	-6.203844	-1.712012	-1.373404
H	-6.653074	-2.365234	0.988670
H	6.540211	-3.004186	-0.431230
H	4.808006	-3.660359	-2.095938
C	0.618424	-3.389478	-0.703361
C	-5.381965	-1.785293	-0.657672
C	-5.633398	-2.149987	0.662433
C	-0.687150	-3.002852	-1.021510
H	-3.921879	-1.242243	-2.124536
C	5.627614	-2.413541	-0.534329
C	-4.080239	-1.510665	-1.078040
C	4.656970	-2.782348	-1.463643
H	1.284151	-2.670372	-0.223608
C	-4.572827	-2.241795	1.563293
H	-4.756827	-2.530976	2.600296
H	6.197288	-0.972348	0.972993
C	5.435006	-1.276825	0.252625
C	-3.007806	-1.585221	-0.178683
H	-1.468329	-1.386211	-3.097668
C	-3.275580	-1.958368	1.146232
C	3.491226	-2.027740	-1.602078
H	2.748614	-2.339932	-2.339993
H	1.045884	-1.236378	-2.714542
H	-2.458980	-2.013437	1.868880
H	-0.256902	-0.229170	-4.875192
P	-1.257432	-1.284960	-0.673514
C	4.274477	-0.522122	0.115358
C	-1.420357	-0.554815	-2.375928
C	3.282985	-0.892540	-0.807880
H	4.134544	0.370800	0.733589
H	-2.391113	-0.040177	-2.430202
H	-1.496483	1.000884	-4.557871
C	1.084649	-0.146150	-2.555019
C	-0.474777	0.683427	-4.301849
H	1.793225	0.237581	-3.304625
C	-0.326273	0.443358	-2.798435
H	0.215090	1.467320	-4.647386
P	1.769046	0.146638	-0.872213
Ru	0.034520	-0.056216	0.730317
H	3.378217	1.453557	-2.995981
H	-5.322711	1.269123	2.006745
H	-2.866997	1.186619	1.834788
C	-4.842541	1.496872	1.052495
C	-3.455037	1.455778	0.953986
C	-0.521396	1.803773	-2.084039
C	3.288621	2.153821	-2.160799
H	-1.277567	2.397183	-2.617181
C	2.571850	1.803259	-1.004584
H	-0.389231	0.257139	3.631474
H	-6.704616	1.843658	0.011557
C	-5.615356	1.817754	-0.062704
H	0.405586	2.392428	-2.157888
P	-0.959664	1.712386	-0.293104
C	-2.808575	1.736022	-0.258537
H	4.469157	3.650566	-3.160906
C	3.918881	3.391054	-2.253917
C	-4.987985	2.107511	-1.271644
C	-3.596846	2.073195	-1.367920
C	2.534797	2.703974	0.065454
H	-5.581647	2.368702	-2.150408
H	2.005026	2.441739	0.981635
H	-3.144030	2.314687	-2.331860
C	3.861496	4.289987	-1.186604

C	-0.587485	3.382664	0.355970
C	3.179762	3.938845	-0.025305
H	-0.813031	2.717859	2.402340
H	-0.313694	4.369947	-1.562191
H	4.360163	5.258766	-1.260719
C	-0.605050	3.567083	1.745501
C	-0.327951	4.479549	-0.475176
H	3.138227	4.629108	0.820353
C	-0.353368	4.821301	2.294763
C	-0.076255	5.734206	0.077545
H	-0.367844	4.952401	3.378746
H	0.128062	6.582016	-0.579878
C	-0.084308	5.907107	1.460586
H	0.114251	6.891129	1.890383
H	-1.277138	-0.167564	1.843325
C	0.051451	-2.786834	2.591966
C	2.233672	-2.948678	3.501072
C	0.995431	-3.788209	3.226329
H	-0.592034	-3.224835	1.812020
H	-0.592478	-2.281688	3.334136
H	3.146751	-3.542175	3.633939
H	2.102106	-2.331106	4.402868
H	1.229393	-4.595143	2.514449
H	0.569211	-4.256712	4.122086
O	0.897392	-1.783899	1.988087
C	2.282413	-2.074231	2.272648
H	2.810298	-1.119252	2.401514
H	2.730002	-2.601493	1.408374
H	-0.823075	0.036181	2.849387
O	1.200988	0.952981	2.352916
C	1.044480	0.655253	3.527073
O	1.543598	0.521233	4.589719

II-III

H	-3.173858	5.976014	1.054657
H	-4.215909	4.255956	2.522673
C	-2.909031	4.923454	0.934341
C	-3.491176	3.959671	1.761526
H	-1.555161	5.283361	-0.711948
C	-2.003793	4.538455	-0.050912
C	-3.155805	2.617764	1.613179
H	-3.636875	1.871074	2.252900
H	-5.527467	-2.821148	1.056543
H	-6.847578	-1.860124	-0.824059
H	2.189023	6.717537	-1.257883
H	1.705596	4.969399	-2.965589
C	-1.666703	3.192693	-0.203636
C	-5.145383	-1.963262	0.498738
C	-5.882498	-1.426622	-0.554006
C	-2.225425	2.224863	0.636781
H	-3.344628	-1.879661	1.656672
C	2.035694	5.680683	-0.951527
C	-3.909225	-1.417220	0.843734
C	1.766526	4.702249	-1.908638
H	-0.974811	2.895666	-0.991758
C	-5.386934	-0.329313	-1.260043
H	-5.959315	0.100084	-2.084377
H	2.324606	6.097480	1.148195
C	2.110623	5.334036	0.397362
C	-3.405172	-0.315400	0.142206
H	-1.928262	0.593164	2.873925
C	-4.160896	0.226170	-0.910203
C	1.566072	3.380553	-1.516492
H	1.327441	2.620583	-2.267547
H	0.102193	2.148433	2.109193
H	-3.794082	1.102464	-1.454565
H	-0.155787	0.931191	4.506745
P	-1.780927	0.454922	0.468844
C	1.917846	4.011790	0.793026
C	-1.348250	-0.045773	2.189646
C	1.637787	3.026248	-0.162250
H	1.989866	3.757467	1.853827
H	-1.707609	-1.073225	2.356342
H	-0.293211	-0.838273	4.517009
C	0.817327	1.311357	2.038587
C	0.256512	0.006580	4.077888
H	1.649440	1.590607	2.701766
C	0.148739	0.009412	2.551719
H	1.304294	-0.073817	4.403196
P	1.401570	1.273038	0.294411
Ru	-0.057169	-0.007321	-1.055372
H	3.395240	0.719261	2.533156
H	-3.376140	-4.444626	-1.217991
H	-1.836934	-2.518101	-1.319577
C	-2.582608	-4.352211	-0.473320
C	-1.708631	-3.266950	-0.532896
C	0.867427	-1.254817	2.032964
C	3.860719	0.515331	1.565662
H	0.609768	-2.103243	2.684512
C	3.144791	0.674285	0.371680
H	0.920262	-0.706729	-2.359403
H	-3.139319	-6.147716	0.588528
C	-2.452143	-5.300523	0.537166
H	1.951363	-1.111614	2.139898
P	0.529359	-1.746514	0.280877
C	-0.687511	-3.120972	0.412280
H	5.728175	-0.032128	2.490444
C	5.189163	0.088174	1.548169
C	-1.430619	-5.171760	1.480520
C	-0.548176	-4.097615	1.413172
C	3.804526	0.416906	-0.838811
H	-1.311013	-5.921020	2.265831
H	3.284040	0.558415	-1.791190
H	0.269474	-4.042306	2.137727
C	5.826750	-0.174347	0.338243

C	2.040672	-2.631613	-0.261952
C	5.131107	-0.001977	-0.858349
H	1.150709	-3.017117	-2.201929
H	3.210499	-2.470321	1.567094
H	6.866710	-0.507134	0.326075
C	2.037580	-3.140267	-1.570703
C	3.170993	-2.834267	0.538542
H	5.623002	-0.196301	-1.813676
C	3.145545	-3.814173	-2.073846
C	4.281492	-3.509397	0.030911
H	3.125194	-4.205673	-3.092935
H	5.158206	-3.652896	0.666532
C	4.275342	-3.994426	-1.274254
H	5.146774	-4.521525	-1.667969
H	-1.179109	-0.832322	-2.044844
O	-0.913451	1.317285	-2.707257
H	1.400052	-0.054148	-2.073780
C	-1.573894	0.342178	-3.021607
O	-2.382251	-0.151815	-3.723441

III

H	-4.259082	5.227308	1.113187
H	-4.749329	3.410612	2.744277
C	-3.795556	4.245942	0.992519
C	-4.067292	3.229408	1.911010
H	-2.746429	4.783499	-0.818372
C	-2.948407	4.000058	-0.084660
C	-3.478228	1.977884	1.761935
H	-3.725623	1.186126	2.475273
H	-4.916173	-3.835209	0.996222
H	-6.305340	-3.154331	-0.954393
H	0.567263	6.965946	-1.323032
H	0.439002	5.135152	-3.007664
C	-2.353919	2.746777	-0.237965
C	-4.675593	-2.925577	0.441458
C	-5.452426	-2.544065	-0.650475
C	-2.598932	1.732448	0.694222
H	-2.972962	-2.494802	1.672962
C	0.658854	5.924537	-1.007555
C	-3.578999	-2.156154	0.829217
C	0.589246	4.900385	-1.951979
H	-1.713820	2.551296	-1.098713
C	-5.141462	-1.378830	-1.352664
H	-5.744390	-1.072519	-2.209332
H	0.904120	6.419276	1.081115
C	0.846464	5.619058	0.340433
C	-3.262131	-0.984704	0.130143
H	-1.998728	0.091341	2.911219
C	-4.058617	-0.599602	-0.960535
C	0.701110	3.571325	-1.549429
H	0.610425	2.770089	-2.289578
H	-0.345731	2.099989	2.123461
H	-3.836102	0.323300	-1.505422
H	-0.352568	0.875461	4.512637
P	-1.829755	0.081497	0.502102
C	0.966722	4.292001	0.747431
C	-1.292289	-0.368125	2.203264
C	0.888619	3.259014	-0.196086
H	1.124852	4.071816	1.806562
H	-1.406090	-1.456468	2.327906
H	-0.095238	-0.880450	4.544044
C	0.525326	1.428925	2.038994
C	0.255852	0.060030	4.095811
H	1.295969	1.879715	2.681284
C	0.156014	0.020252	2.570013
H	1.294047	0.216876	4.423842
P	1.075619	1.508787	0.284807
Ru	-0.053118	-0.025352	-1.032161
H	3.173257	1.387370	2.489166
H	-2.407670	-4.986165	-1.172032
H	-1.280217	-2.796473	-1.267713
C	-1.629290	-4.752441	-0.442589
C	-0.986216	-3.515672	-0.497570
C	1.138761	-1.056852	2.065038
C	3.657728	1.302521	1.513476
H	1.064996	-1.943531	2.712822
C	2.908124	1.315227	0.329254
H	1.143011	-0.511299	-2.273544
H	-1.793733	-6.640127	0.590843
C	-1.287926	-5.673486	0.543645
H	2.164750	-0.681574	2.181651
P	0.921165	-1.596113	0.309688
C	0.010382	-3.188893	0.428449
H	5.611764	1.170885	2.410664
C	5.047034	1.179652	1.475851
C	-0.287328	-5.363042	1.467227
C	0.364406	-4.135268	1.404829
C	3.592491	1.228520	-0.892260
H	-0.003285	-6.088001	2.232799
H	3.043565	1.271991	-1.837722
H	1.172250	-3.929635	2.113366
C	5.710261	1.077042	0.255787

C	2.583724	-2.145081	-0.232530
C	4.977979	1.108542	-0.930937
H	1.793821	-2.715528	-2.171350
H	3.693106	-1.737211	1.595268
H	6.797578	0.980719	0.227799
C	2.687309	-2.644319	-1.540852
C	3.731834	-2.101473	0.566772
H	5.488013	1.041699	-1.894067
C	3.912724	-3.067716	-2.045018
C	4.960223	-2.524668	0.057946
H	3.974929	-3.456209	-3.063509
H	5.848289	-2.477597	0.692237
C	5.055841	-3.001697	-1.246649
H	6.019017	-3.331612	-1.641108
H	-1.059230	-1.023013	-2.197224
O	-1.196057	1.003908	-2.647400
H	1.321313	0.305664	-2.124511
C	-1.570408	-0.150264	-2.961110
O	-2.275677	-0.679050	-3.771146

IV

H	-4.417833	5.117286	1.101067
H	-4.959900	3.227907	2.630317
C	-3.930688	4.149219	0.966415
C	-4.231627	3.091873	1.828103
H	-2.794995	4.776837	-0.761720
C	-3.022779	3.960899	-0.072137
C	-3.610800	1.857913	1.662293
H	-3.877760	1.033757	2.330755
H	-4.823107	-3.980904	0.786390
H	-6.282198	-3.234088	-1.087602
H	0.514435	6.975256	-1.331585
H	0.451589	5.149968	-3.025716
C	-2.398586	2.724250	-0.243302
C	-4.618987	-3.038597	0.273080
C	-5.433923	-2.621001	-0.776426
C	-2.672489	1.669799	0.633821
H	-2.900260	-2.622859	1.488123
C	0.603491	5.933251	-1.017272
C	-3.533324	-2.260316	0.674693
C	0.570402	4.911891	-1.966718
H	-1.713060	2.572576	-1.076768
C	-5.167981	-1.414054	-1.425454
H	-5.807292	-1.078418	-2.244410
H	0.781144	6.420487	1.079966
C	0.751918	5.623197	0.334402
C	-3.262773	-1.047088	0.031445
H	-2.080445	0.041245	2.842043
C	-4.092007	-0.630749	-1.023006
C	0.680260	3.582131	-1.565884
H	0.618289	2.783186	-2.311072
H	-0.450651	2.096097	2.080980
H	-3.899553	0.325170	-1.521264
H	-0.496226	0.877457	4.475675
P	-1.856834	0.039973	0.437396
C	0.868274	4.295074	0.739417
C	-1.346482	-0.400706	2.151070
C	0.826775	3.264564	-0.209000
H	0.993992	4.072117	1.802246
H	-1.435105	-1.491658	2.275051
H	-0.192894	-0.870876	4.521829
C	0.434208	1.440818	2.019278
C	0.143984	0.076911	4.077944
H	1.182936	1.908019	2.675542
C	0.082957	0.028353	2.550532
H	1.169241	0.262975	4.430551
P	1.016504	1.512297	0.273270
Ru	-0.053981	-0.016471	-1.052177
H	3.082015	1.502480	2.517505
H	-2.224668	-5.035115	-1.292482
H	-1.136301	-2.819342	-1.352200
C	-1.493209	-4.776374	-0.523692
C	-0.875632	-3.524812	-0.559985
C	1.108421	-1.020403	2.073127
C	3.584369	1.400183	1.552472
H	1.045423	-1.908487	2.719669
C	2.853635	1.358933	0.357156
H	1.254710	-0.509126	-2.176898
H	-1.677506	-6.658337	0.518014
C	-1.189815	-5.681527	0.489184
H	2.120232	-0.615172	2.213075
P	0.939115	-1.566311	0.316028
C	0.060960	-3.175230	0.420223
H	5.526436	1.343852	2.483349
C	4.976694	1.310568	1.540159
C	-0.248682	-5.343251	1.464428
C	0.381291	-4.103320	1.425480
C	3.559382	1.248173	-0.850049
H	0.006332	-6.055596	2.251887
H	3.022983	1.241080	-1.802884
H	1.144662	-3.875919	2.175274
C	5.661978	1.188373	0.333962

C	2.620154	-2.084413	-0.195873
C	4.948064	1.163501	-0.863882
H	1.867880	-2.673665	-2.144534
H	3.690625	-1.651920	1.648515
H	6.751734	1.119363	0.325762
C	2.751250	-2.585872	-1.501005
C	3.753084	-2.020821	0.622707
H	5.474998	1.077356	-1.816421
C	3.991509	-2.991358	-1.983555
C	4.995806	-2.427250	0.135411
H	4.076706	-3.382038	-2.999585
H	5.872794	-2.364507	0.783633
C	5.119929	-2.906189	-1.166233
H	6.094395	-3.222842	-1.543558
H	-2.195358	-0.045324	-4.059547
O	-1.215341	1.071480	-2.648120
H	1.238894	0.331184	-2.248042
C	-1.569989	-0.032572	-3.143025
O	-1.221920	-1.119974	-2.612806

IV-V

H	6.678410	0.300605	0.603954
H	5.548643	1.205966	2.629874
C	5.608004	0.516120	0.582769
C	4.975464	1.019638	1.719220
H	5.357961	-0.102021	-1.472464
C	4.870714	0.292709	-0.578238
C	3.610364	1.298189	1.696826
H	3.147328	1.708596	2.597494
H	0.108039	6.317221	0.889315
H	0.935832	6.833035	-1.400552
H	5.673372	-3.911217	-1.184362
H	3.945307	-3.274958	-2.863498
C	3.502887	0.557366	-0.602743
C	0.494039	5.518287	0.252750
C	0.958144	5.806500	-1.029006
C	2.857269	1.060312	0.536768
H	0.133544	4.008907	1.733713
C	4.726395	-3.475994	-0.857982
C	0.514091	4.208301	0.729599
C	3.758796	-3.121281	-1.798614
H	2.929253	0.354221	-1.510797
C	1.459382	4.782773	-1.835093
H	1.832553	5.005529	-2.836600
H	5.233377	-3.560903	1.239832
C	4.481105	-3.279003	0.500013
C	1.010514	3.175618	-0.075795
H	1.205056	1.799954	2.825391
C	1.488852	3.476026	-1.360577
C	2.554578	-2.560530	-1.382939
H	1.814344	-2.254379	-2.128060
H	1.936233	-0.808156	2.346786
H	1.895760	2.678033	-1.989711
H	1.008955	0.130156	4.584047
P	1.061501	1.425275	0.431390
C	3.273351	-2.725220	0.922166
C	0.432192	1.387782	2.159565
C	2.304187	-2.354324	-0.018430
H	3.100974	-2.587744	1.992420
H	-0.409806	2.096507	2.212416
H	-0.593590	0.895211	4.595822
C	0.890315	-1.124906	2.209138
C	-0.019738	0.042769	4.204930
H	0.760936	-2.007979	2.853399
C	-0.046299	0.009160	2.677316
H	-0.454446	-0.873314	4.630455
P	0.701338	-1.609896	0.442468
Ru	-0.053125	0.044812	-0.918531
H	-0.408881	-3.583677	2.512905
H	-2.699991	4.827975	-1.080587
H	-1.572598	2.627796	-1.152953
C	-2.959151	4.066608	-0.341460
C	-2.318052	2.827641	-0.379490
C	-1.500353	-0.260329	2.226808
C	-0.810781	-3.805807	1.520842
H	-2.198688	0.287865	2.877467
C	-0.451496	-3.036625	0.407867
H	-1.503240	-1.457746	-3.479199
H	-4.418163	5.301473	0.661637
C	-3.919083	4.330519	0.631984
H	-1.725522	-1.329787	2.361192
P	-1.827260	0.200944	0.473041
C	-2.636850	1.842743	0.564020
H	-1.959011	-5.476116	2.253746
C	-1.688179	-4.881791	1.378373
C	-4.254505	3.346632	1.564913
C	-3.622956	2.107154	1.528646
C	-0.982054	-3.372083	-0.847154
H	-5.019425	3.543389	2.319025
H	-0.712722	-2.777056	-1.726074
H	-3.919929	1.338361	2.248535
C	-2.208042	-5.205094	0.126350

C	-3.147267	-0.898840	-0.134871
C	-1.852538	-4.447380	-0.989893
H	-3.025483	0.044381	-2.085372
H	-3.525358	-2.030081	1.683765
H	-2.891833	-6.050006	0.020036
C	-3.500111	-0.742065	-1.486183
C	-3.775951	-1.888177	0.629644
H	-2.258249	-4.690344	-1.974461
C	-4.457062	-1.572707	-2.063053
C	-4.736379	-2.713853	0.047088
H	-4.727103	-1.442433	-3.113290
H	-5.222259	-3.483639	0.650900
C	-5.074352	-2.562374	-1.296945
H	-5.827013	-3.212674	-1.747689
H	0.492495	1.172274	-4.379699
O	1.185414	0.062482	-2.800292
H	-0.813639	-1.658094	-3.724999
C	0.356289	0.857592	-3.323763
O	-0.628165	1.295763	-2.668972

V

H	1.766550	-6.458680	-1.792675
H	-0.491697	-6.002969	-2.739894
C	1.268172	-5.509390	-1.585405
C	0.002341	-5.253861	-2.117625
H	2.879936	-4.741684	-0.367335
C	1.888496	-4.551742	-0.787326
C	-0.638141	-4.047816	-1.850025
H	-1.637902	-3.871735	-2.258982
H	-5.631519	-2.932448	-1.376732
H	-5.991567	-3.424043	1.037527
H	6.915789	-1.974492	-0.100429
H	5.312855	-2.981825	-1.720670
C	1.247390	-3.340606	-0.520820
C	-4.824291	-2.773626	-0.658251
C	-5.025697	-3.047951	0.693399
C	-0.018633	-3.073966	-1.050173
H	-3.463292	-2.092827	-2.168849
C	5.926842	-1.533001	-0.240039
C	-3.592720	-2.294408	-1.102528
C	5.029455	-2.098049	-1.144382
H	1.731698	-2.592707	0.108695
C	-3.990500	-2.836471	1.603144
H	-4.143069	-3.039129	2.665480
H	6.263855	0.068538	1.171582
C	5.561265	-0.390759	0.472989
C	-2.546163	-2.079318	-0.194700
H	-1.073698	-1.634384	-3.122082
C	-2.761777	-2.351359	1.163156
C	3.767870	-1.532994	-1.329753
H	3.088468	-1.996888	-2.047492
H	1.398452	-1.051893	-2.674295
H	-1.973500	-2.144077	1.888912
H	0.018416	-0.343134	-4.873685
P	-0.884848	-1.484549	-0.699789
C	4.303424	0.175191	0.288764
C	-1.171785	-0.804667	-2.403784
C	3.384475	-0.394363	-0.608043
H	4.034006	1.075797	0.848177
H	-2.216538	-0.462617	-2.463004
H	-1.421773	0.666973	-4.636241
C	1.226416	0.022433	-2.498908
C	-0.372270	0.532531	-4.335569
H	1.900140	0.546474	-3.193474
C	-0.247900	0.354575	-2.822248
H	0.188892	1.413618	-4.679778
P	1.732525	0.398595	-0.769825
Ru	0.081575	-0.012809	0.753449
H	3.091322	2.104773	-2.820673
H	-5.396852	0.223824	1.781561
H	-2.955073	0.562075	1.668782
C	-4.947164	0.587288	0.854790
C	-3.570046	0.788966	0.794982
C	-0.679320	1.678216	-2.151217
C	2.914230	2.708315	-1.926414
H	-1.507726	2.135702	-2.710185
C	2.263827	2.164497	-0.806990
H	-6.821912	0.664137	-0.217242
C	-5.743338	0.830037	-0.262661
H	0.147839	2.399215	-2.223624
P	-1.148700	1.557048	-0.372688
C	-2.966863	1.249058	-0.385123
H	3.865224	4.436316	-2.789691
C	3.366318	4.025108	-1.909438
C	-5.155040	1.286835	-1.440434
C	-3.779148	1.502533	-1.500378
C	2.105573	2.957468	0.336622
H	-5.769856	1.486541	-2.320736
H	1.608668	2.552735	1.220475
H	-3.357768	1.873714	-2.437009
C	3.198887	4.810325	-0.766925
C	-1.087263	3.282424	0.235797

C	2.578099	4.270710	0.355849
H	-1.729606	2.659508	2.207091
H	-0.472742	4.244798	-1.616783
H	3.561143	5.840632	-0.754466
C	-1.427896	3.502320	1.578575
C	-0.744336	4.376889	-0.567140
H	2.443833	4.875425	1.255710
C	-1.406357	4.786894	2.112437
C	-0.729843	5.663781	-0.031015
H	-1.672805	4.943796	3.159672
H	-0.459370	6.508929	-0.667868
C	-1.052963	5.871337	1.308187
H	-1.037702	6.880298	1.725571
C	0.501816	-2.651175	2.697827
C	2.481988	-2.151410	3.880099
C	1.573120	-3.320150	3.531026
H	0.068375	-3.298331	1.918625
H	-0.318261	-2.248371	3.314981
H	3.483894	-2.452018	4.211319
H	2.037112	-1.536811	4.678939
H	2.120401	-4.065485	2.932039
H	1.161031	-3.840867	4.404388
O	1.166331	-1.540561	2.053724
C	2.501572	-1.388040	2.578616
H	2.705981	-0.312287	2.668982
H	3.218557	-1.828579	1.861243
C	-0.272208	0.739940	3.201660
O	-1.141403	-0.001772	2.669681
O	0.697890	1.192978	2.539703
H	-0.364180	1.001203	4.278433

VI

H	1.543870	-6.395800	-2.040161
H	-0.730671	-5.875857	-2.911011
C	1.068012	-5.444732	-1.792036
C	-0.207211	-5.153337	-2.281404
H	2.723273	-4.744388	-0.592293
C	1.726356	-4.522583	-0.982231
C	-0.820117	-3.945595	-1.960902
H	-1.827647	-3.736976	-2.334603
H	-5.834070	-2.261993	-1.653595
H	-6.235344	-3.250929	0.598134
H	6.875490	-2.269776	-0.023462
H	5.288104	-3.118123	-1.745368
C	1.114300	-3.309372	-0.662162
C	-5.017225	-2.336567	-0.932189
C	-5.241192	-2.889722	0.326416
C	-0.160802	-3.007808	-1.149947
H	-3.611675	-1.453067	-2.280130
C	5.909270	-1.781651	-0.166425
C	-3.749670	-1.873123	-1.281974
C	5.020297	-2.257576	-1.127968
H	1.624745	-2.594060	-0.013114
C	-4.187305	-2.985073	1.234606
H	-4.352095	-3.421031	2.222224
H	6.262056	-0.279637	1.347751
C	5.565441	-0.668575	0.602052
C	-2.686721	-1.952946	-0.371253
H	-1.064426	-1.428794	-3.178153
C	-2.921446	-2.520586	0.889776
C	3.787791	-1.631935	-1.318926
H	3.114431	-2.023658	-2.084118
H	1.402324	-1.031109	-2.644661
H	-2.110212	-2.579999	1.615163
H	0.126497	-0.133664	-4.853551
P	-0.977974	-1.406638	-0.753905
C	4.336409	-0.044298	0.413491
C	-1.135882	-0.622110	-2.431038
C	3.426826	-0.522797	-0.543773
H	4.082894	0.834095	1.015710
H	-2.151308	-0.211365	-2.517913
H	-1.254026	0.952060	-4.597771
C	1.310519	0.050637	-2.454001
C	-0.220340	0.741578	-4.285556
H	2.023363	0.538440	-3.135677
C	-0.133997	0.495081	-2.778515
H	0.400017	1.599693	-4.582694
P	1.831285	0.374677	-0.718361
Ru	0.108063	0.000730	0.777475
H	3.664114	1.657297	-2.614633
H	-5.507599	0.687867	1.637435
H	-3.045683	0.849649	1.648593
C	-4.983044	0.975966	0.723745
C	-3.594436	1.078735	0.735636
C	-0.507906	1.818288	-2.063019
C	3.391414	2.412414	-1.871669
H	-1.284074	2.340987	-2.638858
C	2.519433	2.091354	-0.816974
H	1.108122	1.149066	1.715138
H	-6.787656	1.146079	-0.453923
C	-5.698649	1.228774	-0.444951
H	0.355500	2.501932	-2.065084
P	-1.059544	1.642645	-0.311130
C	-2.892731	1.436527	-0.425551
H	4.622780	3.913026	-2.800825
C	3.947881	3.683338	-1.973604
C	-5.014456	1.590002	-1.603515
C	-3.624324	1.698128	-1.594369
C	2.255965	3.067802	0.148088
H	-5.563423	1.798079	-2.524609
H	1.605287	2.849929	0.995195
H	-3.129461	1.991351	-2.522649
C	3.654696	4.656802	-1.016203

C	-0.952650	3.330515	0.385605
C	2.816478	4.342781	0.049030
H	-0.942059	2.601487	2.428941
H	-0.961113	4.377679	-1.518554
H	4.091325	5.654346	-1.097722
C	-0.931444	3.482115	1.778789
C	-0.934632	4.469007	-0.429800
H	2.587909	5.089310	0.812983
C	-0.878585	4.753349	2.345876
C	-0.877647	5.739035	0.141714
H	-0.859812	4.859932	3.432396
H	-0.857794	6.620830	-0.502230
C	-0.846919	5.883491	1.528104
H	-0.802522	6.879741	1.973107
C	0.436602	-2.794374	2.551153
C	2.254493	-2.352286	3.966758
C	1.464125	-3.515970	3.389838
H	0.054601	-3.373067	1.696739
H	-0.422668	-2.464880	3.157782
H	3.235863	-2.635430	4.367282
H	1.688608	-1.862000	4.773576
H	2.107175	-4.147680	2.755780
H	1.007304	-4.164275	4.148058
O	1.127202	-1.635034	2.037029
C	2.357805	-1.434481	2.773097
H	2.440179	-0.369007	3.029813
H	3.199388	-1.710917	2.115052
H	0.634291	0.887637	2.383115
O	-1.397777	-0.340513	2.306940
C	-1.092787	-0.033616	3.508651
H	-1.873777	-0.309744	4.256235
O	-0.058634	0.516382	3.899577

VI-VII

H	1.042937	-6.491837	-2.069734
H	-1.242105	-5.857348	-2.828252
C	0.635142	-5.511278	-1.815312
C	-0.646122	-5.155418	-2.241148
H	2.390929	-4.883356	-0.720901
C	1.387395	-4.613321	-1.061181
C	-1.172883	-3.908689	-1.913923
H	-2.184193	-3.649451	-2.242234
H	-5.996953	-1.916153	-1.677234
H	-6.495154	-2.737512	0.621235
H	6.723785	-2.652522	-0.029459
H	5.092736	-3.403330	-1.755857
C	0.859970	-3.363542	-0.733762
C	-5.196355	-2.002122	-0.939036
C	-5.474859	-2.461841	0.346135
C	-0.421079	-2.996135	-1.157779
H	-3.711390	-1.301715	-2.312860
C	5.787194	-2.109501	-0.171953
C	-3.894827	-1.649072	-1.293820
C	4.873320	-2.531228	-1.135538
H	1.442160	-2.664855	-0.128923
C	-4.442613	-2.571091	1.277334
H	-4.651264	-2.931717	2.286970
H	6.226344	-0.631705	1.343451
C	5.507877	-0.979135	0.597913
C	-2.851597	-1.745742	-0.362103
H	-1.209401	-1.343998	-3.174803
C	-3.143324	-2.213963	0.927407
C	3.678421	-1.835774	-1.324596
H	2.984005	-2.186086	-2.091258
H	1.292969	-1.135720	-2.606691
H	-2.349050	-2.275038	1.672232
H	0.089756	-0.144125	-4.844774
P	-1.102972	-1.333163	-0.744793
C	4.317349	-0.284095	0.410028
C	-1.212614	-0.538213	-2.423047
C	3.380864	-0.709892	-0.546237
H	4.113354	0.606800	1.012587
H	-2.193599	-0.047211	-2.501515
H	-1.208463	1.038807	-4.589527
C	1.283087	-0.045788	-2.442091
C	-0.193205	0.753480	-4.276172
H	2.025468	0.368276	-3.140531
C	-0.127279	0.499250	-2.769368
H	0.488264	1.564776	-4.571588
P	1.831555	0.267021	-0.713653
Ru	0.123533	-0.022468	0.779956
H	3.595902	1.546719	-2.704049
H	-5.364295	0.946711	1.749730
H	-2.899839	0.979988	1.703081
C	-4.847447	1.243848	0.834451
C	-3.455297	1.271614	0.811846
C	-0.404861	1.843529	-2.054195
C	3.412794	2.275667	-1.909823
H	-1.168024	2.402440	-2.612718
C	2.601320	1.947993	-0.810238
H	1.132280	0.972565	1.811754
H	-6.668409	1.555731	-0.287511
C	-5.576651	1.581459	-0.303868
H	0.494002	2.478486	-2.089352
P	-0.915497	1.714700	-0.283508
C	-2.762948	1.636383	-0.352087
H	4.646804	3.760725	-2.860602
C	4.021897	3.523753	-1.996887
C	-4.902034	1.953606	-1.464601
C	-3.508304	1.986058	-1.488432
C	2.455958	2.888521	0.213993
H	-5.461265	2.228134	-2.361781
H	1.855486	2.648492	1.091905
H	-3.021399	2.291523	-2.416871
C	3.845532	4.463851	-0.979615

C	-0.682193	3.409269	0.372589
C	3.071767	4.138453	0.130271
H	-0.655135	2.721421	2.424750
H	-0.681810	4.419757	-1.551259
H	4.323266	5.443282	-1.050203
C	-0.613629	3.589507	1.760044
C	-0.619794	4.529286	-0.465704
H	2.936456	4.858486	0.940589
C	-0.471222	4.865145	2.300220
C	-0.471740	5.804770	0.076886
H	-0.416522	4.992257	3.383417
H	-0.416863	6.670741	-0.586235
C	-0.395429	5.975311	1.458360
H	-0.280285	6.975754	1.880699
C	0.206454	-2.891850	2.481896
C	2.214446	-2.791802	3.709005
C	1.198567	-3.793288	3.181839
H	-0.298985	-3.356900	1.621674
H	-0.567323	-2.519386	3.173309
H	3.177883	-3.238511	3.984500
H	1.821951	-2.265188	4.593112
H	1.672347	-4.478420	2.460847
H	0.730263	-4.407679	3.960952
O	0.984582	-1.769767	2.007766
C	2.322744	-1.843430	2.541361
H	2.644959	-0.823459	2.795007
H	2.997078	-2.239180	1.760374
H	0.738493	0.754478	2.699480
O	-1.322308	-0.230558	2.464736
C	-0.888669	0.070325	3.599597
H	-1.553807	-0.102745	4.470645
O	0.246875	0.561554	3.850461

VII

H	-0.000549	-6.558206	-2.162570
H	-2.177478	-5.585918	-2.878923
C	-0.256769	-5.532086	-1.890624
C	-1.477385	-4.986505	-2.293001
H	1.587087	-5.182993	-0.816107
C	0.629359	-4.764135	-1.137807
C	-1.810474	-3.679533	-1.945416
H	-2.775907	-3.268667	-2.256997
H	-6.261605	-0.944551	-1.664446
H	-6.873971	-1.662423	0.640756
H	6.246689	-3.598774	0.015072
H	4.531067	-4.118762	-1.713692
C	0.294295	-3.455392	-0.788736
C	-5.481165	-1.157066	-0.930204
C	-5.823955	-1.558801	0.358929
C	-0.923567	-2.896934	-1.190914
H	-3.908200	-0.717130	-2.316046
C	5.401485	-2.924573	-0.138377
C	-4.141267	-1.024334	-1.294036
C	4.439758	-3.217169	-1.103431
H	0.980618	-2.855084	-0.185806
C	-4.817919	-1.830365	1.285750
H	-5.076982	-2.146798	2.298658
H	6.047862	-1.511215	1.364038
C	5.289037	-1.756561	0.617813
C	-3.121765	-1.286188	-0.368245
H	-1.448158	-1.119784	-3.181080
C	-3.480204	-1.690771	0.926407
C	3.361680	-2.354575	-1.307763
H	2.626309	-2.605736	-2.075251
H	1.070028	-1.325642	-2.579243
H	-2.701812	-1.877444	1.668480
H	0.039807	-0.151893	-4.839744
P	-1.327871	-1.151405	-0.746887
C	4.215420	-0.895564	0.414977
C	-1.313860	-0.333877	-2.420141
C	3.230789	-1.188331	-0.542800
H	4.140053	0.021067	1.008462
H	-2.200856	0.314270	-2.487436
H	-1.052612	1.223181	-4.583624
C	1.236108	-0.245224	-2.436465
C	-0.096332	0.778720	-4.270030
H	2.026781	0.029507	-3.150340
C	-0.072950	0.514824	-2.763425
H	0.706476	1.471003	-4.564043
P	1.840504	0.002765	-0.716097
Ru	0.139797	-0.043721	0.779473
H	3.664904	1.091664	-2.789323
H	-5.111283	1.689091	1.817475
H	-2.674499	1.340766	1.728813
C	-4.564101	1.942274	0.906519
C	-3.185140	1.752869	0.859026
C	-0.133997	1.885172	-2.049040
C	3.634198	1.805114	-1.961547
H	-0.820906	2.546361	-2.594332
C	2.844016	1.555413	-0.826890
H	1.253340	0.748730	1.771383
H	-6.325786	2.582700	-0.169486
C	-5.243610	2.439230	-0.203479
H	0.846580	2.381611	-2.109471
P	-0.615096	1.836422	-0.264370
C	-2.454717	2.056807	-0.298541
H	5.017014	3.134226	-2.938329
C	4.412781	2.955946	-2.046198
C	-4.530527	2.753892	-1.358274
C	-3.149165	2.570689	-1.404426
C	2.891683	2.468042	0.231749
H	-5.049033	3.152833	-2.232936
H	2.303701	2.278162	1.131078
H	-2.629940	2.842180	-2.325768
C	4.430982	3.871300	-0.992320

C	-0.105725	3.480355	0.369934
C	3.678711	3.617551	0.150705
H	-0.139002	2.801651	2.421528
H	0.013628	4.471409	-1.559849
H	5.041498	4.774109	-1.061636
C	0.020684	3.654278	1.753866
C	0.115122	4.573749	-0.476464
H	3.693061	4.318962	0.988189
C	0.373091	4.893009	2.283066
C	0.475731	5.811705	0.053766
H	0.470233	5.014946	3.364031
H	0.654030	6.655235	-0.616574
C	0.606791	5.973881	1.431984
H	0.888246	6.945003	1.844619
C	-0.237671	-2.933108	2.429299
C	1.805044	-3.296358	3.549325
C	0.603009	-4.051829	3.003505
H	-0.845056	-3.226143	1.559435
H	-0.914075	-2.500428	3.186085
H	2.683564	-3.929003	3.727387
H	1.556013	-2.796023	4.498703
H	0.915770	-4.744716	2.206318
H	0.063505	-4.639803	3.756502
O	0.705239	-1.918258	2.017652
C	2.029989	-2.280829	2.457130
H	2.548657	-1.361366	2.765130
H	2.583587	-2.721327	1.608432
H	0.856292	0.524111	3.074001
O	-1.292592	-0.044277	2.555827
C	-0.848947	0.120125	3.689822
H	-1.502266	0.030422	4.577062
O	0.378373	0.408115	3.980898

VIII

H	5.055159	4.844890	-0.820773
H	5.589882	2.848865	-2.212491
C	4.467383	3.930745	-0.714587
C	4.764782	2.813437	-1.498011
H	3.188201	4.735700	0.830588
C	3.426813	3.870362	0.207977
C	4.017766	1.647286	-1.363961
H	4.281194	0.773566	-1.968736
H	5.399058	-3.518141	-1.305096
H	6.244744	-3.580483	1.035893
H	-0.141127	7.062502	1.562402
H	0.197191	6.509957	-0.841661
C	2.678533	2.699713	0.342099
C	4.958466	-2.868918	-0.545344
C	5.432429	-2.902946	0.764719
C	2.955552	1.579786	-0.447554
H	3.567054	-1.998839	-1.928614
C	-0.273655	6.036895	1.211915
C	3.920295	-2.005604	-0.894685
C	-0.083956	5.727578	-0.133511
H	1.864902	2.673809	1.070021
C	4.870711	-2.063180	1.727278
H	5.243384	-2.079951	2.753635
H	-0.803482	5.270667	3.161920
C	-0.642029	5.033073	2.108401
C	3.345460	-1.165022	0.067465
H	2.313645	0.034047	-2.699804
C	3.834391	-1.200457	1.381551
C	-0.250742	4.418765	-0.584536
H	-0.096450	4.204076	-1.644306
H	0.805240	2.182679	-1.881273
H	3.395330	-0.547294	2.141165
H	0.897294	1.079447	-4.340321
P	1.991881	0.014278	-0.289669
C	-0.806969	3.724841	1.660919
C	1.522320	-0.351599	-2.037278
C	-0.604102	3.401828	0.311656
H	-1.100460	2.944184	2.370234
H	1.524768	-1.447569	-2.162231
H	0.472803	-0.638435	-4.482473
C	-0.134645	1.608035	-1.917416
C	0.178886	0.312755	-4.015736
H	-0.784295	2.165390	-2.608132
C	0.148026	0.195621	-2.490423
H	-0.808066	0.588343	-4.416107
P	-0.846921	1.663160	-0.216944
Ru	0.042713	0.043226	1.107136
H	-2.709300	2.068158	-2.583534
H	2.374163	-5.025842	0.356512
H	1.415024	-2.776727	0.710732
C	1.439723	-4.754910	-0.140736
C	0.902638	-3.480866	0.054815
C	-0.983289	-0.786692	-2.127956
C	-3.298784	1.875218	-1.684049
H	-0.949304	-1.657458	-2.802189
C	-2.679325	1.623415	-0.451495
H	-1.388102	0.025285	1.963892
H	1.205476	-6.669101	-1.110332
C	0.786930	-5.671642	-0.960220
H	-1.944440	-0.287594	-2.317946
P	-1.002090	-1.430312	-0.388042
C	-0.289567	-3.113404	-0.576961
H	-5.151361	2.076819	-2.764607
C	-4.689295	1.883914	-1.793936
C	-0.410990	-5.316436	-1.585001
C	-0.947991	-4.047428	-1.394240
C	-3.491761	1.401049	0.669751
H	-0.932265	-6.035383	-2.220551
H	-3.031335	1.193094	1.638932
H	-1.894870	-3.785362	-1.877467
C	-5.483496	1.658681	-0.671721

C	-2.768144	-1.808091	-0.069765
C	-4.879341	1.422447	0.562332
H	-2.316957	-2.418387	1.958786
H	-3.571357	-1.316984	-2.032355
H	-6.572104	1.668569	-0.758739
C	-3.099037	-2.289426	1.205355
C	-3.784282	-1.675432	-1.023196
H	-5.491932	1.242221	1.448448
C	-4.413095	-2.613449	1.526367
C	-5.103112	-1.996146	-0.698789
H	-4.650459	-2.988746	2.523969
H	-5.884884	-1.877299	-1.452356
C	-5.422208	-2.460362	0.574395
H	-6.455305	-2.709783	0.825104
O	0.891015	-1.515887	2.507167
C	0.301787	-1.876794	3.519889
H	0.744927	-2.613580	4.211595
O	-0.867797	-1.456924	3.895995
H	-1.176575	-0.803055	3.187720
H	1.085596	1.107492	2.062687
H	0.302780	1.150058	2.443734

VIII-IX

H	-4.485225	5.315374	0.729237
H	-3.490283	4.381365	2.811097
C	-4.004798	4.334958	0.715126
C	-3.447197	3.813271	1.879517
H	-4.390126	3.995043	-1.385066
C	-3.950923	3.596104	-0.468133
C	-2.838273	2.557204	1.866497
H	-2.420152	2.171437	2.799905
H	-5.590383	-3.159070	1.056807
H	-6.067724	-3.244571	-1.383671
H	1.105084	6.867770	-1.000289
H	-0.953356	5.476719	-1.237308
C	-3.335220	2.348361	-0.486949
C	-5.010394	-2.545788	0.363697
C	-5.278060	-2.592809	-1.003733
C	-2.772783	1.811136	0.683162
H	-3.831300	-1.672640	1.933959
C	1.123891	5.809991	-0.730028
C	-4.010239	-1.708400	0.856277
C	-0.027610	5.032955	-0.864027
H	-3.276944	1.792912	-1.427103
C	-4.536747	-1.804997	-1.884146
H	-4.741316	-1.838416	-2.956529
H	3.205620	5.835458	-0.158064
C	2.301261	5.231703	-0.257657
C	-3.256228	-0.920904	-0.021890
H	-2.163989	0.164179	2.961780
C	-3.524039	-0.981482	-1.398787
C	-0.000374	3.681482	-0.525462
H	-0.900176	3.073050	-0.650884
H	-0.268508	2.040498	2.159565
H	-2.924224	-0.388959	-2.099053
H	-0.460954	0.791032	4.583403
P	-1.916678	0.195174	0.528921
C	2.335640	3.879815	0.083107
C	-1.486758	-0.319085	2.240993
C	1.181153	3.094032	-0.043284
H	3.268533	3.438586	0.442706
H	-1.701293	-1.398073	2.314510
H	-0.378224	-0.981545	4.592333
C	0.527951	1.279969	2.125598
C	0.072067	-0.074682	4.163363
H	1.309384	1.664791	2.798794
C	-0.006215	-0.084701	2.636397
H	1.114291	-0.024003	4.511410
P	1.165762	1.314977	0.396784
Ru	-0.087989	0.076633	-0.944855
H	3.186871	1.037521	2.650790
H	-2.552336	-4.399455	-2.120378
H	-0.964103	-2.523710	-1.823369
C	-2.028580	-4.280150	-1.169621
C	-1.132725	-3.228652	-0.999037
C	0.871051	-1.247259	2.124281
C	3.666435	0.819121	1.693235
H	0.668630	-2.150030	2.720191
C	2.945666	0.886911	0.492666
H	1.107718	-0.068272	-2.143263
H	-2.966242	-5.994230	-0.250156
C	-2.259873	-5.171210	-0.122556
H	1.927112	-1.001309	2.309536
P	0.705428	-1.632055	0.322071
C	-0.455117	-3.050898	0.215766
H	5.563162	0.416882	2.634048
C	5.016321	0.468069	1.690130
C	-1.583578	-5.012877	1.086470
C	-0.681355	-3.963336	1.254503
C	3.612582	0.614743	-0.711161
H	-1.753137	-5.715560	1.905034
H	3.065360	0.665562	-1.657530
H	-0.150249	-3.874603	2.205725
C	5.663989	0.188053	0.488584

C	2.317362	-2.380629	-0.133182
C	4.960605	0.269172	-0.713126
H	1.836230	-2.279606	-2.239863
H	3.100955	-2.648917	1.878303
H	6.719541	-0.092212	0.488229
C	2.578102	-2.585654	-1.495333
C	3.276683	-2.774749	0.807332
H	5.462008	0.053585	-1.659030
C	3.774909	-3.161214	-1.910071
C	4.481410	-3.340720	0.388913
H	3.962260	-3.317149	-2.974623
H	5.224993	-3.635282	1.132796
C	4.734340	-3.532859	-0.967271
H	5.677369	-3.978380	-1.290966
O	-0.896201	1.090401	-2.630850
C	0.283541	0.978747	-3.119890
H	0.475934	0.289166	-3.959195
O	1.100462	2.040123	-3.209997
H	0.781705	2.731123	-2.601689

IX

H	-1.947313	6.391155	0.009945
H	-2.362515	5.327477	2.221155
C	-1.905584	5.304453	0.110431
C	-2.134502	4.709604	1.349976
H	-1.444092	4.969442	-1.974656
C	-1.624841	4.509424	-1.000752
C	-2.082677	3.322055	1.482830
H	-2.278704	2.880179	2.463494
H	-6.297965	-1.005362	1.291192
H	-6.925964	-0.975144	-1.117247
H	2.081784	6.344733	-1.614023
H	1.867239	6.039348	0.846063
C	-1.561430	3.125372	-0.868609
C	-5.575691	-0.678374	0.540113
C	-5.926990	-0.659717	-0.808422
C	-1.787115	2.516834	0.375836
H	-4.057690	-0.289726	2.003676
C	1.959419	5.343175	-1.196181
C	-4.302461	-0.274307	0.939260
C	1.839513	5.172551	0.182125
H	-1.320495	2.505694	-1.740976
C	-5.001634	-0.231568	-1.760851
H	-5.272697	-0.212315	-2.818664
H	2.019637	4.361538	-3.120793
C	1.925274	4.232989	-2.040436
C	-3.365419	0.144978	-0.014644
H	-2.042938	0.924960	2.824363
C	-3.726000	0.167602	-1.370632
C	1.687893	3.895892	0.721894
H	1.606996	3.784766	1.806226
H	0.248413	2.158824	2.286460
H	-2.995528	0.467312	-2.126361
H	-0.395311	1.064008	4.610275
P	-1.674895	0.686998	0.423733
C	1.762831	2.957670	-1.506401
C	-1.493922	0.207300	2.195454
C	1.644834	2.777836	-0.119440
H	1.713252	2.090475	-2.175991
H	-2.018960	-0.754080	2.317497
H	-0.775908	-0.669745	4.637307
C	0.835071	1.228257	2.227432
C	-0.077622	0.079466	4.237216
H	1.687333	1.375968	2.907078
C	-0.045625	0.051721	2.709599
H	0.915269	-0.131575	4.660891
P	1.416529	1.068993	0.490750
Ru	0.017199	-0.039455	-0.897170
H	3.379748	0.423119	2.750789
H	-3.921219	-3.492723	-1.529880
H	-1.898513	-2.064086	-1.434774
C	-3.262532	-3.548867	-0.660466
C	-2.120274	-2.751625	-0.609790
C	0.540101	-1.313321	2.272316
C	3.831538	0.197839	1.781466
H	0.137182	-2.110631	2.914947
C	3.131649	0.418042	0.587922
H	-4.470759	-5.013712	0.367398
C	-3.569502	-4.397897	0.400887
H	1.627636	-1.310290	2.444038
P	0.231760	-1.743001	0.506947
C	-1.267292	-2.803008	0.502764
H	5.661160	-0.480892	2.696923
C	5.128415	-0.317589	1.757474
C	-2.720574	-4.464812	1.506361
C	-1.570964	-3.679465	1.555184
C	3.765113	0.122268	-0.630415
H	-2.950497	-5.138843	2.334209
H	3.236866	0.294973	-1.572354
H	-0.907878	-3.769302	2.419891
C	5.744944	-0.611376	0.543145
C	1.567164	-2.874678	-0.019641

C	5.060921	-0.385204	-0.651863
H	0.375039	-3.527251	-1.712425
H	3.011351	-2.457559	1.550976
H	6.761909	-1.009168	0.527172
C	1.350266	-3.584902	-1.215389
C	2.804077	-2.995540	0.623761
H	5.540814	-0.604840	-1.608084
C	2.353670	-4.384416	-1.756881
C	3.803241	-3.804963	0.082791
H	2.168488	-4.938092	-2.680016
H	4.761103	-3.892024	0.600449
C	3.585797	-4.493252	-1.108382
H	4.371250	-5.124417	-1.528753
O	-0.906766	-0.188537	-2.728723
C	0.071073	-0.714085	-3.479696
H	-0.071523	-1.787307	-3.737085
O	1.307086	-0.630256	-2.654252
H	1.728816	-1.501006	-2.575536
H	0.274039	-0.150525	-4.411047

X

H	5.713083	-3.891358	-0.962780
H	4.476028	-3.335905	-3.052682
C	4.697765	-3.492943	-0.910092
C	4.006518	-3.181385	-2.078819
H	4.609334	-3.563403	1.247224
C	4.079439	-3.306656	0.326854
C	2.706908	-2.677876	-2.012857
H	2.197302	-2.441800	-2.950044
H	-2.256807	-6.046628	-0.574090
H	-2.324413	-6.187438	1.909304
H	6.715501	2.116481	0.055385
H	5.969274	0.019452	-1.072274
C	2.783162	-2.803675	0.395409
C	-1.771929	-5.277782	0.031401
C	-1.809732	-5.355912	1.423019
C	2.080652	-2.474728	-0.774636
H	-1.082788	-4.184984	-1.686794
C	5.651895	1.926684	-0.104386
C	-1.112156	-4.221439	-0.594490
C	5.235101	0.755422	-0.736158
H	2.309182	-2.662392	1.369375
C	-1.188204	-4.372475	2.192680
H	-1.214802	-4.430471	3.282971
H	5.016978	3.772518	0.822472
C	4.702344	2.854003	0.322608
C	-0.496179	-3.224441	0.171873
H	0.262867	-2.351758	-2.958393
C	-0.539672	-3.308447	1.571322
C	3.879205	0.517811	-0.950914
H	3.582319	-0.408223	-1.445255
H	1.885255	-0.114478	-2.724583
H	-0.078708	-2.520900	2.178906
H	0.498265	-0.811799	-4.830469
P	0.380536	-1.803549	-0.579190
C	3.345110	2.621875	0.104873
C	-0.279338	-1.665733	-2.292011
C	2.915793	1.455197	-0.547747
H	2.619283	3.366475	0.440397
H	-1.312551	-2.046858	-2.264378
H	-1.264413	-0.966672	-4.687166
C	1.012634	0.536330	-2.553492
C	-0.401271	-0.349409	-4.397870
H	1.133105	1.375146	-3.256441
C	-0.289255	-0.234103	-2.877923
H	-0.525188	0.637504	-4.866686
P	1.120773	1.164066	-0.820220
Ru	-0.038362	-0.007292	0.703454
H	1.036444	3.375437	-2.896747
H	-4.354604	-3.131502	1.906770
H	-2.710328	-1.290629	1.630010
C	-4.224607	-2.665075	0.927487
C	-3.304963	-1.629964	0.775983
C	-1.511594	0.557366	-2.363641
C	0.564672	3.724502	-1.974121
H	-2.405348	0.317572	-2.958749
C	0.462016	2.882127	-0.859087
H	-5.680242	-3.930016	-0.047367
C	-4.965150	-3.112990	-0.165569
H	-1.326397	1.627947	-2.536031
P	-1.905891	0.351407	-0.574951
C	-3.122943	-1.020941	-0.475136
H	0.162024	5.676262	-2.794997
C	0.081231	5.031193	-1.917446
C	-4.790930	-2.513210	-1.411730
C	-3.880670	-1.468294	-1.566282
C	-0.113277	3.384242	0.316939
H	-5.372309	-2.853810	-2.271228
H	-0.205643	2.727953	1.191576
H	-3.776692	-1.009472	-2.552689
C	-0.489522	5.518632	-0.741494
C	-2.920382	1.823903	-0.164333

C	-0.579587	4.695004	0.379481
H	-3.279248	1.095437	1.840132
H	-2.770719	2.845139	-2.083877
H	-0.863137	6.543999	-0.699608
C	-3.467111	1.900923	1.127711
C	-3.169906	2.865355	-1.068222
H	-1.029373	5.070779	1.301512
C	-4.227466	3.004316	1.505758
C	-3.935574	3.965728	-0.684502
H	-4.650647	3.049127	2.511980
H	-4.119812	4.768015	-1.402619
C	-4.461728	4.042191	0.602687
H	-5.062968	4.904192	0.899703
O	-1.198278	0.247614	2.371477
C	-0.731535	0.461762	3.622719
H	-1.550258	0.377638	4.365998
O	-0.148854	1.771119	3.752077
H	-0.824463	2.413014	3.492995
H	0.085314	-0.218613	3.942301
O	1.771433	-0.296436	1.991342
O	2.377258	1.562898	3.102069
H	1.393378	1.740340	3.195923
C	2.622568	0.404021	2.549647
C	4.041592	-0.024017	2.669770
H	4.700849	0.787523	2.335305
H	4.283360	-0.202768	3.726305
H	4.233355	-0.928204	2.085564

X-XI

H	-5.221767	-4.123322	-0.978624
H	-4.459970	-3.065630	-3.101284
C	-4.558426	-3.256054	-0.952235
C	-4.129892	-2.666827	-2.139588
H	-4.460209	-3.193658	1.204950
C	-4.133839	-2.734196	0.269229
C	-3.282216	-1.559633	-2.106949
H	-2.972536	-1.117453	-3.056696
H	-3.062877	5.050276	0.570907
H	-5.495145	4.710683	0.127134
H	-2.269072	-6.381593	0.959340
H	-1.785789	-5.953689	-1.444672
C	-3.283469	-1.632603	0.302572
C	-3.430230	4.081522	0.224461
C	-4.789340	3.890751	-0.022472
C	-2.844237	-1.029953	-0.885439
H	-1.473983	3.192446	0.259384
C	-1.749187	-5.469048	0.659742
C	-2.530160	3.034751	0.040093
C	-1.478291	-5.229595	-0.686901
H	-2.944006	-1.241009	1.265466
C	-5.244828	2.647107	-0.456563
H	-6.308053	2.488572	-0.649198
H	-1.552397	-4.721140	2.678970
C	-1.349618	-4.541709	1.620812
C	-2.977726	1.782462	-0.406327
H	-2.026716	0.563477	-3.149847
C	-4.347752	1.597331	-0.648599
C	-0.804557	-4.072228	-1.074975
H	-0.580776	-3.921383	-2.134229
H	-0.734884	-1.846721	-2.570210
H	-4.724993	0.630535	-0.990192
H	-0.639156	-0.598482	-4.787359
P	-1.759319	0.445179	-0.729879
C	-0.693094	-3.376349	1.232545
C	-1.213275	0.801679	-2.448516
C	-0.409995	-3.129629	-0.117262
H	-0.396720	-2.651300	1.993814
H	-1.104471	1.896514	-2.510853
H	-0.006978	1.058503	-4.835833
C	0.214141	-1.334871	-2.348558
C	0.147272	0.062963	-4.394973
H	0.978449	-1.874678	-2.928499
C	0.109683	0.122027	-2.866601
H	1.112726	-0.316970	-4.759806
P	0.552352	-1.624349	-0.540871
Ru	0.008208	0.177332	0.736948
H	2.965934	-1.230613	-2.288499
H	0.983310	5.436701	2.107983
H	1.529026	3.058362	1.667640
C	0.847735	5.026674	1.105049
C	1.161059	3.694243	0.856411
C	1.294283	0.982260	-2.399591
C	3.222542	-1.929167	-1.490056
H	1.303679	1.928116	-2.962302
C	2.263728	-2.319648	-0.545117
H	0.121796	6.884110	0.271101
C	0.365857	5.837662	0.075709
H	2.244133	0.495702	-2.663247
P	1.370342	1.369562	-0.590686
C	0.994561	3.152954	-0.429052
H	5.251544	-2.100932	-2.188634
C	4.524331	-2.422371	-1.439917
C	0.205165	5.311418	-1.204424
C	0.517551	3.975512	-1.457251
C	2.645392	-3.240181	0.443709
H	-0.163690	5.942659	-2.015562
H	1.911044	-3.609780	1.163800
H	0.381471	3.592545	-2.471751
C	4.895646	-3.315984	-0.437966
C	3.180493	1.356601	-0.286675

C	3.949753	-3.727108	0.500295
H	3.496030	3.000373	-1.672344
H	3.193837	-0.254923	1.140433
H	5.915752	-3.703330	-0.396750
C	3.968001	2.273138	-1.003605
C	3.801193	0.453773	0.580670
H	4.221942	-4.447956	1.274282
C	5.352241	2.273653	-0.860974
C	5.188241	0.463933	0.727528
H	5.954948	2.990526	-1.422622
H	5.662526	-0.246688	1.408443
C	5.964935	1.367733	0.007114
H	7.050864	1.373450	0.123877
O	1.334500	0.078079	2.424763
C	1.601670	-1.008721	3.091044
H	1.820976	-1.921197	2.496668
O	0.365435	-1.511540	3.917259
H	-0.678114	-0.894381	3.350027
H	2.380899	-0.888538	3.872739
O	-1.331375	-0.294866	2.681122
O	-0.829737	1.765097	2.131507
C	-1.277621	0.999372	2.979978
H	0.485971	-1.259282	4.841638
C	-1.774169	1.431416	4.308949
H	-2.826381	1.142697	4.429149
H	-1.214198	0.913807	5.100056
H	-1.667641	2.511592	4.437866

XI

H	1.158983	-6.602967	-0.894041
H	0.043468	-5.647883	-2.905531
C	0.713383	-5.606734	-0.851903
C	0.091965	-5.071479	-1.979186
H	1.250483	-5.275990	1.213832
C	0.762516	-4.865409	0.327036
C	-0.481337	-3.802023	-1.927634
H	-0.975722	-3.419189	-2.823609
H	-6.185807	-1.794787	-0.912825
H	-6.391104	-2.811144	1.350559
H	4.939966	-4.805903	0.602905
H	4.410742	-4.325925	-1.782703
C	0.204504	-3.589653	0.378537
C	-5.294375	-1.966869	-0.305700
C	-5.409017	-2.535696	0.960635
C	-0.424671	-3.042961	-0.749790
H	-3.985135	-1.152356	-1.796477
C	4.315844	-3.948510	0.341857
C	-4.042469	-1.609013	-0.805911
C	4.018501	-3.681040	-0.993278
H	0.276134	-3.002913	1.298615
C	-4.263705	-2.762260	1.725889
H	-4.345488	-3.218715	2.714694
H	4.053847	-3.311207	2.388534
C	3.819132	-3.111428	1.340140
C	-2.890021	-1.825547	-0.041728
H	-1.638987	-1.607971	-2.976094
C	-3.013500	-2.413828	1.226211
C	3.225442	-2.585572	-1.331292
H	3.024208	-2.392981	-2.387445
H	1.084053	-1.658707	-2.582329
H	-2.117248	-2.613712	1.821627
H	-0.119003	-1.022167	-4.776637
P	-1.201034	-1.382991	-0.588528
C	3.016058	-2.023430	1.008012
C	-1.400503	-0.764271	-2.312063
C	2.707401	-1.751654	-0.331230
H	2.627049	-1.380453	1.799914
H	-2.297533	-0.124884	-2.323693
H	-1.267117	0.332549	-4.763217
C	1.143936	-0.566973	-2.453744
C	-0.283780	-0.001242	-4.401899
H	1.939006	-0.237716	-3.139806
C	-0.207025	0.047521	-2.875795
H	0.476576	0.647871	-4.860695
P	1.676889	-0.279243	-0.707844
Ru	-0.070661	0.103028	0.717758
H	2.851272	1.521494	-2.889069
H	-5.440350	1.218945	0.937498
H	-3.008723	0.773761	0.979032
C	-4.793554	1.700968	0.200795
C	-3.421581	1.441479	0.220314
C	-0.306173	1.524826	-2.440464
C	3.334734	1.726899	-1.930096
H	-1.046095	2.049368	-3.064252
C	2.969229	1.026847	-0.773336
H	-6.405973	2.761674	-0.766086
C	-5.332298	2.562052	-0.750719
H	0.656039	2.025089	-2.627400
P	-0.766285	1.750334	-0.670750
C	-2.578659	2.046591	-0.718910
H	4.611594	3.230651	-2.799701
C	4.335446	2.698970	-1.886292
C	-4.494579	3.183043	-1.680602
C	-3.126074	2.933193	-1.661588
C	3.642781	1.308369	0.428035
H	-4.909616	3.872992	-2.418416
H	3.396671	0.758587	1.344670
H	-2.481329	3.451839	-2.377742
C	4.987797	2.980263	-0.688151
C	-0.140275	3.399646	-0.187607

C	4.642180	2.277692	0.467109
H	-1.597112	3.520947	1.414459
H	1.453864	3.587711	-1.653329
H	5.774880	3.736520	-0.656706
C	-0.739354	4.001619	0.932311
C	0.960284	4.026368	-0.784483
H	5.161423	2.478669	1.407135
C	-0.244232	5.199434	1.441502
C	1.454460	5.227316	-0.271864
H	-0.723868	5.658368	2.308828
H	2.311677	5.704471	-0.752293
C	0.857093	5.814493	0.841811
H	1.243434	6.755258	1.239420
O	0.857073	1.460894	2.205558
C	1.516051	2.463543	2.378249
H	1.894861	3.087893	1.539894
O	2.647854	0.030410	3.696199
H	1.827385	-0.433996	3.455543
H	1.776703	2.778319	3.410320
O	0.219479	-1.171118	2.562184
O	-1.518077	0.147422	2.391569
C	-0.818073	-0.641797	3.085717
H	3.174712	-0.646243	4.134407
C	-1.171207	-0.934437	4.502976
H	-0.992811	-1.990196	4.739561
H	-0.518697	-0.342050	5.160623
H	-2.210060	-0.664331	4.718212

XII

H	-5.715267	-3.502543	-0.875191
H	-5.092587	-2.241116	-2.931560
C	-4.942835	-2.731614	-0.832606
C	-4.592712	-2.028463	-1.984126
H	-4.568186	-2.991704	1.278498
C	-4.302919	-2.444285	0.371234
C	-3.609458	-1.041932	-1.931862
H	-3.371151	-0.502215	-2.851106
H	-3.792971	5.062058	-1.195376
H	-4.866889	5.021640	1.050293
H	-2.752614	-6.273347	1.093061
H	-2.347001	-5.874223	-1.329630
C	-3.310118	-1.468057	0.425808
C	-3.656349	4.201213	-0.537237
C	-4.256934	4.178504	0.719449
C	-2.949854	-0.754698	-0.727250
H	-2.403635	3.180096	-1.947576
C	-2.163066	-5.411632	0.772440
C	-2.873318	3.128307	-0.962722
C	-1.937002	-5.188160	-0.585259
H	-2.794607	-1.273400	1.369497
C	-4.085561	3.070068	1.550431
H	-4.564514	3.040480	2.531630
H	-1.812669	-4.701510	2.783121
C	-1.634793	-4.533811	1.718618
C	-2.690332	2.016688	-0.132126
H	-1.964238	0.783911	-2.985929
C	-3.311251	1.995300	1.125984
C	-1.180364	-4.093348	-0.999540
H	-1.000672	-3.947338	-2.067879
H	-1.069778	-1.788470	-2.451087
H	-3.198868	1.118892	1.771796
H	-0.830090	-0.534578	-4.692279
P	-1.672311	0.563700	-0.576598
C	-0.892931	-3.428404	1.308938
C	-1.109931	0.880314	-2.300559
C	-0.658110	-3.200999	-0.054674
H	-0.524297	-2.715378	2.052596
H	-0.816424	1.940783	-2.354307
H	0.034565	1.016022	-4.726945
C	-0.032951	-1.447767	-2.300952
C	0.055891	-0.003095	-4.314908
H	0.583546	-2.103132	-2.935330
C	0.080552	0.016261	-2.786214
H	0.944299	-0.507789	-4.722469
P	0.365831	-1.757908	-0.524206
Ru	0.075329	0.069120	0.808998
H	2.609891	-2.102593	-2.579586
H	-0.776457	5.482350	0.808671
H	-0.314013	3.057918	0.965115
C	-0.088692	5.019873	0.096954
C	0.164400	3.649246	0.181638
C	1.411144	0.661018	-2.340516
C	2.910824	-2.535373	-1.621946
H	1.643671	1.514117	-2.996146
C	2.042297	-2.524456	-0.523284
H	0.321663	6.859573	-0.955441
C	0.524145	5.788411	-0.888305
H	2.231813	-0.056945	-2.479174
P	1.414492	1.244618	-0.591418
C	1.039012	3.039164	-0.724925
H	4.831011	-3.128215	-2.401932
C	4.173781	-3.122904	-1.529328
C	1.411977	5.189432	-1.785507
C	1.674538	3.825789	-1.700499
C	2.466013	-3.144287	0.665134
H	1.909772	5.790845	-2.549181
H	1.788493	-3.191438	1.523216
H	2.397072	3.380634	-2.391328
C	4.589096	-3.712720	-0.338232
C	3.165433	1.287063	-0.067288

C	3.725932	-3.729540	0.758238
H	2.646729	2.597152	1.581614
H	4.017715	-0.024006	-1.576463
H	5.574925	-4.176844	-0.269121
C	3.444009	2.021364	1.099099
C	4.195460	0.553365	-0.667399
H	4.030512	-4.215026	1.688257
C	4.721484	2.012009	1.652879
C	5.475048	0.543902	-0.108073
H	4.924978	2.591851	2.555861
H	6.268739	-0.032195	-0.589327
C	5.740025	1.267671	1.052785
H	6.742210	1.260617	1.486267
O	1.608355	-0.441721	2.344800
C	2.796231	-0.684339	2.418552
H	3.463328	-0.728187	1.531867
H	3.257142	-0.891001	3.408367
O	-1.180585	-0.512391	2.573820
O	-0.368785	1.509818	2.416336
C	-1.003328	0.636112	3.083545
C	-1.536159	0.970968	4.434214
H	-0.705730	1.205524	5.112553
H	-2.154226	1.876384	4.374288
H	-2.121670	0.146315	4.851975

XIII

H	2.818437	6.339580	-1.098963
H	4.134156	4.669143	-2.396343
C	2.604129	5.273992	-0.993362
C	3.340499	4.338033	-1.723302
H	1.023667	5.567386	0.451672
C	1.602994	4.843683	-0.126722
C	3.071047	2.979627	-1.589902
H	3.668726	2.255497	-2.152996
H	5.986450	-1.606474	-1.781313
H	7.006914	-1.175212	0.449169
H	-2.967254	6.442269	1.535745
H	-2.485348	6.109162	-0.884028
C	1.331588	3.480853	0.008884
C	5.427594	-1.060917	-1.018023
C	5.998538	-0.818546	0.229143
C	2.054368	2.537377	-0.727399
H	3.715146	-0.822349	-2.284344
C	-2.685263	5.454856	1.164421
C	4.137814	-0.610714	-1.299930
C	-2.414360	5.268805	-0.190144
H	0.547602	3.157612	0.694307
C	5.281398	-0.108373	1.193435
H	5.728063	0.094279	2.169314
H	-2.824663	4.512236	3.103578
C	-2.603926	4.374064	2.043003
C	3.406565	0.092287	-0.334707
H	1.851679	0.946305	-3.010490
C	3.997166	0.345669	0.912171
C	-2.054976	4.009348	-0.668192
H	-1.849533	3.888903	-1.734375
H	-0.288530	2.334144	-2.085046
H	3.440144	0.901836	1.670010
H	0.031855	1.314200	-4.578794
P	1.714360	0.732263	-0.601098
C	-2.243529	3.115287	1.569256
C	1.309020	0.262500	-2.337901
C	-1.960240	2.921659	0.209661
H	-2.176696	2.273893	2.264616
H	1.719640	-0.744000	-2.520208
H	0.286609	-0.437740	-4.710700
C	-0.948485	1.450775	-2.081273
C	-0.314431	0.336771	-4.212978
H	-1.799023	1.727585	-2.721664
C	-0.193619	0.241525	-2.691083
H	-1.357229	0.208226	-4.538824
P	-1.519742	1.232263	-0.345341
Ru	0.041115	0.055622	0.891945
H	-3.489232	0.776303	-2.612794
H	3.951776	-3.959536	0.107097
H	2.302790	-2.163629	0.539143
C	2.984787	-3.983213	-0.401824
C	2.060735	-2.962910	-0.162909
C	-0.821611	-1.097766	-2.256683
C	-3.922631	0.415984	-1.676827
H	-0.496281	-1.892544	-2.946347
C	-3.204741	0.483490	-0.474818
H	3.401277	-5.818638	-1.459244
C	2.678111	-5.020746	-1.277144
H	-1.912579	-1.024496	-2.364884
P	-0.445817	-1.670113	-0.531210
C	0.819750	-2.975781	-0.808033
H	-5.749092	-0.166219	-2.659126
C	-5.210495	-0.119121	-1.710140
C	1.436082	-5.045444	-1.916390
C	0.511827	-4.033235	-1.681557
C	-3.824103	0.019600	0.694758
H	1.183587	-5.863226	-2.594625
H	-3.298572	0.061423	1.652712
H	-0.466678	-4.079063	-2.170847
C	-5.809190	-0.581585	-0.540738
C	-1.908869	-2.667573	-0.055010

C	-5.112891	-0.505050	0.664598
H	-1.149885	-2.929049	1.965259
H	-2.944007	-2.642329	-1.971644
H	-6.817776	-0.999332	-0.567804
C	-1.953885	-3.157728	1.259250
C	-2.948530	-2.991285	-0.936710
H	-5.570612	-0.864352	1.588690
C	-3.026463	-3.936664	1.684772
C	-4.020895	-3.771218	-0.504915
H	-3.046564	-4.308507	2.711254
H	-4.827340	-4.008629	-1.202531
C	-4.065942	-4.241109	0.805651
H	-4.907938	-4.850590	1.140732
O	0.386797	1.673560	2.409669
C	1.094472	1.729932	3.393072
H	1.027189	2.602133	4.075935
H	1.826591	0.932387	3.640772
O	0.068107	-1.673031	3.550577
O	1.558501	-0.903540	2.070277
C	1.224579	-1.586798	3.112167
H	-1.381739	-0.339470	1.889736
H	-0.802549	-0.762288	2.373357
C	2.356041	-2.319315	3.776096
H	3.298038	-1.763157	3.694227
H	2.127188	-2.544103	4.822817
H	2.503078	-3.275199	3.252449

XIII-XIV

H	3.653172	5.933820	-1.130887
H	4.805330	4.081153	-2.332837
C	3.299140	4.906717	-1.019892
C	3.944300	3.868583	-1.695631
H	1.696985	5.431258	0.332941
C	2.208032	4.626687	-0.201343
C	3.496666	2.558571	-1.554759
H	4.021345	1.752296	-2.077489
H	5.783449	-2.306297	-1.764798
H	6.794299	-2.063630	0.497935
H	-2.236549	6.701450	1.569344
H	-1.725578	6.351092	-0.841898
C	1.758767	3.312524	-0.058216
C	5.278103	-1.714382	-0.998647
C	5.843882	-1.578030	0.266917
C	2.391525	2.267428	-0.738389
H	3.642208	-1.226547	-2.295983
C	-2.063782	5.693218	1.187175
C	4.062616	-1.097757	-1.296195
C	-1.776677	5.497386	-0.162644
H	0.904325	3.106617	0.587835
C	5.195767	-0.810067	1.235703
H	5.639213	-0.690267	2.226710
H	-2.374756	4.743665	3.102948
C	-2.139406	4.596608	2.046644
C	3.399920	-0.333195	-0.328146
H	1.976399	0.735113	-3.015696
C	3.985648	-0.191572	0.938529
C	-1.556285	4.211428	-0.654714
H	-1.332562	4.084026	-1.716484
H	0.005807	2.349752	-2.082318
H	3.481855	0.407443	1.701405
H	0.214007	1.303770	-4.589701
P	1.805253	0.524268	-0.603899
C	-1.919079	3.311332	1.558872
C	1.358631	0.112911	-2.347853
C	-1.619311	3.106591	0.204305
H	-1.975834	2.455362	2.237484
H	1.652857	-0.932584	-2.536818
H	0.266387	-0.465652	-4.722474
C	-0.753022	1.549765	-2.093249
C	-0.243030	0.372404	-4.225281
H	-1.557493	1.926680	-2.742075
C	-0.136465	0.263719	-2.703302
H	-1.293121	0.364702	-4.553156
P	-1.362395	1.384048	-0.364240
Ru	0.040208	0.071667	0.879207
H	-3.373779	1.250777	-2.632562
H	3.452436	-4.368010	0.255869
H	1.995302	-2.403387	0.634156
C	2.513670	-4.279467	-0.296509
C	1.696786	-3.166032	-0.085817
C	-0.914217	-0.994820	-2.270864
C	-3.840492	0.890416	-1.712690
H	-0.673295	-1.823633	-2.954984
C	-3.116802	0.821383	-0.513777
H	2.777292	-6.136094	-1.365980
C	2.137698	-5.265876	-1.203442
H	-1.988879	-0.798631	-2.391356
P	-0.627027	-1.594758	-0.536787
C	0.495087	-3.032281	-0.788743
H	-5.720921	0.553020	-2.707891
C	-5.177399	0.495777	-1.762265
C	0.932720	-5.145542	-1.900142
C	0.115014	-4.040121	-1.691536
C	-3.776167	0.364027	0.636178
H	0.625186	-5.922352	-2.603396
H	-3.235795	0.290158	1.583206
H	-0.838123	-3.973351	-2.226178
C	-5.818192	0.041280	-0.611790
C	-2.195649	-2.443018	-0.099319

C	-5.113607	-0.018779	0.589552
H	-1.521053	-2.769900	1.936629
H	-3.172370	-2.326728	-2.042092
H	-6.865504	-0.265738	-0.651583
C	-2.327094	-2.919346	1.213367
C	-3.239693	-2.665709	-1.006180
H	-5.603506	-0.377522	1.497407
C	-3.482846	-3.582689	1.614960
C	-4.397888	-3.328187	-0.600125
H	-3.567829	-3.945146	2.641537
H	-5.206180	-3.485402	-1.317944
C	-4.525788	-3.782902	0.710088
H	-5.434712	-4.299658	1.025411
O	0.564652	1.665976	2.341641
C	1.301285	1.736055	3.302594
H	1.322969	2.655571	3.923159
H	1.975076	0.901472	3.595100
O	-0.326673	-1.412673	3.605635
O	1.362866	-1.082168	2.193809
C	0.874929	-1.587703	3.246193
H	-1.328077	-0.231097	1.933470
H	-0.856760	-0.719900	2.630283
C	1.754660	-2.455455	4.086252
H	2.809026	-2.180866	3.969930
H	1.456721	-2.430722	5.139506
H	1.645919	-3.493159	3.739187

XIV

H	4.164300	5.623807	-1.115228
H	5.145750	3.694149	-2.347253
C	3.726651	4.628712	-1.010599
C	4.276347	3.547408	-1.702761
H	2.185554	5.271749	0.361467
C	2.622903	4.432299	-0.184530
C	3.721978	2.277851	-1.569586
H	4.171723	1.436213	-2.106347
H	5.617508	-2.720485	-1.856461
H	6.634721	-2.637470	0.414838
H	-1.607459	6.840727	1.667099
H	-1.121546	6.477093	-0.747405
C	2.067284	3.158546	-0.049235
C	5.159093	-2.114212	-1.072126
C	5.728871	-2.067353	0.198274
C	2.604735	2.070487	-0.744632
H	3.576312	-1.446218	-2.356425
C	-1.532764	5.826042	1.270362
C	4.000843	-1.388368	-1.351617
C	-1.259467	5.622698	-0.081151
H	1.201744	3.017156	0.600633
C	5.142458	-1.279407	1.190120
H	5.591311	-1.227509	2.184760
H	-1.946990	4.883984	3.170132
C	-1.720880	4.729522	2.112755
C	3.398548	-0.602570	-0.361201
H	2.041144	0.576975	-3.022536
C	3.988714	-0.553149	0.910695
C	-1.163439	4.328452	-0.591331
H	-0.947629	4.195146	-1.654023
H	0.218220	2.354301	-2.039599
H	3.534344	0.064092	1.690832
H	0.335873	1.326691	-4.567784
P	1.870697	0.382833	-0.603254
C	-1.625431	3.435979	1.606807
C	1.376525	0.014874	-2.346027
C	-1.338468	3.222639	0.250676
H	-1.768696	2.577865	2.269851
H	1.574692	-1.053474	-2.534256
H	0.232518	-0.438703	-4.722280
C	-0.608152	1.624778	-2.066386
C	-0.201039	0.434651	-4.213862
H	-1.371320	2.080073	-2.715043
C	-0.102493	0.298088	-2.693293
H	-1.248003	0.523218	-4.540066
P	-1.241471	1.490989	-0.341060
Ru	0.023068	0.067323	0.886525
H	-3.250973	1.603027	-2.614661
H	3.096620	-4.576172	0.168498
H	1.815146	-2.495064	0.536621
C	2.139617	-4.435584	-0.340275
C	1.418764	-3.256938	-0.134405
C	-0.991205	-0.890527	-2.277788
C	-3.751206	1.264716	-1.704043
H	-0.817029	-1.736689	-2.961540
C	-3.039874	1.097650	-0.507415
H	2.207670	-6.342902	-1.349814
C	1.644569	-5.420926	-1.189608
H	-2.041859	-0.596684	-2.413080
P	-0.790837	-1.514825	-0.538555
C	0.195351	-3.054105	-0.780378
H	-5.653402	1.130619	-2.706488
C	-5.119333	0.999018	-1.762855
C	0.419261	-5.230467	-1.832973
C	-0.302114	-4.058912	-1.627288
C	-3.740442	0.674406	0.631171
H	0.020236	-6.003654	-2.493011
H	-3.201539	0.519057	1.569423
H	-1.271780	-3.932810	-2.119950
C	-5.802159	0.579137	-0.623393
C	-2.438560	-2.232775	-0.148654

C	-5.108083	0.422101	0.575464
H	-1.814418	-2.682578	1.874525
H	-3.384352	-1.967907	-2.091151
H	-6.873875	0.373894	-0.670133
C	-2.628133	-2.736291	1.146212
C	-3.490226	-2.332725	-1.067425
H	-5.631814	0.087254	1.473666
C	-3.841059	-3.305617	1.520918
C	-4.708498	-2.898134	-0.689681
H	-3.968022	-3.692571	2.534248
H	-5.521453	-2.956504	-1.417026
C	-4.889984	-3.380919	0.603708
H	-5.845167	-3.821871	0.896579
O	0.692963	1.577972	2.345079
C	1.457171	1.652764	3.284735
H	1.544506	2.595386	3.863197
H	2.095382	0.798230	3.601815
O	-0.424857	-1.551163	3.693747
O	1.242110	-1.240081	2.245031
C	0.791281	-1.768953	3.268341
H	-1.311687	-0.157553	1.878870
H	-0.865257	-0.944564	3.003272
C	1.593893	-2.719267	4.079401
H	2.662869	-2.583485	3.888572
H	1.368573	-2.628275	5.147359
H	1.323888	-3.742276	3.779686

Xa

H	-6.755450	-1.714056	-0.728493
H	-5.532083	-1.261462	-2.850685
C	-5.781252	-1.221154	-0.706003
C	-5.096076	-0.969861	-1.892621
H	-5.759586	-1.001841	1.443209
C	-5.222163	-0.824258	0.509103
C	-3.853483	-0.335143	-1.866402
H	-3.347096	-0.153199	-2.817442
H	-2.853221	5.708271	-0.741225
H	-2.988567	5.950700	1.731053
H	-3.449025	-5.777263	1.554338
H	-4.252873	-4.367865	-0.337766
C	-3.982080	-0.191813	0.537317
C	-2.655994	4.849643	-0.095634
C	-2.731406	4.985226	1.290072
C	-3.273533	0.053391	-0.650524
H	-2.293063	3.529390	-1.753781
C	-2.791699	-5.025928	1.112009
C	-2.335021	3.618134	-0.664949
C	-3.241714	-4.238099	0.054310
H	-3.560770	0.121055	1.495610
C	-2.480651	3.886036	2.111528
H	-2.539969	3.987798	3.197375
H	-1.132455	-5.475172	2.423815
C	-1.494773	-4.856014	1.600652
C	-2.073302	2.512780	0.152975
H	-1.952552	1.198930	-2.908972
C	-2.146750	2.657759	1.546664
C	-2.405938	-3.275515	-0.513177
H	-2.790077	-2.665288	-1.332659
H	-1.505746	-1.503422	-2.453376
H	-1.928278	1.800130	2.194326
H	-1.038456	-0.204239	-4.680791
P	-1.626574	0.864521	-0.502475
C	-0.655460	-3.900834	1.034149
C	-1.083209	1.142878	-2.237848
C	-1.102473	-3.097319	-0.031007
H	0.362390	-3.779434	1.418497
H	-0.623902	2.143541	-2.275077
H	0.088399	1.166541	-4.652211
C	-0.425623	-1.339177	-2.317016
C	-0.074407	0.144003	-4.281520
H	0.075380	-2.059658	-2.982180
C	-0.059996	0.102541	-2.753712
H	0.712184	-0.494834	-4.708277
P	0.016356	-1.756321	-0.573448
Ru	-0.013365	-0.052910	0.835362
H	1.088093	-3.848483	-2.395708
H	0.637678	5.136679	1.922378
H	0.868556	2.682054	1.665523
C	0.886132	4.716060	0.945515
C	1.020558	3.337950	0.799751
C	1.365580	0.458833	-2.277915
C	1.868680	-3.584469	-1.675699
H	1.796659	1.245289	-2.916866
C	1.636378	-2.608767	-0.693042
H	0.943831	6.633756	-0.046401
C	1.056433	5.553075	-0.156708
H	2.011635	-0.422267	-2.419967
P	1.530506	0.954996	-0.510497
C	1.336699	2.779004	-0.447411
H	3.266757	-5.000956	-2.499176
C	3.092472	-4.245678	-1.729912
C	1.372967	5.008070	-1.400404
C	1.520896	3.629048	-1.545104
C	2.635868	-2.329926	0.246245
H	1.514860	5.660535	-2.264612
H	2.457790	-1.569278	1.012066
H	1.786055	3.227580	-2.526989
C	4.090346	-3.951314	-0.797756
C	3.320085	0.766414	-0.163647

C	3.858362	-3.000456	0.192897
H	3.058606	1.431079	1.880173
H	3.938739	0.067563	-2.131749
H	5.049088	-4.472252	-0.844251
C	3.770818	1.094385	1.125054
C	4.247941	0.326174	-1.117075
H	4.634416	-2.765143	0.924915
C	5.117978	0.967235	1.451900
C	5.596969	0.206285	-0.785544
H	5.455614	1.222897	2.458647
H	6.308617	-0.134944	-1.540463
C	6.034450	0.520513	0.499180
H	7.091361	0.424722	0.757007
O	1.297405	-0.121208	2.416551
C	1.408303	-1.015234	3.419199
H	1.571675	-2.054187	3.059970
O	0.226790	-1.135034	4.245797
H	-0.979642	-1.100127	3.126079
H	2.255955	-0.757389	4.086971
O	-1.497450	-0.886620	2.299603
H	0.184989	-0.355579	4.815309
H	-1.932206	-1.704043	2.019302

Xa-XIa

H	-6.485404	2.250698	-0.668569
H	-5.239234	1.871298	-2.791207
C	-5.399118	2.140355	-0.655306
C	-4.702448	1.924549	-1.841264
H	-5.233067	2.428931	1.479711
C	-4.698349	2.235677	0.547315
C	-3.314491	1.781232	-1.826332
H	-2.801929	1.617450	-2.777612
H	0.956005	6.313024	-0.751310
H	0.948410	6.614135	1.718270
H	-6.338825	-2.311688	1.264423
H	-5.979688	-1.401364	-1.025944
C	-3.312845	2.101813	0.562856
C	0.623170	5.500406	-0.101831
C	0.618615	5.668547	1.282454
C	-2.599911	1.855236	-0.623240
H	0.198417	4.190043	-1.752768
C	-5.325438	-2.154177	0.889473
C	0.198387	4.298008	-0.664968
C	-5.124986	-1.643467	-0.390298
H	-2.773339	2.215354	1.506920
C	0.189920	4.629588	2.108631
H	0.183285	4.758645	3.193216
H	-4.371215	-2.887394	2.688224
C	-4.225471	-2.475343	1.687518
C	-0.224757	3.248696	0.159584
H	-0.870673	2.061918	-2.892794
C	-0.224094	3.421422	1.552779
C	-3.831569	-1.435415	-0.871407
H	-3.711354	-1.037946	-1.880651
H	-1.987030	-0.418514	-2.499952
H	-0.543418	2.596303	2.201171
H	-0.885806	0.401426	-4.664314
P	-0.777516	1.632880	-0.488531
C	-2.936169	-2.271957	1.208467
C	-0.172711	1.537777	-2.223221
C	-2.721821	-1.737522	-0.073595
H	-2.082521	-2.523078	1.844225
H	0.763027	2.117520	-2.267693
H	0.817010	0.900826	-4.637280
C	-1.009456	-0.881266	-2.303320
C	0.107611	0.146861	-4.266154
H	-0.966741	-1.784193	-2.933312
C	0.100084	0.102343	-2.737950
H	0.398311	-0.823401	-4.693747
P	-0.975660	-1.432158	-0.535552
Ru	0.011929	-0.022898	0.850881
H	-1.826520	-3.853010	-1.966722
H	3.477014	3.896456	1.912844
H	2.284762	1.742062	1.661648
C	3.455854	3.400947	0.939774
C	2.791160	2.186185	0.795415
C	1.480159	-0.405045	-2.267708
C	-0.919343	-4.101870	-1.406259
H	2.277228	-0.000819	-2.911163
C	-0.287908	-3.130885	-0.612304
H	4.594877	4.943831	-0.053681
C	4.079726	3.986881	-0.161208
H	1.514552	-1.498561	-2.404904
P	1.873987	-0.082884	-0.499207
C	2.749791	1.533411	-0.445437
H	-0.901573	-6.143167	-2.092373
C	-0.405292	-5.393165	-1.472907
C	4.046294	3.347481	-1.399667
C	3.392873	2.123382	-1.540518
C	0.846968	-3.480770	0.125162
H	4.539751	3.799220	-2.262914
H	1.331880	-2.731948	0.754624
H	3.396029	1.634139	-2.518450
C	0.737843	-5.731307	-0.744135
C	3.262510	-1.231326	-0.145499

C	1.360330	-4.777021	0.056577
H	3.335939	-0.608472	1.928288
H	3.479951	-2.067347	-2.142830
H	1.137808	-6.745998	-0.798837
C	3.773413	-1.252036	1.161549
C	3.840548	-2.064261	-1.112295
H	2.251834	-5.036088	0.632673
C	4.827949	-2.097947	1.494142
C	4.898155	-2.909031	-0.775262
H	5.215746	-2.103278	2.515134
H	5.339793	-3.550734	-1.540715
C	5.390946	-2.932088	0.527453
H	6.218985	-3.594286	0.788893
O	0.963024	-1.053859	2.487299
C	0.528086	-1.991583	3.201850
H	-0.007578	-2.845435	2.741542
O	-1.001377	-1.434984	4.091314
H	-1.299208	-0.687615	3.383832
H	1.093795	-2.266478	4.111346
O	-1.448642	0.242020	2.333400
H	-0.718627	-0.942271	4.873755
H	-2.357043	0.284439	2.009964

XIa

H	-2.285247	6.591626	0.353139
H	-3.677345	5.182033	1.860656
C	-2.163355	5.506454	0.352281
C	-2.941564	4.716671	1.201439
H	-0.630359	5.515532	-1.171540
C	-1.240297	4.906661	-0.500411
C	-2.788909	3.333265	1.204985
H	-3.426858	2.730465	1.858701
H	-6.430281	-0.595882	0.642378
H	-6.729805	-0.680058	-1.826609
H	3.244515	6.161724	-1.017475
H	2.746548	5.700602	1.378477
C	-1.085706	3.520215	-0.495700
C	-5.600419	-0.337887	-0.019268
C	-5.767734	-0.384615	-1.402402
C	-1.845878	2.721023	0.364230
H	-4.271180	0.075690	1.615644
C	2.885698	5.176480	-0.712174
C	-4.375045	0.040479	0.528101
C	2.605831	4.919565	0.628306
H	-0.352423	3.052449	-1.154753
C	-4.705066	-0.049980	-2.242316
H	-4.831122	-0.083122	-3.326730
H	2.939068	4.360085	-2.711640
C	2.713028	4.167445	-1.660441
C	-3.301740	0.368384	-0.310164
H	-2.167013	1.196743	2.679654
C	-3.477293	0.321754	-1.701190
C	2.148335	3.662197	1.021154
H	1.948697	3.485769	2.080453
H	0.246313	2.167951	2.268111
H	-2.634016	0.554906	-2.360711
H	-0.646127	1.257238	4.549079
P	-1.664161	0.892750	0.318313
C	2.251855	2.910752	-1.275658
C	-1.668971	0.401069	2.104205
C	1.956895	2.650819	0.070665
H	2.107351	2.134313	-2.033246
H	-2.299110	-0.496770	2.206926
H	-1.203608	-0.426484	4.622898
C	0.737771	1.180643	2.252250
C	-0.407397	0.228977	4.241281
H	1.570352	1.256200	2.967972
C	-0.280154	0.118517	2.721746
H	0.528404	-0.060708	4.741589
P	1.409308	0.964087	0.550381
Ru	0.014004	-0.048443	-0.859522
H	3.185415	0.108466	2.906971
H	-4.036354	-3.292821	-1.833262
H	-1.853808	-2.157001	-1.560774
C	-3.547719	-3.289084	-0.856490
C	-2.318274	-2.654019	-0.699993
C	0.194996	-1.314948	2.391086
C	3.671146	-0.125508	1.955869
H	-0.308569	-2.040374	3.047796
C	3.047014	0.158233	0.735124
H	-5.125558	-4.392399	0.121773
C	-4.155964	-3.903263	0.237478
H	1.267459	-1.406059	2.625227
P	-0.041979	-1.790693	0.630548
C	-1.671157	-2.636459	0.545139
H	5.410078	-0.937495	2.938316
C	4.933094	-0.721601	1.979732
C	-3.522487	-3.894848	1.479792
C	-2.282902	-3.274501	1.631837
C	3.721167	-0.137622	-0.459693
H	-3.991315	-4.382870	2.336931
H	3.264085	0.127077	-1.420923
H	-1.797860	-3.301203	2.611053
C	5.585072	-1.033136	0.788190
C	1.138094	-3.157404	0.303298

C	4.978864	-0.732970	-0.432796
H	-0.184541	-4.108644	-1.130433
H	2.708435	-2.484257	1.645783
H	6.573269	-1.497385	0.810463
C	0.801224	-4.123673	-0.658546
C	2.408642	-3.212520	0.891241
H	5.494888	-0.954268	-1.370112
C	1.713687	-5.109582	-1.024971
C	3.320330	-4.201200	0.523189
H	1.430008	-5.857085	-1.769146
H	4.303269	-4.227290	0.999228
C	2.979012	-5.149468	-0.438677
H	3.692807	-5.924952	-0.724246
O	0.902245	-1.434703	-2.327599
C	1.891788	-1.771582	-2.943676
H	2.920464	-1.584819	-2.576128
O	2.201713	0.520065	-3.779383
H	1.272403	0.753147	-3.523503
H	1.796377	-2.363219	-3.877766
O	-0.206415	0.969793	-2.617550
H	2.356882	0.971212	-4.615653
H	-0.681676	1.807635	-2.646291

XIIa

H	-2.858430	6.329937	0.301213
H	-4.007560	4.829654	1.922024
C	-2.644031	5.259204	0.300518
C	-3.285511	4.418993	1.213132
H	-1.240111	5.374343	-1.338973
C	-1.740395	4.726798	-0.615406
C	-3.014238	3.053907	1.217582
H	-3.547478	2.409285	1.923100
H	-6.296061	-1.186789	1.076891
H	-6.825179	-1.365174	-1.349131
H	2.632619	6.353836	-1.511554
H	2.601946	5.900691	0.935369
C	-1.466526	3.358818	-0.611148
C	-5.560446	-0.877888	0.331033
C	-5.856480	-0.977139	-1.027075
C	-2.087287	2.511945	0.313258
H	-4.126854	-0.303620	1.819043
C	2.387373	5.355170	-1.143906
C	-4.326361	-0.379478	0.747422
C	2.369919	5.102356	0.227311
H	-0.755729	2.943654	-1.328282
C	-4.911621	-0.577329	-1.972193
H	-5.134998	-0.653435	-3.038611
H	2.102518	4.525813	-3.119222
C	2.092065	4.331276	-2.044589
C	-3.368469	0.014858	-0.196454
H	-2.125764	1.000605	2.663203
C	-3.674258	-0.089915	-1.560978
C	2.061389	3.827987	0.700463
H	2.068372	3.644722	1.778440
H	0.130870	2.228803	2.079857
H	-2.920915	0.177929	-2.307566
H	-0.519527	1.219103	4.459743
P	-1.738040	0.707991	0.280291
C	1.772064	3.058616	-1.576415
C	-1.575834	0.254213	2.069224
C	1.751680	2.799702	-0.198991
H	1.494217	2.267586	-2.282835
H	-2.094938	-0.705520	2.221200
H	-0.882736	-0.516132	4.550999
C	0.734709	1.305212	2.089081
C	-0.186779	0.224141	4.130608
H	1.561948	1.505629	2.786196
C	-0.133819	0.133457	2.605384
H	0.802309	0.039170	4.575406
P	1.396395	1.098700	0.384932
Ru	0.008707	0.011613	-0.996405
H	3.174154	0.342053	2.774745
H	-3.746975	-3.615135	-1.776322
H	-1.702688	-2.226145	-1.604655
C	-3.190093	-3.587641	-0.837176
C	-2.036399	-2.813088	-0.739258
C	0.466844	-1.243453	2.235435
C	3.723129	0.216720	1.837819
H	0.052315	-2.017216	2.898919
C	3.119053	0.489730	0.603647
H	-4.548007	-4.908130	0.199636
C	-3.637153	-4.309486	0.268045
H	1.548679	-1.236279	2.437320
P	0.217171	-1.727283	0.474069
C	-1.306042	-2.758456	0.456498
H	5.503042	-0.427837	2.868854
C	5.046207	-0.223860	1.897935
C	-2.916438	-4.269167	1.461698
C	-1.752269	-3.507945	1.553563
C	3.881643	0.338475	-0.564786
H	-3.257244	-4.842014	2.326771
H	3.438737	0.597353	-1.532106
H	-1.193559	-3.514103	2.493021
C	5.784288	-0.393530	0.728518
C	1.519745	-2.964188	0.098310

C	5.199880	-0.104103	-0.505481
H	0.284427	-3.925797	-1.405918
H	3.026786	-2.252178	1.493427
H	6.820391	-0.734557	0.778648
C	1.271303	-3.884326	-0.935529
C	2.791714	-2.946746	0.685844
H	5.780226	-0.210315	-1.424883
C	2.269623	-4.753295	-1.371791
C	3.789954	-3.817006	0.246380
H	2.053561	-5.466641	-2.170318
H	4.772822	-3.788626	0.722527
C	3.535704	-4.718215	-0.786052
H	4.317841	-5.399972	-1.126207
O	1.338320	-0.946993	-2.525532
C	2.303702	-1.665467	-2.673731
H	3.056698	-1.844924	-1.872276
H	2.482184	-2.164652	-3.650859
O	-0.628111	0.811727	-2.691917
H	-1.328827	1.475923	-2.701294

XIIIa

H	-2.429563	6.395508	1.103717
H	-3.949773	4.774520	2.227612
C	-2.286439	5.322555	0.959109
C	-3.138179	4.414010	1.592139
H	-0.592726	5.560540	-0.363184
C	-1.261139	4.857621	0.139662
C	-2.957684	3.046225	1.412283
H	-3.638850	2.343726	1.903457
H	-5.781974	-2.125681	0.616028
H	-6.894165	-0.916350	-1.257539
H	3.501348	6.195486	-1.174825
H	2.988978	5.715595	1.213908
C	-1.080511	3.485771	-0.046588
C	-5.286661	-1.278457	0.136631
C	-5.907674	-0.602534	-0.910361
C	-1.919225	2.569504	0.594820
H	-3.560491	-1.441151	1.396852
C	3.132144	5.210736	-0.880938
C	-4.025554	-0.881264	0.582912
C	2.843721	4.942611	0.456167
H	-0.284373	3.132284	-0.702099
C	-5.266095	0.481084	-1.513126
H	-5.748633	1.018268	-2.332127
H	3.185010	4.416372	-2.889461
C	2.954375	4.214617	-1.841275
C	-3.375580	0.206534	-0.011899
H	-1.867641	0.945231	2.822085
C	-4.010673	0.882008	-1.068549
C	2.372962	3.686148	0.835179
H	2.158804	3.500938	1.890316
H	0.389818	2.116260	2.064113
H	-3.517351	1.738474	-1.538949
H	-0.103460	0.987963	4.490727
P	-1.687317	0.757592	0.418206
C	2.480002	2.960311	-1.465779
C	-1.395860	0.205082	2.156438
C	2.180096	2.683529	-0.124487
H	2.332084	2.188978	-2.227017
H	-1.934719	-0.741044	2.322542
H	-0.521172	-0.736833	4.508030
C	0.966690	1.176788	2.028161
C	0.164316	0.006186	4.074465
H	1.816591	1.336036	2.708501
C	0.085599	0.011187	2.547290
H	1.179986	-0.237405	4.419814
P	1.569725	1.004376	0.293491
Ru	-0.060111	0.012160	-1.048086
H	3.447828	0.271195	2.580300
H	-3.476908	-4.443831	-1.663340
H	-1.559516	-2.877695	-1.698952
C	-2.991761	-4.163956	-0.725669
C	-1.902421	-3.296353	-0.749087
C	0.617513	-1.349513	2.044949
C	3.860678	-0.096352	1.637595
H	0.298811	-2.154406	2.722169
C	3.180312	0.099984	0.427586
H	-4.320566	-5.346835	0.502563
C	-3.465146	-4.667946	0.485216
H	1.714013	-1.339263	2.111606
P	0.190060	-1.798853	0.309174
C	-1.264071	-2.919885	0.444320
H	5.589034	-0.918294	2.626445
C	5.080718	-0.771971	1.670847
C	-2.835829	-4.305748	1.674699
C	-1.739864	-3.442879	1.655846
C	3.774440	-0.379146	-0.748436
H	-3.192028	-4.702605	2.627899
H	3.281681	-0.235575	-1.714189
H	-1.269625	-3.189800	2.608547
C	5.652659	-1.248207	0.493306
C	1.521832	-2.945169	-0.216945

C	4.998324	-1.041683	-0.720056
H	0.746628	-3.029398	-2.241462
H	2.535314	-3.126438	1.700694
H	6.608422	-1.775693	0.520366
C	1.527448	-3.371778	-1.553784
C	2.514665	-3.419793	0.649141
H	5.436896	-1.406795	-1.651216
C	2.513852	-4.237240	-2.017619
C	3.505087	-4.282242	0.179721
H	2.502245	-4.562522	-3.059994
H	4.277982	-4.636189	0.865661
C	3.510870	-4.689078	-1.152222
H	4.287604	-5.364818	-1.516195
O	-0.246886	1.773598	-2.510358
C	-0.939188	1.747068	-3.509462
H	-0.940130	2.623087	-4.193262
H	-1.563679	0.852158	-3.724445
O	-1.476400	-0.725982	-2.377955
H	1.375037	-0.291062	-2.117657
H	0.823981	-0.868277	-2.364346
H	-2.319083	-0.917522	-1.947696

XIIIa-XIVa

H	-0.298927	-6.938669	1.031836
H	1.772024	-6.019706	2.068451
C	-0.037896	-5.887443	0.892816
C	1.122254	-5.372579	1.475578
H	-1.759455	-5.455741	-0.337444
C	-0.854148	-5.058617	0.128083
C	1.458317	-4.034774	1.295799
H	2.379681	-3.647642	1.742989
H	6.100547	-1.364452	0.901580
H	6.502088	-1.938105	-1.487637
H	-5.917918	-3.856277	-1.385956
H	-4.723204	-4.327740	0.745675
C	-0.517384	-3.715754	-0.053746
C	5.256808	-1.518878	0.225231
C	5.481330	-1.839354	-1.112288
C	0.635337	-3.188453	0.534116
H	3.809537	-1.132204	1.761130
C	-5.150555	-3.168732	-1.024681
C	3.955854	-1.388444	0.708899
C	-4.480584	-3.432928	0.168108
H	-1.157019	-3.078044	-0.663125
C	4.398826	-2.041302	-1.969157
H	4.569294	-2.303753	-3.015387
H	-5.388286	-1.787323	-2.670649
C	-4.849491	-2.012597	-1.747358
C	2.864342	-1.576665	-0.147083
H	1.225114	-1.729264	2.755501
C	3.099068	-1.911377	-1.489156
C	-3.499190	-2.557619	0.635994
H	-2.982383	-2.793758	1.569162
H	-1.301355	-1.719585	2.171334
H	2.249290	-2.081537	-2.157982
H	-0.243721	-0.926295	4.513156
P	1.112839	-1.420597	0.347319
C	-3.875834	-1.134330	-1.278500
C	1.147131	-0.840985	2.108846
C	-3.180818	-1.402509	-0.086855
H	-3.666664	-0.208615	-1.826886
H	2.069206	-0.261809	2.267595
H	0.919995	0.412726	4.467137
C	-1.391823	-0.622633	2.103890
C	-0.050676	0.066705	4.082334
H	-2.198787	-0.339442	2.797260
C	-0.055071	0.018622	2.554031
H	-0.823452	0.751365	4.461135
P	-1.886111	-0.194775	0.382638
Ru	-0.114290	-0.024389	-1.043698
H	-4.021062	0.545968	2.297266
H	5.225361	1.669150	-1.638893
H	2.765720	1.364728	-1.679156
C	4.653841	1.783016	-0.714812
C	3.270082	1.625387	-0.743691
C	0.077022	1.471881	2.035073
C	-3.887314	1.381652	1.603912
H	0.726964	2.051275	2.706677
C	-2.930070	1.312846	0.578099
H	6.394320	2.190444	0.500945
C	5.308803	2.069812	0.481676
H	-0.905005	1.969050	2.085144
P	0.685619	1.648229	0.304678
C	2.519291	1.763322	0.434720
H	-5.437235	2.544946	2.541975
C	-4.698414	2.504506	1.738795
C	4.571118	2.201664	1.656764
C	3.184227	2.057408	1.634291
C	-2.831363	2.377031	-0.323981
H	5.073655	2.429834	2.599344
H	-2.103317	2.333715	-1.137200
H	2.637319	2.182533	2.571974
C	-4.576287	3.570063	0.844158
C	0.255301	3.359991	-0.173139

C	-3.648987	3.500125	-0.191612
H	0.798537	2.984672	-2.238445
H	-0.334060	4.064382	1.797586
H	-5.213623	4.450127	0.952972
C	0.426502	3.720656	-1.517760
C	-0.199741	4.315751	0.742759
H	-3.548985	4.324850	-0.900961
C	0.130327	5.014209	-1.938933
C	-0.496495	5.609560	0.315189
H	0.268217	5.287211	-2.987272
H	-0.855224	6.347279	1.036183
C	-0.336825	5.959573	-1.024179
H	-0.569994	6.973511	-1.356098
O	-0.766019	-1.643242	-2.421660
C	-1.559868	-1.626843	-3.341648
H	-1.765795	-2.548487	-3.924073
H	-2.093484	-0.699513	-3.646347
O	1.224206	0.520143	-2.739120
H	-0.952217	1.068609	-2.248663
H	-0.109771	0.925977	-2.648955
H	1.320441	-0.255128	-3.306924

XIVa

H	-4.819768	5.160287	0.987430
H	-5.002793	3.396669	2.736021
C	-4.242658	4.237660	0.896068
C	-4.342248	3.251107	1.878553
H	-3.335516	4.798864	-0.981153
C	-3.413282	4.034438	-0.204216
C	-3.612004	2.070926	1.762398
H	-3.735951	1.298440	2.526635
H	-5.055591	-3.575594	0.692889
H	-6.096312	-3.030067	-1.504014
H	1.137032	6.833759	-1.429499
H	-0.564864	6.039721	0.209607
C	-2.672037	2.857508	-0.315124
C	-4.713382	-2.708194	0.123559
C	-5.296471	-2.403925	-1.103972
C	-2.754621	1.865740	0.670416
H	-3.261499	-2.179680	1.607697
C	1.172277	5.808802	-1.054474
C	-3.690265	-1.912161	0.640437
C	0.220098	5.364316	-0.138603
H	-2.011607	2.709748	-1.172045
C	-4.862996	-1.281757	-1.811469
H	-5.331417	-1.017762	-2.762472
H	2.942694	5.287145	-2.179507
C	2.180870	4.941751	-1.476782
C	-3.230042	-0.796330	-0.066501
H	-2.009234	0.323631	2.864005
C	-3.840619	-0.486171	-1.297060
C	0.263852	4.056623	0.345169
H	-0.499508	3.735984	1.057373
H	-0.160532	2.084040	2.207855
H	-3.528162	0.413667	-1.842108
H	-0.349629	0.746024	4.532181
P	-1.827705	0.288343	0.427795
C	2.230159	3.636154	-0.989432
C	-1.381540	-0.229284	2.149303
C	1.267516	3.174803	-0.075216
H	3.036874	2.969543	-1.310953
H	-1.650944	-1.287964	2.276168
H	-0.295612	-1.026481	4.460110
C	0.640214	1.333902	2.105047
C	0.171729	-0.107642	4.075238
H	1.439016	1.656486	2.789501
C	0.102993	-0.048142	2.547864
H	1.213120	-0.090328	4.428321
P	1.280023	1.396061	0.371805
Ru	0.076452	0.118026	-1.027104
H	3.306563	2.337813	2.333218
H	-2.040881	-5.141022	-1.915923
H	-0.515172	-3.199343	-1.854651
C	-1.728006	-4.729779	-0.953494
C	-0.873402	-3.631466	-0.917997
C	0.994136	-1.187315	1.999591
C	3.805382	1.677466	1.617873
H	0.902855	-2.077071	2.639537
C	3.085246	1.086407	0.566515
H	-2.837587	-6.178372	0.206526
C	-2.171836	-5.313011	0.233635
H	2.052133	-0.885740	2.079070
P	0.716947	-1.655293	0.235665
C	-0.454684	-3.083724	0.305714
H	5.719818	1.918205	2.574480
C	5.172650	1.452855	1.751788
C	-1.746671	-4.793920	1.453826
C	-0.894338	-3.689415	1.490544
C	3.773101	0.298948	-0.362809
H	-2.073321	-5.253224	2.389729
H	3.222424	-0.137620	-1.199195
H	-0.578726	-3.314619	2.467294
C	5.843378	0.642068	0.833828
C	2.252490	-2.554542	-0.218828

C	5.143668	0.074362	-0.227806
H	1.922915	-2.257402	-2.331990
H	2.861355	-3.033170	1.812193
H	6.915103	0.463340	0.944523
C	2.563602	-2.720291	-1.574606
C	3.087266	-3.130122	0.747354
H	5.660998	-0.553162	-0.957367
C	3.695344	-3.436253	-1.956616
C	4.226069	-3.836858	0.362839
H	3.928704	-3.557487	-3.016779
H	4.875181	-4.271983	1.125742
C	4.533001	-3.990554	-0.987981
H	5.424421	-4.546195	-1.286874
O	-0.412246	1.810368	-2.412269
C	0.389586	2.427489	-3.089092
H	0.064912	3.306989	-3.684528
H	1.456274	2.124340	-3.148958
O	-1.071733	-1.066332	-2.619483
H	1.380988	-0.077993	-2.016360
H	-1.098587	-0.602226	-3.465336
H	-2.002353	-1.227097	-2.383641

XV

H	6.520345	-2.376413	0.075584
H	5.703979	-1.320827	-2.026702
C	5.449806	-2.235357	-0.088240
C	4.993792	-1.646909	-1.263674
H	4.878890	-3.117126	1.801760
C	4.530496	-2.646717	0.879445
C	3.624455	-1.474294	-1.477542
H	3.301231	-1.022675	-2.418462
H	1.201719	-6.526365	-1.460915
H	-1.046224	-7.029156	-0.515518
H	6.949382	1.934742	0.761911
H	5.909625	3.127557	-1.162266
C	3.169002	-2.454835	0.673676
C	0.584579	-5.720518	-1.057890
C	-0.675596	-6.001942	-0.528803
C	2.694431	-1.868418	-0.510089
H	2.055768	-4.208181	-1.484948
C	5.881758	1.822213	0.560818
C	1.063320	-4.412332	-1.072942
C	5.300487	2.488107	-0.519678
H	2.457382	-2.763163	1.446096
C	-1.457827	-4.967786	-0.017862
H	-2.449062	-5.177981	0.392884
H	5.544760	0.491071	2.227167
C	5.094865	1.018335	1.382425
C	0.283002	-3.364474	-0.558912
H	1.464354	-1.599678	-3.015577
C	-0.980595	-3.657643	-0.031878
C	3.939612	2.345869	-0.775878
H	3.493494	2.889816	-1.614512
H	2.341018	0.886531	-2.456001
H	-1.594203	-2.849410	0.366305
H	1.496678	0.159788	-4.639564
P	0.875319	-1.624163	-0.651107
C	3.731394	0.871954	1.124408
C	0.601314	-1.229185	-2.440392
C	3.142742	1.527428	0.040132
H	3.114730	0.223332	1.754455
H	-0.239734	-1.854950	-2.777576
H	-0.189918	-0.347804	-4.851446
C	1.326921	1.208686	-2.171946
C	0.462627	0.363727	-4.325126
H	1.208310	2.206530	-2.623033
C	0.308100	0.240134	-2.804768
H	0.202995	1.373327	-4.675465
P	1.341317	1.395388	-0.318826
Ru	0.053138	-0.092164	0.779281
H	-0.412257	3.335180	-1.810035
H	-4.028093	-3.659073	-2.283936
H	-2.155361	-2.070610	-2.154058
C	-4.144150	-2.739753	-1.705204
C	-3.087319	-1.833844	-1.633134
C	-1.150002	0.623341	-2.480897
C	-0.036024	3.820955	-0.906530
H	-1.819351	0.019484	-3.114852
C	0.840102	3.145629	-0.048393
H	-6.171829	-3.176997	-1.101086
C	-5.340512	-2.471392	-1.042345
H	-1.310994	1.663851	-2.806031
P	-1.764720	0.499195	-0.719463
C	-3.202345	-0.645486	-0.899536
H	-1.133678	5.628760	-1.312708
C	-0.452197	5.121459	-0.626129
C	-5.472778	-1.288435	-0.315036
C	-4.415868	-0.383275	-0.244161
C	1.302328	3.815973	1.096625
H	-6.411604	-1.061916	0.195405
H	2.044272	3.336703	1.743156
H	-4.543351	0.541361	0.325426
C	-0.007831	5.765273	0.526484
C	-2.685603	2.083265	-0.520438

C	0.874081	5.109924	1.386688
H	-2.094302	2.349126	1.537663
H	-3.499132	2.090592	-2.534336
H	-0.334717	6.783470	0.747619
C	-2.681645	2.749330	0.709557
C	-3.447084	2.612496	-1.573984
H	1.251720	5.619704	2.276106
C	-3.408851	3.927680	0.881292
C	-4.164106	3.794408	-1.405759
H	-3.391782	4.439823	1.846209
H	-4.751337	4.195403	-2.234946
C	-4.144672	4.456517	-0.176501
H	-4.710613	5.381357	-0.044533
O	-0.477122	1.259088	2.490001
C	0.286936	1.769500	3.282680
H	1.388776	1.638636	3.201633
H	1.300475	-0.531170	1.748746
H	-0.098257	2.373866	4.131090
C	-2.543333	-1.323000	2.461137
O	-1.142979	-1.429324	2.148898
C	-0.482546	-2.311322	3.076590
C	-1.602746	-2.977533	3.843545
C	-2.668945	-1.891870	3.855655
H	-2.840132	-0.267673	2.364084
H	-3.120014	-1.911889	1.723087
H	0.149385	-3.005567	2.501333
H	0.176772	-1.705560	3.723682
H	-1.963905	-3.864683	3.299402
H	-1.294743	-3.308851	4.843165
H	-3.678233	-2.263501	4.072951
H	-2.430680	-1.123298	4.608042

IXc

```
H       6.301071   -2.769938   -0.986229
H       4.892777   -2.899601   -3.039350
C       5.232425   -2.551466   -0.934405
C       4.445483   -2.619552   -2.083214
H       5.245770   -2.147741    1.188652
C       4.643675   -2.206446    0.279331
C       3.081086   -2.343692   -2.018590
H       2.494019   -2.427264   -2.935773
H      -2.067167   -5.726507   -1.814991
H      -1.019200   -7.124081   -0.037625
H       6.875242    1.193449    1.571881
H       6.414265    1.215324   -0.876573
C       3.282036   -1.915233    0.337446
C      -1.298049   -5.303077   -1.164787
C      -0.711451   -6.085203   -0.172631
C       2.476496   -1.972548   -0.807572
H      -1.382812   -3.393820   -2.132506
C       5.845953    1.218420    1.207499
C      -0.903755   -3.976836   -1.343903
C       5.588426    1.231356   -0.161808
H       2.857462   -1.628899    1.302511
C       0.286529   -5.537923    0.634549
H       0.769910   -6.147992    1.400727
H       4.972928    1.249074    3.184785
C       4.782462    1.246774    2.109425
C       0.084699   -3.409807   -0.528792
H       0.682308   -1.815689   -3.122716
C       0.682434   -4.215590    0.453760
C       4.275538    1.262949   -0.629385
H       4.107519    1.273457   -1.708767
H       2.410651    0.226669   -2.235631
H       1.490817   -3.813214    1.072421
H       1.402369   -0.039339   -4.594078
P       0.653031   -1.661876   -0.684271
C       3.469040    1.275604    1.646294
C       0.148080   -1.191201   -2.391815
C       3.199651    1.274889    0.269645
H       2.655022    1.293607    2.376383
H      -0.911632   -1.469685   -2.502156
H      -0.363702   -0.081433   -4.778905
C       1.568419    0.919506   -2.094967
C       0.462585    0.421858   -4.256033
H       1.834895    1.843389   -2.631946
C       0.307163    0.310253   -2.738138
H       0.472055    1.472550   -4.580160
P       1.450801    1.338551   -0.287408
Ru     -0.013986   -0.005371    0.839671
H       2.881876    3.555806   -1.575990
H      -4.089933   -3.649902   -0.441661
H      -2.267105   -2.076344    0.139155
C      -4.075114   -2.651895   -0.888416
C      -3.045491   -1.767240   -0.561276
C      -0.941786    1.119572   -2.334158
C       2.007249    3.978440   -1.071007
H      -1.731748    0.995917   -3.092652
C       1.137047    3.151503   -0.340832
H      -5.878969   -2.956578   -2.035737
C      -5.071892   -2.266578   -1.780642
H      -0.678864    2.190339   -2.329728
P      -1.676263    0.720899   -0.684093
C      -3.005287   -0.485670   -1.119116
H       2.463663    5.981936   -1.713337
C       1.779327    5.348674   -1.144727
C      -5.038996   -0.989778   -2.345949
C      -4.018368   -0.104180   -2.014064
C       0.053486    3.725267    0.330975
H      -5.820130   -0.678556   -3.042668
H      -0.605768    3.091351    0.927191
H      -4.023189    0.901752   -2.445343
C       0.683548    5.912402   -0.486547
C      -2.694912    2.200853   -0.317023
```

C	-0.172384	5.101158	0.252785
H	-3.037265	1.597343	1.730865
H	-2.543105	3.115277	-2.287660
H	0.506933	6.988600	-0.545480
C	-3.267995	2.328831	0.957088
C	-2.966660	3.180381	-1.283357
H	-1.026066	5.535094	0.778985
C	-4.086554	3.415919	1.254898
C	-3.789476	4.264294	-0.981142
H	-4.519247	3.506677	2.253578
H	-3.991164	5.017088	-1.746176
C	-4.349462	4.386682	0.288848
H	-4.991498	5.237797	0.525568
O	-0.762487	1.373377	2.249411
C	0.091899	1.955712	3.117380
H	0.863214	2.597739	2.632018
O	0.874753	1.028986	3.883015
H	0.278028	0.591096	4.502567
H	1.263544	-0.797096	1.772194
H	1.047743	-0.158594	2.294736
H	-0.475148	2.602010	3.820675
C	-2.642266	-0.988946	2.605841
O	-1.369268	-1.383178	2.067834
C	-0.852249	-2.362995	2.971983
C	-2.047965	-3.250647	3.233632
C	-3.221999	-2.261190	3.225250
H	-2.480981	-0.182720	3.340368
H	-3.232452	-0.577376	1.775535
H	-0.003600	-2.859501	2.485875
H	-0.486919	-1.862702	3.890554
H	-2.141682	-3.981921	2.415287
H	-1.961419	-3.818616	4.168591
H	-4.068673	-2.645764	2.641123
H	-3.602533	-2.064861	4.236592

IXc-XIb

H	5.695925	-3.808347	-0.656193
H	4.556673	-3.484120	-2.847486
C	4.676681	-3.417726	-0.692241
C	4.041316	-3.232342	-1.918072
H	4.494955	-3.239675	1.454115
C	4.004877	-3.100734	0.488067
C	2.740859	-2.729705	-1.965856
H	2.268316	-2.605347	-2.943158
H	-3.911220	-4.127971	0.096063
H	-2.880555	-6.400100	0.144420
H	6.974306	-0.068174	1.311331
H	6.453049	-0.120594	-1.125042
C	2.710885	-2.590745	0.438751
C	-2.837350	-4.249276	-0.067679
C	-2.260769	-5.520050	-0.040215
C	2.060119	-2.392618	-0.787873
H	-2.499993	-2.136444	-0.283844
C	5.959323	0.133914	0.962376
C	-2.044517	-3.127984	-0.293886
C	5.668329	0.105210	-0.399325
H	2.201473	-2.331123	1.371599
C	-0.890596	-5.662708	-0.251329
H	-0.434176	-6.654663	-0.235339
H	5.163761	0.470584	2.945438
C	4.948216	0.431733	1.875561
C	-0.663927	-3.261716	-0.508737
H	0.453134	-1.954522	-3.188786
C	-0.092939	-4.542228	-0.485244
C	4.373310	0.364281	-0.848083
H	4.184672	0.342572	-1.923552
H	2.346557	-0.184356	-2.447217
H	0.979900	-4.673858	-0.645118
H	1.241652	-0.314786	-4.760417
P	0.338304	-1.745900	-0.759642
C	3.652754	0.684051	1.432145
C	-0.049724	-1.264562	-2.494666
C	3.346107	0.646670	0.063313
H	2.885174	0.905538	2.177625
H	-1.120896	-1.479391	-2.620180
H	-0.517370	-0.116329	-4.886880
C	1.622546	0.625134	-2.274784
C	0.386962	0.274848	-4.397288
H	2.006640	1.504499	-2.815045
C	0.267803	0.202564	-2.873829
H	0.528333	1.310654	-4.738193
P	1.624809	1.031826	-0.460940
Ru	-0.019274	-0.045807	0.701925
H	-0.021716	3.074305	0.847436
H	-6.167464	-0.572942	0.388958
H	-4.022934	0.594906	0.744821
C	-5.408554	-0.586862	-0.396717
C	-4.193673	0.061259	-0.193660
C	-0.843848	1.191525	-2.445175
C	0.712060	3.604767	0.240749
H	-1.641091	1.222325	-3.203488
C	1.649065	2.870426	-0.488358
H	-6.616102	-1.742252	-1.769114
C	-5.663444	-1.234268	-1.606254
H	-0.422037	2.211193	-2.422255
P	-1.600267	0.904284	-0.786387
C	-3.201463	0.067842	-1.186279
H	-0.027723	5.557976	0.776523
C	0.716854	4.999494	0.204299
C	-4.703667	-1.204011	-2.614723
C	-3.485388	-0.555107	-2.409141
C	2.618758	3.554504	-1.238529
H	-4.902621	-1.682319	-3.576043
H	3.385732	3.000959	-1.789941
H	-2.767699	-0.535534	-3.232030
C	1.670443	5.670605	-0.556755
C	-2.293638	2.538430	-0.319240

C	2.625932	4.945633	-1.273119
H	-2.642053	3.222919	-2.350954
H	-2.062517	2.159191	1.796977
H	1.678826	6.762181	-0.587674
C	-2.737308	3.449718	-1.285657
C	-2.451072	2.847373	1.038811
H	3.385142	5.468926	-1.858181
C	-3.316091	4.657032	-0.898252
C	-3.039771	4.051467	1.422348
H	-3.654329	5.363537	-1.659210
H	-3.154661	4.285751	2.483248
C	-3.471031	4.958800	0.454677
H	-3.929426	5.903577	0.754600
O	-0.142726	1.118638	2.663292
C	0.719021	2.055803	3.202963
H	1.375280	2.536598	2.451425
O	1.607178	1.482689	4.122742
H	1.090306	1.152040	4.867825
H	1.176950	-0.604115	1.875224
H	0.709325	0.083400	2.372495
H	0.110655	2.859375	3.671191
C	-2.573202	-0.847477	2.664314
O	-1.374343	-1.321971	2.018197
C	-0.774230	-2.368771	2.811940
C	-1.856285	-2.793107	3.775132
C	-2.558412	-1.470896	4.040954
H	-2.548969	0.251469	2.669686
H	-3.436901	-1.193490	2.069539
H	-0.426993	-3.161108	2.131514
H	0.101749	-1.955564	3.343635
H	-2.541332	-3.510368	3.296570
H	-1.452532	-3.271389	4.676382
H	-3.563446	-1.577647	4.468404
H	-1.965168	-0.851589	4.731671

IXc-XV

H	6.172334	-3.013259	-1.005497
H	4.752804	-3.101990	-3.052082
C	5.115083	-2.744819	-0.952286
C	4.321648	-2.790856	-2.098060
H	5.157592	-2.317284	1.164777
C	4.547563	-2.358235	0.259410
C	2.970945	-2.454067	-2.031777
H	2.375369	-2.523394	-2.944946
H	-2.290059	-5.618401	-1.792737
H	-1.264803	-7.059908	-0.037044
H	6.968944	1.021010	1.440043
H	6.455838	1.046652	-0.997191
C	3.200905	-2.004180	0.320073
C	-1.500680	-5.223419	-1.148919
C	-0.926495	-6.030159	-0.169230
C	2.390646	-2.042158	-0.822231
H	-1.535827	-3.306926	-2.104563
C	5.933044	1.062047	1.097101
C	-1.067105	-3.908921	-1.324141
C	5.645385	1.077169	-0.265606
H	2.774783	-1.678377	1.271855
C	0.097475	-5.519319	0.629121
H	0.569982	-6.149039	1.386188
H	5.106882	1.108964	3.091996
C	4.888949	1.108217	2.021421
C	-0.051111	-3.378206	-0.518160
H	0.585408	-1.821819	-3.123525
C	0.533140	-4.209158	0.451304
C	4.323300	1.129833	-0.706624
H	4.134182	1.142567	-1.782238
H	2.400829	0.160924	-2.273390
H	1.359109	-3.834055	1.063379
H	1.369725	-0.085144	-4.606692
P	0.583585	-1.652897	-0.682953
C	3.570269	1.154655	1.579104
C	0.082901	-1.173433	-2.390720
C	3.265601	1.157341	0.211679
H	2.753154	1.177680	2.304709
H	-0.988465	-1.409411	-2.488710
H	-0.397368	-0.052541	-4.783521
C	1.583339	0.878630	-2.111433
C	0.451949	0.417111	-4.266294
H	1.872718	1.802085	-2.637064
C	0.297339	0.318840	-2.747555
H	0.504145	1.465188	-4.595269
P	1.496632	1.258839	-0.293561
Ru	-0.013822	-0.025053	0.820844
H	2.929060	3.469742	-1.628200
H	-4.284786	-3.428455	-0.539948
H	-2.383873	-1.954257	0.064716
C	-4.229985	-2.417998	-0.954824
C	-3.157275	-1.591117	-0.615835
C	-0.913924	1.182322	-2.340936
C	2.110751	3.907246	-1.047284
H	-1.710031	1.096654	-3.098405
C	1.258982	3.087619	-0.288108
H	-6.063174	-2.601660	-2.081666
C	-5.221621	-1.957844	-1.817275
H	-0.601369	2.239854	-2.337105
P	-1.652392	0.810573	-0.682896
C	-3.064121	-0.295663	-1.134064
H	2.607430	5.913676	-1.649993
C	1.938814	5.287734	-1.055093
C	-5.138599	-0.665579	-2.340487
C	-4.071734	0.160500	-1.998934
C	0.253341	3.681482	0.480523
H	-5.915285	-0.296358	-3.013602
H	-0.400563	3.054925	1.088274
H	-4.033501	1.177995	-2.400577
C	0.919433	5.871058	-0.298538
C	-2.573363	2.355861	-0.304090

C	0.083636	5.067690	0.471586
H	-2.918708	1.760937	1.746573
H	-2.402452	3.273640	-2.270299
H	0.785901	6.954996	-0.306958
C	-3.109149	2.515670	0.982692
C	-2.796606	3.361581	-1.255619
H	-0.713073	5.515439	1.070490
C	-3.845104	3.652923	1.307465
C	-3.533063	4.498942	-0.926941
H	-4.251797	3.763019	2.315213
H	-3.696847	5.271389	-1.681503
C	-4.058322	4.649022	0.354839
H	-4.634671	5.540651	0.610833
O	-0.685194	1.331821	2.403494
C	0.127447	1.687027	3.320946
H	1.030505	2.280227	3.035482
O	0.846145	0.296405	3.884055
H	1.716522	0.472724	4.275175
H	1.194240	-0.677564	1.877089
H	1.008069	-0.222296	2.835797
H	-0.317729	2.062922	4.263509
C	-2.705678	-0.912293	2.541606
O	-1.427385	-1.335322	2.043619
C	-1.009862	-2.390297	2.918514
C	-2.257161	-3.236205	3.045547
C	-3.385102	-2.193522	3.026419
H	-2.545494	-0.181328	3.351800
H	-3.226782	-0.403023	1.719326
H	-0.149760	-2.889790	2.458709
H	-0.693111	-1.957452	3.885972
H	-2.329171	-3.913230	2.179674
H	-2.257117	-3.861010	3.947885
H	-4.202934	-2.495132	2.358077
H	-3.829450	-2.047816	4.020137

IXc-XV (thf)

H	4.879970	-2.526106	-3.868881
H	2.736165	-2.833133	-5.101995
C	3.927059	-2.349739	-3.365343
C	2.727345	-2.516868	-4.056614
H	4.832237	-1.827051	-1.476043
C	3.898837	-1.961890	-2.028187
C	1.510353	-2.294555	-3.415619
H	0.589684	-2.458960	-3.980447
H	-2.960986	-5.774708	-1.096749
H	-1.187123	-7.154413	-0.018834
H	6.343528	1.691407	-1.828076
H	4.852664	1.687727	-3.823052
C	2.680677	-1.721989	-1.394001
C	-1.995393	-5.329747	-0.844974
C	-1.003667	-6.101872	-0.244389
C	1.467307	-1.877082	-2.076345
H	-2.559494	-3.411903	-1.615221
C	5.260928	1.617675	-1.703847
C	-1.762275	-3.985915	-1.139597
C	4.426669	1.618042	-2.819698
H	2.670712	-1.392142	-0.352443
C	0.232377	-5.525266	0.050024
H	1.024598	-6.126075	0.502296
H	5.340752	1.539647	0.458372
C	4.702620	1.530600	-0.428521
C	-0.531142	-3.390085	-0.835210
H	-1.135544	-1.786841	-3.413349
C	0.465925	-4.185676	-0.247768
C	3.043727	1.531185	-2.663991
H	2.418166	1.539014	-3.559396
H	0.741621	0.320917	-3.315399
H	1.450254	-3.755169	-0.037922
H	-1.146014	0.001499	-5.031511
P	-0.138608	-1.618476	-1.182619
C	3.322262	1.433349	-0.273481
C	-1.331227	-1.178548	-2.517921
C	2.473485	1.428853	-1.387803
H	2.904492	1.345521	0.732635
H	-2.329535	-1.490459	-2.172063
H	-2.826792	-0.094724	-4.464937
C	0.013841	0.978044	-2.816757
C	-1.872956	0.435629	-4.328883
H	-0.002692	1.914787	-3.394958
C	-1.380293	0.325534	-2.884786
H	-2.030892	1.484835	-4.618415
P	0.652787	1.363722	-1.113123
Ru	-0.158574	-0.035668	0.481393
H	1.099557	3.679329	-2.909127
H	-4.379393	-3.711969	0.939555
H	-2.494580	-2.112228	0.725321
C	-4.567189	-2.700123	0.569037
C	-3.504443	-1.802807	0.446444
C	-2.368764	1.100454	-1.990553
C	0.611402	4.047386	-2.001165
H	-3.400499	0.950090	-2.348358
C	0.286346	3.165646	-0.956826
H	-6.690178	-3.007132	0.317177
C	-5.856835	-2.308034	0.219575
H	-2.156942	2.177489	-2.095129
P	-2.317909	0.693723	-0.182921
C	-3.718761	-0.504056	-0.025411
H	0.593542	6.080979	-2.708333
C	0.337691	5.406776	-1.887990
C	-6.082092	-1.013184	-0.252920
C	-5.023522	-0.117116	-0.370370
C	-0.286175	3.678390	0.211587
H	-7.091706	-0.697312	-0.524323
H	-0.521990	3.000644	1.034659
H	-5.220111	0.900778	-0.721620
C	-0.254186	5.907641	-0.725932
C	-3.087261	2.179325	0.578490

C	-0.556583	5.044037	0.322686
H	-2.498741	1.576594	2.569784
H	-3.811165	3.095822	-1.258361
H	-0.469618	6.975017	-0.640039
C	-3.044269	2.309996	1.974790
C	-3.749613	3.163082	-0.170046
H	-1.014056	5.427783	1.237852
C	-3.646102	3.398387	2.602095
C	-4.349493	4.251779	0.461352
H	-3.599086	3.487809	3.689759
H	-4.860359	5.008246	-0.138433
C	-4.299297	4.373355	1.848462
H	-4.769296	5.226917	2.341832
O	-0.216185	1.272711	2.204418
C	0.906055	1.596472	2.785408
H	1.579845	2.294306	2.225103
O	1.751752	0.323905	2.951305
H	2.730649	0.512796	2.969597
H	1.412317	-0.614667	0.925258
H	1.532469	-0.183152	1.901100
H	0.792863	1.959955	3.827588
C	-1.799136	-1.106184	3.155422
O	-0.843231	-1.453067	2.143169
C	-0.056166	-2.506807	2.705478
C	-1.099301	-3.417710	3.314615
C	-2.157324	-2.431139	3.830897
H	-1.336021	-0.383734	3.848047
H	-2.640328	-0.606239	2.655791
H	0.539791	-2.949457	1.899213
H	0.629760	-2.085631	3.465072
H	-1.515642	-4.070332	2.530910
H	-0.691794	-4.069483	4.098191
H	-3.173873	-2.756175	3.570943
H	-2.129539	-2.332181	4.924449
C	5.415491	1.109047	3.507193
O	4.392331	0.468890	2.726214
C	4.828371	-0.834679	2.306858
C	6.093380	-1.110118	3.092759
C	6.664931	0.289105	3.267073
H	5.492589	2.157613	3.184925
H	5.118232	1.101897	4.570660
H	4.015600	-1.555828	2.493850
H	5.010538	-0.810962	1.216437
H	5.851089	-1.550810	4.072157
H	6.770840	-1.805154	2.580830
H	7.392244	0.371314	4.084539
H	7.171426	0.617137	2.345569

IX-Xb

H	6.449176	2.224879	0.233981
H	5.219911	2.526064	2.379519
C	5.362171	2.123657	0.262281
C	4.675317	2.288113	1.463258
H	5.182056	1.700442	-1.849235
C	4.653779	1.831435	-0.902324
C	3.287413	2.157702	1.502284
H	2.782169	2.301936	2.460206
H	-1.015634	6.265464	1.062289
H	-1.103192	6.802018	-1.366975
H	6.612521	-1.924690	-1.492091
H	6.187015	-1.626808	0.942748
C	3.269631	1.690523	-0.862008
C	-0.704208	5.522288	0.325166
C	-0.751685	5.822657	-1.035371
C	2.565894	1.846104	0.340914
H	-0.222699	4.065195	1.824767
C	5.590022	-1.817352	-1.123874
C	-0.256159	4.273836	0.752869
C	5.352277	-1.650539	0.238767
H	2.729603	1.435144	-1.777680
C	-0.343936	4.872103	-1.971498
H	-0.375178	5.102520	-3.038359
H	4.695318	-1.990419	-3.083760
C	4.517203	-1.852812	-2.015138
C	0.143188	3.310048	-0.183028
H	0.889975	2.104673	2.699935
C	0.100583	3.622017	-1.549271
C	4.048156	-1.514471	0.714027
H	3.891818	-1.396433	1.788741
H	2.253247	-0.181925	2.196827
H	0.399422	2.877703	-2.291429
H	1.248740	0.499087	4.466060
P	0.732893	1.649964	0.305801
C	3.215594	-1.706046	-1.545069
C	0.223677	1.494396	2.073280
C	2.967245	-1.529845	-0.176493
H	2.380678	-1.713091	-2.252682
H	-0.766434	1.969623	2.159029
H	-0.496411	0.808030	4.574988
C	1.338736	-0.789320	2.117404
C	0.260370	0.129578	4.155333
H	1.505715	-1.652641	2.778017
C	0.151144	0.052021	2.632360
H	0.114190	-0.857776	4.617302
P	1.224178	-1.351234	0.364858
Ru	-0.069165	-0.050862	-0.953536
H	0.863271	-3.449345	2.569060
H	-3.670737	3.899453	-1.243359
H	-2.237690	1.877211	-1.286771
C	-3.667913	3.222586	-0.386095
C	-2.862606	2.083879	-0.410875
C	-1.204972	-0.610709	2.289498
C	0.572168	-3.862877	1.600735
H	-1.976214	-0.277351	3.000574
C	0.647804	-3.094752	0.431074
H	-5.075186	4.396377	0.755972
C	-4.452299	3.499599	0.731481
H	-1.125410	-1.701272	2.427594
P	-1.785356	-0.284840	0.573298
C	-2.845059	1.206505	0.682975
H	0.066941	-5.767810	2.472640
C	0.120061	-5.182187	1.552282
C	-4.448642	2.624831	1.819522
C	-3.657090	1.478944	1.793782
C	0.277814	-3.683006	-0.788826
H	-5.073186	2.831886	2.691143
H	0.333359	-3.098700	-1.711685
H	-3.688608	0.794149	2.645919
C	-0.251022	-5.753894	0.336482
C	-2.986912	-1.613326	0.176579

C	-0.165764	-5.002089	-0.835552
H	-4.221233	-0.280528	-1.013204
H	-1.992884	-3.215311	1.261231
H	-0.599197	-6.788457	0.301534
C	-4.054594	-1.312330	-0.688441
C	-2.814594	-2.940517	0.596155
H	-0.446287	-5.445534	-1.793434
C	-4.921755	-2.313704	-1.121460
C	-3.691212	-3.937063	0.167713
H	-5.750142	-2.060362	-1.786489
H	-3.546260	-4.962837	0.513990
C	-4.742081	-3.629715	-0.694476
H	-5.425265	-4.412979	-1.028834
O	-0.982170	0.845785	-2.866889
C	-1.636195	-0.269011	-3.298148
H	-2.732130	-0.180473	-3.157030
O	-1.160071	-1.393945	-2.493771
H	-1.914400	-1.860157	-2.099900
H	0.275940	0.544205	-2.730987
H	1.117218	0.222048	-2.291513
H	-1.427729	-0.511887	-4.356755

Xb

H	6.776550	1.189694	0.553118
H	5.504712	1.939162	2.559074
C	5.687504	1.262443	0.515599
C	4.975654	1.678037	1.640023
H	5.550243	0.612230	-1.540258
C	5.002175	0.941821	-0.654645
C	3.585422	1.769917	1.596309
H	3.059881	2.110178	2.491843
H	-0.169187	6.392874	0.651188
H	0.274383	6.828238	-1.758161
H	6.248618	-3.189000	-1.223550
H	5.712419	-2.910715	1.191497
C	3.612240	1.022227	-0.696135
C	0.177517	5.584451	0.003701
C	0.425918	5.828747	-1.345513
C	2.884050	1.431253	0.429278
H	0.164143	4.141955	1.596769
C	5.253117	-2.859697	-0.918020
C	0.369254	4.309313	0.536636
C	4.953618	-2.703294	0.433797
H	3.074412	0.737315	-1.604947
C	0.883395	4.795275	-2.165288
H	1.100963	4.987956	-3.218321
H	4.501704	-2.726778	-2.939662
C	4.274967	-2.600154	-1.878663
C	0.811198	3.263030	-0.281078
H	1.130918	2.045320	2.698557
C	1.075517	3.521617	-1.636043
C	3.682963	-2.285074	0.827741
H	3.472351	-2.182439	1.894932
H	2.182993	-0.490681	2.183092
H	1.454772	2.711272	-2.270961
H	1.313142	0.374458	4.421082
P	1.047118	1.541826	0.296991
C	3.010169	-2.169378	-1.488610
C	0.432423	1.513905	2.035311
C	2.703180	-2.000425	-0.131929
H	2.254714	-1.931011	-2.243640
H	-0.495678	2.109159	2.052538
H	-0.365070	0.943605	4.544394
C	1.177980	-0.929874	2.083488
C	0.277181	0.157939	4.120873
H	1.182708	-1.813523	2.738732
C	0.142360	0.093279	2.598852
H	-0.012565	-0.794670	4.588299
P	1.007748	-1.456038	0.317793
Ru	-0.029014	0.056805	-0.967112
H	0.259744	-3.492237	2.486778
H	-3.138132	4.728204	-0.426584
H	-1.780743	2.671047	-0.719540
C	-3.359801	3.842678	0.173694
C	-2.592059	2.688060	0.011624
C	-1.301854	-0.363938	2.277513
C	-0.097182	-3.831462	1.511249
H	-2.007076	0.098944	2.986193
C	0.112029	-3.064710	0.357584
H	-5.006989	4.763835	1.221459
C	-4.404596	3.861786	1.094290
H	-1.374165	-1.451502	2.440603
P	-1.845530	0.026466	0.554937
C	-2.859144	1.545552	0.773478
H	-0.919114	-5.640775	2.346022
C	-0.765486	-5.054500	1.437201
C	-4.687379	2.722039	1.850986
C	-3.922085	1.570835	1.690546
C	-0.345819	-3.560893	-0.873420
H	-5.512294	2.730738	2.566659
H	-0.173907	-2.982056	-1.785415
H	-4.163597	0.678337	2.277066
C	-1.223785	-5.532058	0.210921
C	-3.145337	-1.191077	0.122466

C	-1.007330	-4.783634	-0.946466
H	-3.922803	0.151989	-1.396922
H	-2.642435	-2.763931	1.532430
H	-1.741530	-6.492192	0.155821
C	-3.980599	-0.853881	-0.962232
C	-3.275638	-2.461168	0.695662
H	-1.353241	-5.156541	-1.913125
C	-4.910268	-1.766804	-1.458464
C	-4.213727	-3.368674	0.202067
H	-5.558461	-1.483930	-2.291185
H	-4.304379	-4.351881	0.669656
C	-5.026714	-3.030816	-0.877567
H	-5.757987	-3.745264	-1.260500
O	-1.233261	1.218882	-2.652739
C	-1.395438	0.066729	-3.471590
H	-2.380200	0.092978	-3.965040
O	-1.280934	-1.046163	-2.635565
H	-2.155022	-1.289364	-2.275992
H	-0.614317	1.838183	-3.064278
H	1.198922	0.079961	-2.086757
H	-0.592799	0.009360	-4.220777

XIb

H	-6.096468	-3.261300	-0.927524
H	-5.482273	-1.932859	-2.944150
C	-5.273568	-2.544726	-0.882222
C	-4.926719	-1.804577	-2.012637
H	-4.832992	-2.927825	1.197775
C	-4.568456	-2.358003	0.303940
C	-3.877648	-0.889532	-1.958142
H	-3.647836	-0.310620	-2.855793
H	-3.140748	5.258428	-1.568187
H	-4.964144	5.224835	0.130263
H	-3.103908	-6.343566	1.469833
H	-2.667453	-6.061057	-0.965014
C	-3.508863	-1.453208	0.353586
C	-3.323421	4.358899	-0.975598
C	-4.344890	4.339563	-0.028348
C	-3.140297	-0.712779	-0.776492
H	-1.748016	3.275749	-1.940411
C	-2.503994	-5.502975	1.114674
C	-2.533997	3.227561	-1.184499
C	-2.260425	-5.344381	-0.248437
H	-2.945386	-1.326311	1.281238
C	-4.583193	3.175692	0.703144
H	-5.393221	3.143117	1.434983
H	-2.158981	-4.706067	3.093554
C	-1.974811	-4.586485	2.023567
C	-2.754475	2.055672	-0.447204
H	-1.981685	0.654084	-3.093919
C	-3.799854	2.044650	0.490744
C	-1.491793	-4.274494	-0.704758
H	-1.303395	-4.181393	-1.777044
H	-1.419440	-2.002849	-2.249670
H	-4.015914	1.131490	1.053663
H	-1.124713	-1.028820	-4.608603
P	-1.760414	0.515883	-0.662419
C	-1.221420	-3.508186	1.566938
C	-1.135984	0.659020	-2.390563
C	-0.975143	-3.336885	0.198650
H	-0.842764	-2.767635	2.278735
H	-0.689351	1.661837	-2.485842
H	-0.071503	0.381721	-4.844222
C	-0.348185	-1.768460	-2.152056
C	-0.163569	-0.579383	-4.318091
H	0.168982	-2.556914	-2.718628
C	-0.075700	-0.392871	-2.802170
H	0.636815	-1.237711	-4.687709
P	0.062537	-1.924331	-0.354947
Ru	-0.063963	0.038775	0.757695
H	2.075125	-2.898769	-2.476897
H	-0.044595	5.518202	-0.603083
H	-0.104687	3.091459	-0.062456
C	0.741832	4.857932	-0.979850
C	0.700833	3.494815	-0.682862
C	1.347718	0.107301	-2.478707
C	2.462270	-3.089150	-1.473352
H	1.650485	0.865634	-3.218853
C	1.724346	-2.739425	-0.335802
H	1.821672	6.438473	-1.979566
C	1.783863	5.371708	-1.748896
H	2.044224	-0.733006	-2.605969
P	1.594296	0.829955	-0.784001
C	1.694511	2.632187	-1.157503
H	4.265909	-3.968367	-2.258221
C	3.710777	-3.702103	-1.355623
C	2.785480	4.519830	-2.220184
C	2.743094	3.159355	-1.926732
C	2.263651	-3.056555	0.921613
H	3.607066	4.920576	-2.817769
H	1.670209	-2.865450	1.820567
H	3.538865	2.502388	-2.292605
C	4.246832	-3.976620	-0.100918
C	3.360450	0.464633	-0.401486

C	3.512534	-3.659642	1.042415
H	3.197843	1.512819	1.486519
H	3.911813	-0.615287	-2.206887
H	5.225271	-4.452900	-0.011863
C	3.856233	0.945707	0.823878
C	4.237692	-0.242527	-1.234283
H	3.906065	-3.902707	2.032097
C	5.173623	0.711610	1.209788
C	5.556760	-0.479659	-0.846219
H	5.535532	1.100344	2.164259
H	6.220233	-1.035519	-1.512911
C	6.028556	-0.010289	0.376753
H	7.061656	-0.197014	0.676775
O	1.564767	-0.310539	2.325818
C	1.213217	-0.868197	3.609399
H	1.120635	-1.971371	3.498867
O	0.074656	-0.276701	4.044814
H	-0.598394	-0.412069	3.338517
H	-1.213056	-0.543385	1.810723
H	2.022329	-0.650650	4.325788
C	0.877146	2.726024	2.487127
O	-0.219084	1.927242	2.024382
C	-1.337962	2.296132	2.844102
C	-1.255798	3.804688	2.858486
C	0.256346	4.057933	2.916814
H	1.370153	2.203807	3.325141
H	1.593061	2.810408	1.658187
H	-2.240374	1.873999	2.389713
H	-1.206061	1.861478	3.851423
H	-1.690393	4.205775	1.928825
H	-1.803862	4.254847	3.696073
H	0.559684	4.881005	2.255694
H	0.584297	4.329916	3.929234
H	2.363453	-0.738483	1.975286

XIIb

H	-6.753363	0.889263	-0.596023
H	-5.486992	1.845766	-2.516198
C	-5.672640	1.029453	-0.524577
C	-4.963748	1.560958	-1.600877
H	-5.541026	0.263734	1.491368
C	-4.995124	0.681306	0.642352
C	-3.584257	1.741061	-1.512393
H	-3.062966	2.174580	-2.369416
H	-0.568853	6.588146	-0.504562
H	-0.916065	6.956180	1.932970
H	-6.120051	-3.465407	0.597758
H	-5.496338	-2.826271	-1.727455
C	-3.615138	0.849775	0.727172
C	-0.749154	5.738814	0.157876
C	-0.944121	5.944829	1.522277
C	-2.888887	1.375468	-0.349835
H	-0.628748	4.314798	-1.443555
C	-5.121421	-3.078533	0.384234
C	-0.783009	4.448579	-0.370403
C	-4.773081	-2.719989	-0.916031
H	-3.085271	0.547872	1.634698
C	-1.184177	4.855497	2.360556
H	-1.349483	5.011453	3.428628
H	-4.442717	-3.240293	2.430192
C	-4.184654	-2.950130	1.409502
C	-1.006914	3.347964	0.466974
H	-1.138280	2.267462	-2.512356
C	-1.215867	3.565909	1.836409
C	-3.498345	-2.227509	-1.193435
H	-3.253863	-1.971169	-2.226208
H	-2.002992	-0.329141	-2.265379
H	-1.409217	2.716007	2.498905
H	-1.116460	0.768426	-4.385784
P	-1.070517	1.623812	-0.151930
C	-2.916830	-2.444708	1.136845
C	-0.411492	1.744526	-1.873501
C	-2.560716	-2.067969	-0.164486
H	-2.194544	-2.337258	1.948469
H	0.463711	2.413784	-1.824642
H	0.516916	1.466445	-4.382666
C	-0.979713	-0.724616	-2.174990
C	-0.079314	0.601079	-4.059452
H	-0.919384	-1.533949	-2.918100
C	0.002905	0.407405	-2.543790
H	0.299936	-0.281362	-4.595784
P	-0.861936	-1.440230	-0.475686
Ru	0.082449	0.075822	1.028284
H	0.404950	-2.975458	-2.805363
H	2.815749	5.075337	0.776552
H	1.593287	2.936347	0.931147
C	3.135342	4.227952	0.165752
C	2.446247	3.016889	0.251961
C	1.463646	0.030830	-2.204345
C	0.552852	-3.521474	-1.870302
H	2.153726	0.601868	-2.845575
C	0.066202	-3.020015	-0.656989
H	4.767476	5.295702	-0.758405
C	4.225395	4.350080	-0.691958
H	1.627708	-1.028591	-2.454859
P	1.944158	0.313438	-0.442422
C	2.835322	1.919850	-0.522934
H	1.605356	-5.113831	-2.870774
C	1.237519	-4.737085	-1.913696
C	4.629815	3.257651	-1.462604
C	3.940919	2.051686	-1.379219
C	0.261159	-3.779853	0.510467
H	5.491148	3.345449	-2.128143
H	-0.156605	-3.435524	1.463097
H	4.277923	1.197830	-1.975927
C	1.444768	-5.470351	-0.747913
C	3.315899	-0.847035	-0.095508

C	0.948157	-4.991181	0.464819
H	4.003461	0.395777	1.541809
H	2.891312	-2.328249	-1.627602
H	1.979007	-6.421884	-0.785260
C	4.142891	-0.544999	0.999773
C	3.508254	-2.059417	-0.768774
H	1.082197	-5.571582	1.380404
C	5.135485	-1.430806	1.405497
C	4.500171	-2.950542	-0.355400
H	5.774382	-1.176307	2.253853
H	4.637539	-3.889601	-0.896473
C	5.313927	-2.640791	0.731530
H	6.092097	-3.336931	1.051170
O	1.380660	-1.374416	2.241551
C	0.949558	-1.739141	3.556937
H	1.604351	-2.533002	3.943674
O	-0.341310	-2.198586	3.586009
H	-0.903665	-1.454673	3.297478
H	-1.218289	-0.190341	2.026899
H	1.097900	-0.823739	4.155429
H	1.609905	-2.169465	1.732331
H	0.277404	1.102237	2.435009
H	1.012607	1.243565	1.985128

XIIb-XV

H	0.667573	-6.999755	-0.373901
H	-1.272704	-6.245833	-1.743319
C	0.339927	-5.958932	-0.331895
C	-0.744980	-5.535900	-1.102953
H	1.837901	-5.373479	1.111172
C	0.993437	-5.052035	0.497342
C	-1.163494	-4.210243	-1.051094
H	-2.029796	-3.901751	-1.645221
H	-6.021188	-1.978161	-0.986323
H	-6.528470	-2.329976	1.426376
H	5.706752	-4.331329	1.212619
H	5.034194	-4.153973	-1.178351
C	0.571939	-3.722269	0.550229
C	-5.214316	-1.978149	-0.249823
C	-5.498260	-2.173960	1.099420
C	-0.500252	-3.282042	-0.231026
H	-3.709701	-1.641255	-1.738939
C	4.990255	-3.569126	0.899638
C	-3.898940	-1.781672	-0.672116
C	4.612998	-3.470522	-0.438073
H	1.094103	-3.028018	1.208866
C	-4.458357	-2.179928	2.030039
H	-4.669977	-2.346821	3.088438
H	4.754512	-2.746929	2.884619
C	4.455125	-2.684493	1.836335
C	-2.850509	-1.766860	0.255890
H	-1.163107	-2.037358	-2.571877
C	-3.147140	-1.975139	1.611277
C	3.698151	-2.498179	-0.841326
H	3.428346	-2.436307	-1.897915
H	1.311781	-1.899000	-2.055866
H	-2.338396	-1.977699	2.348618
H	0.213081	-1.294432	-4.416981
P	-1.083182	-1.532635	-0.189707
C	3.535514	-1.717600	1.436827
C	-1.132050	-1.098164	-1.997656
C	3.138608	-1.620482	0.095156
H	3.125436	-1.028197	2.183244
H	-2.082241	-0.584885	-2.202057
H	-0.996823	0.003952	-4.443143
C	1.384812	-0.798349	-2.063393
C	-0.008965	-0.281210	-4.052086
H	2.172633	-0.564919	-2.796029
C	0.023164	-0.221100	-2.523487
H	0.732041	0.401968	-4.493077
P	1.945633	-0.308440	-0.381989
Ru	0.091325	0.003968	1.060824
H	2.881554	1.325783	-2.784031
H	-5.332577	1.623503	1.550792
H	-2.864834	1.544312	1.612242
C	-4.768514	1.623839	0.615590
C	-3.378156	1.576641	0.647985
C	-0.163759	1.260831	-2.111527
C	3.360812	1.691249	-1.872408
H	-0.810183	1.783155	-2.833018
C	3.102763	1.096672	-0.630408
H	-6.529763	1.690318	-0.634738
C	-5.438455	1.661136	-0.607013
H	0.796396	1.798095	-2.166117
P	-0.794473	1.577470	-0.409658
C	-2.628114	1.568841	-0.536892
H	4.430754	3.220981	-2.948812
C	4.241666	2.768725	-1.973049
C	-4.707422	1.662923	-1.792666
C	-3.313314	1.623808	-1.758263
C	3.756044	1.595798	0.508608
H	-5.221964	1.701391	-2.755237
H	3.548467	1.164209	1.489673
H	-2.769602	1.638266	-2.706001
C	4.882709	3.258169	-0.837590
C	-0.421310	3.353221	-0.163913

C	4.640350	2.665416	0.402399
H	-2.446006	4.083637	-0.464637
H	1.669556	2.955935	0.226370
H	5.574848	4.098909	-0.919165
C	-1.407145	4.346966	-0.252091
C	0.902670	3.723990	0.115220
H	5.141939	3.040826	1.297024
C	-1.067055	5.687411	-0.071118
C	1.235987	5.064141	0.289396
H	-1.841660	6.453767	-0.143730
H	2.271032	5.337200	0.507224
C	0.251876	6.048923	0.197618
H	0.512723	7.099986	0.338711
O	-0.554514	1.362417	3.810664
C	0.546874	2.211815	3.038584
H	-0.083594	2.987961	2.538267
O	1.217037	1.397082	2.292273
H	1.072237	-1.121086	2.045193
H	0.289840	-1.193771	2.366653
H	1.119020	2.664198	3.873118
H	-1.277065	1.933754	4.116540
H	-0.940492	0.696424	2.861315
H	-1.325785	0.184976	2.037832

XIb-XV

H	4.540467	-5.219878	0.428826
H	4.125882	-4.291728	-1.843000
C	3.672927	-4.581796	0.247116
C	3.440745	-4.063306	-1.023481
H	2.961566	-4.689408	2.285725
C	2.790084	-4.282867	1.286308
C	2.326658	-3.255729	-1.261111
H	2.163155	-2.881146	-2.274968
H	-3.106305	-5.647147	-1.460249
H	-3.903830	-5.893057	0.885293
H	6.989278	-1.776730	0.657504
H	6.570578	-0.630068	-1.516003
C	1.695283	-3.458207	1.054287
C	-2.732735	-4.978242	-0.681840
C	-3.178230	-5.117198	0.631820
C	1.445957	-2.935053	-0.223375
H	-1.458330	-3.915869	-2.043004
C	6.003911	-1.359400	0.438902
C	-1.802936	-3.992189	-1.008993
C	5.769003	-0.720767	-0.779645
H	1.024945	-3.204828	1.882235
C	-2.687442	-4.265027	1.621251
H	-3.027767	-4.370301	2.654165
H	5.153412	-1.957970	2.330371
C	4.977539	-1.457452	1.375082
C	-1.315566	-3.122385	-0.025139
H	0.221818	-2.441268	-2.756613
C	-1.764529	-3.274943	1.292405
C	4.512555	-0.189972	-1.056512
H	4.354487	0.328239	-2.007443
H	2.347624	-0.746737	-2.513920
H	-1.398695	-2.590149	2.060311
H	1.146143	-1.214436	-4.592956
P	-0.034267	-1.848537	-0.393210
C	3.717109	-0.926005	1.097777
C	-0.239676	-1.596481	-2.221836
C	3.468482	-0.296085	-0.123026
H	2.908382	-1.006458	1.829814
H	-1.320667	-1.685285	-2.422916
H	-0.575644	-0.817224	-4.767841
C	1.672412	0.101819	-2.322792
C	0.377104	-0.468268	-4.343641
H	2.073353	0.929772	-2.928976
C	0.265646	-0.263245	-2.829659
H	0.645021	0.467798	-4.855572
P	1.850922	0.510720	-0.514953
Ru	0.017576	0.126882	0.715765
H	1.488323	2.917519	-2.331159
H	-5.033383	-2.380356	0.226493
H	-2.946837	-1.094785	0.594462
C	-4.715378	-1.640792	-0.512321
C	-3.540733	-0.916723	-0.302966
C	-0.736940	0.894428	-2.603138
C	2.137907	3.200818	-1.499060
H	-1.525262	0.849075	-3.370008
C	2.490473	2.256010	-0.526912
H	-6.384405	-1.995262	-1.835538
C	-5.467295	-1.427428	-1.664328
H	-0.231694	1.860258	-2.765712
P	-1.536233	0.944513	-0.933162
C	-3.099980	0.023664	-1.239390
H	2.317326	5.228212	-2.213636
C	2.608646	4.514214	-1.439919
C	-5.048721	-0.478631	-2.600353
C	-3.876712	0.242381	-2.389569
C	3.374703	2.659050	0.490016
H	-5.642270	-0.297250	-3.499119
H	3.723109	1.928030	1.226144
H	-3.579444	0.996766	-3.124373
C	3.457835	4.908162	-0.407752
C	-2.138626	2.674198	-0.787485

C	3.848696	3.971198	0.553912
H	-4.238889	2.208167	-0.511675
H	-0.143435	3.477340	-0.959780
H	3.834684	5.931907	-0.363421
C	-3.480173	2.994915	-0.535861
C	-1.196661	3.713277	-0.790235
H	4.547282	4.256847	1.344435
C	-3.861948	4.318893	-0.314878
C	-1.576819	5.035182	-0.574652
H	-4.912689	4.550794	-0.127080
H	-0.823015	5.827253	-0.589278
C	-2.916637	5.342790	-0.335839
H	-3.222103	6.376929	-0.164642
O	-0.040164	2.073240	1.797643
C	0.876726	2.565641	2.647413
H	0.549940	2.734964	3.689541
O	0.933737	3.954086	2.018991
H	1.775843	4.066219	1.526061
H	1.035985	-0.469771	1.860488
H	1.891005	2.122879	2.615809
C	-2.699931	0.834532	2.445190
O	-1.623637	-0.131686	2.329640
C	-1.124359	-0.439994	3.644229
C	-2.266737	-0.113345	4.572428
C	-2.816232	1.147618	3.922929
H	-2.455398	1.711841	1.830252
H	-3.615594	0.366706	2.047005
H	-0.796650	-1.489659	3.647337
H	-0.235275	0.182436	3.849583
H	-3.016690	-0.920038	4.560781
H	-1.946498	0.026381	5.612782
H	-3.843948	1.390236	4.221705
H	-2.187624	2.013572	4.182560
H	0.171156	3.364716	1.373086

XVI

H	6.750726	-1.251857	0.036846
H	5.447701	-1.334927	2.156530
C	5.765842	-0.783721	0.094162
C	5.036941	-0.832599	1.278287
H	5.810234	-0.063339	-1.944528
C	5.236820	-0.123137	-1.016927
C	3.779890	-0.228532	1.354882
H	3.247295	-0.260560	2.308071
H	4.330868	5.257978	0.660697
H	2.745857	6.721027	-0.584303
H	4.630621	-5.434783	-0.290689
H	2.858773	-5.796298	1.421474
C	3.976661	0.459797	-0.946798
C	3.409332	4.842040	0.248246
C	2.520765	5.661111	-0.448909
C	3.226381	0.407628	0.238780
H	3.838801	2.855011	0.955411
C	3.796297	-4.734600	-0.211736
C	3.129082	3.488438	0.415306
C	2.804590	-4.936920	0.749976
H	3.567705	0.965524	-1.827388
C	1.348946	5.122404	-0.975757
H	0.648525	5.756400	-1.523771
H	4.484820	-3.484030	-1.832980
C	3.714414	-3.644967	-1.075438
C	1.950896	2.937385	-0.114170
H	1.914003	1.265241	2.595711
C	1.063944	3.767561	-0.810115
C	1.738182	-4.048652	0.849137
H	0.957124	-4.225823	1.595302
H	1.544844	-1.427306	2.223962
H	0.136168	3.365233	-1.222776
H	1.097762	-0.172108	4.360678
P	1.558614	1.174871	0.203658
C	2.649529	-2.749435	-0.974864
C	1.010475	1.224598	1.966860
C	1.657704	-2.939555	-0.008677
H	2.594805	-1.889093	-1.646736
H	0.534637	2.208337	2.102938
H	-0.103100	1.133205	4.398584
C	0.476866	-1.284412	2.003255
C	0.105483	0.131397	3.996052
H	-0.035508	-2.042859	2.615269
C	0.048508	0.124848	2.465231
H	-0.632525	-0.560022	4.427975
P	0.274925	-1.762617	0.222709
Ru	0.072132	0.082967	-1.188470
H	-1.976179	-2.426684	2.164099
H	-3.296589	4.712056	-2.214861
H	-2.896309	2.300242	-1.840347
C	-2.865171	4.377115	-1.269191
C	-2.640127	3.021118	-1.055611
C	-1.403886	0.443827	2.063194
C	-2.117620	-2.967584	1.225986
H	-1.764392	1.300827	2.653175
C	-1.192305	-2.856051	0.179960
H	-2.721931	6.369869	-0.441482
C	-2.547233	5.305007	-0.274370
H	-2.054316	-0.391611	2.361825
P	-1.778469	0.764092	0.275579
C	-2.099026	2.567046	0.158959
H	-3.951932	-3.856979	1.922840
C	-3.240894	-3.783544	1.097148
C	-2.015014	4.865112	0.933910
C	-1.794464	3.503229	1.152096
C	-1.411766	-3.591203	-0.995474
H	-1.767926	5.583094	1.718720
H	-0.691090	-3.540725	-1.815545
H	-1.381783	3.191897	2.114822
C	-3.452980	-4.500065	-0.077832
C	-3.465823	0.072709	0.120317

C	-2.532328	-4.405851	-1.122314
H	-3.043990	-1.170776	-1.596754
H	-4.200302	1.212893	1.820168
H	-4.332765	-5.139170	-0.178713
C	-3.798399	-0.847935	-0.878736
C	-4.441297	0.475695	1.047514
H	-2.684616	-4.975284	-2.041477
C	-5.094595	-1.362502	-0.942208
C	-5.727647	-0.050211	0.983208
H	-5.347933	-2.081135	-1.725005
H	-6.480544	0.267296	1.707786
C	-6.055894	-0.971572	-0.013775
H	-7.067985	-1.378780	-0.066877
O	-1.229543	-0.967285	-2.609405
C	-0.045578	-1.157388	-3.028908
H	0.466100	-2.129240	-2.853236
H	1.514997	-0.213970	-1.879028
H	0.335698	-0.595258	-3.904522
H	0.288092	1.351154	-2.409188
H	-0.510958	1.482109	-2.127888

XVI-XVII

H	6.196226	-2.983175	-0.129057
H	4.942244	-2.801967	2.013577
C	5.365735	-2.280111	-0.037132
C	4.664717	-2.179367	1.160530
H	5.564431	-1.531216	-2.056459
C	5.008909	-1.470135	-1.118110
C	3.606593	-1.275601	1.281605
H	3.090763	-1.207014	2.242312
H	5.540418	3.911792	0.772016
H	4.461006	5.718125	-0.561285
H	2.711836	-6.622264	-0.296384
H	0.752499	-6.506673	1.236416
C	3.946644	-0.580492	-1.001738
C	4.559642	3.748708	0.320488
C	3.954398	4.760198	-0.426541
C	3.227290	-0.474857	0.199633
H	4.410905	1.734716	1.063584
C	2.144612	-5.693727	-0.203792
C	3.919566	2.523750	0.486307
C	1.047162	-5.629632	0.656493
H	3.670417	0.045374	-1.856889
C	2.705597	4.542291	-1.003624
H	2.224365	5.328231	-1.589921
H	3.363451	-4.619664	-1.628150
C	2.508766	-4.574897	-0.949451
C	2.660177	2.295254	-0.092673
H	2.228467	0.604443	2.608370
C	2.060817	3.316649	-0.838069
C	0.317748	-4.449782	0.770831
H	-0.551750	-4.414562	1.434700
H	1.087986	-1.810156	2.251826
H	1.077515	3.169796	-1.289365
H	1.011543	-0.473761	4.403986
P	1.812886	0.696132	0.216504
C	1.783127	-3.389528	-0.833406
C	1.348018	0.864670	1.999859
C	0.685049	-3.314986	0.029136
H	2.081389	-2.513514	-1.414367
H	1.203395	1.943139	2.169289
H	0.249230	1.127846	4.428643
C	0.100127	-1.369649	2.048551
C	0.154114	0.106364	4.032819
H	-0.597413	-1.951988	2.671267
C	0.102154	0.105798	2.502106
H	-0.756296	-0.333707	4.465084
P	-0.260484	-1.766184	0.276490
Ru	0.080852	0.047445	-1.150383
H	-2.595218	-1.722202	2.230191
H	-1.857828	5.443789	-2.197095
H	-2.143777	3.018258	-1.810660
C	-1.515854	5.002976	-1.258292
C	-1.675865	3.638766	-1.038039
C	-1.188931	0.838403	2.088794
C	-2.901140	-2.185820	1.289580
H	-1.275983	1.772576	2.664798
C	-1.990077	-2.358028	0.239748
H	-0.801512	6.877099	-0.450048
C	-0.928267	5.806238	-0.278052
H	-2.060684	0.240377	2.393173
P	-1.434778	1.232152	0.293963
C	-1.254772	3.053649	0.167703
H	-4.919613	-2.469140	1.987893
C	-4.221586	-2.614921	1.160744
C	-0.513082	5.237140	0.922291
C	-0.676806	3.868534	1.146313
C	-2.425808	-2.985332	-0.938196
H	-0.056279	5.858507	1.695397
H	-1.727670	-3.148197	-1.762462
H	-0.342439	3.454498	2.100725
C	-4.645283	-3.227247	-0.016037
C	-3.237112	1.011840	0.081405

C	-3.742092	-3.417905	-1.062634
H	-3.072793	-0.173029	-1.719841
H	-3.724839	2.179286	1.849566
H	-5.679486	-3.563452	-0.116497
C	-3.750852	0.283614	-0.996624
C	-4.113965	1.587839	1.014602
H	-4.063992	-3.909616	-1.982932
C	-5.131257	0.127420	-1.131802
C	-5.488590	1.421521	0.876168
H	-5.526970	-0.444968	-1.973891
H	-6.166075	1.872635	1.604340
C	-5.998708	0.689575	-0.198361
H	-7.078214	0.564046	-0.307582
O	-1.282572	-0.728144	-2.676602
C	-0.153689	-1.227637	-3.005054
H	0.057769	-2.300063	-2.792558
H	1.225172	-0.763077	-2.039142
H	0.375667	-0.846357	-3.902247
H	0.642726	1.240003	-2.345332
H	-0.135207	1.522052	-2.128162

XVII

H	-2.760618	6.268432	-0.474472
H	-1.828785	5.486607	1.697233
C	-2.585788	5.204096	-0.304040
C	-2.066501	4.766760	0.911214
H	-3.309712	4.608528	-2.252879
C	-2.890015	4.274767	-1.301499
C	-1.845270	3.406164	1.132930
H	-1.435364	3.096682	2.097550
H	-6.592879	0.226398	1.399578
H	-7.099085	-1.411268	-0.408351
H	2.642766	6.649351	-0.368482
H	4.142955	5.208291	1.000901
C	-2.668690	2.918994	-1.081767
C	-5.801998	-0.106157	0.723991
C	-6.085570	-1.022090	-0.290917
C	-2.141384	2.466864	0.139423
H	-4.308747	1.130979	1.655025
C	2.413820	5.589521	-0.238778
C	-4.512478	0.396198	0.869547
C	3.255395	4.782654	0.528334
H	-2.916693	2.197415	-1.867868
C	-5.074022	-1.432857	-1.155133
H	-5.286427	-2.151467	-1.949794
H	0.624373	5.664306	-1.447730
C	1.285661	5.039515	-0.843390
C	-3.483315	-0.020303	0.009188
H	-1.916757	1.218811	2.649025
C	-3.779066	-0.936939	-1.002703
C	2.972272	3.428945	0.686636
H	3.647327	2.801621	1.276383
H	0.473115	2.068755	2.325774
H	-2.994118	-1.297075	-1.671287
H	-0.354371	0.966958	4.496763
P	-1.797486	0.667246	0.268366
C	0.995148	3.685113	-0.682667
C	-1.545134	0.352442	2.080223
C	1.835397	2.867754	0.082396
H	0.103780	3.267788	-1.157650
H	-2.225749	-0.470628	2.348020
H	-0.916615	-0.715257	4.462574
C	0.899018	1.072273	2.129237
C	-0.144379	-0.030253	4.083837
H	1.795671	1.019962	2.767249
C	-0.123039	-0.004684	2.552768
H	0.821773	-0.360934	4.491944
P	1.458701	1.099431	0.369800
Ru	-0.031086	0.041957	-1.078357
H	3.060151	-0.571762	2.330762
H	-2.406067	-5.124560	-2.270163
H	-0.498758	-3.607583	-1.849531
C	-2.365939	-4.553298	-1.340311
C	-1.295063	-3.697050	-1.102336
C	0.282666	-1.413550	2.076372
C	3.621421	-0.444536	1.402187
H	-0.313753	-2.164902	2.616503
C	3.113791	0.329577	0.350791
H	-4.216205	-5.365414	-0.572797
C	-3.377089	-4.691673	-0.387236
H	1.322661	-1.614328	2.373052
P	0.183053	-1.796041	0.265467
C	-1.218675	-2.963966	0.092837
H	5.246386	-1.664045	2.119504
C	4.867464	-1.060485	1.292081
C	-3.307088	-3.974554	0.804191
C	-2.234327	-3.115609	1.043869
C	3.890105	0.493786	-0.808464
H	-4.093120	-4.078340	1.555286
H	3.514452	1.096099	-1.636806
H	-2.210195	-2.564285	1.986895
C	5.625498	-0.899525	0.134908
C	1.634612	-2.866282	-0.020893

C	5.137629	-0.113683	-0.909747
H	2.278596	-1.808590	-1.797338
H	1.232701	-4.128363	1.703738
H	6.602301	-1.380555	0.049540
C	2.478229	-2.637977	-1.113440
C	1.899252	-3.927902	0.858702
H	5.732813	0.027140	-1.814428
C	3.585705	-3.463395	-1.314233
C	3.008052	-4.743182	0.652270
H	4.245626	-3.278952	-2.164991
H	3.209806	-5.569217	1.337609
C	3.853540	-4.509697	-0.434516
H	4.721951	-5.152379	-0.595402
O	1.485419	-0.011067	-2.556655
C	0.908584	1.108828	-3.015446
H	1.523059	2.035212	-2.944525
H	-0.041101	1.452344	-2.378393
H	0.454771	1.042859	-4.028008
H	-1.279454	-0.549200	-2.239825
H	-0.682603	-1.150176	-2.246240

XVIII

H	-6.649183	-0.655998	-1.002036
H	-5.074814	-1.126691	-2.874569
C	-5.622035	-0.287413	-0.962485
C	-4.742636	-0.548713	-2.009547
H	-5.875726	0.697075	0.945190
C	-5.186168	0.463546	0.130532
C	-3.430562	-0.077346	-1.960429
H	-2.769689	-0.304476	-2.799770
H	-3.039433	5.664825	-1.989474
H	-2.292338	6.849552	0.072459
H	-4.421138	-5.386164	-0.856540
H	-4.623028	-3.220960	0.358402
C	-3.878656	0.938348	0.174814
C	-2.502312	5.121858	-1.208926
C	-2.086369	5.784514	-0.052863
C	-2.975763	0.667309	-0.865231
H	-2.596132	3.253615	-2.267968
C	-3.575660	-4.695746	-0.822219
C	-2.244145	3.763502	-1.365880
C	-3.687509	-3.486147	-0.140448
H	-3.562801	1.557319	1.019057
C	-1.413026	5.079855	0.941237
H	-1.080564	5.589011	1.848598
H	-2.284042	-5.975305	-1.991517
C	-2.378171	-5.026866	-1.458545
C	-1.553109	3.045170	-0.375893
H	-1.445921	1.209560	-3.100456
C	-1.147088	3.719910	0.777735
C	-2.607621	-2.603982	-0.093573
H	-2.702157	-1.655458	0.439375
H	-1.012772	-1.324229	-2.861598
H	-0.594203	3.201435	1.565278
H	-0.248844	0.051098	-4.858485
P	-1.238744	1.251272	-0.664122
C	-1.295527	-4.153239	-1.408489
C	-0.592827	1.282526	-2.408651
C	-1.401967	-2.930847	-0.724698
H	-0.356278	-4.429459	-1.897070
H	-0.188701	2.293392	-2.568362
H	0.910054	1.374986	-4.628916
C	0.016774	-1.190439	-2.497057
C	0.664486	0.347763	-4.322451
H	0.611311	-1.908978	-3.082845
C	0.478103	0.248573	-2.805440
H	1.479066	-0.304939	-4.668583
P	0.018618	-1.769998	-0.737465
Ru	0.102129	-0.000717	0.807480
H	2.486800	-2.336051	-2.418637
H	2.750211	4.751907	2.503976
H	2.611805	2.337331	1.991950
C	2.466360	4.428435	1.500105
C	2.383891	3.070256	1.209903
C	1.837184	0.561307	-2.153333
C	2.489108	-2.962351	-1.523421
H	2.292803	1.442811	-2.632098
C	1.438089	-2.909032	-0.599860
H	2.265640	6.440245	0.733556
C	2.201407	5.373854	0.506982
H	2.539188	-0.260485	-2.361376
P	1.890676	0.814479	-0.320682
C	2.035556	2.630381	-0.077767
H	4.374292	-3.859488	-2.057569
C	3.564478	-3.828538	-1.325814
C	1.868074	4.950575	-0.776527
C	1.789982	3.587907	-1.068329
C	1.480051	-3.748119	0.526183
H	1.668533	5.682829	-1.562042
H	0.668549	-3.718393	1.254792
H	1.542529	3.288848	-2.089519
C	3.601343	-4.649683	-0.201799
C	3.591477	0.261253	0.077294

C	2.554631	-4.609982	0.720831
H	4.501325	1.962307	-0.926854
H	2.986807	-1.512411	1.145220
H	4.442933	-5.328543	-0.047468
C	4.675357	1.024641	-0.389565
C	3.833470	-0.926439	0.777711
H	2.573360	-5.258910	1.598994
C	5.980650	0.598827	-0.163036
C	5.146151	-1.342387	1.005258
H	6.817337	1.198355	-0.528010
H	5.327020	-2.268099	1.556863
C	6.217690	-0.586139	0.536512
H	7.243198	-0.914670	0.719532
O	1.048439	-1.370085	2.041470
C	1.585271	-0.947850	3.236007
H	1.958373	-1.813776	3.819110
H	2.457000	-0.261291	3.122035
H	-0.060845	1.483959	1.767022
H	0.556273	1.057908	2.166054
H	0.859568	-0.422280	3.903078
O	-1.645203	-0.584492	2.238148
C	-1.713723	-1.836755	2.943245
C	-2.131455	0.378078	3.179867
C	-3.093777	-1.794577	3.569647
H	-1.542021	-2.645736	2.224232
H	-0.908262	-1.873469	3.696051
C	-3.316798	-0.300110	3.857831
H	-1.325674	0.611135	3.902191
H	-2.368280	1.301496	2.633208
H	-3.161309	-2.423163	4.466649
H	-3.842884	-2.170315	2.856699
H	-3.339893	-0.074222	4.931970
H	-4.270936	0.050681	3.441735

XVIII-XXIV

H	5.446966	-3.899246	-1.053177
H	4.407327	-2.829960	-3.047012
C	4.433286	-3.497390	-0.994806
C	3.852295	-2.897962	-2.109032
H	4.140871	-4.088468	1.064265
C	3.704445	-3.597084	0.191544
C	2.556420	-2.385118	-2.038126
H	2.130722	-1.933197	-2.936654
H	-2.459784	-5.899523	-1.491888
H	-2.667765	-6.499502	0.916158
H	6.936656	1.315417	-0.901731
H	5.726611	-0.617501	0.108816
C	2.412158	-3.086229	0.260365
C	-2.024883	-5.253329	-0.726274
C	-2.140531	-5.589782	0.621255
C	1.821260	-2.460453	-0.848532
H	-1.250608	-3.872221	-2.172513
C	5.845891	1.316744	-0.849016
C	-1.345593	-4.097063	-1.107611
C	5.169183	0.235741	-0.286131
H	1.844850	-3.190391	1.188924
C	-1.575685	-4.760929	1.589602
H	-1.659157	-5.016654	2.648278
H	5.645452	3.260346	-1.770598
C	5.123550	2.405277	-1.335794
C	-0.792390	-3.245886	-0.141569
H	0.108226	-1.989578	-3.038400
C	-0.915929	-3.595105	1.209185
C	3.777131	0.236955	-0.215108
H	3.260697	-0.601528	0.254890
H	1.744227	0.079996	-2.715947
H	-0.512580	-2.930035	1.978388
H	0.209404	-0.343974	-4.817815
P	0.145598	-1.731076	-0.608819
C	3.731715	2.412642	-1.264118
C	-0.454936	-1.370005	-2.324052
C	3.042394	1.324588	-0.707309
H	3.183726	3.280171	-1.641032
H	-1.491332	-1.736964	-2.377012
H	-1.548652	-0.420728	-4.582572
C	0.921197	0.768555	-2.472930
C	-0.637533	0.124488	-4.294954
H	1.081183	1.635441	-3.132500
C	-0.434014	0.106581	-2.779196
H	-0.732671	1.153193	-4.672256
P	1.199394	1.294841	-0.723801
Ru	-0.040587	0.057454	0.819359
H	0.671684	3.510904	-2.786740
H	-4.509592	-3.214133	1.626106
H	-2.831934	-1.407814	1.621058
C	-4.353233	-2.627919	0.717854
C	-3.407533	-1.605158	0.713264
C	-1.579311	0.916531	-2.132875
C	0.589580	3.905323	-1.770338
H	-2.507452	0.815216	-2.712883
C	0.788091	3.078670	-0.655532
H	-5.820425	-3.715883	-0.436075
C	-5.086089	-2.907584	-0.433302
H	-1.338109	1.989759	-2.186088
P	-1.944681	0.538258	-0.365700
C	-3.179270	-0.834677	-0.434891
H	0.120783	5.887107	-2.478081
C	0.276070	5.254375	-1.601481
C	-4.878621	-2.144685	-1.581063
C	-3.941921	-1.112396	-1.579597
C	0.676786	3.630628	0.629812
H	-5.454806	-2.346829	-2.486484
H	0.800441	2.984924	1.505585
H	-3.819922	-0.528498	-2.494068
C	0.166818	5.793677	-0.319969
C	-3.007691	1.954932	0.128266

C	0.370294	4.979746	0.794267
H	-4.435392	0.727474	1.208694
H	-1.797414	3.482174	-0.839241
H	-0.078029	6.850279	-0.190991
C	-4.147027	1.740464	0.918613
C	-2.677885	3.270408	-0.230630
H	0.280271	5.394597	1.800852
C	-4.930167	2.811847	1.341126
C	-3.467741	4.339209	0.187591
H	-5.814373	2.623833	1.953694
H	-3.196588	5.354762	-0.109787
C	-4.592696	4.114911	0.978962
H	-5.209857	4.953643	1.307631
O	-0.303445	1.331852	2.641611
C	-1.382881	2.150210	2.956525
H	-1.240794	2.583993	3.963024
H	-1.509482	2.995016	2.251562
H	-0.977432	-0.810974	2.113655
H	-0.783311	0.004948	2.505635
H	-2.350470	1.608649	2.977846
O	1.762979	-0.366406	2.158806
C	2.544946	0.716308	2.714104
C	1.645736	-1.285374	3.253475
C	3.666166	0.003669	3.447716
H	2.866315	1.362245	1.888722
H	1.893050	1.298394	3.384062
C	3.046381	-1.349090	3.840101
H	0.909696	-0.886341	3.975353
H	1.263339	-2.237384	2.863504
H	4.014354	0.583406	4.312143
H	4.533358	-0.138568	2.786843
H	3.020765	-1.513344	4.925096
H	3.612453	-2.184101	3.404097

XXIV

H	2.540250	-6.123699	-1.333081
H	1.861635	-4.821554	-3.344967
C	1.903609	-5.242784	-1.227940
C	1.524841	-4.513476	-2.352653
H	1.728975	-5.424638	0.918513
C	1.451249	-4.848654	0.032072
C	0.708430	-3.388691	-2.221245
H	0.432300	-2.842142	-3.126147
H	-5.177081	-3.743352	-1.691647
H	-5.642768	-4.343323	0.679987
H	6.832031	-1.923315	-0.296765
H	4.931696	-3.109045	-1.395639
C	0.637354	-3.727246	0.161795
C	-4.470231	-3.465556	-0.906681
C	-4.730286	-3.802392	0.420162
C	0.262774	-2.971898	-0.959956
H	-3.119036	-2.546010	-2.293805
C	5.866647	-1.426421	-0.413183
C	-3.304204	-2.778338	-1.242441
C	4.804351	-2.088191	-1.027251
H	0.277123	-3.429502	1.150543
C	-3.816455	-3.449472	1.412746
H	-4.010245	-3.712428	2.455210
H	6.518344	0.412177	0.515290
C	5.689911	-0.119854	0.042122
C	-2.392375	-2.396945	-0.249389
H	-1.032815	-1.585741	-3.091797
C	-2.661581	-2.746966	1.079520
C	3.571657	-1.454167	-1.174820
H	2.749491	-2.006509	-1.634924
H	1.418372	-0.745220	-2.722739
H	-1.968323	-2.432326	1.864498
H	-0.120123	-0.131120	-4.804056
P	-0.823376	-1.512851	-0.647876
C	4.460518	0.517922	-0.112277
C	-1.173479	-0.809309	-2.325608
C	3.380481	-0.140802	-0.723284
H	4.346801	1.545875	0.244702
H	-2.249577	-0.578171	-2.347779
H	-1.656961	0.698329	-4.483538
C	1.126180	0.287072	-2.470783
C	-0.588814	0.675219	-4.220508
H	1.691780	0.931588	-3.162021
C	-0.390181	0.461207	-2.718736
H	-0.142141	1.624769	-4.549685
P	1.711992	0.638581	-0.744095
Ru	-0.041862	0.013122	0.817065
H	3.495683	2.091128	-2.612965
H	-5.622737	-0.547694	1.619792
H	-3.225327	0.063029	1.641506
C	-5.183163	-0.057894	0.747855
C	-3.836141	0.295715	0.764765
C	-0.953165	1.708828	-1.984758
C	3.077192	2.827096	-1.919139
H	-1.790580	2.135310	-2.555984
C	2.200868	2.415725	-0.901087
H	-7.014559	-0.091068	-0.398943
C	-5.960769	0.195107	-0.380402
H	-0.185001	2.498452	-1.966828
P	-1.456304	1.398746	-0.237618
C	-3.240421	0.914461	-0.342641
H	4.126579	4.465991	-2.841887
C	3.443090	4.163770	-2.045415
C	-5.384291	0.816949	-1.486274
C	-4.038729	1.181217	-1.465088
C	1.713333	3.379376	-0.013875
H	-5.985044	1.026852	-2.373894
H	1.025526	3.075685	0.777410
H	-3.625845	1.674850	-2.347660
C	2.943301	5.115702	-1.153432
C	-1.629993	3.067444	0.510874

C	2.078754	4.721372	-0.135992
H	-2.038213	2.191719	2.448898
H	-1.302867	4.254301	-1.277761
H	3.232814	6.163948	-1.253021
C	-1.903931	3.124831	1.888306
C	-1.507674	4.264192	-0.204489
H	1.681831	5.457434	0.567263
C	-2.030748	4.351996	2.537750
C	-1.639755	5.490836	0.447235
H	-2.249677	4.381667	3.607555
H	-1.542805	6.417201	-0.123534
C	-1.892529	5.539237	1.817095
H	-1.993982	6.501906	2.322233
O	0.606716	1.408225	2.531441
C	1.958933	1.609609	2.906048
H	2.061598	2.484063	3.562342
H	2.299774	0.725877	3.459191
H	-1.264699	-0.356100	1.835486
H	2.608728	1.745799	2.024217
O	1.215196	-1.305064	2.203181
C	0.628607	-1.788552	3.427430
C	2.594650	-1.717695	2.112391
C	1.582515	-2.849765	3.925822
H	0.526025	-0.943597	4.131430
H	-0.384129	-2.151015	3.201344
C	2.926029	-2.304899	3.467734
H	2.687011	-2.455379	1.297354
H	3.207114	-0.845205	1.842903
H	1.371901	-3.816357	3.440613
H	1.514672	-3.007917	5.009577
H	3.717708	-3.063167	3.414026
H	3.276574	-1.520858	4.158032
H	0.125984	2.249193	2.522874

XXIV-I'

H	2.996521	-5.846057	-0.992878
H	1.588530	-5.253928	-2.958783
C	2.270317	-5.033524	-0.921650
C	1.484349	-4.699510	-2.023644
H	2.726109	-4.589757	1.142637
C	2.117497	-4.330237	0.272854
C	0.553236	-3.663982	-1.937563
H	-0.060628	-3.442547	-2.813788
H	-5.180727	-3.830263	-1.300118
H	-5.491417	-4.404430	1.102389
H	6.931411	-1.776995	-0.588780
H	5.023146	-3.063037	-1.552936
C	1.195892	-3.289963	0.355310
C	-4.425785	-3.542341	-0.565293
C	-4.599105	-3.864126	0.779089
C	0.408122	-2.935547	-0.748926
H	-3.175354	-2.623246	-2.044513
C	5.938743	-1.325977	-0.649012
C	-3.287980	-2.852064	-0.982266
C	4.872147	-2.044684	-1.186965
H	1.088487	-2.725460	1.284007
C	-3.625667	-3.497957	1.709269
H	-3.752365	-3.751743	2.764051
H	6.562737	0.554398	0.211780
C	5.731267	-0.022487	-0.199318
C	-2.316915	-2.460763	-0.051377
H	-0.997163	-1.766979	-2.956884
C	-2.495877	-2.798532	1.296500
C	3.604488	-1.470513	-1.264920
H	2.786822	-2.062566	-1.684022
H	1.417729	-0.819413	-2.753832
H	-1.750616	-2.479848	2.030912
H	-0.199428	-0.377076	-4.774732
P	-0.790922	-1.553134	-0.526681
C	4.466436	0.556501	-0.281396
C	-1.169932	-0.953218	-2.237337
C	3.382344	-0.160626	-0.812357
H	4.326655	1.581632	0.072844
H	-2.253071	-0.757559	-2.268702
H	-1.748990	0.429836	-4.455461
C	1.089543	0.200751	-2.493961
C	-0.674384	0.445509	-4.220160
H	1.620546	0.864358	-3.194583
C	-0.433199	0.316041	-2.715101
H	-0.262052	1.388354	-4.607960
P	1.685053	0.552348	-0.777100
Ru	-0.033933	0.022352	0.839310
H	2.848927	2.489808	-2.788537
H	-5.571531	-0.574347	1.751931
H	-3.181803	0.065173	1.701918
C	-5.157616	-0.104569	0.856709
C	-3.815184	0.265244	0.833434
C	-1.004552	1.583047	-2.029830
C	2.605724	3.029887	-1.868817
H	-1.857653	1.974879	-2.602075
C	2.049853	2.354208	-0.772668
H	-7.013823	-0.190398	-0.246925
C	-5.963275	0.107800	-0.260368
H	-0.246458	2.380819	-2.058686
P	-1.469476	1.345990	-0.263604
C	-3.252387	0.860239	-0.304569
H	3.300480	4.911335	-2.656266
C	2.872855	4.394995	-1.794038
C	-5.419673	0.705262	-1.395974
C	-4.078327	1.085270	-1.416223
C	1.795107	3.072836	0.401655
H	-6.043316	0.884284	-2.274505
H	1.357841	2.558082	1.263598
H	-3.691884	1.560042	-2.320766
C	2.608358	5.100395	-0.617912
C	-1.534609	3.037133	0.434770

C	2.076915	4.435829	0.484955
H	-1.900322	2.251104	2.416579
H	-1.196665	4.142147	-1.407879
H	2.822957	6.169861	-0.563098
C	-1.756720	3.155388	1.815516
C	-1.367007	4.198439	-0.330147
H	1.866274	4.978747	1.409253
C	-1.795820	4.408872	2.420415
C	-1.409895	5.452127	0.278443
H	-1.972006	4.488511	3.495533
H	-1.277158	6.350569	-0.328452
C	-1.618935	5.560670	1.652168
H	-1.651744	6.544594	2.124880
H	-1.229143	-0.240578	1.937536
O	1.327967	-0.738770	2.453850
C	0.840099	-1.406776	3.643591
C	2.773164	-0.679894	2.470742
C	2.066868	-2.028412	4.270429
H	0.363369	-0.652431	4.292113
H	0.065428	-2.124236	3.338580
C	3.158060	-1.040288	3.886638
H	3.164265	-1.397667	1.726562
H	3.075932	0.331064	2.158606
H	2.267937	-3.016875	3.827150
H	1.961235	-2.172258	5.352943
H	4.172775	-1.451630	3.955404
H	3.118382	-0.151374	4.535590
H	0.416083	2.167158	3.282544
H	0.782988	1.501816	3.282007

XIX

H	6.330642	-2.687291	-0.896500
H	5.354491	-1.013132	-2.465088
C	5.257786	-2.484197	-0.884802
C	4.711933	-1.551039	-1.764686
H	4.840297	-3.920078	0.674804
C	4.424128	-3.170150	-0.001580
C	3.343863	-1.290530	-1.746120
H	2.945380	-0.535765	-2.429665
H	-0.419731	-6.347444	-1.595359
H	-1.574708	-6.650258	0.588507
H	6.349316	3.132551	-0.652225
H	5.658525	1.071796	0.566886
C	3.053353	-2.919974	0.005707
C	-0.487564	-5.514344	-0.892247
C	-1.131604	-5.684646	0.335171
C	2.494961	-1.966523	-0.859228
H	0.596592	-4.167892	-2.177084
C	5.303518	2.817782	-0.654628
C	0.077900	-4.284218	-1.219918
C	4.916324	1.668538	0.030154
H	2.407817	-3.497977	0.672812
C	-1.201845	-4.625019	1.237647
H	-1.702750	-4.754859	2.199829
H	4.645844	4.474099	-1.878976
C	4.349385	3.570146	-1.342721
C	-0.008063	-3.208131	-0.325011
H	1.115453	-1.493235	-3.152327
C	-0.643933	-3.390180	0.908152
C	3.580209	1.264927	0.029030
H	3.286068	0.358540	0.561407
H	1.596993	0.977126	-2.947735
H	-0.714415	-2.549540	1.607518
H	0.450930	0.002381	-4.936460
P	0.702815	-1.569001	-0.753351
C	3.014728	3.175568	-1.338945
C	0.226325	-1.304462	-2.531126
C	2.615983	2.015512	-0.653478
H	2.273054	3.783534	-1.866699
H	-0.487906	-2.096414	-2.806322
H	-1.153015	-0.741017	-4.782790
C	0.603122	1.201527	-2.528999
C	-0.522598	0.089134	-4.432851
H	0.298208	2.138015	-3.021177
C	-0.365241	0.067485	-2.909712
H	-0.989047	1.025072	-4.772739
P	0.843439	1.517872	-0.716820
Ru	-0.028185	-0.027055	0.709633
H	-1.205895	3.226493	-2.205596
H	-5.835361	-2.318865	1.521914
H	-4.318456	-0.390023	1.260660
C	-5.041049	-2.357099	0.773409
C	-4.179574	-1.270640	0.629243
C	-1.765463	0.281862	-2.304615
C	-0.915200	3.718861	-1.274294
H	-2.477600	-0.405294	-2.787587
C	0.038566	3.145927	-0.424587
H	-5.579855	-4.326957	0.070773
C	-4.900773	-3.479133	-0.041214
H	-2.115584	1.292641	-2.568012
P	-1.969722	0.105609	-0.462980
C	-3.156962	-1.293478	-0.329891
H	-2.265598	5.361259	-1.623053
C	-1.521878	4.931750	-0.948161
C	-3.900106	-3.506276	-1.011973
C	-3.037443	-2.422970	-1.152752
C	0.365292	3.816319	0.764718
H	-3.787366	-4.374561	-1.664995
H	1.112350	3.389069	1.440952
H	-2.260685	-2.470325	-1.919137
C	-1.182422	5.590566	0.230957
C	-3.014662	1.533239	0.007952

C	-0.235514	5.028848	1.088609
H	-4.407307	1.282955	-1.638488
H	-1.823768	2.021367	1.743174
H	-1.654831	6.542515	0.482706
C	-4.138995	1.874610	-0.757384
C	-2.706054	2.282859	1.148423
H	0.041469	5.541835	2.012172
C	-4.931931	2.960127	-0.392835
C	-3.505876	3.363793	1.515499
H	-5.805592	3.220782	-0.994112
H	-3.254343	3.946012	2.404845
C	-4.615763	3.705381	0.744368
H	-5.240755	4.554179	1.030700
O	-0.830225	-0.249282	2.508290
C	-2.070529	-0.510776	3.057424
H	-2.010222	-0.523609	4.161144
H	-2.837533	0.243217	2.789859
H	-2.471832	-1.496300	2.746113
O	1.771764	-0.186122	2.105820
C	2.047271	0.935356	2.961576
C	1.881177	-1.340088	2.954786
C	3.141357	0.450147	3.911516
H	2.348811	1.770339	2.312987
H	1.116189	1.208061	3.487947
C	3.157678	-1.074407	3.721273
H	0.990291	-1.391320	3.603516
H	1.894812	-2.226335	2.307709
H	2.914612	0.735987	4.947126
H	4.114978	0.896266	3.667775
H	3.202118	-1.628430	4.667453
H	4.025844	-1.384634	3.120211

XX

H	2.236081	-6.269308	1.033754
H	2.674546	-5.270254	-1.204190
C	1.680097	-5.386855	0.709679
C	1.923088	-4.830580	-0.544513
H	0.528000	-5.246463	2.533332
C	0.725425	-4.813099	1.550511
C	1.210681	-3.707578	-0.963748
H	1.432230	-3.283264	-1.946773
H	-4.371484	-4.632225	-1.928335
H	-6.016542	-3.776934	-0.263883
H	5.618046	-4.018570	0.989955
H	3.791818	-3.316036	2.531388
C	0.014301	-3.691707	1.134012
C	-4.089350	-3.833994	-1.238412
C	-5.009376	-3.356311	-0.302958
C	0.243993	-3.130764	-0.132416
H	-2.086549	-3.704248	-2.008039
C	4.917010	-3.237281	0.688107
C	-2.803931	-3.302747	-1.285224
C	3.896759	-2.842751	1.552681
H	-0.739443	-3.250347	1.795359
C	-4.634849	-2.348452	0.581862
H	-5.351454	-1.969506	1.315185
H	5.848952	-2.931769	-1.237724
C	5.044895	-2.631404	-0.562399
C	-2.426007	-2.274636	-0.405483
H	-0.097783	-2.299160	-2.809723
C	-3.350607	-1.804943	0.531782
C	2.999261	-1.849404	1.165821
H	2.189772	-1.549343	1.841107
H	2.135777	-1.099849	-2.660304
H	-3.066597	-1.000097	1.209059
H	0.816719	-1.112279	-4.742523
P	-0.710935	-1.618221	-0.536144
C	4.153023	-1.633176	-0.948492
C	-0.551919	-1.396024	-2.373824
C	3.117669	-1.239644	-0.089273
H	4.281011	-1.147519	-1.920895
H	-1.576725	-1.374900	-2.775479
H	-0.755562	-0.296420	-4.832495
C	1.660658	-0.147317	-2.374507
C	0.254590	-0.230460	-4.403195
H	2.222020	0.638047	-2.904961
C	0.205912	-0.155105	-2.874305
H	0.740886	0.657322	-4.832193
P	1.952339	0.091318	-0.554265
Ru	0.078316	0.102750	0.739898
H	1.588151	2.803396	-1.595914
H	-5.418673	2.265470	1.285708
H	-2.957822	2.464040	1.096562
C	-4.898747	1.783263	0.454466
C	-3.515023	1.891065	0.349637
C	-0.528593	1.150946	-2.505365
C	2.563258	2.773244	-1.104635
H	-1.438639	1.245275	-3.117570
C	3.029818	1.582660	-0.530168
H	-6.708290	0.997755	-0.428165
C	-5.622014	1.076938	-0.507833
H	0.099822	2.007942	-2.795973
P	-1.001050	1.386815	-0.730997
C	-2.831188	1.287389	-0.716855
H	2.943229	4.852571	-1.515776
C	3.327982	3.936009	-1.063024
C	-4.954613	0.482214	-1.576334
C	-3.568565	0.583550	-1.677901
C	4.294507	1.579675	0.074477
H	-5.512716	-0.071071	-2.334695
H	4.689983	0.666173	0.524860
H	-3.071328	0.095332	-2.519873
C	4.579078	3.924440	-0.447192
C	-0.725669	3.168495	-0.400402

C	5.061425	2.742962	0.113271
H	-1.547102	3.830616	-2.299203
H	0.119287	2.824119	1.558836
H	5.181484	4.834453	-0.411638
C	-1.082359	4.131642	-1.355072
C	-0.163061	3.576160	0.814208
H	6.045361	2.722972	0.586514
C	-0.856499	5.482752	-1.102443
C	0.052158	4.930667	1.067509
H	-1.133464	6.228237	-1.850919
H	0.493451	5.241423	2.016975
C	-0.287220	5.883037	0.108014
H	-0.112942	6.943210	0.303697
O	1.057506	0.735141	2.428110
C	2.321279	1.264452	2.686429
H	3.132263	0.600976	2.335313
H	2.470461	2.249628	2.204477
H	2.464981	1.401684	3.771993
O	-1.640095	0.175215	2.162735
O	-0.815697	1.103697	4.020650
H	0.052588	0.993612	3.451665
C	-1.787971	0.635182	3.308528
C	-3.135723	0.697293	3.941504
H	-3.441236	1.748771	4.034873
H	-3.882319	0.159210	3.349634
H	-3.096676	0.296412	4.961447

XX-XXI

H	1.981317	-6.310591	1.107794
H	2.440087	-5.381056	-1.155363
C	1.455469	-5.416067	0.766924
C	1.710214	-4.898242	-0.501660
H	0.321934	-5.193013	2.594000
C	0.527996	-4.789521	1.600358
C	1.036831	-3.759871	-0.942661
H	1.266875	-3.365254	-1.936282
H	-4.638637	-4.360307	-1.959363
H	-6.198489	-3.482309	-0.226546
H	5.422875	-4.245522	1.045129
H	3.671468	-3.375764	2.588402
C	-0.145603	-3.652860	1.162882
C	-4.300041	-3.602291	-1.249778
C	-5.172102	-3.112133	-0.275709
C	0.097335	-3.130695	-0.118061
H	-2.313165	-3.546606	-2.066078
C	4.759092	-3.440623	0.722009
C	-2.990584	-3.134886	-1.311306
C	3.780847	-2.952643	1.587504
H	-0.878882	-3.168844	1.817483
C	-4.725424	-2.158069	0.635036
H	-5.403511	-1.771700	1.400388
H	5.664470	-3.271823	-1.232823
C	4.892454	-2.898133	-0.556678
C	-2.541231	-2.159877	-0.404752
H	-0.215630	-2.298908	-2.817349
C	-3.416352	-1.679170	0.574164
C	2.932067	-1.928203	1.172947
H	2.158301	-1.555151	1.854146
H	2.067103	-1.209604	-2.696929
H	-3.067924	-0.921789	1.276768
H	0.719683	-1.146536	-4.766469
P	-0.798365	-1.592779	-0.546269
C	4.047716	-1.870677	-0.971414
C	-0.628042	-1.375699	-2.381421
C	3.054276	-1.382639	-0.111453
H	4.180720	-1.437401	-1.967501
H	-1.651831	-1.307718	-2.780327
H	-0.812066	-0.254825	-4.831332
C	1.643344	-0.234397	-2.407049
C	0.205779	-0.240155	-4.415706
H	2.236096	0.522468	-2.944754
C	0.182522	-0.169938	-2.885966
H	0.728920	0.624922	-4.847600
P	1.950878	-0.005957	-0.591222
Ru	0.080770	0.078899	0.720975
H	1.688665	2.746968	-1.563744
H	-5.281047	2.347119	1.405902
H	-2.819982	2.477529	1.178223
C	-4.792247	1.885647	0.544799
C	-3.408162	1.954460	0.418561
C	-0.481140	1.169920	-2.501033
C	2.671543	2.659474	-1.095762
H	-1.387205	1.318560	-3.108289
C	3.094746	1.435509	-0.558549
H	-6.640541	1.192117	-0.334024
C	-5.553964	1.241865	-0.431680
H	0.192036	1.993198	-2.788808
P	-0.934892	1.427794	-0.724941
C	-2.764275	1.374296	-0.685232
H	3.138661	4.728334	-1.468399
C	3.489375	3.785172	-1.043557
C	-4.925618	0.670605	-1.536011
C	-3.539103	0.732950	-1.660457
C	4.368726	1.362159	0.022052
H	-5.514900	0.166182	-2.304774
H	4.730387	0.422256	0.445847
H	-3.072816	0.265032	-2.531163
C	4.749809	3.703431	-0.452290
C	-0.603638	3.200045	-0.406981

C	5.187855	2.489031	0.072934
H	-1.297291	3.868163	-2.355275
H	0.105607	2.856059	1.604889
H	5.393257	4.584456	-0.407830
C	-0.873622	4.162874	-1.390318
C	-0.097275	3.604066	0.833038
H	6.178091	2.414209	0.527248
C	-0.614875	5.508342	-1.139370
C	0.151126	4.953116	1.083897
H	-0.825110	6.253443	-1.909626
H	0.544218	5.260039	2.055420
C	-0.099460	5.904194	0.096325
H	0.099705	6.960215	0.290413
O	1.147798	0.792584	2.422965
C	2.399272	1.377869	2.687179
H	3.210441	0.691056	2.400706
H	2.538825	2.324390	2.136200
H	2.495818	1.584341	3.763409
O	-1.551951	0.128790	2.161292
O	-0.716601	1.326265	3.858136
H	0.369303	1.087258	3.149537
C	-1.659151	0.726575	3.278345
C	-3.001846	0.721140	3.945871
H	-3.335703	1.756343	4.098204
H	-3.755374	0.175529	3.368060
H	-2.914741	0.280311	4.947103

XXI

H	1.376387	-6.458055	1.117041
H	1.931578	-5.588181	-1.147827
C	0.935510	-5.521810	0.767791
C	1.244651	-5.036845	-0.501816
H	-0.185930	-5.187047	2.585154
C	0.062487	-4.809469	1.590996
C	0.680055	-3.844988	-0.954332
H	0.948447	-3.480121	-1.949739
H	-5.008676	-3.892850	-2.070221
H	-6.508828	-2.888793	-0.352946
H	4.975403	-4.732991	1.104104
H	3.301981	-3.689543	2.626028
C	-0.502202	-3.618697	1.143305
C	-4.612948	-3.176271	-1.347279
C	-5.451782	-2.615504	-0.382206
C	-0.203449	-3.128773	-0.138872
H	-2.616646	-3.300412	-2.131674
C	4.397310	-3.868116	0.771452
C	-3.264509	-2.833356	-1.383168
C	3.462842	-3.283191	1.625184
H	-1.191632	-3.064916	1.789968
C	-4.932773	-1.715678	0.545128
H	-5.582927	-1.274504	1.305220
H	5.336124	-3.796691	-1.173418
C	4.597121	-3.346291	-0.507206
C	-2.740378	-1.912969	-0.459629
H	-0.412704	-2.249747	-2.846486
C	-3.584131	-1.361135	0.509534
C	2.724628	-2.181152	1.198322
H	1.988353	-1.728780	1.873775
H	1.971103	-1.387442	-2.686881
H	-3.175436	-0.653582	1.230237
H	0.650665	-1.169382	-4.772489
P	-0.948507	-1.516222	-0.574076
C	3.861052	-2.243319	-0.934737
C	-0.734916	-1.295058	-2.402117
C	2.911879	-1.656540	-0.086983
H	4.045399	-1.829532	-1.930889
H	-1.742853	-1.118635	-2.809085
H	-0.788265	-0.134552	-4.836177
C	1.640450	-0.376882	-2.396582
C	0.223043	-0.222265	-4.413681
H	2.305837	0.321120	-2.929065
C	0.195302	-0.168994	-2.883170
H	0.829798	0.593771	-4.831363
P	1.951815	-0.180421	-0.578328
Ru	0.082628	0.042932	0.722499
H	1.983528	2.575316	-1.579309
H	-4.964253	2.784060	1.512068
H	-2.504856	2.650445	1.270910
C	-4.533442	2.309458	0.627377
C	-3.150481	2.232667	0.491023
C	-0.337345	1.224128	-2.485264
C	2.939902	2.398053	-1.082946
H	-1.225122	1.464967	-3.089830
C	3.228567	1.143394	-0.527684
H	-6.451298	1.861383	-0.262076
C	-5.366378	1.796540	-0.368658
H	0.413301	1.977689	-2.771567
P	-0.755564	1.521446	-0.705660
C	-2.581312	1.640598	-0.647273
H	3.613408	4.409816	-1.453270
C	3.860118	3.440719	-1.013941
C	-4.810393	1.207695	-1.501899
C	-3.425737	1.127538	-1.639896
C	4.473850	0.952658	0.087769
H	-5.455530	0.801297	-2.283791
H	4.732199	-0.014847	0.525225
H	-3.018165	0.648957	-2.534120
C	5.090678	3.243057	-0.387913
C	-0.258319	3.256812	-0.400477

C	5.395811	1.996294	0.155266
H	-0.724532	3.955964	-2.406810
H	0.222212	2.893356	1.672670
H	5.814546	4.058510	-0.330907
C	-0.349012	4.222021	-1.414350
C	0.182342	3.635719	0.872031
H	6.362192	1.830203	0.636026
C	0.026674	5.539094	-1.160219
C	0.551484	4.956356	1.124074
H	-0.045767	6.285260	-1.954367
H	0.890749	5.242086	2.121995
C	0.483200	5.906457	0.106836
H	0.775654	6.940255	0.302330
O	1.182069	0.784857	2.444832
C	2.442461	1.367178	2.700055
H	3.232431	0.638967	2.472129
H	2.613010	2.271160	2.090712
H	2.521173	1.635903	3.762703
O	-1.493328	0.021880	2.180803
O	-0.860719	1.595751	3.646336
H	0.440637	1.200680	3.025701
C	-1.684186	0.768288	3.207412
C	-3.014726	0.598852	3.883165
H	-3.816111	0.925359	3.204385
H	-3.201182	-0.462130	4.094362
H	-3.069366	1.177631	4.810262

XXII

H	3.977142	-5.446254	0.769971
H	3.972682	-4.338588	-1.460332
C	3.180053	-4.747455	0.506774
C	3.176030	-4.131853	-0.742169
H	2.154705	-4.954415	2.399509
C	2.161084	-4.470194	1.420627
C	2.155815	-3.242597	-1.081133
H	2.184283	-2.767743	-2.065454
H	-2.838336	-5.859074	-1.400007
H	-4.799112	-5.170905	-0.027826
H	6.970304	-2.024817	0.452306
H	5.120395	-2.317230	2.097393
C	1.143020	-3.582423	1.086019
C	-2.831527	-4.900677	-0.876502
C	-3.929977	-4.514210	-0.105924
C	1.129398	-2.959435	-0.172777
H	-0.851751	-4.393544	-1.555303
C	6.001961	-1.550167	0.280372
C	-1.717807	-4.071189	-0.968170
C	4.969659	-1.711154	1.201353
H	0.348512	-3.372004	1.810196
C	-3.911531	-3.294712	0.566574
H	-4.769504	-2.987641	1.169912
H	6.604521	-0.631767	-1.581575
C	5.796398	-0.773854	-0.860927
C	-1.698107	-2.836748	-0.298833
H	0.382817	-2.258236	-2.784647
C	-2.801943	-2.454793	0.468756
C	3.731796	-1.108067	0.980206
H	2.932223	-1.263694	1.707963
H	2.387523	-0.559266	-2.606407
H	-2.798754	-1.487575	0.971811
H	1.137352	-0.945415	-4.665906
P	-0.224595	-1.762060	-0.479310
C	4.562330	-0.168039	-1.079303
C	-0.199844	-1.452606	-2.311833
C	3.510152	-0.338339	-0.164630
H	4.426693	0.456308	-1.967641
H	-1.230192	-1.621363	-2.658680
H	-0.575261	-0.504741	-4.801975
C	1.702664	0.256108	-2.324271
C	0.386648	-0.201164	-4.363910
H	2.075423	1.147912	-2.854511
C	0.292083	-0.089136	-2.838787
H	0.670494	0.758707	-4.819174
P	1.917215	0.517255	-0.497704
Ru	0.034407	0.118265	0.752563
H	1.189471	3.068022	-1.902638
H	-5.876773	0.656629	1.036785
H	-3.570430	1.517009	1.014949
C	-5.217473	0.405550	0.202642
C	-3.915939	0.898633	0.185998
C	-0.702000	1.053135	-2.535253
C	1.856807	3.300059	-1.069186
H	-1.569088	0.988064	-3.209449
C	2.420773	2.272447	-0.302460
H	-6.703554	-0.781624	-0.825300
C	-5.681814	-0.396361	-0.840413
H	-0.215437	2.003422	-2.799663
P	-1.315347	1.198836	-0.803775
C	-3.050859	0.595897	-0.878016
H	1.689194	5.423247	-1.401058
C	2.136968	4.637404	-0.788728
C	-4.837250	-0.694080	-1.906197
C	-3.533025	-0.201579	-1.925210
C	3.280475	2.615421	0.753480
H	-5.190719	-1.313992	-2.732752
H	3.727449	1.829278	1.368382
H	-2.902944	-0.452783	-2.780723
C	2.987815	4.965697	0.265141
C	-1.537426	2.993416	-0.508382

C	3.562732	3.950477	1.031519
H	-1.472406	3.658380	-2.577208
H	-1.735635	2.657974	1.615195
H	3.210530	6.011883	0.484506
C	-1.568027	3.952766	-1.528906
C	-1.712873	3.410599	0.820645
H	4.239179	4.200037	1.851849
C	-1.735497	5.302303	-1.221851
C	-1.881277	4.760665	1.124443
H	-1.759004	6.040071	-2.026902
H	-2.020671	5.071314	2.162718
C	-1.881432	5.710639	0.103466
H	-2.010143	6.768953	0.339097
O	0.622868	1.895179	2.084708
C	0.240909	2.020434	3.446371
H	0.886788	2.746770	3.959542
H	-0.813019	2.320785	3.560431
H	0.372876	1.038650	3.915061
O	-0.776970	-1.396591	3.390121
O	-1.679816	0.148460	2.046467
C	-1.699340	-0.632736	3.075157
H	0.591857	2.760013	1.648910
H	0.450861	-0.913608	2.287190
H	1.175200	-0.682198	1.880143
C	-2.967700	-0.582477	3.880128
H	-3.743028	-1.159251	3.354296
H	-2.829031	-1.024687	4.871622
H	-3.342749	0.444848	3.968709

XXII-XXIII

H	5.046873	-4.523690	0.502307
H	4.733408	-3.368371	-1.681161
C	4.112279	-4.000750	0.287907
C	3.936531	-3.357617	-0.934209
H	3.215367	-4.486713	2.194506
C	3.088174	-3.977206	1.236872
C	2.741081	-2.693038	-1.210605
H	2.634523	-2.193812	-2.177186
H	-1.507889	-6.370924	-1.275010
H	-3.630941	-6.057418	-0.011734
H	7.184386	-0.682027	0.472522
H	5.414946	-1.309282	2.111483
C	1.897660	-3.310715	0.964244
C	-1.727917	-5.410988	-0.803030
C	-2.917624	-5.234534	-0.094186
C	1.709584	-2.660047	-0.265659
H	0.125884	-4.523649	-1.443214
C	6.145870	-0.393073	0.297134
C	-0.812179	-4.367374	-0.901376
C	5.157229	-0.741283	1.214702
H	1.100537	-3.302288	1.714792
C	-3.188504	-4.009407	0.510382
H	-4.118750	-3.863604	1.065492
H	6.577561	0.620432	-1.563003
C	5.806130	0.332737	-0.845475
C	-1.082010	-3.127440	-0.299061
H	0.714708	-2.125582	-2.830904
C	-2.277479	-2.957555	0.407026
C	3.830531	-0.375025	0.990121
H	3.061456	-0.665930	1.709588
H	2.416542	-0.044991	-2.610292
H	-2.504658	-1.994755	0.866419
H	1.263947	-0.659887	-4.666090
P	0.126866	-1.759428	-0.496556
C	4.482330	0.701367	-1.069278
C	0.029692	-1.429598	-2.322179
C	3.477738	0.340496	-0.157179
H	4.236570	1.288977	-1.959179
H	-0.974132	-1.757744	-2.631940
H	-0.500787	-0.550762	-4.805930
C	1.584028	0.611928	-2.312977
C	0.384962	-0.073518	-4.361912
H	1.770528	1.569022	-2.827644
C	0.266490	0.006245	-2.836580
H	0.482692	0.926217	-4.809435
P	1.751816	0.883013	-0.481167
Ru	0.000425	0.118034	0.754201
H	0.504330	3.253996	-1.818767
H	-5.869464	-0.464899	1.135811
H	-3.758959	0.802118	1.078753
C	-5.201015	-0.571264	0.278245
C	-4.012576	0.151856	0.240340
C	-0.928360	0.933922	-2.527670
C	1.136799	3.604944	-0.999997
H	-1.768510	0.701809	-3.199449
C	1.913595	2.699925	-0.265451
H	-6.475444	-1.987589	-0.744581
C	-5.542321	-1.421019	-0.774649
H	-0.636331	1.960833	-2.792609
P	-1.541325	0.952427	-0.788137
C	-3.139389	0.040187	-0.854104
H	0.544899	5.656908	-1.292698
C	1.155778	4.968103	-0.704868
C	-4.689333	-1.534507	-1.868783
C	-3.498275	-0.809721	-1.908878
C	2.717602	3.193333	0.774353
H	-4.948527	-2.190338	-2.702538
H	3.327741	2.503780	1.364338
H	-2.859439	-0.921048	-2.787447
C	1.954195	5.445525	0.333137
C	-2.118570	2.672974	-0.506936

C	2.738589	4.554735	1.067805
H	-2.213958	3.309195	-2.583127
H	-2.221327	2.341425	1.624501
H	1.972465	6.512514	0.564480
C	-2.355334	3.592740	-1.537069
C	-2.361804	3.067573	0.818082
H	3.374995	4.923374	1.875250
C	-2.791439	4.884195	-1.244605
C	-2.801025	4.358339	1.108889
H	-2.973291	5.589542	-2.058517
H	-2.990264	4.648562	2.145124
C	-3.005494	5.273655	0.077126
H	-3.345833	6.286514	0.301588
O	0.214105	1.973944	2.080804
C	-0.134700	2.046803	3.452218
H	0.351115	2.910100	3.928480
H	-1.224456	2.109237	3.606236
H	0.233375	1.132991	3.932509
O	-0.290354	-1.531579	3.375251
O	-1.655344	-0.295067	2.123215
C	-1.434622	-1.063485	3.105766
H	0.019776	2.813997	1.638705
H	0.500754	-0.974212	2.495614
H	1.148075	-0.539588	1.912561
C	-2.586247	-1.466179	3.968446
H	-2.873880	-2.493672	3.703235
H	-2.298639	-1.484450	5.025755
H	-3.452166	-0.813837	3.814832

XXIII

H	5.903608	-3.423660	0.299792
H	5.289636	-2.314683	-1.843739
C	4.875577	-3.099216	0.124547
C	4.532199	-2.482081	-1.074751
H	4.156545	-3.800042	2.039587
C	3.898386	-3.305409	1.100587
C	3.217329	-2.071715	-1.300971
H	2.978854	-1.593055	-2.254163
H	-0.095708	-6.577903	-1.132780
H	-2.329018	-6.663949	-0.033355
H	7.183814	0.706861	0.489616
H	5.572931	-0.273294	2.117668
C	2.590988	-2.885853	0.878324
C	-0.541207	-5.667963	-0.725016
C	-1.792664	-5.715668	-0.109310
C	2.231830	-2.262211	-0.326664
H	1.134788	-4.442085	-1.289445
C	6.108658	0.792089	0.317244
C	0.148808	-4.462026	-0.815816
C	5.207419	0.246394	1.228854
H	1.832872	-3.060215	1.648745
C	-2.353172	-4.549780	0.407419
H	-3.335711	-4.576623	0.885909
H	6.333793	1.897750	-1.526787
C	5.633419	1.455582	-0.814906
C	-0.408441	-3.281590	-0.297411
H	1.051324	-1.944659	-2.849664
C	-1.667630	-3.338241	0.311680
C	3.834431	0.352722	1.008798
H	3.127375	-0.084622	1.718698
H	2.373808	0.451755	-2.585728
H	-2.127161	-2.428390	0.700623
H	1.374529	-0.366906	-4.638358
P	0.493310	-1.687436	-0.491860
C	4.262845	1.563653	-1.035334
C	0.280464	-1.371942	-2.310391
C	3.349397	1.002063	-0.129327
H	3.905982	2.104552	-1.917332
H	-0.666980	-1.859755	-2.586886
H	-0.377953	-0.592116	-4.794388
C	1.429930	0.928580	-2.278476
C	0.397977	0.041197	-4.339936
H	1.426677	1.910873	-2.779178
C	0.253302	0.087786	-2.815305
H	0.308850	1.044110	-4.782414
P	1.549031	1.201765	-0.441510
Ru	-0.006719	0.107657	0.771696
H	-0.120648	3.307415	-1.761132
H	-5.710630	-1.520433	1.131026
H	-3.863829	0.109041	1.077184
C	-5.039155	-1.495463	0.269399
C	-4.000690	-0.569587	0.233103
C	-1.097990	0.774591	-2.517197
C	0.424302	3.761542	-0.930093
H	-1.870620	0.392491	-3.201804
C	1.356205	3.012679	-0.200562
H	-6.044186	-3.107800	-0.764440
C	-5.229024	-2.381610	-0.791915
H	-0.998929	1.838835	-2.777873
P	-1.718023	0.675341	-0.780615
C	-3.130570	-0.505882	-0.867695
H	-0.555498	5.663357	-1.196544
C	0.172751	5.096342	-0.612332
C	-4.376416	-2.326543	-1.890775
C	-3.337036	-1.396467	-1.929265
C	2.038032	3.634167	0.857404
H	-4.517877	-3.009821	-2.730842
H	2.764294	3.064967	1.444531
H	-2.694140	-1.380760	-2.812050
C	0.851530	5.700864	0.444349
C	-2.616584	2.260156	-0.521006

C	1.788525	4.967172	1.174270
H	-2.809863	2.852717	-2.602487
H	-2.674367	1.940006	1.615547
H	0.657858	6.746131	0.694036
C	-3.010592	3.113590	-1.560268
C	-2.940671	2.616982	0.797981
H	2.332204	5.438192	1.996278
C	-3.680853	4.304485	-1.284108
C	-3.615470	3.805834	1.073344
H	-3.982303	4.957023	-2.106614
H	-3.863988	4.064673	2.105298
C	-3.976916	4.658808	0.031671
H	-4.501315	5.592759	0.243441
O	-0.213296	1.955168	2.094581
C	-0.451802	1.966818	3.487738
H	-0.121808	2.916859	3.932251
H	-1.513655	1.800092	3.734502
H	0.142188	1.156528	3.926700
O	-0.071159	-1.848245	3.352537
O	-1.562577	-0.682404	2.175003
C	-1.295421	-1.492073	3.072940
H	-0.611718	2.731968	1.674577
H	0.529804	-1.303877	2.736234
H	1.183939	-0.333891	1.873646
C	-2.348378	-2.148529	3.888607
H	-2.369946	-3.218894	3.640360
H	-2.111987	-2.082893	4.957483
H	-3.330451	-1.712205	3.685863
